# Identification of miRNA, lncRNA and mRNA-associated ceRNA networks and potential biomarker for MELAS with mitochondrial DNA A3243G mutation

**DOI:** 10.1038/srep41639

**Published:** 2017-01-31

**Authors:** Wei Wang, Qianqian Zhuang, Kunqian Ji, Bing Wen, Pengfei Lin, Yuying Zhao, Wei Li, Chuanzhu Yan

**Affiliations:** 1 Laboratory of Neuromuscular Disorders and Department of Neurology, Qilu Hospital, Shandong University, Jinan, China; 2 School of Bioengineering, Qilu University of Technology, Jinan, China; 3 Key Laboratory for Experimental Teratology of the Ministry of Education, Brain Science Research Institute, Department of Neurology, Qilu Hospital, Shandong University, Jinan, China

## Abstract

Researchers in the field of mitochondrial biology are increasingly unveiling of the complex mechanisms between mitochondrial dysfunction and noncoding RNAs (ncRNAs). However, roles of ncRNAs underlying mitochondrial myopathy remain unexplored. The aim of this study was to elucidate the regulating networks of dysregulated ncRNAs in Mitochondrial myopathy, Encephalopathy, Lactic Acidosis, and Stroke-like episodes (MELAS) with mitochondrial DNA (mtDNA) A3243G mutation, which might make contributions to the unveiling of the complex mechanisms underlying mitochondrial myopathy and, possibly, new tools applicable to clinical practice. Through high-throughput technology followed by quantitative real-time polymerase chain reaction (qRT-PCR) and bioinformatics analyses, for the first time, we found that the dysregulated muscle miRNAs and lncRNAs between 20 MELAS patients with mtDNA A3243G mutation and 20 controls formed complex regulation networks and participated in immune system, signal transduction, translation, muscle contraction and other pathways in discovery and training phase. Then, selected ncRNAs were validated in muscle and serum in independent validation cohorts by qRT-PCR. Finally, ROC curve analysis indicated reduced serum miR-27b-3p had the better diagnosis value than lactate and might serve as a novel, noninvasive biomarker for MELAS. Follow-up investigation is warranted to better understand roles of ncRNAs in mitochondrial myopathy pathogenesis.

Mitochondrial diseases are heterogeneous group with varying clinical features, showing tissue-specific manifestations and affecting multiple organ systems^1^,^2^. Although earlier considered to be a rare class of disorders, the recent epidemiological studies suggested approximately 1 in 3000–5000 people worldwide being affected by mitochondrial diseases^3^. A large number of studies have explored the role of mtDNA genetics in both inherited metabolic diseases and many other disorders, such as diabetes, cancer, neurodegenerative and cardiovascular diseases, which have been reported to be associated with mitochondrial dysfunction^4^,^5^. Numerous pathogenic mtDNA mutations responsible for mitochondrial disease have been identified, such as the most prevalent mtDNA A3243G mutation, the well-recognized phenotype of which is MELAS syndrome^6^. Although there is increasing clarification of the primary aberrant cellular processes responsible for the respiratory chain dysfunction, the underlying mechanisms of many mitochondrial disorders are still not fully understood. In addition, comparatively little has been accomplished to date in the field of mitochondrial epigenetics^7^. The complexity of the genetics, epigenetics and clinical variability makes mitochondrial disease a particularly challenging area of medicine, not only in proper diagnosis but also in the development of effective therapeutic approaches^1^. Improved understanding of mitochondrial epigenetics to mitochondrial dysfunction promises to deepen our understanding of mitochondrial disease pathogenesis, nominate new biomarkers, and motivate new therapeutic strategies.

The microRNAs and lncRNAs are non-coding RNAs that have been implicated as fine-tuning regulators controlling diverse biological processes at the level of post-transcriptional repression of their target genes in a sequence-specific manner^8^. At present, ncRNAs have become an integral component in understanding of the post-transcriptional mechanisms of disease. Nearly 2000 different miRNAs and more than 50,000 lncRNAs have been identified within the human genome with a constant increase in their number^9^,^10^. However, only a very limited number of nRNAs have biological function annotations. There have been accumulating evidences at remendous increase describing the involvement of ncRNAs regulating various biological and pathological processes in the past 10 years^11^. Researchers in the field of mitochondrial biology are increasingly incorporating these concepts into their studies and discovering a “gold mine” of regulatory interactions between mitochondrial function and ncRNAs biology^12^. An emerging body of research points to close interactions between miRNA and mitochondria^13^. Several studies have documented the localization of various miRNAs and their processing machinery in mitochondria, and that uncoupling of mitochondria decreased cellular miRNA functional efficiency^13^,^14^. It is noteworthy that miRNAs are the only class of relatively well-investigated ncRNAs in mitochondrial disorders until now, with previous studies profiling their dysregulation involved in metabolism and redox signaling, regulating mitochondrial gene expression, and association with occurrence and progression of varieties of mitochondrial disorders^14^,^15^. LncRNAs have also been implicated in mitochondrial function, such as previously mitochondrial-associated RMRP^16^, MDRL^17^, TGFB2-OT1^18^, CARL^19^, and UCA1^20^. But so far, the significance of ncRNAs in mitochondrial myopathy is sparsely characterized. Mitochondria-associated miRNAs, lncRNAs and their regulatory networks need to be specifically and thoroughly investigated in mitochondrial disease. In the present study we aimed to arouse the attention of ncRNAs regulation in studying mitochondrial myopathy, which might make contributions to the unveiling of the complex mechanisms underlying mitochondrial myopathy and, possibly, new tools applicable to clinical practice.

Through high-throughput technology, qRT-PCR and bioinformatics analyses, we performed, for the first time, one systematic and integrative analysis of the potential ncRNA-mRNA regulatory networks in muscle biopsies between MELAS patients with mtDNA A3243G mutation and controls. We found that the deregulated ncRNAs caused by mtDNA A3243G mutation formed complex regulation networks and participated in immune system, signal transduction, translation, muscle contraction and other pathways. Among these dysregulated ncRNAs, we found miR-27b-3p reduced in muscle and serum of MELAS patients with A3243G mutation or non-A3243G mutation, which was negatively associated with lactate, NMADS and the mutation load in muscle, had the better diagnosis value for MELAS than lactate. These results will enrich the genome-wide analysis of RNA molecules with potential cross-talk, provide the rationale for further confirmation studies in larger prospective cohorts and contribute to further systematic studies of mitochondrial disorders.

## Results

### Study population and design

**Figure 1:**
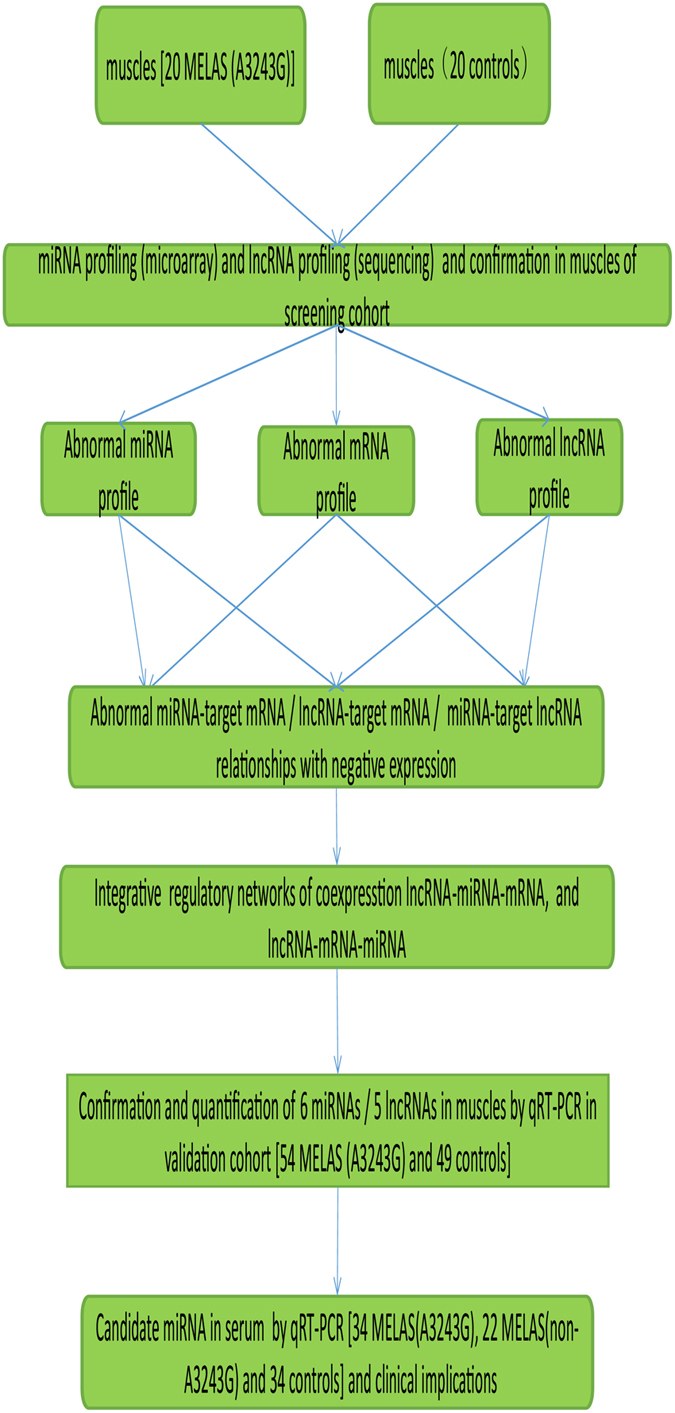
Flow-chat of the study design. First, differentially expressed RNAs in muscle were identified through high-throughout profiling. Then bioinformatics analysis was used to annotate the biological function and construct complex regulation networks between them. Furthermore, the selected meaningful RNAs were validated by qRT-PCR and then we searched their potential clinical implications in independent validation cohorts.

The study population comprised a total of 74 MELAS patients with A3243G mutation, 22 MELAS patients with non-A3243G mutation and 69 controls without neuromuscular diseases. The demographic details are in Supplementary Table S1. This multistage, case-control study consisted of a preliminary step of high-throughput screening, secondary validation and quantification of candidate ncRNAs and mRNAs. In the primary screening, pooled muscle samples were from 20 MELAS patients with A3243G mutation and 20 normal controls for miRNA microarray analysis and lncRNA sequencing. The secondary screening of the most promising RNAs used the individual samples of the primary screening. The validation stage of selected ncRNAs was in a larger cohort of 54 MELAS patients with A3243G mutation and 49 age- and gender- matched normal controls. In addition, 34 MELAS with A3243G mutation from the validation cohort, 22 MELAS with non-A3243G mutation and 34 age- and sex-matched controls were employed to test the promising RNAs in serum. An overview of the study design is illustrated in Fig. 1.

### Profiling the distinct miRNAs, lncRNAs and mRNAs caused by mtDNA A3243G mutation in muscle biopsies

**Figure 2:**
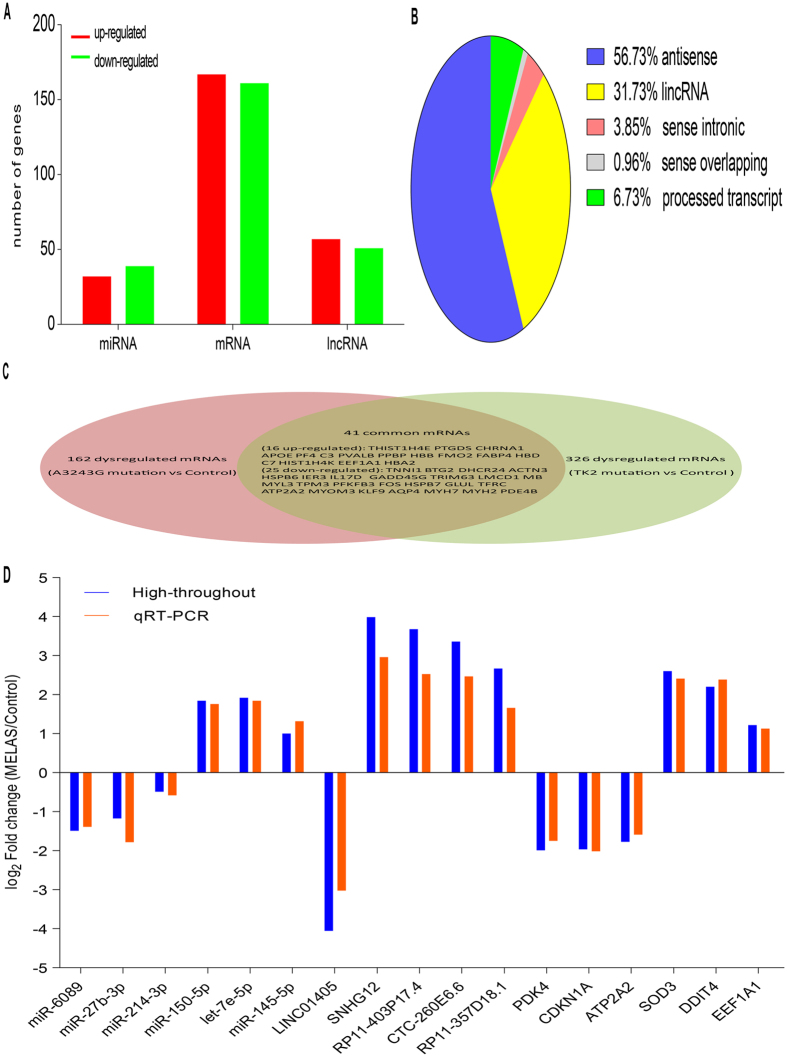
Dysregulated RNAs in muscle biopsies of MELAS patients vs. controls detected with microarray and Solexa sequencing in discovery phase. (**A**) Numbers of the dysregulated RNAs. (**B**) Categorization of the dysregulated lncRNAs. (**C**) Comparison of dysregulated mRNAs of mitchondrial myopathy caused by mtDNA A3243G mutation and TK2 mutation. (**D**) QRT-PCR validation of the selected differential miRNAs, lncRNAs, mRNAs.

Firstly, the genome wide miRNA, lncRNA and mRNA expression profiles in muscle biopsies were detected in discovery phase (including 20 MELAS patients with A3243G mutation and 20 matched controls). The criteria of corrected p-value < 0.05 and absolute fold change >2 was used to identify the significantly dysregulated RNAs. The complete lists of dysregulated genes between two groups are shown in Supplementary Table S2. By microarry profiling, there were 31 and 41 miRNAs significantly up- and down-regulated in MELAS vs. controls, respectively (Fig. 2A, Supplementary Table S2A). By sequencing profiling, 57 and 52 lncRNAs were identified significantly up- and down-regulated by A3243G mutation, respectively (Fig. 2A, Supplementary Table S2B). Meanwhile, categorization of lncRNAs indicated that more antisense and intergenic than intronic and processed transcripts were dysregulated (Fig. 2B). Using the same sequencing profiling with lncRNAs, we found 167 and 162 mRNAs up- and down-regulated in muscle in MELAS patients vs. controls (Fig. 2A, Supplementary Table S2C). In addition, mRNA profiles were compared to that of mitochondrial myopathy with TK2 mutation (GSE43698). When compared with normal subjects, differential gene expression profiling in muscle tissue was identified between TK2 and mtDNA A3243G mutation. Interestingly, 41 dysregulated mRNAs were common between two kinds of mitchondrial myopathy (Fig. 2C), while others seemed to be specific to the given phenotype. For example, compared patients with TK2 and mtDNA A3243G mutation to normal controls, 16 mRNAs, including EEF1A1, C3 and FMO2 were commonly upregulated; 25 mRNAs, including MYC, KLF9, PDE4B, ATP2A2, and HSPB6 were commonly down-regulated. This suggested that different kinds of mutation just caused partly common pathogenesis of mitochondrial myopathy.

### Technical validation of the dysregulated RNAs by qRT-PCR using a training set

The qRT-PCR was not only used to ensure the abnormal gene expression profiles detected by the initial high-throughout step not technical specific, but also to better define and select the meaningful ncRNAs for further analysis. MiRNAs/lncRNAs that met the following criteria were selected for subsequent qRT-PCR validation. Firstly, meaningful ncRNAs should be dysregulated RNAs showing fold-change (log_2_) >1.5 or <−1.5 (*P* < 0.01 and *P* ≠ 0) and having at least 30 copies/500 signal abundantly expressed in either group. Besides, the meaningful ncRNAs were selected from focus of the following network of miRNA-mRNA-lncRNA or lncRNA-miRNA-mRNA. Although seldomly selected, the mRNAs for subsequent validation should be potential targets of the selected miRNAs or lncRNAs. Corresponding to the above features, we screened out subsets of 6 miRNAs (including miR-6089, miR-27b-3p, miR-214-3p, miR-150-5p, let-7e-5p and miR-145-5p), 5 lncRNAs (including LINC01405, SNHG12, RP11-403P17.4, CTC-260E6.6 and RP11-357D18.1) and 6 mRNAs (including PDK4, CDKN1A, ATP2A2, SOD3, DDIT4 and EEF1A1) for qRT-PCR confirmation. Detailed characteristics of these RNAs are available in Supplementary Table S3. As shown in Fig. 2D, the findings were all consistent with results from our high-throughput data.

### The miRNA, mRNA and lncRNA regulatory networks in MELAS with mtDNA A3243G mutation

**Figure 3:**
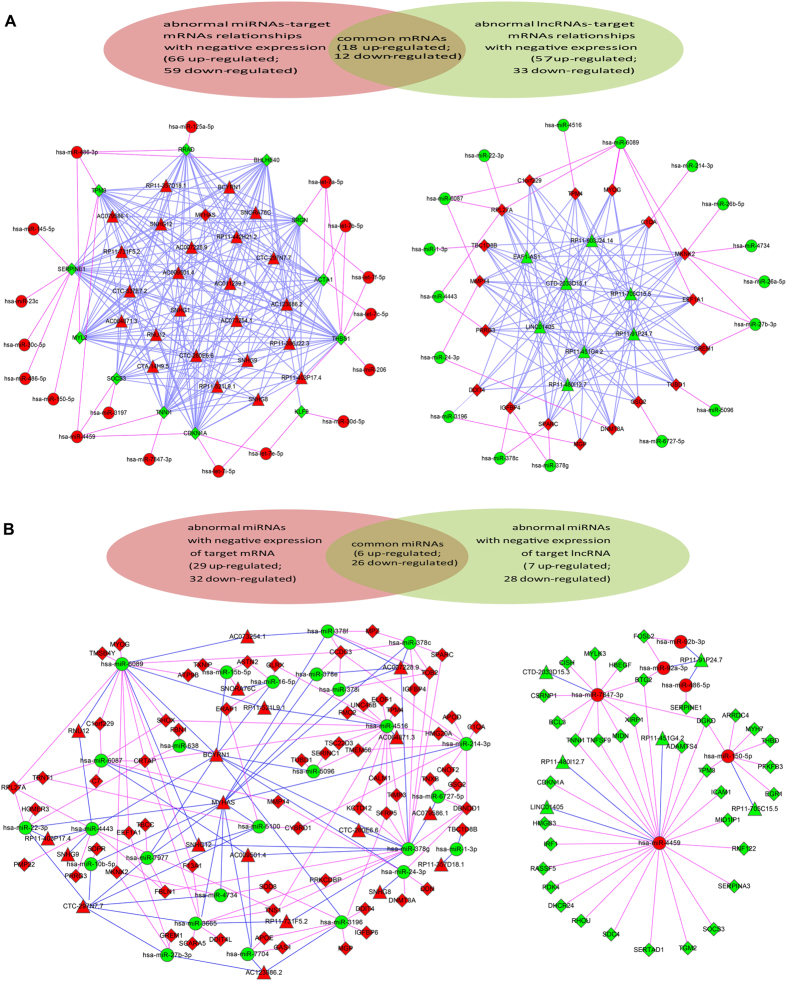
The mtDNA A3243G-regulated miRNA-mRNA-lncRNA (**A**) and lncRNA-miRNA-mRNA (**B**) networks in muscle biopsies of MELAS. Numbers of target mRNAs (**A**) or miRNAs (**B**) in common or specifically in comparison are depicted in venn diagrams, which correspond to the Supplementary Table S4. For the network, circular node represent miRNA, diamond node represent mRNA and triangle node represent lncRNA. Color variations of the nodes and lines represent the following: red node, up-regulated RNA; green node, down-regulated RNA; pink line, miRNA-mRNA network; blue line, miRNA- or mRNA- lncRNA network.

To explore biologic function of significantly dysregulated ncRNAs with fold change >2 that were screened out from the discovery phase, firstly we obtained the potential target pairs between three kinds of RNAs by bioinformatics analyses. Because ncRNAs could induce repression or degradation of their targets, we just focused on target regulation with contrasting expression pattern. Based on that, three mtDNA A3243G-regulated interaction networks of miRNA-mRNA, lncRNA-mRNA and miRNA-lncRNA were constructed (Supplementary Figure S1 and Table S4). There were 61 miRNAs, 125 mRNAs and 259 potential pairs with contrasting expression within the miRNA-mRNA network. In regards to lncRNA-mRNA network, 973 potential lncRNA-mRNA pairs consisted of 34 lncRNAs and 90 mRNAs were constructed. In addition, the miRNA-lncRNA regulating network was formed by 77 miRNA-lncRNA pairs, which consisted of 7 up-regulated miRNAs with 6 down-regulated lncRNAs and 28 down-regulated miRNAs with 19 up-regulated lncRNAs. Finally, we combined all the three regulating networks together and constructed the whole target networks of miRNA-mRNA-lncRNA and lncRNA-miRNA-mRNA (Fig. 3A and B, Supplementary Table S4), both of which were significantly dysregulated in muscle biopsies of MELAS patients vs. controls.

### Gene ontology and KEGG pathway analyses

**Figure 4:**
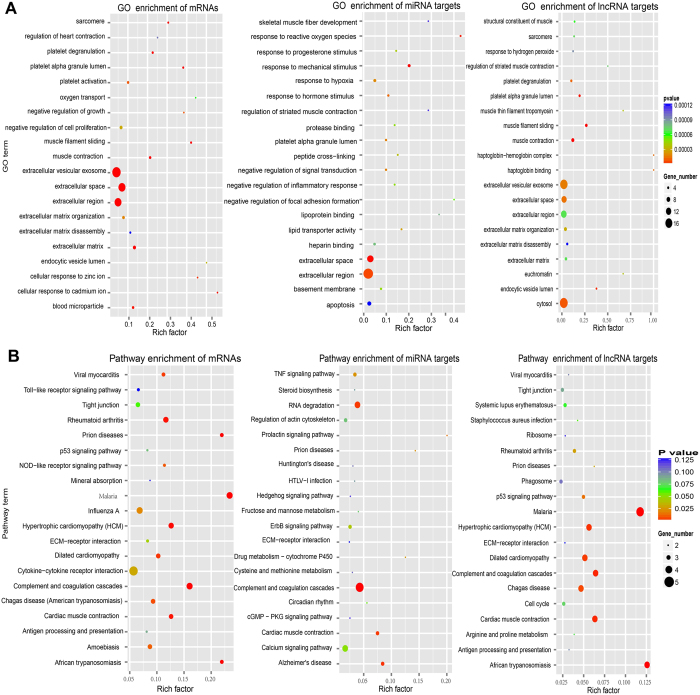
Scatterplots of significant enrichment items of GO analyses (**A**) and pathway analyses (**B**) associated with abnormally expressed mRNAs, miRNA targets, lncRNA targets in muscle biopsies between MELAS patients and controls. The vertical axis is the GO or pathway term, and the horizontal axis is rich factor of GO or pathway enrichment. The P value denotes the significance of the GO or pathway item (the recommend cut-off of P value is 0.05).

Functional annotation was performed by Gene Ontology (GO) and KEGG (Kyoto Encyclopedia of Genes and Genomes) pathway analyses, to determine the biological significance of the whole dysregulated expressed mRNAs and two subsets of mRNAs which were potentially targeted by altered miRNAs/lncRNAs with negative expression between MELAS patients and controls. Classification and main enrichment items of GO and pathway analyses were indicated in Fig. 4 and Supplementary Table S5. In terms of the biological process, the common classification and significantly related categories of mRNAs, miRNAs, lncRNAs pointed to extracellular matrix organization, muscle contraction and protein binding (Fig. 4A, Supplementary Figure S2 and Table S5A). In addition, the significant GO items of the whole differential mRNAs still indicated the small molecule metabolic process, signal transduction and transcription (Fig. 4A, Supplementary Figure S2A and Table S5A). The significant GO classifications of miRNA-mRNA pairs were signal transduction, apoptotic process and development (Fig. 4A, Supplementary Figure S2B and Table S5A). The main associated GO classifications of lncRNA-mRNA pairs were small molecule metabolic process and muscle contraction (Fig. 4A, Supplementary Figure S2C and Table S5A). Correspondingly, all the three KEGG pathway analyses of mRNAs, miRNAs, lncRNAs indicated that the common significant items were immune system, infectious diseases, translation, signal transduction and cell communication. Specifically, KEGG pathway analyses of the whole differential mRNAs were associated with metabolic pathways, cytokine-cytokine receptor interaction, MAPK signaling pathway and so on (Fig. 4B, Supplementary Figure S2A and Table S5B). Subsequently, the pathway annotations of differential miRNAs were mainly involved in complement and coagulation cascades, RNA degradation and calcium signaling pathway (Fig. 4B, Supplementary Figure S2B and Table S5B). Pathway enrichment items of differential lncRNAs included metabolic pathways, muscle contraction, p53 signaling pathway and cytokine-cytokine receptor interaction (Fig. 4B, Supplementary Figure S2C and Table S5B).

### Clinical validation of candidate miRNAs and lncRNAs using an independent MELAS patients and controls

**Figure 5:**
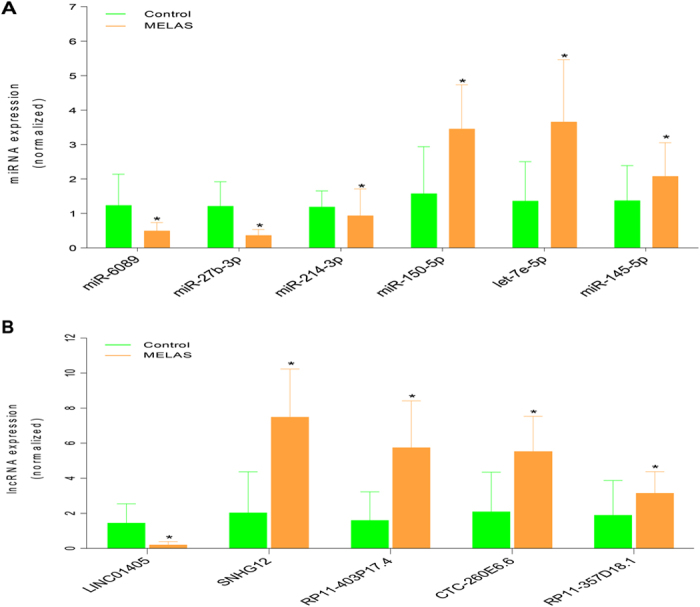
Confirmation of muscle expressions of 6 miRNAs (**A**) and 5 lncRNAs (**B**) using qRT-PCR in validation phase. Expression levels of miRNAs and lncRNAs were normalized to GAPDH and U6 snoRNA, respectively, and calculated utilizing the 2^−ΔΔCT^ method. Data are presented as the mean ± SD. *P < 0.05 versus control group.

**Figure 6:**
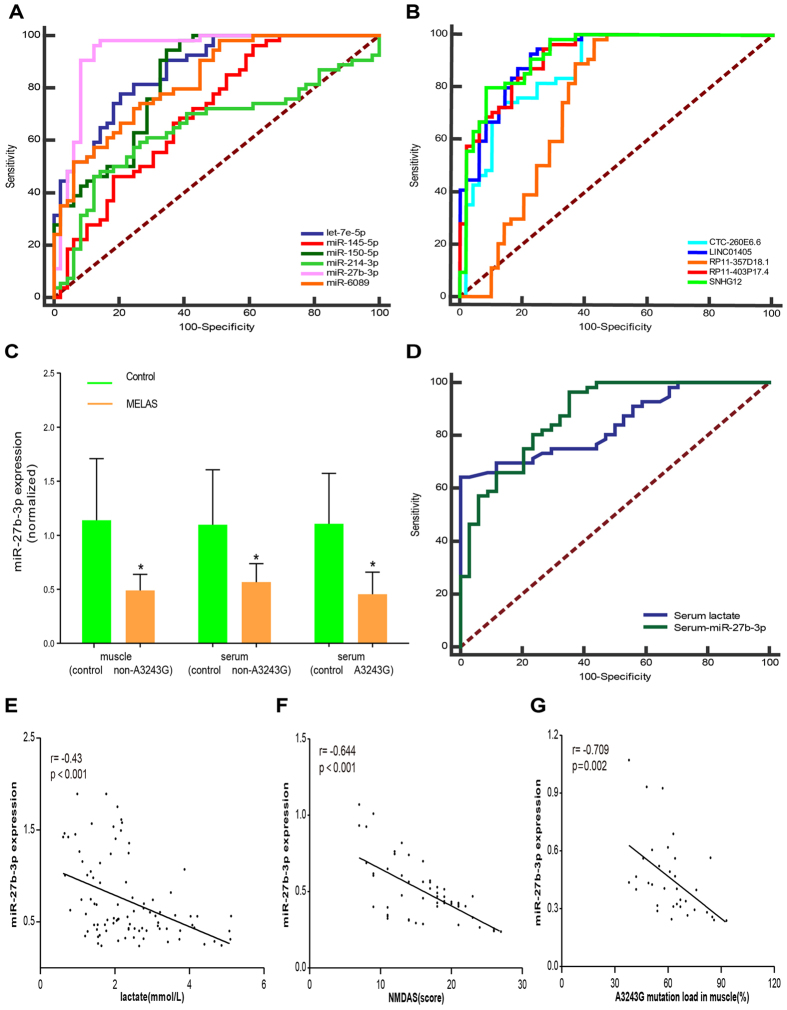
MiR-27b-3p-based biomarker for MELAS diagnosis and association with clinical parameters. ROC curve analyses of 6 miRNAs (**A**) and 5 lncRNAs (**B**) in muscle biopises for discriminating MELAS patients from controls. Serum and muscle miR-27b-3p were reduced in MELAS patients (**C**). It showed higher diagnostic accuracy than lactate for MELAS patients (**D**). Significant correlation was found between serum miR-27b-3p level and lactate (**E**), NMDAS (**F**) or A3243G mutation load in muscle (**G**). Expression level of serum miR-27b-3p was normalized to spiked-in cel-miR-39. Data are presented as the mean ± SD. *P < 0.05 versus control group.

To test whether our findings in training set were replicable for the promising ncRNAs, an independent cohort of 54 MELAS patients and 49 age- and sex-matched controls were chosen for the validation of the selected ncRNAs. As shown in Fig. 5, muscle expressions of miR-6089, miR-27b-3p, miR-214-3p and LINC01405 were significantly down-regulated in MELAS patients vs. controls (P < 0.05). In contrast, muscle expressions of 3 miRNAs (miR-150-5p, let-7e-5p and miR-145-5p) and 4 lncRNAs (SNHG12, RP11-403P17.4, CTC-260E6.6 and RP11-357D18.1) were significantly up-regulated (*P* < 0.05). Furthermore, Receiver Operator Characteristic (ROC) curve analyses were performed to compare the diagnostic value of muscle ncRNAs for MELAS patients (Fig. 6A and B). Interestingly, muscle miR-27b-3p showed the highest diagnostic accuracy with AUC of 0.940 (95%CI: 0.875–0.977) among them (Fig. 6A). The areas under the curve (AUC), adjusted p-value, and asymptotic 95% confidence interval of 11 candidate ncRNAs are shown in Supplementary Table S6. In addition, we continued to test whether miR-27b-3p was also dysregulated in serum between 34 MELAS patients with A3243G mutation and 34 age- and sex- matched controls selected from the validation cohort above. Surprisingly, serum miR-27b-3p was also down-regulated in MELAS patients vs. controls (Fig. 6C). In addition, we further validated that it was also significantly reduced in muscle and serum of 22 MELAS patients with non-A3243G mutation compared with 34 age- and sex- matched controls (Fig. 6C).

### Serum miR-27b-3p-based biomarker for MELAS diagnosis and association with clinical parameters

To test whether reduced serum miR-27b-3p-based biomarker could distinguish MELAS patients from controls, ROC curve analyses were performed to compare the diagnostic value of serum miR-27b-3p with lactate. Serum lactate was up-regulated in MELAS patients vs. controls (Supplementary Figure S3). The AUC, adjusted p-value, and asymptotic 95% confidence interval of serum miR-27b-3p and lactate are shown in Supplementary Table S7. The AUC for lactate distinguishing the MELAS group from controls was 0.841 (95% CI: 0.762–0.919) (Fig. 6D). Serum miR-27b-3p showed higher diagnostic accuracy with AUC of 0.879 (95% CI: 0.806–0.952) than lactate (Fig. 6D). At the cut-off value of 0.93 for miR-27b-3p, the optimal sensitivity and specificity were 96.4% and 64.7%, respectively. Furthermore, correlation analyses were performed to examine the association of expression level of miR-27b-3p with clinical parameters, such as age, sex, BMI, NMDAS, disease duration, lactate and A3243G mutation load in muscle. Significant inverse correlation was found between miR-27b-3p and lactate (R = −0.43, P < 0.001), NMDAS (R = −0.644, P < 0.001) or A3243G mutation load in muscle (R = −0.709, P = 0.002) (Fig. 6E,F and G). Serum miR-27b-3p appeared to decrease with the clinical severity of MELAS patients, and might serve as a useful tool to quantitatively monitor disease severity of MELAS patients.

## Discussion

As the most commonly occurred pathogenic mutation locus of MELAS, mtDNA A3243G mutation in tRNALeu gene leads to pre-termination of transcription, compromising mitochondrial protein synthesis and ATP production, and activation of diverse retrograde signaling pathways^6^. Until recently, the clinical diagnosis and treament of patients with mitochondrial disorders remain a major challenge to clinicians due to the complex pathogenesis and the clinical variability^1^. In this study, we hypothesized that ncRNAs driven by mtDNA A3243G mutation could regulate the signaling pathways involved in mitochondrial myopathy and have firstly employed an integrated genomic approach to identify the dysregulated miRNAs and lncRNAs in muscle biopsies of a well characterized cohort of MELAS with mtDNA A3243G mutation (including 20 MELAS patients and 20 healthy controls in discovery and training phase, 54 MELAS patients and 49 controls in validation phase), which allowed us to identify ncRNAs of interest that may underlie MELAS pathogenesis for further studies.

The decisive factors involved in the secondary pathogenic cascades and compensatory adaptations in mitochondrial myopathy are still obscure. In current study, by coexpression profiling of mRNAs, miRNAs and lncRNAs, we identified the differential expressed RNAs in muscle biopsies of MELAS with mtDNA A3243G cytotoxicity. The patients were only restricted to MELAS with mtDNA A3243G mutation to reduce the potential for confounding effects due to sample heterogeneity, which would be expected with the inclusion of other kinds of mitochondrial mutation. Indeed the mitochondrial dysfunction associated with mtDNA A3243G cytotoxicity could activate a far greater retrograde signaling pathways than previously anticipated. We found that 329 mRNAs were significantly dysregulated in MELAS patients vs. controls. Interestingly, some of them were previously reported to be dysregulated in mitochondrial myopathy, including MYC, ATP2A2, ACTN3, AQP4, GLUL, TRIM63 and PFKFB3, which were partly consistent with transcriptome datasets from previous studies of mitochondrial genetic disorders^21^,^22^,^23^,^24^. This suggested that mitochondrial disease have disparate causes, and yet share common mechanistic themes^25^. In accordance with previous reports, further GO & Pathway analyses indicated these deregulated mRNAs were mainly involved in small molecule metabolic process, extracellular matrix organization, muscle contraction, protein binding, signal transduction, transcription and so on, indicating the importance of these signaling pathways in mitochondrial myopathy. As for the dysregulated ncRNAs, 72 miRNAs and 102 lncRNAs were found to be dysregulated in MELAS patients vs. controls. Interestingly, some miRNAs were also reported in gene expression profiling (GEP) datasets obtained from previously published oxidative stress cell models, such as miR-1-3p^26^, miR-15b-5p^27^, miR-29a-3p^28^, miR-128-3p^29^, miR-133a-3p^30^, miR-145-5p^31^, miR-150-5p^32^, miR-499a-5p^33^, which indicated that they might participate in the occurrence and development of MELAS through oxidative stress pathway. For example, some of these meaningful ncRNAs haven been investigated to be associated with mitochondrial function. Both miR-150-5p and let-7 family were thought to be mitochondria-enriched miRNAs^32^. The induction of miR-145-5p might promote cell apoptosis as its downstream target SOD2 was known to play the antioxidant defense^34^. However, miR-145-5p could suppresses ROS-induced Ca^2+^ overload of cardiomyocytes by targeting CaMKIIδ^31^. In addition, PDE4B was found to be the potential target of miR-145-5p in our target analysis, and has been implicated in the suppression of apoptosis^35^. Over-expression of miR-145-5p significantly inhibited the H_2_O_2_-induced cellular apoptosis, ROS production, mitochondrial structure disruption as well as the activation of key signaling proteins in mitochondrial apoptotic pathway^36^. Previous studies have declared that miR-214-3p could regulate mitochondrial morphology and cell cycle by targeting Mitofusin2^37^, or Ca^2+^ homeostasis by repression of Ncx1^38^. At present, more than 50,000 lncRNA genes have been identified. However, only a very limited number of lncRNAs have biological function annotations. Although some mitochondrial-related lncRNAs, including RMRP^16^, MDRL^17^, TGFB2-OT1^18^, CARL^19^ and UCA1^20^, were already reported to regulate mitochondrial function, detailed participation mechanisms in mitochondrial myopathy of the dysregulated lncRNAs identified in this study have yet to be elucidated.

Integrated analyses of target prediction and functional enrichment based on coexpression networks may provide a clue for elucidating the precise underlying mechanisms of ncRNAs in pathogenesis caused by mtDNA A3243G mutation. We made interaction analyses and constructed three regulatory networks which consisted of miRNA-mRNA, lncRNA-mRNA or miRNA-lncRNA with contrast coexpresstion. In addition, the hypothesis of competing endogenous RNA (ceRNA) suggested large-scale regulatory networks between coding and noncoding RNAs across the transcriptome^39^. LncRNAs and mRNAs might interact through competition with the same miRNA, or miRNAs and lncRNAs could interact with each other, thus regulating function of the same mRNAs. So the mtDNA A3243G-regulated miRNA-mRNA-lncRNA and lncRNA-miRNA-mRNA networks in MELAS muscle were constructed. Because most of the ncRNAs in the coexpression network were not annotated yet, GO & Pathway analyses were performed to further annotate the biological function of these dysregulated ncRNAs. In accordance with analysis of the whole deregulated mRNAs, function analyses of potential targets of miRNA and lncRNA all showed that the mtDNA A3243G-regulated ncRNAs signaling pathways were enriched in immune response, signal transduction, apoptotic process and development, suggesting that they might contribute to MELAS pathogenesis through these signaling pathways. Finally, our study provided valuable clues for the identification of potential ceRNA pairs between miRNAs and mRNAs, lncRNAs and mRNAs or even miRNAs and lncRNAs caused by mtDNA A3243G mutation. It is very much worthy to perform further validation and functional examination *in vivo* and *in vitro* experiments to reveal the underlying mechanisms of these ncRNAs in MELAS.

Reduced serum miR-27b-3p showed potential as a biomarker for MELAS diagnosis. Although allowed for high-throughput analyses, the approach of the high-throughput assay with mixed samples in the first step of preliminary screening ignored the individual differences. So qRT-PCR validation was then followed to verify the meaningful ncRNAs selected from the regulatory network focus of MELAS. Consistent with results from the high-throughput data, we confirmed that muscle expressions of 4 ncRNAs (miR-6089, miR-27b-3p, miR-214-3p and LINC01405) were significantly down-regulated, and 7 ncRNAs (miR-150-5p, let-7e-5p, miR-145-5p, SNHG12, RP11-403P17.4, CTC-260E6.6 and RP11-357D18.1l) significantly up-regulated in MELAS muscle biopsies vs. controls, indicating that they might be acting as suppressors or accelerator in occurrence or progression of MELAS pathogenesis. Some of these meaningful ncRNAs haven been investigated to be associated with mitochondrial function. The oxidative stress-responsive miR-27b-3p has been shown to alleviate oxidative stress and inflammation^40^, regulate NO production^41^, improve mitochondrial function^42^ and suppresses apoptosis^43^,^44^. On the other hand, miR-27b-3p might prevent the autophagic degradation of impaired mitochondria^45^. The dual effect of miR-27b-3p is possibly tissue- or cellular context-dependent, which is consistent with other miRNAs^46^. Apart from many targets including PINK1, FOXO1, PPARγ and Apaf-1 investigated before, MKNK2, GREM1 and EEF1A1 that might be potential targets of miR-27b-3p were revealed in the regulatory network of MELAS pathogenesis. Anyhow the role of miR-27b-3p in MELAS pathogenesis is still needed to elucidate. In addition, the following ROC curve analyses showed the highest sensitivity and specificity of muscle miR-27b-3p in differentiating MELAS patients from controls among the selected ncRNAs. So we evaluated its serum expression and confirmed its reduction not only in muscle and serum of MELAS with A3243G but also non-A3243G mutation. ROC curve analyses further indicated that serum miR-27b-3p had the better diagnostic value for MELAS that lactate. Correlation analyses also showed significant inverse correlation between miR-27b-3p and lactate or NMDAS. Muscle tissue might be an important source of serum miR-27b-3p because of its relevance with A3243G mutation load in muscle. Although elevated lactate level in serum and CSF may be helpful in diagnosis of mitochondrial diseases, it still lacks consistency as some patients with confirmed diagnosis don’t show increased lactate level. The results in this study indicated that reduced serum miR-27b-3p could be a new potential biomarker applicable for MELAS diagnosis. However, it is still needed to expand the sample size in future studies.

Taken together, to our knowledge, this is the first study to construct coexpression networks among a series of miRNAs, mRNAs, and lncRNAs downstream of mtDNA A3243G mutation in mitochondrial myopathy. And we identified reduced serum miR-27b-3p for MELAS diagnosis with high sensitivity and specificity. However, sufficient sensitivity and specificity of the miR-27b-3p should be determined in the further well designed clinical studies. These findings may have substantial clinical significance or implications for illustrating the pathogenesis of mitochondrial myopathy, and follow-up investigation is warranted to better understand the detailed participation mechanisms of miRNA and lncRNA in mitochondrial myopathy pathogenesis.

## Materials and Methods

### Characteristics of individuals and muscle biopsy

The diagnosis of MELAS was based on the criteria of Newcastle Pediatric Mitochondrial Disease scale NPMDS and Sanger sequencing^47^. All muscle biopsy specimens were retrospectively collected from patients with mitochondrial myopathy and from subjects who were ultimately deemed to be free of neuromuscular diseases matched for age and gender selected as non-diseased controls. All procedures were approved by the Ethics committee of Qilu Hospital affiliated to Shandong University (Jinan, China), and carried out in accordance with the principles of Helsinki Declaration. All collecting samples of muscle biopsies were informed consent of patients at the Neuromuscular Center at Qilu Hospital.

### MicroRNA microarray Assay

Total RNA (including miRNAs) from each frozen muscle was extracted using Animal Tissue Kit(LCS, TRK-1002) according to the manufacturer’s instructions. The total RNA quantity and purity was analysed with Bioanalyzer 2100 and RNA 6000 Nano Lab Chip Kit (Agilent, CA, USA) with RIN number >8.0. Qualified total RNA was further purified by RNeasy mini kit (QIAGEN, GmbH, Germany) and RNase-Free DNase Set (QIAGEN, GmbH, Germany). Total RNA from the same stage were mixed in equal amounts into two pooled samples: MELAS and Control. Microarray assay (miRHumanViruses-21b array) was performed by the service provider (LC Sciences). The assay started from 4 to 8 μg total RNA sample which were 3′-extended with a poly(A) tail using poly(A) polymerase. An oligonucleotide tag was then ligated to the poly(A) tail for later fluorescent dye staining. Hybridization was performed overnight on a μParaflo microfluidic chip using a micro-circulation pump (Atactic Technologies)^48^. On the microfluidic chip, each detection probe consisted of a chemically modified nucleotide coding segment complementary to target microRNA (from miRBase, http://www.mirbase.org/) or other RNA (control or customer defined sequences) and a spacer segment of polyethylene glycol to extend the coding segment away from the substrate. The detection probes were made by *in situ* synthesis using PGR (photogenerated reagent) chemistry. The hybridization melting temperatures were balanced by chemical modifications of the detection probes. Hybridization used 100 L 6xSSPE buffer (0.90 M NaCl, 60 mM Na2HPO4, 6 mM EDTA, pH 6.8) containing 25% formamide at 34 °C After RNA hybridization, tag-conjugating Cy3 dye were circulated through the microfluidic chip for dye staining. Fluorescence images were collected using a laser scanner (GenePix 4000B, Molecular Device) and digitized using Array-Pro image analysis software (Media Cybernetics). Data was analyzed by first subtracting the background and then normalizing the signals using a LOWESS filter (Locally-weighted Regression)^49^. To identify statistically significance for differentially expressed miRNAs, a criterion of signal >500, |fold change| ≥1.0 and FDR < 0.01 was used. Microarray data was deposited in GEO database (GSE89059).

### LncRNA library construction and Illumina sequencing

Total RNA was collected as described above. First, approximately 5 ug of total RNA was used to deplete ribosomal RNA according to the manufacturer’s instructions of the Ribo-Zero™ Magnetic Kit (Epicentre). Following purification, the RNA is fragmented into small pieces using divalent cations under elevated temperature. Then the cleaved RNA fragments were reverse-transcribed to create the final cDNA library in accordance with the protocol for the RNA-Seq sample preparation kit (Illumina, San Diego, USA). And then we performed the single-end sequencing (50 nt) on an Illumina Hiseq2500 sequencer at the LC Biotech (Hangzhou, China) following the vendor’s recommended protocol. The sequence results were obtained as the FPKM (fragment per kilobase of exons per million reads) for each transcript and were deposited in GEO database (GSE89065).

### Confirmation and quantification of miRNAs, lncRNAs and mRNAs by qRT-PCR

To validate the microarray and sequencing datasets, qRT-PCR were performed for most promising differential miRNAs, lncRNAs and mRNAs. For muscle miRNA and lncRNA/mRNA confirmation, U6 and GAPDH were used as the endogenous reference gene, respectively. Total muscle RNA for qRT-PCR was collected as described above. For serum miR-27b-3p, synthetic cel-miR-39 was added to each denatured sample (25 fmol in a 5 μl total volume) as stable control for normalization in serum. The serum miRNA was isolated from 400 μL serum sample for each individual using the Ambion mirVana™ PARIS™ Kit (Ambion). For muscle lncRNA/mRNA, cDNA was synthesized from total RNA using the M-MLV RTase cDNA Synthesis kit (Takara, Dalian, China) according to the manufacturer’s instructions. For muscle miRNA, cDNA was reverse transcribed from total RNA in a final volume of 20 μl using One Step PrimeScript miRNA cDNA Synthesis Kit (Takara, Dalian, China). Quantitative real-time PCR was carried out on an ABI Prism 7900HT (Applied Biosystems) with SYBR Premix Ex Taq^TM^ II (Takara, Japan) according to the manufacturer’s protocol. The primer sequences are listed in the Supplementary Table S7. All reactions were tested in triplicate, and differences of threshold cycles between target genes and house-keeping genes (U6 in muscle miRNA, GAPDH in muscle lncRNA/mRNA, and cel-miR-39 in serum miR-27b-3p) were calculated using the 2^−ΔΔCT^ method and analyzed by paired t tests to evaluate statistical significance relative to controls. Correct qPCR product size was verified by agarose gel electrophoresis, and melting curve measurements were made after every experiment.

### The construction of miRNA, lncRNA and mRNA regulatory networks

Integration of ncRNA and mRNA expression profiling may allow the identification of functional links between dysregulated ncRNAs and their targets. MiRNA target genes were predicted by TargetScan, miRecords, TarBase, and miRecords software. The putative miRNA binding sites of the lncRNAs sequences were performed using the RegRNA software (http://regrna2.mbc.nctu.edu.tw/). LncRNAs were predicted via two independent algorithms: cis- or trans-regulatory effects^50^. We selected the dysregulated target RNA with an inverse correlation of expression with the respective differential ncRNA. The miRNA-mRNA-lncRNA and lncRNA-miRNA-mRNA interaction networks were built with the Cytoscape Software (version 3.1.0).

### Functional annotation of differentially expressed miRNAs and lncRNAs

Ingenuity pathways analysis (IPA) software was used to identify signaling pathways regulated by mtDNA A3243G mutation mining from our miRNAs/lncRNAs datasets. Functional annotation was performed by Gene Ontology (GO) and Pathway analysis to determine the biological significance of the differentially expressed mRNAs targeted by miRNA/lncRNA. Dysregulated mRNAs were input into the Database for Annotation, Visualization and Integrated Discovery (DAVID; http://david.abcc.ncifcrf.gov/) v6.7, which utilized GO to identify the molecular function represented in the gene profile and the Kyoto Encyclopedia of Genes and Genomes (KEGG; http://www.genome.jp/kegg/) to analyze the potential functions of these genes in pathways. The GO analysis was divided into molecular function, biological process and cellular component. Finally, results of the enrichment analysis were ranked by the significantly enriched GO term and KEGG pathway according to hypergeometric distribution. A corrected p-value < 0.05 was the threshold for statistically significant correlation. As the enrichment increases, the corresponding function is more specific, which helps us to identify GOs and pathways with more concrete functional description in MELAS.

### Statistical analysis

Clinical characteristics were compared using χ^2^ test of independence for qualitative variables, t-test of quantitative variables with normal distribution, the non-parametric Kruskall-Wallis test or the Mann-Whitney U test of quantitative variables with skewed distribution. The threshold value we used to screen differentially expressed lncRNAs/miRNAs/mRNAs was a fold change >1.5(P < 0.05). Expression levels of lncRNAs/miRNAs were compared using the Kruskall-Wallis test or the Mann-Whitney U test. ROC curves and AUC were established to evaluate the diagnostic value of lncRNAs and miRNAs for differentiating melas patients with healthy controls. In ROC analysis, the normalized expression level of RNAs (2^−ΔΔCT^) was selected as the test variable for the selected lncRNAs and miRNAs. All data were expressed as mean ± standard deviation (mean ± SD). A p value of less than 0.05 was considered statistically significant. All analyses were performed by SPSS 17.0 software (SPSS, Chicago, IL, USA), R (http://www.r-project.org/, version 2.15.3) and Bioconductor 30, or Graphpad Prism (version 5.0; Graphpad software).

## Additional Information

**How to cite this article**: Wang, W. *et al*. Identification of miRNA, lncRNA and mRNA-associated ceRNA networks and potential biomarker for MELAS with mitochondrial DNA A3243G mutation. *Sci. Rep.* **7**, 41639; doi: 10.1038/srep41639 (2017).

**Publisher's note:** Springer Nature remains neutral with regard to jurisdictional claims in published maps and institutional affiliations.

## Supplementary Material

Supplementary Information
