# Supplementary material for: Identification of miRNA, lncRNA and mRNA-associated ceRNA networks and potential biomarker for MELAS with mitochondrial DNA A3243G mutation
Source: Sci Rep. 2017 Jan 31;7:41639. doi: 10.1038/srep41639 (PMC5282567; doi:10.1038/srep41639)
Supplement: Supplementary Information [file srep41639-s1.pdf]

Identification of miRNA, lncRNA and mRNA-associated ceRNA networks and potential biomarker for MELAS with mitochondrial DNA A3243G mutation

Wei Wang[1], Qianqian Zhuang[2], Kunqian Ji[1], Bing Wen[1], Pengfei Lin[1], Yuying Zhao[1], Wei Li[1] & Chuanzhu Yan[1,3]

[1] Laboratory of Neuromuscular Disorders and Department of Neurology, Qilu Hospital, Shandong University, Jinan, China.
[2] School of Food Science and Engineering, Qilu University of Technology, Jinan, China.
[3] Key Laboratory for Experimental Teratology of the Ministry of Education, Brain Science Research Institute, Department of Neurology, Qilu Hospital, Shandong University, Jinan, China.

Supplementary Figure and Table legends

Supplementary Figure S1. The mtDNA A3243G-regulated interaction networks of miRNA-mRNA(A), lncRNA-mRNA (B) and miRNA-lncRNA (C) dysregulated in muscle biopsies of MELASpatients.
Supplementary Figure S2. GO and pathway classification of abnormally expressed mRNAs(A)、 miRNA targets(B)、lncRNA targets(C) in muscle biopsies between MELAS patients and controls.
Supplementary Figure S3. Differential expression levels of serum lactate between 56 MELAS patients and 34 Controls.

Description of the supplementary tables:
Supplementary Table S1. The characteristics of the study population.
Supplementary Table S2. Differential expression of miRNAs, lncRNAs and mRNAs between MELAS patients vs. controls.
Supplementary Table S3. The miRNAs, lncRNAs and mRNAs screened from high-throughout results for qRT-PCR validation.
Supplementary Table S4. Target pridicitions of miRNA-mRNA, lncRNA-mRNA and miRNA-lncRNA with negative expression.
Supplementary Table S5. GO and KEGG analyses of the dysregulated mRNAs, miRNA targets and lncRNA targets.
Supplementary Table S6. ROC curve analyses of the selected ncRNAs.
Supplementary Table S7. The primer sequences of selected lncRNAs and mRNAs for qPCR.

Figure S1. The mtDNA A3243G-regulated interaction networks of miRNA-mRNA (**A**), lncRNA-mRNA (**B**) and miRNA-lncRNA (**C**) in muscle biopsies of MELAS. For the network, circular nodes represent miRNAs, diamond nodes represent mRNAs and triangle nodes represent lncRNAs. Color variations of the nodes represent the following: red, upregulated RNAs; green, downregulated RNAs.

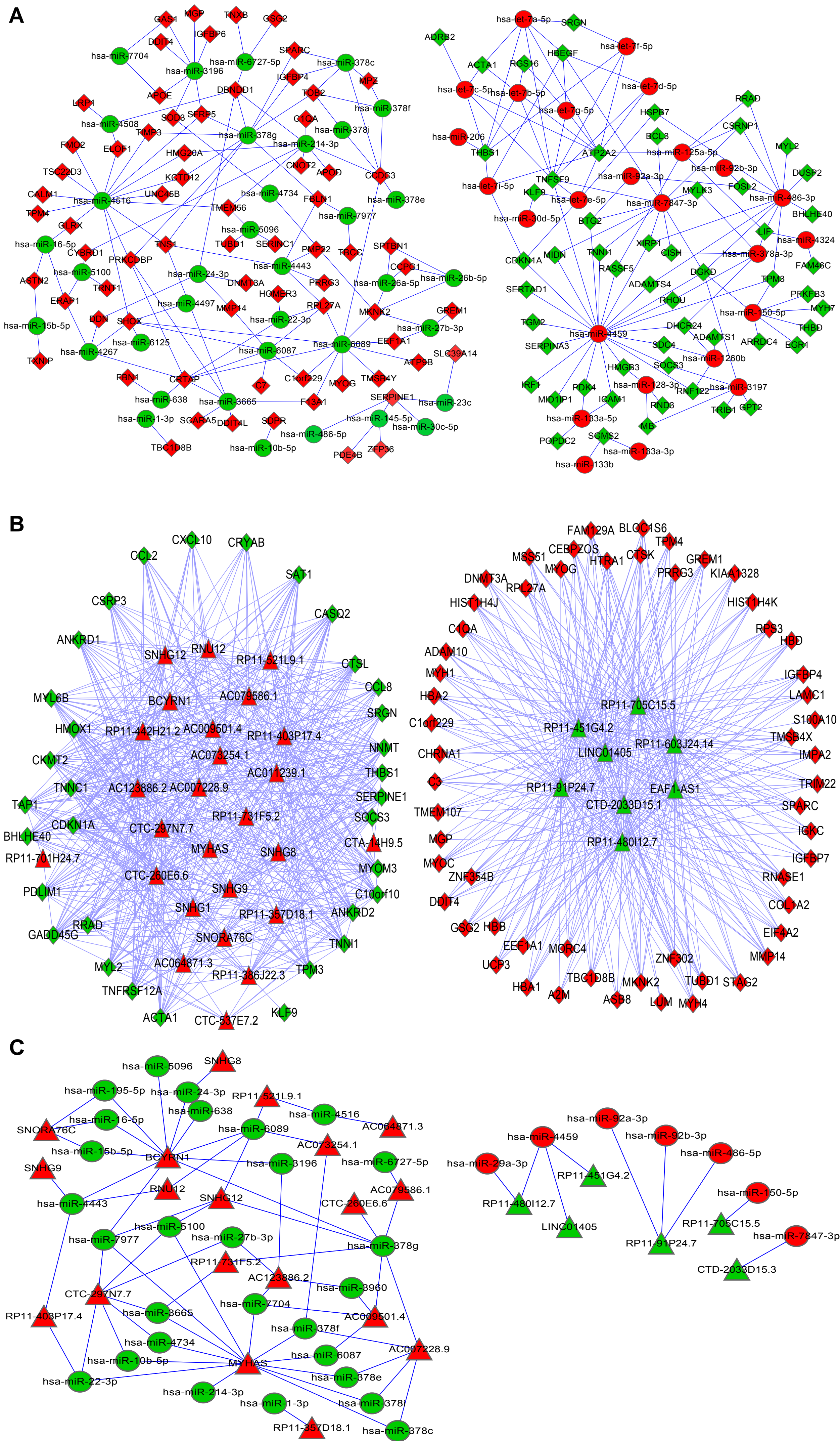

A
GAS1
MGP
TNXB
GSG2
IGFBP6
DDIT4
hsa-miR-7704
hsa-miR-3196
hsa-miR-6727-5p
SPARC
hsa-miR-378c
APOE
IGFBP4
MPZ
DBNDD1
TOB2
LRP1
hsa-miR-378f
hsa-miR-4508
SOD3
SFRP5
C1QA
hsa-miR-378i
TIMP3
hsa-miR-378g
FMO2
ELOF1
hsa-miR-214-3p
HMG20A
CNOT2
APOD
TSC22D3
CCDC3
KCTD12
CALM1
hsa-miR-4516
UNC45B
hsa-miR-4734
FBLN1
hsa-miR-378e
TMEM56
TPM4
hsa-miR-7977
GLRX
hsa-miR-5096
hsa-miR-16-5p
TNS1
TUBD1
SERINC1
SRTBN1
CYBRD1
PRKCDBP
PMP22
TBCC
hsa-miR-5100
hsa-miR-4443
CCPG1
ASTN2
TRNT1
hsa-miR-24-3p
hsa-miR-26b-5p
DNMT3A
PRRG3
hsa-miR-26a-5p
ERAP1
hsa-miR-4497
HOMER3
RPL27A
MMP14
MKNK2
GREM1
DDN
SHOX
hsa-miR-22-3p
hsa-miR-15b-5p
hsa-miR-6125
hsa-miR-27b-3p
hsa-miR-4267
hsa-miR-6087
hsa-miR-6089
EEF1A1
SLC39A14
TXNIP
ATP9B
FBN1
CRTAP
C7
C1orf229
MYOG
TMSB4Y
hsa-miR-638
hsa-miR-3665
F13A1
SERPINE1
hsa-miR-23c
hsa-miR-1-3p
SCARA5
DDIT4L
SDPR
hsa-miR-145-5p
hsa-miR-30c-5p
hsa-miR-486-5p
TBC1D8B
hsa-miR-10b-5p
PDE4B
ZFP36
hsa-let-7a-5p
SRGN
ADRB2
hsa-let-7f-5p
ACTA1
HBEGF
RGS16
hsa-let-7c-5p
hsa-let-7b-5p
hsa-let-7d-5p
hsa-let-7g-5p
HSPB7
RRAD
hsa-miR-206
BCL3
CSRNP1
THBS1
ATP2A2
hsa-miR-125a-5p
MYL2
hsa-miR-92b-3p
TNFSF9
hsa-miR-92a-3p
DUSP2
hsa-let-7i-5p
FOSL2
KLF9
MYLK3
hsa-let-7e-5p
hsa-miR-486-3p
hsa-miR-7847-3p
hsa-miR-30d-5p
BTG2
BHLHE40
LIF
hsa-miR-4324
XIRP1
CDKN1A
MIDN
TNNI1
CISH
hsa-miR-378a-3p
RASSF5
FAM46C
DGKD
ADAMTS4
TPM3
SERTAD1
PRKFB3
RHOU
hsa-miR-150-5p
MYH7
TGM2
DHCR24
THBD
hsa-miR-4459
ADAMTS1
ARRDC4
EGR1
SERPINA3
SDC4
HMGB3
SOCS3
hsa-miR-1260b
IRF1
PDK4
hsa-miR-128-3p
RNF122
hsa-miR-3197
MID1IP1
ICAM1
RND3
TRIB1
GPT2
hsa-miR-133a-5p
MB
POPDC2
SGMS2
hsa-miR-133a-3p
hsa-miR-133b
B
CXCL10
CRYAB
CCL2
SAT1
CSRP3
CASQ2
ANKRD1
SNHG12
RNU12
RP11-521L9.1
CTSL
CCL8
MYL6B
BCYRN1
AC079586.1
SRGN
HMOX1
RP11-442H21.2
AC009501.4
RP11-403P17.4
NNMT
CKMT2
AC073254.1
AC011239.1
THBS1
TNNC1
AC123886.2
AC007228.9
SERPINE1
TAP1
CDKN1A
RP11-731F5.2
SOCS3
BHLHE40
CTC-297N7.7
CTA-14H9.5
RP11-701H24.7
MYHAS
SNHG8
MYOM3
CTC-260E6.6
PDLIM1
SNHG9
C10orf10
SNHG1
RP11-357D18.1
ANKRD2
RRAD
GADD45G
SNORA76C
TNNI1
AC064871.3
MYL2
RP11-386J22.3
TPM3
TNFRSF12A
ACTA1
CTC-537E7.2
KLF9
FAM129A
BLOC1S6
MSS51
CEBPZOS
TPM4
HTRA1
CTSK
GREM1
MYOG
PRRG3
KIAA1328
DNMT3A
RPL27A
HIST1H4J
HIST1H4K
C1QA
RPS3
HBD
ADAM10
IGFBP4
MYH1
HBA2
RP11-705C15.5
LAMC1
RP11-451G4.2
S100A10
C1orf229
RP11-603J24.14
TMSB4X
LINC01405
CHRNA1
IMPA2
C3
RP11-91P24.7
EAF1-AS1
TRIM22
CTD-2033D15.1
TMEM107
SPARC
RP11-480I12.7
MGP
IGKC
MYOC
IGFBP7
ZNF354B
RNASE1
DDIT4
COL1A2
GSG2
HBB
EIF4A2
EEF1A1
MORC4
ZNF302
MMP14
UCP3
TBC1D8B
MKNK2
TUBD1
STAG2
HBA1
A2M
ASB8
LUM
MYH4
C
hsa-miR-5096
SNHG8
hsa-miR-195-5p
hsa-miR-24-3p
RP11-521L9.1
hsa-miR-638
hsa-miR-16-5p
hsa-miR-4516
SNORA76C
hsa-miR-6089
AC064871.3
hsa-miR-15b-5p
AC073254.1
BCYRN1
hsa-miR-3196
hsa-miR-6727-5p
SNHG9
RNU12
AC079586.1
hsa-miR-4443
SNHG12
CTC-260E6.6
hsa-miR-5100
hsa-miR-7977
hsa-miR-27b-3p
hsa-miR-378g
RP11-731F5.2
AC123886.2
hsa-miR-3960
CTC-297N7.7
hsa-miR-7704
RP11-403P17.4
hsa-miR-3665
AC009501.4
hsa-miR-378f
hsa-miR-4734
AC007228.9
hsa-miR-6087
hsa-miR-10b-5p
MYHAS
hsa-miR-378e
hsa-miR-22-3p
hsa-miR-214-3p
hsa-miR-1-3p
hsa-miR-378i
RP11-357D18.1
hsa-miR-378c
hsa-miR-92a-3p
hsa-miR-4459
hsa-miR-92b-3p
hsa-miR-29a-3p
hsa-miR-486-5p
RP11-451G4.2
RP11-480I12.7
hsa-miR-150-5p
LINC01405
RP11-705C15.5
RP11-91P24.7
hsa-miR-7847-3p
CTD-2033D15.3

Figure S2. GO and pathway classification of abnormally expressed mRNAs (**A**)、miRNA targets (**B**)、lncRNA targets (**C**) in muscle biopsies between MELAS patients and controls. The vertical axis is the GO or pathway item, and the horizontal axis is percent of genes specific to GO or pathway item. Each item is indexed by the number of genes correlated to the specific GO or pathway item.

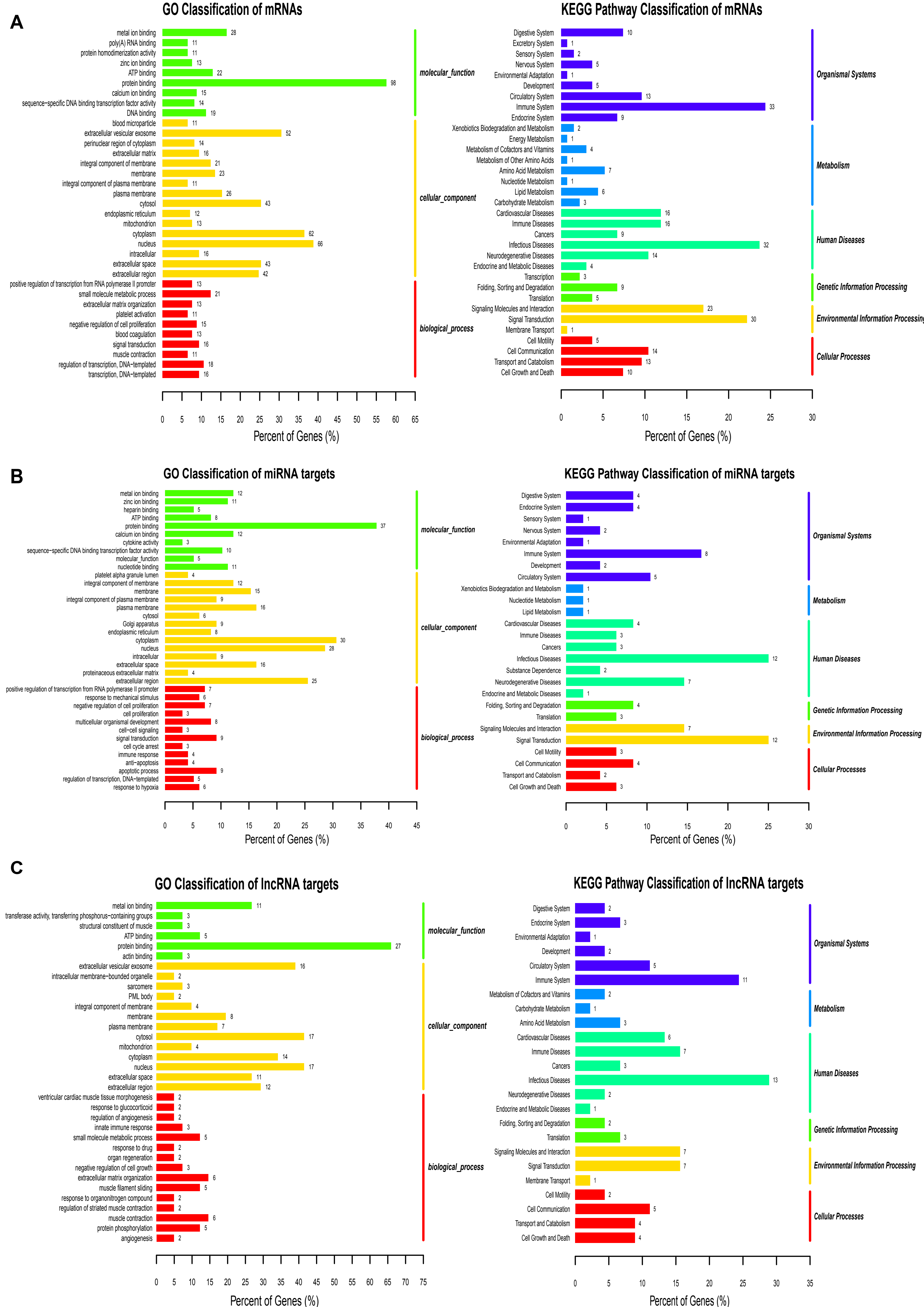
A
GO Classification of mRNAs
metal ion binding 28
poly(A) RNA binding 11
protein homodimerization activity 11
zinc ion binding 13
ATP binding 22
protein binding 98
calcium ion binding 15
sequence−specific DNA binding transcription factor activity 14
DNA binding 19
blood microparticle 11
extracellular vesicular exosome 52
perinuclear region of cytoplasm 14
extracellular matrix 16
integral component of membrane 21
membrane 23
integral component of plasma membrane 11
plasma membrane 26
cytosol 43
endoplasmic reticulum 12
mitochondrion 13
cytoplasm 62
nucleus 66
intracellular 16
extracellular space 43
extracellular region 42
positive regulation of transcription from RNA polymerase II promoter 13
small molecule metabolic process 21
extracellular matrix organization 13
platelet activation 11
negative regulation of cell proliferation 15
blood coagulation 13
signal transduction 16
muscle contraction 11
regulation of transcription, DNA−templated 18
transcription, DNA−templated 16
molecular_function
cellular_component
biological_process
0 5 10 15 20 25 30 35 40 45 50 55 60 65
Percent of Genes (%)
KEGG Pathway Classification of mRNAs
Digestive System 10
Excretory System 1
Sensory System 2
Nervous System 5
Environmental Adaptation 1
Development 5
Circulatory System 13
Immune System 33
Endocrine System 9
Xenobiotics Biodegradation and Metabolism 2
Energy Metabolism 1
Metabolism of Cofactors and Vitamins 4
Metabolism of Other Amino Acids 1
Amino Acid Metabolism 7
Nucleotide Metabolism 1
Lipid Metabolism 6
Carbohydrate Metabolism 3
Cardiovascular Diseases 16
Immune Diseases 16
Cancers 9
Infectious Diseases 32
Neurodegenerative Diseases 14
Endocrine and Metabolic Diseases 4
Transcription 3
Folding, Sorting and Degradation 9
Translation 5
Signaling Molecules and Interaction 23
Signal Transduction 30
Membrane Transport 1
Cell Motility 5
Cell Communication 14
Transport and Catabolism 13
Cell Growth and Death 10
Organismal Systems
Metabolism
Human Diseases
Genetic Information Processing
Environmental Information Processing
Cellular Processes
0 5 10 15 20 25 30
Percent of Genes (%)
B
GO Classification of miRNA targets
metal ion binding 12
zinc ion binding 11
heparin binding 5
ATP binding 8
protein binding 37
calcium ion binding 12
cytokine activity 3
sequence−specific DNA binding transcription factor activity 10
molecular_function 5
nucleotide binding 11
platelet alpha granule lumen 4
integral component of membrane 12
membrane 15
integral component of plasma membrane 9
plasma membrane 16
cytosol 6
Golgi apparatus 9
endoplasmic reticulum 8
cytoplasm 30
nucleus 28
intracellular 9
extracellular space 16
proteinaceous extracellular matrix 4
extracellular region 25
positive regulation of transcription from RNA polymerase II promoter 7
response to mechanical stimulus 6
negative regulation of cell proliferation 7
cell proliferation 3
multicellular organismal development 8
cell−cell signaling 3
signal transduction 9
cell cycle arrest 3
immune response 4
anti−apoptosis 4
apoptotic process 9
regulation of transcription, DNA−templated 5
response to hypoxia 6
molecular_function
cellular_component
biological_process
0 5 10 15 20 25 30 35 40 45
Percent of Genes (%)
KEGG Pathway Classification of miRNA targets
Digestive System 4
Endocrine System 4
Sensory System 1
Nervous System 2
Environmental Adaptation 1
Immune System 8
Development 2
Circulatory System 5
Xenobiotics Biodegradation and Metabolism 1
Nucleotide Metabolism 1
Lipid Metabolism 1
Cardiovascular Diseases 4
Immune Diseases 3
Cancers 3
Infectious Diseases 12
Substance Dependence 2
Neurodegenerative Diseases 7
Endocrine and Metabolic Diseases 1
Folding, Sorting and Degradation 4
Translation 3
Signaling Molecules and Interaction 7
Signal Transduction 12
Cell Motility 3
Cell Communication 4
Transport and Catabolism 2
Cell Growth and Death 3
Organismal Systems
Metabolism
Human Diseases
Genetic Information Processing
Environmental Information Processing
Cellular Processes
0 5 10 15 20 25 30
Percent of Genes (%)
C
GO Classification of lncRNA targets
metal ion binding 11
transferase activity, transferring phosphorus−containing groups 3
structural constituent of muscle 3
ATP binding 5
protein binding 27
actin binding 3
extracellular vesicular exosome 16
intracellular membrane−bounded organelle 2
sarcomere 3
PML body 2
integral component of membrane 4
membrane 8
plasma membrane 7
cytosol 17
mitochondrion 4
cytoplasm 14
nucleus 17
extracellular space 11
extracellular region 12
ventricular cardiac muscle tissue morphogenesis 2
response to glucocorticoid 2
regulation of angiogenesis 2
innate immune response 3
small molecule metabolic process 5
response to drug 2
organ regeneration 2
negative regulation of cell growth 3
extracellular matrix organization 6
muscle filament sliding 5
response to organonitrogen compound 2
regulation of striated muscle contraction 2
muscle contraction 6
protein phosphorylation 5
angiogenesis 2
molecular_function
cellular_component
biological_process
0 5 10 15 20 25 30 35 40 45 50 55 60 65 70 75
Percent of Genes (%)
KEGG Pathway Classification of lncRNA targets
Digestive System 2
Endocrine System 3
Environmental Adaptation 1
Development 2
Circulatory System 5
Immune System 11
Metabolism of Cofactors and Vitamins 2
Carbohydrate Metabolism 1
Amino Acid Metabolism 3
Cardiovascular Diseases 6
Immune Diseases 7
Cancers 3
Infectious Diseases 13
Neurodegenerative Diseases 2
Endocrine and Metabolic Diseases 1
Folding, Sorting and Degradation 2
Translation 3
Signaling Molecules and Interaction 7
Signal Transduction 7
Membrane Transport 1
Cell Motility 2
Cell Communication 5
Transport and Catabolism 4
Cell Growth and Death 4
Organismal Systems
Metabolism
Human Diseases
Genetic Information Processing
Environmental Information Processing
Cellular Processes
0 5 10 15 20 25 30 35
Percent of Genes (%)

Figure S3. Differential expression levels of serum lactate between 56 MELAS patients and 34 Controls. Mann-Whitney U test were used to determine statistical significance. Data are presented as the mean±SD. *P <0.001 versus control group.

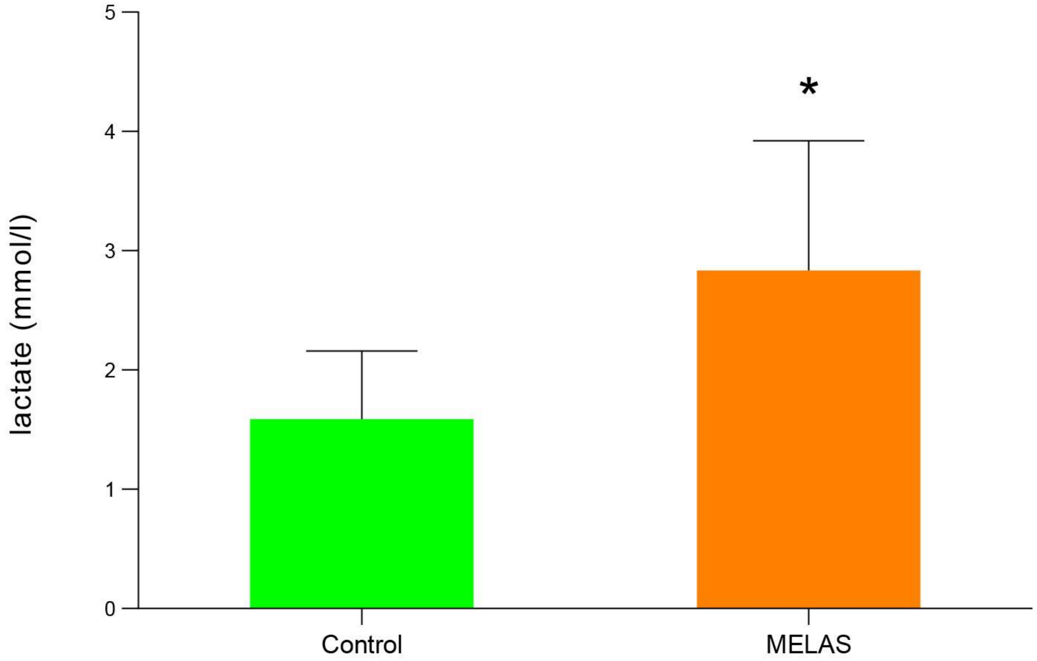

Supplementary Table S1. The characteristics of the study population

	Discovery and training set			Muscle validation set			Serum validation set			Serum validation set		
	MELAS with A3243G (20)	Controls (20)	P	MELAS with A3243G (54)	Controls (49)	P	MELAS with A3243G (34)	Controls (34)	P	MELAS with non-A3243G (22)	Controls (34)	P
Age at examination (years) (mean ± SD)	31.6±10.0	32.7±6.1	0.76	26.2±7.3	27.2±3.5	0.35	22.6±5.5	23.8±5.3	0.38	21.3±5.2	23.8±5.3	0.06
Average disease course (years) (mean± SD)	14.3±5.1	NA		14.9±5.9	NA		13.1±6.8	NA		12.8.1±6.2	NA	
Sex (male:female)	10:10	10:10	1	25:29	20:29	0.58	17:17	19:15	0.63	13:9	19:15	0.81
BMI (mean± SD)(kg/m^2)	17.7± 1.3	22.3±2.1	<0.001	18.3±1.2	21.4±1.8	<0.001	16.3±1.7	21.6±1.9	<0.001	17.3±2.7	21.6±1.9	<0.001
NMDAS score (mean± SD)	19.9±3.2	NA		18.3±3.1	NA		16.1±6.0	NA		16.4±4.0	NA	
Lactate(mmol/l)	3.3±0.6	1.7±0.5	<0.001	3.2±0.7	1.6±0.8	<0.001	3.0±1.3	1.6±0.6	<0.001	2.6±0.7	1.6±0.6	<0.001

Abbreviations: SD, standard deviation; BMI, body mass index; NMDAS,Newcastle Mitochondrial Disease Adult Scale; NA, not applicable.

Supplementary Table S2A.

Differential expression of miRNAs between MELAS patients vs controls.

Reporter Name	Fold Change	Regulation	Log 2 (G2/G1)	p-value (chi square)	Control Signal	MElAS Signal	Accession No.
hsa-miR-4734	1.25	down	-0.32	0.00E+00	60,327	48,373	MIMAT0019859
hsa-miR-1-3p	1.27	down	-0.35	0.00E+00	32,883	25,793	MIMAT0000416
hsa-miR-133a-3p	1.32	up	0.40	0.00E+00	21,304	28,145	MIMAT0000427
hsa-miR-133b	1.29	up	0.37	0.00E+00	20,236	26,126	MIMAT0000770
hsa-miR-26a-5p	1.33	down	-0.41	0.00E+00	13,032	9,806	MIMAT0000082
hsa-let-7a-5p	1.74	up	0.80	0.00E+00	7,461	12,958	MIMAT0000062
hsa-let-7d-5p	1.72	up	0.78	0.00E+00	6,573	11,277	MIMAT0000065
hsa-miR-24-3p	1.57	down	-0.65	0.00E+00	6,183	3,929	MIMAT0000080
hsa-miR-16-5p	2.37	down	-1.24	0.00E+00	4,331	1,827	MIMAT0000069
hsa-miR-26b-5p	1.78	down	-0.84	0.00E+00	3,281	1,838	MIMAT0000083
hsa-let-7c-5p	2.05	up	1.04	0.00E+00	3,110	6,373	MIMAT0000064
hsa-miR-7641	2.04	down	-1.03	0.00E+00	2,853	1,401	MIMAT0029782
hsa-miR-4267	15.25	down	-3.93	0.00E+00	2,318	152	MIMAT0016893
hsa-let-7b-5p	1.89	up	0.92	0.00E+00	2,145	4,050	MIMAT0000063
hsa-miR-4454	3.10	down	-1.63	0.00E+00	2,132	688	MIMAT0018976
hsa-miR-486-5p	2.60	up	1.38	0.00E+00	1,599	4,163	MIMAT0002177
hsa-miR-5096	6.10	down	-2.61	0.00E+00	1,139	187	MIMAT0020603
hsa-miR-145-5p	2.10	up	1.07	0.00E+00	1,127	2,371	MIMAT0000437
hsa-miR-6125	5.01	down	-2.32	0.00E+00	736	147	MIMAT0024598
hsa-miR-92a-3p	2.63	up	1.39	0.00E+00	602	1,583	MIMAT0000092
hsa-let-7e-5p	3.79	up	1.92	0.00E+00	391	1,484	MIMAT0000066
hsa-miR-4459	1121	up	18.66	0.00E+00	1	1,121	MIMAT0018981
hsa-miR-1-5p	594	up	18.00	0.00E+00	1	594	MIMAT0031892
hsa-miR-7847-3p	554	up	17.93	0.00E+00	1	554	MIMAT0030422
hsa-miR-206	1.34	up	0.42	1.20E-84	9,621	12,859	MIMAT0000462
hsa-miR-6089	2.81	down	-1.49	4.65E-82	1,121	398	MIMAT0023714
hsa-miR-92b-3p	3.95	up	1.98	1.75E-75	203	801	MIMAT0003218
hsa-miR-6087	2.59	down	-1.37	3.08E-75	1,154	446	MIMAT0023712
hsa-miR-30c-5p	1.47	up	0.55	4.12E-69	4,099	6,015	MIMAT0000244
hsa-miR-3665	1.78	down	-0.83	1.26E-67	2,191	1,231	MIMAT0018087
hsa-miR-6727-5p	1.74	down	-0.80	6.59E-62	2,142	1,233	MIMAT0027355
hsa-miR-378e	1.30	down	-0.38	2.50E-61	7,134	5,492	MIMAT0018927
hsa-miR-378g	1.40	down	-0.49	2.72E-56	4,287	3,055	MIMAT0018937
hsa-let-7g-5p	1.47	up	0.55	1.02E-55	3,307	4,849	MIMAT0000414
hsa-miR-378f	1.65	down	-0.72	1.13E-53	2,158	1,307	MIMAT0018932
hsa-miR-150-5p	3.60	up	1.85	3.16E-47	149	538	MIMAT0000451
hsa-let-7i-5p	1.74	up	0.80	4.29E-47	1,155	2,015	MIMAT0000415
hsa-miR-3960	1.32	down	-0.40	8.49E-39	4,072	3,088	MIMAT0019337
hsa-miR-27b-3p	2.25	down	-1.17	4.64E-37	696	309	MIMAT0000419
hsa-miR-378i	1.36	down	-0.44	1.19E-36	3,265	2,406	MIMAT0019074
hsa-miR-30a-5p	1.42	down	-0.50	1.47E-32	2,321	1,638	MIMAT0000087

hsa-miR-15b-5p	1.54	down	-0.62	2.29E-32	1,638	1,066	MIMAT0000417
hsa-miR-3197	2.01	up	1.00	4.59E-32	455	912	MIMAT0015082
hsa-miR-126-3p	1.58	down	-0.66	1.89E-31	1,447	918	MIMAT0000445
hsa-miR-4324	1.83	up	0.88	3.18E-31	612	1,123	MIMAT0016876
hsa-miR-195-5p	1.96	down	-0.97	3.51E-31	769	392	MIMAT0000461
hsa-miR-3196	1.84	down	-0.88	1.02E-26	762	414	MIMAT0015080
hsa-miR-7704	1.47	down	-0.55	5.93E-26	1,572	1,073	MIMAT0030019
hsa-miR-23c	1.44	up	0.53	9.64E-24	1,506	2,172	MIMAT0018000
hsa-miR-638	1.59	down	-0.67	9.22E-23	999	629	MIMAT0003308
hsa-miR-4508	1.72	down	-0.78	2.17E-20	689	401	MIMAT0019045
hsa-miR-214-3p	1.41	down	-0.49	6.96E-20	1,422	1,011	MIMAT0000271
hsa-miR-4497	1.79	down	-0.84	1.12E-18	557	311	MIMAT0019032
hsa-miR-499a-5p	1.40	down	-0.49	3.47E-17	1,237	883	MIMAT0002870
hsa-miR-7977	1.27	down	-0.34	6.08E-16	2,033	1,604	MIMAT0031180
hsa-miR-128-3p	1.47	up	0.55	7.84E-15	812	1,191	MIMAT0000424
hsa-miR-22-3p	1.20	down	-0.26	2.19E-14	2,754	2,293	MIMAT0000077
hsa-let-7f-5p	1.13	up	0.18	8.85E-14	11,853	13,420	MIMAT0000067
hsa-miR-125a-5p	1.56	up	0.64	1.14E-13	521	813	MIMAT0000443
hsa-miR-30e-5p	1.47	down	-0.55	2.76E-13	755	515	MIMAT0000692
hsa-miR-4516	1.48	down	-0.56	1.24E-12	690	467	MIMAT0019053
hsa-miR-133a-5p	1.52	up	0.60	1.93E-12	548	831	MIMAT0026478
hsa-miR-5100	1.47	down	-0.55	6.44E-11	604	412	MIMAT0022259
hsa-miR-4443	1.24	down	-0.31	6.46E-11	1,531	1,232	MIMAT0018961
hsa-miR-378c	1.19	down	-0.25	1.81E-09	1,833	1,538	MIMAT0016847
hsa-miR-1260b	1.38	up	0.47	8.68E-08	572	791	MIMAT0015041
hsa-miR-486-3p	1.22	up	0.29	2.10E-07	1,667	2,041	MIMAT0004762
hsa-miR-199a-3p	1.44	up	0.53	1.10E-06	359	517	MIMAT0000232
hsa-miR-30d-5p	1.22	up	0.28	2.22E-06	1,492	1,816	MIMAT0000245
hsa-miR-29a-3p	1.22	up	0.29	9.57E-06	1,268	1,547	MIMAT0000086
hsa-miR-378a-3p	1.10	up	0.14	2.23E-05	8,195	9,018	MIMAT0000732
hsa-miR-10b-5p	1.13	down	-0.18	4.58E-05	1,464	1,294	MIMAT0000254

Supplementary Table S2B.

Differential expression of lncRNAs between MELAS patients vs controls.

gene_name	MELAS_rpkm	Control_rpkm	log2 fold_change	p value	chr	strand	ensembl_gene_id
AC002398.12	11.01	75.00	-2.77	0.00	chr19	+	ENSG00000267328.1
AC003991.3	8.93	35.97	-2.01	0.00	chr7	+	ENSG00000228113.5
AC007228.9	64.77	32.35	1.00	0.00	chr19	-	ENSG00000268568.1
AC009501.4	16749.76	7336.17	1.19	0	chr2	-	ENSG00000231609.3
AC011239.1	28.15	6.10	2.21	0.00	chr2	-	ENSG00000224361.1
AC064871.3	16.01	0.52	4.95	0.00	chr2	-	ENSG00000224643.3
AC073254.1	54.53	18.06	1.59	0.00	chr2	-	ENSG00000237126.6
AC079586.1	33.00	7.78	2.08	0.00	chr2	-	ENSG00000204460.3

AC090616.2	137.85	333.64	-1.28	0.00	chr17	-	ENSG00000214708.4
AC123886.2	22.52	3.98	2.50	0.00	chr2	+	ENSG00000231536.1
AC131097.4	2.57	19.08	-2.89	0.00	chr2	-	ENSG00000216921.5
BCYRN1	58.77	23.64	1.31	0.00	chr2	+	ENSG00000236824.1
CTA-14H9.5	29.62	7.53	1.98	0.00	chr6	+	ENSG00000261353.1
CTC-250I14.6	16.44	52.80	-1.68	0.00	chr19	-	ENSG00000267598.1
CTC-260E6.6	20.57	2.00	3.36	0.00	chr19	-	ENSG00000267383.4
CTC-297N7.7	119.66	43.28	1.47	0.00	chr17	+	ENSG00000214970.6
CTC-340A15.2	83.54	39.69	1.07	0.00	chr5	+	ENSG00000241956.7
CTC-444N24.7	26.05	72.66	-1.48	0.00	chr19	-	ENSG00000279541.1
CTC-537E7.2	11.24	0	Inf	0.00	chr5	-	ENSG00000248359.1
CTD-2033D15.1	0	62.00	-Inf	0.00	chr15	-	ENSG00000259279.1
CTD-2033D15.3	13.81	75.66	-2.45	0.00	chr15	-	ENSG00000278621.1
CTD-2201G16.1	526.36	3514.13	-2.74	0	chr14	+	ENSG00000258444.1
CTD-2210P15.3	31.51	67.29	-1.09	0.00	chr5	-	ENSG00000279557.1
CTD-2369P2.8	3.70	28.17	-2.93	0.00	chr19	-	ENSG00000267607.1
CTD-3064M3.7	1.55	28.55	-4.20	0.00	chr8	+	ENSG00000271959.1
CTD-3074O7.2	37.57	198.33	-2.40	0.00	chr11	-	ENSG00000250105.1
CTD-3149D2.4	0	10.95	-Inf	0.00	chr19	-	ENSG00000268112.1
CYP1B1-AS1	13.62	37.88	-1.48	0.00	chr2	+	ENSG00000232973.9
DYX1C1-CCPG1	65.36	25.80	1.34	0.00	chr15	-	ENSG00000261771.3
EAF1-AS1	0	10.80	-Inf	0.00	chr3	-	ENSG00000249786.5
GABARAPL3	38.21	90.60	-1.25	0.00	chr15	+	ENSG00000279980.1
KB-1732A1.1	0	11.16	-Inf	0.00	chr8	+	ENSG00000253669.3
LINC00312	10.95	47.75	-2.12	0.00	chr3	+	ENSG00000237697.2
LINC00685	1310.18	537.68	1.28	0.00	chrX	+	ENSG00000226179.3
LINC01405	29.15	483.01	-4.05	0.00	chr12	+	ENSG00000185847.5
LINCMD1	24.82	3.86	2.69	0.00	chr6	-	ENSG00000225613.2
MIR22HG	12.46	42.34	-1.76	0.00	chr17	-	ENSG00000186594.10
MYHAS	126.50	44.61	1.50	0.00	chr17	+	ENSG00000272975.1
RNU12	76.00	25.35	1.58	0.00	chr22	+	ENSG00000270022.3
RP11-102K13.5	38.86	184.50	-2.25	0.00	chr13	-	ENSG00000278309.1
RP11-1100L3.8	32.83	107.50	-1.71	0.00	chr12	-	ENSG00000259884.1
RP11-124N14.3	405.59	149.48	1.44	0.00	chr10	-	ENSG00000234961.1
RP11-142G1.3	78.59	37.41	1.07	0.00	chr16	-	ENSG00000260975.1
RP11-152P23.2	3.11	19.80	-2.67	0.00	chr16	-	ENSG00000260350.1
RP11-178L8.3	41.60	16.63	1.32	0.00	chr16	-	ENSG00000261592.1
RP11-203M5.8	1.55	48.69	-4.97	0.00	chr14	-	ENSG00000258908.1
RP11-255P5.3	12.70	46.66	-1.88	0.00	chr13	+	ENSG00000278445.1
RP11-259O2.3	204.85	95.19	1.11	0.00	chr5	+	ENSG00000249731.1
RP11-289F5.1	64.14	30.40	1.08	0.00	chr9	-	ENSG00000226798.1
RP11-309L24.2	23.94	80.76	-1.75	0.00	chr7	-	ENSG00000242902.1
RP11-323N12.5	157.30	52.87	1.57	0.00	chr17	+	ENSG00000267601.1
RP11-334A14.5	42.57	14.41	1.56	0.00	chr1	-	ENSG00000232993.1
RP11-338K17.8	10.72	0	Inf	0.00	chr12	-	ENSG00000255839.1
RP11-343H19.2	123.16	334.36	-1.44	0.00	chr16	+	ENSG00000259827.1

RP11-345P4.9	14.03	38.85	-1.47	0.00	chr1	-	ENSG00000272106.1
RP11-355B11.2	43.87	16.50	1.41	0.00	chr2	+	ENSG00000270820.3
RP11-357D18.1	108.69	17.12	2.67	0.00	chr5	-	ENSG00000250978.3
RP11-373D23.2	1.29	15.38	-3.57	0.00	chr2	+	ENSG00000270640.1
RP11-379B18.5	75.14	31.26	1.27	0.00	chr3	-	ENSG00000241288.5
RP11-382J12.1	58.32	27.28	1.10	0.00	chr8	+	ENSG00000246366.4
RP11-386G11.3	50.70	21.29	1.25	0.00	chr12	+	ENSG00000258283.1
RP11-386J22.3	64.29	19.64	1.71	0.00	chr9	+	ENSG00000274421.1
RP11-388M20.6	14.86	41.48	-1.48	0.00	chr16	-	ENSG00000260304.1
RP11-403P17.4	814.92	63.52	3.68	0	chr16	+	ENSG00000261519.3
RP11-423H2.3	58.70	27.54	1.09	0.00	chr5	+	ENSG00000249684.3
RP11-43D4.3	227.58	52.91	2.10	0.00	chr12	+	ENSG00000279176.1
RP11-442H21.2	65.90	18.93	1.80	0.00	chr10	-	ENSG00000269926.1
RP11-451G4.2	599.95	1373.02	-1.19	0.00	chr3	-	ENSG00000240045.1
RP11-466A19.1	51.80	19.59	1.40	0.00	chr17	+	ENSG00000266718.1
RP11-46J23.1	0	10.63	-Inf	0.00	chr4	-	ENSG00000272986.1
RP11-480G7.1	25.60	70.52	-1.46	0.00	chr16	-	ENSG00000261788.1
RP11-480I12.7	251.44	514.13	-1.03	0.00	chr1	+	ENSG00000234996.3
RP11-521L9.1	29.41	7.62	1.95	0.00	chr16	-	ENSG00000260999.1
RP11-532N4.2	64.90	29.59	1.13	0.00	chr6	+	ENSG00000235535.5
RP11-554I8.2	15.01	45.77	-1.61	0.00	chr10	+	ENSG00000223784.1
RP11-603J24.14	43.19	231.72	-2.42	0.00	chr12	-	ENSG00000257809.1
RP11-617F23.1	26.02	60.48	-1.22	0.00	chr15	-	ENSG00000259291.2
RP11-627J17.1	22.56	3.25	2.80	0.00	chr3	-	ENSG00000272149.1
RP11-638I2.8	18.87	52.46	-1.48	0.00	chr14	+	ENSG00000258666.1
RP11-656E20.5	1.16	19.26	-4.05	0.00	chr12	-	ENSG00000255958.1
RP11-66B24.4	10.59	57.19	-2.43	0.00	chr15	-	ENSG00000259583.2
RP11-67L3.5	15.96	72.14	-2.18	0.00	chr1	+	ENSG00000242396.1
RP11-69M1.6	323.98	139.58	1.21	0.00	chr12	-	ENSG00000279865.1
RP11-701H24.7	68.47	29.55	1.21	0.00	chr15	+	ENSG00000271347.1
RP11-705C15.5	40.41	89.82	-1.15	0.00	chr12	+	ENSG00000272917.1
RP11-731F5.2	32.10	5.29	2.60	0.00	chr14	-	ENSG00000253364.1
RP11-762H8.4	23.83	64.39	-1.43	0.00	chr15	-	ENSG00000272418.1
RP1-178F15.4	103.90	399.66	-1.94	0.00	chr1	-	ENSG00000272030.1
RP11-81K2.1	20.61	4.25	2.28	0.00	chr17	+	ENSG00000262039.1
RP11-91P24.7	42.48	100.86	-1.25	0.00	chr11	-	ENSG00000254459.1
RP11-946L20.4	106.91	50.38	1.09	0.00	chr8	-	ENSG00000253103.1
RP11-96A15.1	15.08	142.59	-3.24	0.00	chr18	-	ENSG00000278464.1
RP11-96L14.7	20.70	49.17	-1.25	0.00	chr1	-	ENSG00000236782.3
RP11-977G19.14	29.62	104.48	-1.82	0.00	chr12	-	ENSG00000258260.1
RP1-20B21.4	30.73	7.64	2.01	0.00	chr1	+	ENSG00000227751.1
RP1-309I22.2	43.69	17.28	1.34	0.00	chr22	+	ENSG00000279338.1
RP13-270P17.1	63.72	141.56	-1.15	0.00	chr18	-	ENSG00000264235.3
RP1-56K13.3	46.95	16.93	1.47	0.00	chr17	-	ENSG00000265784.1
RP1-67K17.3	18.62	46.52	-1.32	0.00	chr6	+	ENSG00000233138.1
RP4-598P13.1	91.26	41.45	1.14	0.00	chr1	-	ENSG00000248458.2

SHANK2-AS3	407.43	161.05	1.34	0.00	chr11	+	ENSG00000171671.6
SNHG1	55.31	22.82	1.28	0.00	chr11	-	ENSG00000255717.4
SNHG12	44.19	2.79	3.99	0.00	chr1	-	ENSG00000197989.11
SNHG8	255.14	121.52	1.07	0.00	chr4	+	ENSG00000269893.4
SNHG9	38.55	8.62	2.16	0.00	chr16	+	ENSG00000255198.4
SNORA76C	382.30	44.16	3.11	0.00	chr17	+	ENSG00000266402.3
SSTR5-AS1	270.06	106.42	1.34	0.00	chr16	-	ENSG00000261713.4
XXbac-BPG252P(	5.80	97.31	-4.07	0.00	chr6	+	ENSG00000272273.1
ZNF252P-AS1	174.69	71.54	1.29	0.00	chr8	+	ENSG00000255559.1

Supplementary Table S2C.

Differential expression of mRNAs between MELAS patients vs controls.

Gene Symbol	MEAS_count	Control_count	MEAS_rpkm	Control_rpkm	log2fold_change	p value	Accession
ABRA	889	5705.75	27.96	75.87	-1.44	0.00	ENST00000311955.3
ACTA1	52946.83	260333.01	4089.46	8500.37	-1.06	0	ENST00000366683.3
ACTN3	764.03	8515.04	21.47	101.15	-2.24	0.00	ENST00000502692.3
ADAMTS1	382	2600.83	6.38	18.36	-1.53	0.02	ENST00000284984.5
ADAMTS4	4	785.50	0.18	14.67	-6.38	0.00	ENST00000367995.3
ADAMTS9	12.83	363.67	0.39	4.64	-3.58	0.03	ENST00000459780.1
ADRB2	128.91	1903.78	3.24	20.26	-2.64	0.00	ENST00000305988.5
AGT	193	2066	7.30	33.04	-2.18	0.00	ENST00000366667.4
AL161784.1	25.67	136.17	12.36	27.71	-1.17	0.01	ENST00000624625.1
ALDH1A3	137.17	1209.08	3.03	11.29	-1.90	0.04	ENST00000329841.7
ANKRD1	1191	39879	52.15	738.24	-3.82	0	ENST00000371697.3
ANKRD2	47.25	446.25	3.82	15.25	-2.00	0.01	ENST00000455090.1
ANKRD37	50.50	434	3.84	13.93	-1.86	0.02	ENST00000335174.5
AQP4	799.33	3940.70	13.13	27.37	-1.06	0.03	ENST00000383168.6
ARID5A	64.17	766.33	2.54	12.83	-2.34	0.01	ENST00000357485.5
ARRDC4	225	1609	4.79	14.48	-1.60	0.04	ENST00000268042.6
ATF3	2.24	278.87	0.29	15.02	-5.72	0.00	ENST00000366981.6
ATP2A2	198.18	1600.73	32.47	110.86	-1.77	0.00	ENST00000552636.1
BCL3	19.50	430	0.83	7.73	-3.22	0.02	ENST00000164227.7
BHLHE40	681	7661	16.99	80.81	-2.25	0.00	ENST00000256495.3
BTG2	599.50	15987.50	19.16	215.97	-3.49	0.00	ENST00000290551.4
C10orf10	272	3702	11.12	63.97	-2.52	0.00	ENST00000298295.3
C11orf96	42.67	698.50	2.86	19.78	-2.79	0.00	ENST00000617612.1
CALML6	45.50	530	3.58	17.64	-2.30	0.00	ENST00000307786.5
CASQ2	589	2836	19.09	38.85	-1.03	0.01	ENST00000261448.5
CCDC71L	21	422	1.16	9.82	-3.09	0.01	ENST00000315965.6
CCL2	14.50	741.50	1.71	36.91	-4.43	0.00	ENST00000580907.3
CCL8	7	159	0.48	4.62	-3.26	0.03	ENST00000394620.1
CCNL1	4.25	117.03	0.46	5.41	-3.54	0.03	ENST00000468977.3
CCRN4L	62.35	1063.84	3.06	22.06	-2.85	0.00	ENST00000280614.2
CDKN1A	39	359.53	4.26	16.61	-1.96	0.01	ENST00000373711.3
CEBPD	299.58	3918.33	11.92	65.91	-2.47	0.00	ENST00000408965.3

CISH	12.50	1350	0.53	24.21	-5.51	0.00	ENST00000348721.3
CKMT2	145.37	794.63	23.07	53.32	-1.21	0.00	ENST00000505060.1
COQ10A	165.20	1016.65	10.15	26.42	-1.38	0.01	ENST00000433805.4
CRYAB	122.06	581.52	29.96	60.35	-1.01	0.00	ENST00000528961.3
CRYM	6.43	222.15	0.38	5.53	-3.87	0.02	ENST00000219599.5
CSRNP1	85	1447	2.34	16.84	-2.85	0.00	ENST00000273153.7
CSRP3	2201.03	12618.09	131.00	317.49	-1.28	0.00	ENST00000533783.1
CTSL	60.83	625.01	5.65	24.54	-2.12	0.00	ENST00000342020.5
CXCL1	2.50	378.17	0.20	12.63	-6.00	0.00	ENST00000395761.3
CXCL10	10	603	0.74	18.79	-4.67	0.00	ENST00000306602.2
CXCL2	10.50	813.17	0.75	24.46	-5.03	0.00	ENST00000508487.2
CXCL3	0	137.67	0	4.69	-Inf	0.03	ENST00000296026.4
CXCL8	2	359	0.25	18.79	-6.25	0.00	ENST00000401931.1
DGKD	9.85	140.58	1.77	10.69	-2.59	0.01	ENST00000427930.3
DHCR24	34.67	253.33	3.48	10.75	-1.63	0.04	ENST00000436604.1
DUSP2	12	300	0.62	6.51	-3.40	0.04	ENST00000288943.4
EGR1	184	8264.67	5.08	96.49	-4.25	0.00	ENST00000239938.4
FAM189A2	50.75	491	3.18	12.99	-2.03	0.01	ENST00000377216.3
FAM46C	504	3595	7.59	22.90	-1.59	0.01	ENST00000369448.3
FOS	10.08	782.52	1.39	45.58	-5.04	0.00	ENST00000555242.1
FOSL2	41.50	432.83	4.05	17.84	-2.14	0.00	ENST00000436647.1
GADD45A	21.67	493.17	2.34	22.50	-3.27	0.00	ENST00000370985.3
GADD45B	0.90	687.15	0.28	91.21	-8.33	0.00	ENST00000587887.3
GADD45G	39.50	849.50	3.50	31.85	-3.18	0.00	ENST00000375769.1
GJA1	163	1859	4.65	22.43	-2.27	0.00	ENST00000282561.3
GLUL	651.07	5870.80	45.35	172.89	-1.93	0.00	ENST00000621524.1
GPT2	38.33	416.17	6.00	27.52	-2.20	0.00	ENST00000562132.3
HBEGF	167	2319	6.07	35.61	-2.55	0.00	ENST00000230990.6
HES1	185	2667	10.16	61.92	-2.61	0.00	ENST00000232424.3
HES6	2.56	93.20	0.42	6.44	-3.95	0.02	ENST00000450098.1
HMGB3	305.08	1593.69	33.34	73.63	-1.14	0.00	ENST00000455596.3
HMOX1	144.94	2427.97	6.89	48.82	-2.82	0.00	ENST00000216117.8
HSPA1A	1640.50	18808.18	57.26	277.51	-2.28	0.00	ENST00000375651.6
HSPA1B	1765.50	20690.18	60.69	300.67	-2.31	0.00	ENST00000375650.4
HSPA1L	160.88	1342.78	5.48	19.34	-1.82	0.01	ENST00000375654.4
HSPB6	72.83	641.33	4.33	16.11	-1.90	0.01	ENST00000004982.4
HSPB7	94.57	465.60	14.11	29.36	-1.06	0.03	ENST00000463576.3
HSPB8	125	592	17.01	34.05	-1.00	0.02	ENST00000541798.1
ICAM1	12.83	437.50	0.86	12.37	-3.85	0.00	ENST00000423829.2
IER3	15	441.50	0.96	11.98	-3.64	0.00	ENST00000376377.2
IER5	238.53	3666.10	4.94	32.07	-2.70	0.00	ENST00000367577.6
IL17D	99.50	1443	4.63	28.41	-2.62	0.00	ENST00000304920.3
IL1B	2	433	0.11	9.73	-6.52	0.00	ENST00000263341.4
IL6	0	101.53	0	4.86	-Inf	0.03	ENST00000401630.5
IRF1	6.30	177.62	0.66	7.82	-3.58	0.02	ENST00000437654.3
JUNB	306	13773	14.57	277.24	-4.25	0.00	ENST00000302754.5

KLF10	435	4763.50	12.17	56.35	-2.21	0.00	ENST00000285407.8
KLF9	1381	6511	23.13	46.09	-1.00	0.01	ENST00000377126.3
KLHL40	1866	12572	64.25	182.99	-1.51	0.00	ENST00000287777.4
LIF	15	677	0.33	6.25	-4.25	0.02	ENST00000249075.3
LMCD1	169.15	1274.21	16.85	53.66	-1.67	0.00	ENST00000415597.3
LPL	389.47	2230.23	8.78	21.24	-1.28	0.03	ENST00000311322.8
MAT2A	661	4352	20.32	56.56	-1.48	0.00	ENST00000306434.5
MB	1979.36	11939.62	314.74	802.59	-1.35	0.00	ENST00000442617.1
MID1IP1	245.08	1645.75	11.17	31.70	-1.51	0.00	ENST00000457894.3
MIDN	26.75	369.25	1.99	11.63	-2.54	0.01	ENST00000586757.3
MIR1-1HG	305.67	2257.09	21.73	67.83	-1.64	0.00	ENST00000370527.5
MT1A	15.86	184.83	3.47	17.10	-2.30	0.00	ENST00000290705.10
MT1E	57.45	407.29	7.07	21.19	-1.58	0.01	ENST00000306061.8
MT1G	16.78	163.54	2.32	9.54	-2.04	0.02	ENST00000568675.1
MT1M	60.08	507.97	6.28	22.45	-1.84	0.00	ENST00000379818.3
MT1X	268.04	2098.17	42.31	140.01	-1.73	0.00	ENST00000564974.1
MT2A	92.97	1036.87	13.87	65.38	-2.24	0.00	ENST00000561491.1
MTRNR2L1	276.38	2238.77	15.42	52.81	-1.78	0.00	ENST00000540040.2
MTRNR2L10	77.16	590.58	4.37	14.14	-1.69	0.02	ENST00000545075.2
MTRNR2L6	38.53	476.75	2.31	12.07	-2.39	0.01	ENST00000604952.1
MTRNR2L9	397.53	2095.03	65.37	145.64	-1.16	0.00	ENST00000544932.1
MYC	1.98	199.00	0.30	12.64	-5.41	0.00	ENST00000520751.1
MYH2	2471.21	12832.13	317.27	696.46	-1.13	0.00	ENST00000420805.1
MYH6	4681.23	62974.91	68.28	388.34	-2.51	0.00	ENST00000405093.5
MYH7	27780.54	359342.42	395.51	2162.75	-2.45	0	ENST00000355349.3
MYL2	3621	34428	472.58	1899.53	-2.01	0	ENST00000548438.1
MYL3	711.00	17114.50	66.68	678.57	-3.35	0	ENST00000292327.4
MYL6B	316.83	4893.42	26.55	173.38	-2.71	0.00	ENST00000550443.3
MYLK3	166.50	1210	4.37	13.43	-1.62	0.03	ENST00000394809.6
MYOM3	326.34	1736.34	10.50	23.62	-1.17	0.03	ENST00000338909.7
NAMPT	209.75	1707.25	14.99	51.56	-1.78	0.00	ENST00000354289.6
NFIL3	154	4095	6.40	71.95	-3.49	0.00	ENST00000297689.3
NMRK2	52.98	592.03	5.28	24.96	-2.24	0.00	ENST00000593949.1
NNMT	34.50	1087.88	1.86	24.75	-3.74	0.00	ENST00000299964.3
NR4A3	8.83	627.25	0.20	6.06	-4.91	0.02	ENST00000395097.4
OTUD1	176	3070	5.20	38.35	-2.88	0.00	ENST00000376495.4
PDE4B	39.39	1577.02	1.08	18.20	-4.08	0.00	ENST00000341517.6
PDK4	956	8959	23.17	91.78	-1.99	0.00	ENST00000005178.5
PDLIM1	272	1757	16.23	44.33	-1.45	0.00	ENST00000329399.6
PFKFB3	257.33	3209.61	5.22	27.53	-2.40	0.00	ENST00000536985.3
PLAU	60	1319.58	2.24	20.83	-3.22	0.00	ENST00000372764.3
PNP	15	522.67	0.86	12.69	-3.88	0.00	ENST00000361505.7
POPDC2	85.35	654.05	4.41	14.27	-1.70	0.02	ENST00000264231.5
PPP1R27	549.73	3898.55	33.01	98.98	-1.58	0.00	ENST00000570394.1
PTX3	4	673	0.18	12.71	-6.15	0.00	ENST00000295927.3
RASSF5	37.72	1151.13	0.93	12.06	-3.69	0.00	ENST00000577571.3

RGS16	14	6074	0.50	91.69	-7.52	0.00	ENST00000367558.5
RHOU	245	2102	4.86	17.61	-1.86	0.01	ENST00000366691.3
RND3	3.30	94.80	0.59	7.22	-3.60	0.04	ENST00000454202.3
RNF122	180	1265	8.35	24.81	-1.57	0.00	ENST00000256257.1
RP11-249C24.12	40.68	343.89	14.57	52.06	-1.84	0.00	ENST00000379816.5
RRAD	215.17	3212.25	38.53	243.15	-2.66	0.00	ENST00000566577.1
S100A1	55.25	333.75	20.29	51.81	-1.35	0.00	ENST00000436839.1
SAT1	26.18	224.03	2.50	9.03	-1.85	0.04	ENST00000379253.5
SBK3	0	172	0	4.96	-Inf	0.03	ENST00000612221.1
SDC4	380	1898	12.60	26.61	-1.08	0.03	ENST00000372733.3
SERPINA3	6.50	183.45	0.44	5.21	-3.58	0.03	ENST00000556968.2
SERPINB1	246.96	6143.28	8.17	85.93	-3.39	0.00	ENST00000380739.5
SERPINE1	27.76	984.69	0.75	11.31	-3.91	0.00	ENST00000223095.4
SERTAD1	133.99	1726.32	5.49	29.92	-2.45	0.00	ENST00000357949.4
SGMS2	104.78	1271.87	1.48	7.58	-2.36	0.02	ENST00000394684.6
SLC22A4	16.75	485	0.66	8.07	-3.61	0.02	ENST00000200652.3
SLC39A14	1.20	140.71	0.18	8.92	-5.63	0.00	ENST00000520832.1
SOCS3	2	553	0.48	56.12	-6.87	0.00	ENST00000587578.1
SRGN	221	1185	15.85	35.94	-1.18	0.01	ENST00000242465.3
TAP1	251.80	1511.80	7.37	18.72	-1.34	0.02	ENST00000354258.4
TECRL	113.67	1156.03	2.76	11.85	-2.10	0.02	ENST00000381210.5
TFRC	14.67	126.75	2.43	8.86	-1.87	0.04	ENST00000421258.1
TGM2	268.83	2698.50	4.60	19.50	-2.09	0.01	ENST00000361475.4
THBD	222	29057	4.68	259.07	-5.79	0.00	ENST00000377103.2
THBS1	397.87	7772.09	4.43	36.62	-3.05	0.00	ENST00000260356.5
TM4SF1	36.25	260.10	4.47	13.57	-1.60	0.02	ENST00000493298.1
TNFAIP6	14	446	0.85	11.47	-3.75	0.00	ENST00000243347.4
TNFRSF12A	23.45	270.12	2.31	11.23	-2.28	0.01	ENST00000341627.5
TNFSF9	5	327	0.26	7.20	-4.79	0.01	ENST00000245817.4
TNNC1	2781	31471.50	331.50	1585.93	-2.26	0	ENST00000496590.1
TNNI1	1047.05	7747.62	82.49	258.03	-1.65	0.00	ENST00000367312.3
TOR3A	62.10	844.75	1.98	11.36	-2.52	0.01	ENST00000367627.5
TPM3	528.02	5843.16	25.84	120.87	-2.23	0.00	ENST00000328159.6
TRIB1	70.17	632.17	4.32	16.44	-1.93	0.01	ENST00000519576.1
TRIM63	1663	11690	81.42	241.96	-1.57	0.00	ENST00000374272.3
UGCG	205	2179	4.43	19.92	-2.17	0.00	ENST00000374279.3
XIRP1	332.33	8582.83	11.09	121.12	-3.45	0.00	ENST00000421646.1
ZFP36	12.75	628.75	2.49	51.88	-4.38	0.00	ENST00000600033.1
A2M	182.40	211.37	25.37	12.43	1.03	0.03	ENST00000404455.2
ACTA2	123	102.62	14.33	5.05	1.50	0.03	ENST00000458159.3
ADAM10	6739.74	5345.64	51.09	17.13	1.58	0.00	ENST00000260408.5
AHNAK	148.56	156.32	22.24	9.89	1.17	0.03	ENST00000531324.1
ANAPC15	200.89	85.04	11.75	2.10	2.48	0.01	ENST00000502597.2
ANGPTL7	93	28	3.60	0.46	2.97	0.04	ENST00000376819.3
APOD	790.11	341.16	72.07	13.16	2.45	0.00	ENST00000458447.3
APOE	163.33	100.58	11.72	3.05	1.94	0.02	ENST00000252486.6

ASB8	214.38	200.13	20.13	7.94	1.34	0.02	ENST00000536953.3
ASTN2	891.84	903.49	22.66	9.70	1.22	0.02	ENST00000358637.4
ATP9B	590.94	616.85	31.04	13.70	1.18	0.01	ENST00000586722.3
BCAS4	203.12	167.26	11.75	4.09	1.52	0.04	ENST00000463943.1
BLOC1S6	1005.71	1052.46	43.66	19.32	1.18	0.00	ENST00000566753.3
C1orf229	1659.49	1731.42	63.69	28.09	1.18	0.00	ENST00000408893.4
C1QA	92.67	40	7.03	1.28	2.45	0.03	ENST00000374642.5
C3	1716.50	1609.17	28.26	11.20	1.34	0.01	ENST00000245907.8
C7	455.08	38.46	9.26	0.33	4.81	0.00	ENST00000313164.9
CALM1	252.81	295.94	39.33	19.46	1.01	0.01	ENST00000557020.3
CAV1	287.78	150.30	9.22	2.04	2.18	0.03	ENST00000341049.4
CCDC3	197.50	43	5.38	0.50	3.44	0.02	ENST00000378839.1
CCPG1	78.41	45.97	8.60	2.13	2.01	0.03	ENST00000563171.3
CEBPZOS	9012.62	7362.91	247.32	85.42	1.53	0.00	ENST00000402297.3
CFAP54	687.86	742.44	21.63	9.87	1.13	0.03	ENST00000550977.2
CFD	1474	1306	106.36	39.84	1.42	0.00	ENST00000327726.8
CHRNA1	60.50	18.92	3.59	0.47	2.92	0.04	ENST00000409542.3
CIRBP	387.38	351.70	31.29	12.01	1.38	0.00	ENST00000589235.3
CNOT2	110.78	96.44	15.53	5.72	1.44	0.03	ENST00000551132.3
COL1A2	1560	1609.50	24.98	10.90	1.20	0.02	ENST00000297268.8
CPE	382.75	354.75	13.70	5.37	1.35	0.03	ENST00000402744.6
CRTAP	228.04	132.63	10.91	2.68	2.02	0.03	ENST00000449224.1
CTD-2369P2.10	283.11	204.55	12.54	3.83	1.71	0.03	ENST00000452032.4
CTSK	215.50	104	11.04	2.25	2.29	0.01	ENST00000271651.5
CXCL14	610.52	657.95	30.06	13.70	1.13	0.01	ENST00000512158.3
CYBRD1	194.25	101.92	11.28	2.50	2.17	0.03	ENST00000409484.3
DBNDD1	171.28	84.88	7.14	1.50	2.25	0.03	ENST00000002501.8
DCN	207.95	245.56	34.72	17.33	1.00	0.01	ENST00000441303.4
DDIT4	333	171	16.51	3.58	2.20	0.00	ENST00000307365.3
DDIT4L	295.00	226.33	46.23	14.99	1.62	0.00	ENST00000513992.1
DDN	896	1063	20.71	10.39	1.00	0.04	ENST00000421952.2
DHRS3	538.83	613.83	27.12	13.06	1.05	0.02	ENST00000616661.2
DHRS7	260.31	265.68	17.06	7.36	1.21	0.04	ENST00000557185.3
DNMT3A	17045.72	14522.28	155.48	56.00	1.47	0.00	ENST00000264709.5
EEF1A1	702.89	711.85	86.89	37.20	1.22	0.00	ENST00000455918.1
EIF4A2	1237.70	1459.97	54.25	27.05	1.00	0.00	ENST00000425053.3
ELOF1	164.91	122.29	24.43	7.66	1.67	0.00	ENST00000590700.3
ERAP1	420.63	161.78	7.59	1.23	2.62	0.02	ENST00000443439.4
F13A1	640.25	258	13.87	2.36	2.55	0.00	ENST00000264870.5
FABP4	106	84	14.65	4.91	1.58	0.02	ENST00000522659.1
FAM129A	7139.33	7695.69	89.30	40.69	1.13	0.00	ENST00000367511.3
FBLN1	455.86	167.91	13.64	2.12	2.68	0.00	ENST00000327858.8
FBN1	723.00	630.67	16.64	6.14	1.44	0.02	ENST00000537463.4
FMO2	491	208	11.05	1.98	2.48	0.01	ENST00000209929.7
FSIP2	632.39	649.72	20.10	8.73	1.20	0.04	ENST00000429412.1
GAS1	279	181	8.55	2.35	1.87	0.03	ENST00000298743.8

GLRX	207.20	166.60	13.14	4.47	1.56	0.03	ENST00000379979.6
GPD2	1038.80	772.71	14.90	4.69	1.67	0.02	ENST00000310454.8
GPNMB	463.83	172.67	14.55	2.29	2.67	0.00	ENST00000381990.4
GREM1	16347.14	13690.25	96.99	34.34	1.50	0.00	ENST00000622074.1
GSG2	1311.20	746.31	40.63	9.78	2.06	0.00	ENST00000325418.5
GSN	1181.83	924.56	38.55	12.75	1.60	0.00	ENST00000373818.6
HBA1	3076.42	1302.17	462.05	82.68	2.48	0.00	ENST00000320868.7
HBA2	3735.92	1489.67	535.13	90.21	2.57	0.00	ENST00000251595.8
HBB	9602.93	3530.32	1325.14	205.95	2.69	0	ENST00000335295.4
HBD	76.13	33	21.63	3.96	2.45	0.00	ENST00000417377.1
HIST1H4E	79	79.50	21.94	9.33	1.23	0.02	ENST00000615164.1
HIST1H4J	99.17	80.77	23.04	7.93	1.54	0.01	ENST00000355057.2
HIST1H4K	80.17	55.77	22.27	6.55	1.77	0.00	ENST00000611927.1
HMG20A	163.71	111.37	22.03	6.34	1.80	0.00	ENST00000558845.1
HOMER3	253.42	167.88	11.10	3.11	1.84	0.03	ENST00000539827.3
HSPG2	4037.25	4187.75	24.42	10.71	1.19	0.02	ENST00000374695.5
HTRA1	139	75	9.92	2.26	2.13	0.02	ENST00000420892.1
HYKK	497.70	229.62	10.53	2.05	2.36	0.01	ENST00000569878.3
IFI27L2	24.50	6.60	4.15	0.47	3.13	0.04	ENST00000238609.3
IGFBP4	620	654	24.42	10.89	1.17	0.02	ENST00000269593.4
IGFBP6	242.83	105.83	17.88	3.29	2.44	0.00	ENST00000548547.3
IGFBP7	1437.60	1576.05	87.30	40.46	1.11	0.00	ENST00000295666.4
IGHG2	45.99	13.89	3.52	0.45	2.97	0.04	ENST00000390545.2
IGKC	37.87	11.83	3.61	0.48	2.92	0.04	ENST00000430694.2
IMPA2	421.26	498.84	23.80	11.91	1.00	0.04	ENST00000269159.5
KCTD12	1209	1248.27	16.83	7.35	1.20	0.04	ENST00000377474.3
KIAA0368	90.25	87.08	14.51	5.92	1.29	0.04	ENST00000602978.1
KIAA1328	1082.90	1129.83	59.51	26.25	1.18	0.00	ENST00000586501.1
LAMC1	2742.50	3127.50	30.13	14.52	1.05	0.02	ENST00000258341.4
LAMP1	766	695	24.59	9.43	1.38	0.00	ENST00000332556.4
LHFP	494.75	455.65	20.32	7.91	1.36	0.02	ENST00000379589.3
LRP1	5115.38	4227.03	29.76	10.40	1.52	0.00	ENST00000243077.5
LUM	516	333	14.87	4.06	1.87	0.01	ENST00000266718.4
MAMDC2	364	211	8.72	2.14	2.03	0.03	ENST00000377182.4
MARCO	87	12	4.10	0.24	4.10	0.04	ENST00000327097.4
MEOX2	236	37	8.18	0.54	3.92	0.02	ENST00000262041.5
MGP	201.33	155.17	12.42	4.05	1.62	0.04	ENST00000539261.3
MKNK2	126.22	146.57	24.09	11.83	1.03	0.04	ENST00000589534.2
MMP14	408.50	141	9.49	1.38	2.78	0.01	ENST00000311852.8
MORC4	851.97	891.68	36.42	16.12	1.18	0.00	ENST00000604604.1
MPZ	177.17	21.25	9.07	0.46	4.30	0.00	ENST00000463290.3
MSS51	1663.62	1667.70	72.41	30.69	1.24	0.00	ENST00000487126.3
MSTN	378	176	11.61	2.28	2.34	0.01	ENST00000260950.4
MYH1	212930.67	113783.27	3063.18	691.98	2.15	0	ENST00000226207.5
MYH4	24891.56	23584.70	358.56	143.62	1.32	0.00	ENST00000255381.2
MYL9	148.39	100.86	12.56	3.61	1.80	0.03	ENST00000346786.2

MYOC	380.50	151	15.74	2.64	2.58	0.00	ENST00000037502.8
MYOG	535	414	30.34	9.93	1.61	0.00	ENST00000241651.4
NEK7	1526.15	1794.23	31.88	15.84	1.01	0.02	ENST00000367385.6
PF4	33	5	4.73	0.30	3.96	0.02	ENST00000296029.3
PMP22	345.68	340.94	16.55	6.90	1.26	0.04	ENST00000312280.5
PPBP	91	21	11.03	1.08	3.36	0.00	ENST00000296028.3
PRDX1	671.32	691.65	56.98	24.82	1.20	0.00	ENST00000319248.10
PRKCDBP	123.50	67.50	9.12	2.11	2.11	0.03	ENST00000303927.3
PRRG3	28645.03	23810.68	415.32	145.95	1.51	0.00	ENST00000370353.3
PTGDS	89.25	73.33	11.70	4.06	1.53	0.04	ENST00000457950.3
PVALB	44.15	5.72	6.43	0.35	4.19	0.01	ENST00000216200.7
RNASE1	156.87	117.43	15.17	4.80	1.66	0.02	ENST00000412779.2
RP11-47I22.4	156.40	148.87	17.40	7.00	1.31	0.04	ENST00000556347.1
RPL27A	679.93	539.04	73.20	24.53	1.58	0.00	ENST00000530913.1
RPL3	675.15	782.80	40.57	19.89	1.03	0.01	ENST00000216146.6
RPS3	679.46	586.86	92.15	33.65	1.45	0.00	ENST00000528847.1
S100A10	102	79.50	14.35	4.73	1.60	0.03	ENST00000368809.1
S100A13	170.20	160.20	26.96	10.73	1.33	0.01	ENST00000392622.3
S100A4	196.67	120	30.11	7.77	1.95	0.00	ENST00000354332.6
S100A6	763.17	463.25	99.45	25.52	1.96	0.00	ENST00000368719.6
SAA1	53.56	2.45	6.88	0.13	5.69	0.01	ENST00000405158.2
SAA2	28.10	3.12	3.54	0.17	4.41	0.04	ENST00000526900.1
SCARA5	369.92	260.83	10.93	3.26	1.75	0.03	ENST00000380385.4
SDPR	542	248	14.55	2.81	2.37	0.00	ENST00000304141.4
SEPP1	259.38	258.17	28.60	12.03	1.25	0.01	ENST00000507920.3
SERINC1	2465.02	2809.13	68.10	32.81	1.05	0.00	ENST00000339697.4
SFRP4	455.50	222.50	13.31	2.75	2.28	0.01	ENST00000436072.4
SFRP5	88	19	4.15	0.38	3.45	0.04	ENST00000266066.3
SH3BGRL	236	181	10.64	3.45	1.62	0.03	ENST00000373212.5
SH3RF2	591.61	367.27	9.14	2.40	1.93	0.03	ENST00000511217.1
SHOX	1323.03	1067.74	84.87	28.95	1.55	0.00	ENST00000381575.3
SLC52A1	226.73	77.54	6.57	0.95	2.79	0.03	ENST00000512825.4
SLPI	61	33	8.87	2.03	2.13	0.03	ENST00000338380.2
SMIM20	105.33	95.00	11.79	4.50	1.39	0.04	ENST00000514384.1
SOD3	122	47.50	6.93	1.14	2.60	0.03	ENST00000382120.3
SPARC	131.60	139.57	22.32	10.01	1.16	0.03	ENST00000539687.3
SPARCL1	1075.34	1124.30	32.07	14.17	1.18	0.01	ENST00000282470.8
SPINK9	136	156	19.51	9.46	1.04	0.04	ENST00000511717.4
SPTBN1	2008.58	2249.33	20.52	9.72	1.08	0.04	ENST00000356805.6
SSB	361.19	173.48	46.44	9.43	2.30	0.00	ENST00000413002.3
STAG2	3600.48	3092.22	149.36	54.23	1.46	0.00	ENST00000455404.3
TBC1D8B	1488.42	1467.64	31.48	13.12	1.26	0.01	ENST00000481617.4
TBCC	285.13	251.29	15.29	5.70	1.42	0.04	ENST00000372876.1
TIMP3	3508	2684	66.04	21.36	1.63	0.00	ENST00000266085.6
TMEM107	852.53	167.50	61.06	5.07	3.59	0.00	ENST00000449985.4
TMEM14B	209.06	63.80	24.09	3.11	2.95	0.00	ENST00000473166.3

TMEM14E	35	12	8.02	1.16	2.79	0.02	ENST00000408960.4
TMEM56	841.97	618.30	10.70	3.32	1.69	0.03	ENST00000370203.6
TMSB4X	679.66	617.22	93.79	36.01	1.38	0.00	ENST00000451311.4
TMSB4Y	22.43	11.86	14.40	3.22	2.16	0.01	ENST00000622974.1
TNS1	2892.66	3315.06	24.26	11.76	1.05	0.04	ENST00000171887.6
TNXB	1116.66	875.38	15.91	5.27	1.59	0.01	ENST00000611016.1
TOB1	720	427.50	27.99	7.03	1.99	0.00	ENST00000499247.2
TOB2	1141.50	1151	21.69	9.25	1.23	0.02	ENST00000327492.3
TPM4	462.37	399.41	56.28	20.55	1.45	0.00	ENST00000588032.3
TRIM22	284.59	265.94	48.55	19.18	1.34	0.00	ENST00000450670.3
TRNT1	1576.76	1575.06	365.35	154.29	1.24	0.00	ENST00000397779.2
TSC22D3	117.63	74.86	18.50	4.98	1.89	0.01	ENST00000503515.1
TUBD1	380.01	190.77	19.60	4.16	2.24	0.00	ENST00000394239.5
TXNIP	7975.50	6525	236.21	81.70	1.53	0.00	ENST00000582401.3
UCP3	992.33	1174.83	32.79	16.41	1.00	0.01	ENST00000314032.6
UNC45B	1184.67	1391.67	40.21	19.97	1.01	0.01	ENST00000591048.2
VIM	282.40	207.00	32.50	10.07	1.69	0.00	ENST00000421459.2
VWF	4068.67	4529.33	39.89	18.78	1.09	0.00	ENST00000261405.7
WDPCP	690.68	699.94	16.89	7.24	1.22	0.04	ENST00000409562.5
ZNF302	12418.60	10204.02	364.20	126.51	1.53	0.00	ENST00000505365.2
ZNF354B	1965.80	632.03	60.80	8.26	2.88	0.00	ENST00000322434.5

Supplementary Table S3A.

6 miRNAs screened from microarray for qRT-PCR validation and Target Sequence

miR-name	miRNA Signal		log2 Fold-change (MELAS /Control)	Target Sequence (5' to 3')	Accession
	Control	MELAS			
miR-6089	1121	398	-1.49	GGAGGCCGGGGUGG GGCGGGGCGG	MIMAT0023714
miR-27b-3p	696	309	-1.17	UUCACAGUGGCUAA GUUCUGC	MIMAT0000419
miR-214-3p	1422	1011	-0.49	ACAGCAGGCACAGA CAGGCAGU	MIMAT0000271
miR-150-5p	149	538	1.85	UCUCCCAACCCUUG UACCAGUG	MIMAT0000451
let-7e-5p	391	1484	1.92	UGAGGUAGGAGGUU GUAUAGUU	MIMAT0000066
miR-145-5p	1127	2371	1.07	GUCCAGUUUUCCCA GGAAUCCCU	MIMAT0000437

Supplementary Table S3B.

5 lncRNAs and 6 mRNA screened from high-throughout sequencing for qRT-PCR validation

lncRNA/mRNA	RNA rpkm		log2 Fold-	Transcript type	Ensembl gene id
	Control	MELAS			
LINC01405	483.01	29.15	-4.05	lincRNA	ENSG00000185847.5
SNHG12	2.79	44.19	3.99	antisense	ENSG00000197989.1
RP11-403P17.4	63.52	814.92	3.68	lincRNA	ENSG00000261519.3
CTC-260E6.6	2	20.57	3.36	antisense	ENSG00000267383.4
RP11-357D18.1	17.12	108.69	2.67	Processed transcript	ENSG00000250978.3
PDK4	91.78	23.17	-1.99	mRNA	ENST00000005178.5
CDKN1A	16.61	4.26	-1.96	mRNA	ENST00000373711.3
ATP2A2	110.86	32.47	-1.77	mRNA	ENST00000552636.1
SOD3	6.93	47.50	1.14	mRNA	ENST00000382120.3
DDIT4	3.58	16.51	2.2	mRNA	ENST00000307365.3
MKNK2	11.83	24.09	1.03	mRNA	ENST00000589534.2

Supplementary Table S3C. The results of qRT-PCR validation of the miRNAs, lncRNAs and mRNAs screened from high-throughout results between 20 MELAS patients and 20 Controls

(Expression levels of miRNAs, lncRNAs and mRNAs were normalized to GAPDH and U6 snoRNA, respectively, and calculated utilizing the 2-ΔΔCT method.)

Sample	miR-6089	miR-27b-3p	miR-214-3p	miR-150-5p	let-7e-5p	miR-145-5p	LINC01405	SNHG12	RP11-403P17.4	CTC-260E6.6	RP11-357D18.1	PDK4	CDKN1A	ATP2A2	SOD3	DDIT4	EEF1A1
C1	1.18	1.23	0.58	1.7	1.45	2.64	0.39	1.26	0.85	0.84	0.75	0.38	0.27	0.96	2.1	0.71	1.01
C2	1.09	0.84	0.69	0.56	1.12	0.52	1.16	0.84	0.9	1.62	0.47	1.45	0.32	0.36	1.4	0.48	1.97
C3	0.49	0.71	1.06	1.47	1.2	0.64	0.22	2.17	0.4	1.36	0.44	0.56	2.86	1.96	2	1.27	3.18
C4	1.76	0.55	0.56	0.71	1.44	0.49	1.59	1.39	0.74	2.61	1.58	0.84	0.34	0.6	0.6	0.57	1.04
C5	1.95	1.84	0.87	2.48	0.89	0.47	2.11	2.44	0.73	2.29	1.77	1.87	0.26	1.6	3.6	0.79	2.19
C6	2.93	0.54	0.89	1.36	0.77	0.58	2.86	2.55	0.79	1.84	1.91	3.33	3.15	1.34	0.5	3.44	1.64
C7	1.31	0.76	1.27	0.77	0.86	2.03	2.69	0.7	1.36	2.18	3.41	2.2	1.9	0.52	0.4	1.45	2.85
C8	0.63	0.58	1.49	1.22	1.2	0.65	1.6	0.54	1.44	4.04	0.57	0.46	1.85	1.28	0.5	2.13	1.21
C9	0.39	1.31	1.59	0.65	1	0.76	0.64	0.95	1.01	0.49	2.19	2.01	2.07	1.56	1.3	0.88	0.88
C10	0.39	1.43	1.45	1.37	0.78	2.06	0.29	0.76	2.57	2.37	2.2	1.6	0.66	0.43	0.7	1.47	0.84
C11	0.88	0.65	0.76	2.59	1.6	0.59	0.23	0.76	1.82	1.17	0.31	2.88	1.03	1.71	0.7	0.96	0.43
C12	0.41	1.68	1.6	0.42	0.51	2.23	2.73	1.17	0.44	0.8	2.68	0.47	7.71	0.43	1.4	0.45	0.67
C13	2.37	0.55	1.96	0.56	0.95	2.3	0.94	0.62	0.42	0.61	1.24	0.38	4.4	0.59	0.6	0.69	0.58
C14	0.49	1.66	0.68	1.72	1.59	2.28	0.86	0.32	1.39	1.53	0.32	2.37	0.27	1.62	1.6	1.72	1.47
C15	0.73	1.27	2	0.96	1.37	0.69	3.11	1.58	1.95	0.11	0.69	0.38	0.94	2.8	0.6	0.97	1.99
C16	1.01	0.7	0.79	1.45	1.17	0.93	1.23	1.13	0.69	0.86	1.42	0.56	1.52	1.14	0.4	0.42	0.49
C17	2.74	1.21	1.08	2.24	0.92	1.02	0.13	0.75	2.02	1.69	0.3	0.76	1.19	1.82	0.6	3.01	0.46
C18	1.53	0.91	0.52	0.42	0.83	1.06	1.14	2.19	0.9	0.68	1.43	2.11	0.59	0.56	1.2	0.47	0.39
C19	0.7	1.99	0.56	0.63	0.55	0.76	2.58	0.48	1.23	0.16	2.01	0.45	0.7	0.49	0.6	0.73	0.44
C20	1.42	1.52	1.54	0.41	0.78	1.13	1.74	0.75	1.06	0.32	0.46	1.05	0.46	2.32	1.7	1.69	0.88
M1	0.75	0.27	0.72	2.18	3.09	1.68	0.24	11.26	9.65	6.13	3.51	0.53	0.4	0.7	5.3	5.46	3.73
M2	0.27	0.42	0.7	2.27	2.59	2.62	0.12	9.96	10.65	8.45	5.48	0.75	0.33	0.38	4.9	7.68	1.52
M3	0.67	0.32	0.62	3.52	3.77	1.33	0.3	10.29	3.14	8.41	3.36	0.27	0.53	0.28	5.5	5.95	1.47
M4	0.54	0.29	0.97	2.52	2.43	1.36	0.09	10.55	3.75	5.78	4.12	0.37	0.3	0.52	5.7	4.69	1.89
M5	0.38	0.43	0.63	1.89	3.18	3.23	0.26	6.51	1.65	9.38	5.04	0.36	0.39	0.26	5.2	7.61	3.59
M6	0.76	0.32	0.74	2.82	2.56	0.83	0.12	4.82	5.19	6.26	5.53	0.59	0.24	0.41	5.6	7.78	3.55
M7	0.31	0.45	1.1	3.46	2.64	0.71	0.11	11.54	5.68	9.43	3.38	0.29	0.25	0.61	7	4.62	2.82
M8	0.52	0.44	1.27	2.58	3.51	1.98	0.11	6.51	6.09	9.57	3.78	0.41	0.31	0.3	5.4	6.88	1.78
M9	0.66	0.37	0.85	3.42	3.68	1.98	0.49	10.97	4.63	5.69	3.41	0.21	0.34	0.4	7.5	6.07	3.34
M10	0.34	0.41	0.79	2.58	2.44	3.32	0.17	8.78	3.98	9.04	3.06	0.31	0.47	0.26	4.6	5.11	2.66
M11	0.52	0.35	0.53	2.98	2.79	2.52	0.13	10.14	11.51	7.65	3.87	0.56	0.65	0.24	6.4	7.54	2.64
M12	0.32	0.29	1.47	2.73	3.57	1.67	0.1	9.72	5.99	6.74	5.81	0.61	0.29	0.49	4.2	4.72	1.22
M13	0.33	0.27	1.08	3.29	3.05	1.59	0.12	10.4	8.05	9.15	6.02	0.29	0.26	0.56	6.2	8.06	1.65
M14	0.75	0.38	0.74	1.95	3.4	2.43	0.2	10.13	7.76	5.88	2.91	0.25	0.28	0.32	5.5	5.88	3.18
M15	0.65	0.27	0.48	3.81	2.52	2.88	0.18	7.07	8.56	8.92	3.52	0.4	0.37	0.26	6.4	7.33	3.37
M16	0.51	0.34	0.68	3.45	3.01	1.33	0.16	6.9	4.59	5.86	3.39	0.22	0.58	0.54	7.6	8.03	2.69
M17	0.37	0.29	1.79	3.82	3.77	0.98	0.29	9.97	10.13	5.72	5.43	0.28	0.48	0.45	7	7.57	2.99
M18	0.38	0.24	1.23	2.39	2.65	0.77	0.11	9.13	3.15	6.86	2.45	0.52	0.45	0.43	7.2	4.8	3.67
M19	0.74	0.36	0.68	2.93	3.48	3.17	0.09	7.24	9.22	7.75	4.75	0.24	0.7	0.36	6	7.86	3.31
M20	0.48	0.34	1.37	2.12	3.32	1.78	0.11	9.75	7.81	10.14	3.84	0.31	0.45	0.24	6.2	3.79	2.77

Supplementary Table S3D. The results of qRT-PCR for 6 miRNAs and 5 lncRNAs in muscle between 54 MELAS patients and 49 Controls in validation stage

(Expression levels of miRNAs and lncRNAs were normalized to GAPDH and U6 snoRNA, respectively, and calculated utilizing the 2-ΔΔCT method.)

Sample	miR-6089	miR-27b-3p	miR-214-3p	miR-150-5p	let-7e-5p	miR-145-5p	LINC01405	SNHG12	RP11-403P17.4	CTC-260E6.6	RP11-357D18.1
C1	1.85	0.27	0.94	3.48	0.31	2.97	4.23	6.28	1.19	1.28	1.18
C2	0.95	0.71	1.07	3.84	0.3	0.56	2.17	0.99	0.46	0.14	0.24
C3	0.34	1.56	1.31	4.59	0.74	0.73	0.34	3.1	0.37	0.24	0.21
C4	2.3	0.61	1.15	1.31	0.6	3.32	0.94	3.27	4.41	5.03	0.66
C5	0.67	0.85	1.04	3.61	1.89	1.31	0.61	2.85	3.84	1.72	0.24
C6	1.48	1.8	0.9	3.36	0.36	1.89	2.22	0.6	0.58	0.23	0.18
C7	2.31	0.91	2.45	2.33	0.8	2.73	1.96	0.11	0.22	0.31	0.24
C8	1.32	2.97	1.41	1.75	0.59	1.21	1.19	0.36	0.41	6.15	1.79
C9	1.45	1.03	1.33	1.58	0.27	2.83	0.5	1.73	0.38	1.36	0.14
C10	1.66	0.57	1.84	3.94	0.76	1.43	0.35	0.65	2.69	0.35	0.9
C11	1.05	1.13	1.67	0.41	0.5	1.38	1.04	6.92	0.81	2.75	0.56
C12	1.73	2.28	1.76	0.52	0.42	1.95	0.19	4.47	0.37	2.79	0.53
C13	1.2	1.06	1.71	0.38	0.65	0.27	1.42	4.06	0.67	2.95	2.57
C14	1.09	0.76	1.36	0.38	1.69	2.16	1.89	1.26	0.66	3.78	0.15
C15	1.51	4.29	1.61	1.44	2.93	0.79	2.57	2.13	0.36	6.06	5.24
C16	1.23	1.66	1.45	0.38	0.93	0.91	2.41	0.36	4.92	3.14	5.78
C17	0.79	1.49	1.28	0.41	0.75	0.36	0.29	4.06	0.95	2.13	1
C18	1.45	2.1	1.2	0.2	1.35	1.72	1.09	2.14	0.17	0.17	0.46
C19	0.71	1.18	1.18	0.52	1.71	2.99	3.43	0.74	1.11	2.91	2.13
C20	1.4	0.88	0.67	1.79	0.62	3.66	3.1	7.89	0.74	4.03	0.14
C21	0.29	0.4	0.66	4	1.23	1.78	1.43	0.28	7.36	4.11	0.69
C22	0.62	1.99	1.39	2.66	1.67	1.01	0.57	3.89	0.91	0.2	4.26
C23	1.56	1.91	1.15	1.02	1.26	1.56	0.26	0.39	2.33	0.98	5.28
C24	3.92	1.56	1.16	3.12	0.68	0.48	0.21	0.39	1.65	1.93	0.18
C25	3.18	0.99	1.18	0.33	0.51	2.14	2.45	0.6	3.2	0.33	4.23
C26	0.7	1.46	0.79	1.56	0.42	3.56	0.85	0.32	0.38	0.25	1.48
C27	0.48	0.81	0.99	0.49	0.52	0.25	0.77	1.3	1.66	10.13	0.39
C28	1.83	0.95	0.42	0.62	0.71	0.48	0.18	0.33	1.09	3.76	2.73
C29	2.39	1.33	0.97	0.09	0.21	1.16	1.66	1.85	4.2	2.46	2.53
C30	0.66	0.92	0.93	0.33	0.43	0.62	0.79	2.1	0.31	1.01	4.96
C31	0.6	2.14	0.93	3.76	2.66	2.08	1.95	0.33	5.17	6.68	6.92
C32	0.66	0.82	0.42	0.43	0.45	0.26	2.79	1.62	1.77	0.04	0.41
C33	1.32	1.21	1.13	0.27	2.04	0.91	1.1	0.58	0.62	0.35	1.71
C34	0.7	1.53	0.85	0.52	0.66	0.6	0.13	0.29	0.46	3.78	2.93
C35	0.51	0.78	0.75	0.57	0.74	0.24	0.12	0.39	3.66	0.7	0.36
C36	2.51	0.9	1.49	0.68	0.78	0.25	1.02	2.25	0.82	0.28	6.87
C37	0.59	1.35	1.1	0.31	3.53	0.7	2.31	0.06	1.12	0.07	3.41
C38	0.79	0.15	1.09	0.47	3.92	1.31	1.56	1.55	0.96	0.13	0.55
C39	0.51	0.84	0.48	0.63	2.38	0.24	4.06	0.02	1.04	2.11	1.24
C40	0.63	1.21	1.36	0.33	1.06	0.35	1.79	1.25	0.11	0.26	1.23
C41	0.35	0.33	2.08	0.17	1.57	1.78	1.82	0.94	0.41	0.29	4.59
C42	0.69	0.8	2.15	3.2	1.61	2.85	1.01	4.14	2.79	1.27	4.2
C43	4.59	1.14	0.92	2.3	2.03	2.58	0.13	10.85	1.67	0.2	0.22
C44	0.91	0.75	0.73	3.29	1.11	0.33	0.81	0.39	0.98	0.5	0.15

C45	0.7	0.84	1.55	1	1.07	2.43	3.43	0.3	3.1	0.51	3.34
C46	0.45	1.35	0.75	2.71	3.1	0.37	0.43	4.59	0.36	0.31	2.25
C47	0.48	0.95	0.54	1.11	3.76	0.55	2.68	4.79	3.73	2.81	0.82
C48	0.66	0.81	0.8	3.29	5.1	0.67	2.31	0.11	0.19	6.23	0.2
C49	0.69	0.86	2.15	1.75	3.34	0.62	0.78	0.31	1.55	4.06	0.99
M1	0.28	0.33	0.71	5.42	5.96	3.63	0.12	4.88	4.9	2.89	2.66
M2	0.45	0.11	0.76	5	5.22	1.56	0.15	9.34	1.76	2.58	2.64
M3	0.58	0.44	1.74	3.07	6.41	3.52	0.15	7.61	5.49	2.61	5.01
M4	0.81	0.36	0.55	4.35	5.37	2.59	0.18	10.21	1.42	6.83	2.48
M5	0.49	0.28	2.73	3.84	5.23	2.44	0.15	8.61	3.97	7.85	1.33
M6	0.67	0.72	0.28	4.12	5	1.35	0.13	9.7	3.55	2.16	4.67
M7	0.4	0.41	0.56	2.65	2.84	0.71	0.06	6.58	4.44	6.65	3.25
M8	0.79	0.57	0.23	4.92	6.14	3.38	0.11	9.38	8.05	6.67	3.11
M9	0.29	0.23	0.4	2.23	5.78	2.73	0.13	3.89	8.76	7.07	1.7
M10	0.74	0.27	0.82	5.53	2.27	1.16	0.25	10.88	9.24	2.26	2.32
M11	0.24	0.08	1.47	3.53	4.05	1.64	0.12	8.75	10.24	6.17	4.79
M12	0.29	0.52	0.87	3.91	1.75	3.42	0.3	8.97	2.71	6.13	2.17
M13	0.48	0.44	3.28	3.11	2.04	2.55	0.06	9.22	3.34	2.23	3.01
M14	0.28	0.67	0.52	1.78	1.11	2.99	0.26	4.36	1.19	8.1	4.55
M15	0.27	0.32	1.28	4.61	2.8	2.75	0.1	3.69	4.78	3.98	4.54
M16	0.22	0.25	0.42	5.19	5.45	3.54	0.08	10.24	5.27	8.02	2.19
M17	0.44	0.29	3.28	1.92	1.86	1.68	0.31	8.01	7.21	5.78	3.19
M18	0.35	0.31	1.59	2.28	3.04	2.62	0.1	5.42	4.26	5.65	2.36
M19	0.23	0.67	0.36	1.97	3.14	1.33	0.49	2.79	5.9	6.81	5.06
M20	0.26	0.53	0.84	2.13	0.87	0.96	0.1	6.03	9.84	7.23	4.62
M21	0.77	0.43	2.12	3.01	5.06	3.23	0.12	5.32	5.68	8.03	2.69
M22	0.21	0.41	0.38	3.73	4.42	0.83	0.47	9.63	3.65	3.41	1.82
M23	0.24	0.66	0.36	3.35	4.3	0.71	0.15	7.51	3.57	6.77	1.87
M24	0.34	0.46	0.5	2.06	5.18	1.78	0.11	9.79	11.1	5.22	2.61
M25	0.27	0.51	0.91	1.53	1	3.44	0.07	8.42	4.58	4.1	4.62
M26	0.36	0.53	3.52	1.77	5.42	2.59	0.06	11.09	6.64	7.87	5.01
M27	0.56	0.25	0.53	3.08	6.25	2.87	0.22	8.82	7.35	2.5	1.42
M28	0.62	0.23	1.32	5.65	1.35	2.47	0.65	9.7	2.03	4.51	2.41
M29	0.76	0.13	1.42	2.61	2.19	0.76	0.94	10.77	7.3	7.32	3.49
M30	0.31	0.37	0.74	2.75	3.04	0.6	0.47	8.77	7.03	8.09	1.19
M31	0.32	0.34	0.65	2.45	1.61	0.95	0.18	2.93	2.91	5.3	2.08
M32	0.4	0.11	1.13	4.85	6.3	1.76	0.18	5.83	8.15	6.62	2.32
M33	0.69	0.2	0.41	1.63	1.49	0.74	0.11	3.77	3.18	2.3	2.2
M34	0.34	0.36	0.58	4.75	5.26	1.84	0.62	9.62	8.52	6.68	5
M35	0.15	0.37	0.49	5.11	5.73	1.98	0.34	8.66	9.72	2.24	4.54
M36	0.62	0.43	0.41	3.8	5.88	3.32	0.06	9.78	1.98	4.59	1.26
M37	0.65	0.39	0.67	1.66	2.45	2.52	0.09	6.05	8.81	4.22	4.11
M38	0.74	0.49	1.22	4.77	6.74	0.64	0.11	8.45	7.4	7.76	2.63
M39	0.92	0.32	1.3	5.43	3.65	1.59	0.1	4.92	5.4	5.68	3.48
M40	0.75	0.41	0.78	2.35	3.84	2.46	0.53	2.72	3.77	5.72	2.2
M41	0.64	0.13	0.71	4.05	1.45	2.89	0.08	10.99	3.28	5.37	2.38
M42	0.93	0.22	0.44	3.36	1.48	1.13	0.19	11.55	1.93	7.92	3.99
M43	0.37	0.47	1.42	5.1	2.79	2.11	0.12	4.03	8.95	7.91	3.15
M44	0.14	0.18	0.34	2.97	4.22	1.48	0.11	8.33	6.36	5.1	3.16
M45	0.74	0.16	0.41	2.54	2.12	1.77	0.07	10.5	6.02	7.88	3.66
M46	0.6	0.21	0.59	4.03	0.91	0.98	0.35	2.58	9.78	7.93	5.15

M47	0.66	0.13	0.38	1.91	3.68	0.77	0.07	6.83	10.26	2.19	1.88
M48	1.32	0.31	0.97	5.34	4.69	3.57	0.27	7.23	5.28	3.17	4.83
M49	0.64	0.19	0.16	2.82	6.25	1.78	0.13	1.87	5.07	6.18	4.25
M50	0.68	0.93	0.56	2.39	2.08	2.86	0.27	3.3	5.67	7.69	4.48
M51	0.4	0.51	0.56	2.07	1.04	1.16	0.19	9.94	8.79	5.49	2.28
M52	0.24	0.19	0.37	3.77	3.91	3.33	0.13	7.05	5.84	5.43	5.01
M53	0.37	0.4	0.91	5.66	3.05	3.51	0.25	8.73	2.02	3.73	2.46
M54	0.48	0.18	0.55	2.35	2.03	1.24	0.27	11.16	6.57	6.77	1.31

Supplementary Table S4. Target pridicitions of miRNA-mRNA, lncRNA-mRNA and miRNA-lncRNA with negative expression

**Potential target pairs of miRNA-mRNA**				**Potential target pairs of miRNA-lncRNA**				**Potential target pairs of lncRNA-mRNA**							
**miRNA**		**target mRNA**		**miRNA**		**target lncRNA**		**lncRNA**		**target mRNA**		**lncRNA**		**target mRNA**	
hsa-let-7a-5p	up	ACTA1	down	hsa-miR-378c	down	AC007228.9	up	AC007228.9	up	ACTA1	down	RNU12	up	ANKRD1	down
hsa-let-7b-5p	up	ACTA1	down	hsa-miR-378e	down	AC007228.9	up	AC007228.9	up	ACTA1	down	RNU12	up	ANKRD2	down
hsa-let-7c-5p	up	ACTA1	down	hsa-miR-378f	down	AC007228.9	up	AC007228.9	up	ANKRD1	down	RNU12	up	ANKRD2	down
hsa-miR-1260b	up	ADAMTS1	down	hsa-miR-378g	down	AC007228.9	up	AC007228.9	up	ANKRD2	down	RNU12	up	ANKRD2	down
hsa-miR-4459	up	ADAMTS4	down	hsa-miR-378i	down	AC007228.9	up	AC007228.9	up	BHLHE40	down	RNU12	up	ANKRD2	down
hsa-miR-486-3p	up	ADAMTS4	down	hsa-miR-3960	down	AC009501.4	up	AC007228.9	up	CASQ2	down	RNU12	up	BHLHE40	down
hsa-let-7c-5p	up	ADRB2	down	hsa-miR-378g	down	AC007228.9	up	AC007228.9	up	CCL2	down	RNU12	up	C10orf10	down
hsa-miR-214-3p	down	APOD	up	hsa-miR-6087	down	AC007228.9	up	AC007228.9	up	CCL8	down	RNU12	up	CASQ2	down
hsa-miR-3196	down	APOE	up	hsa-miR-7704	down	AC007228.9	up	AC007228.9	up	CDKN1A	down	RNU12	up	CDKN1A	down
hsa-miR-7704	down	APOE	up	hsa-miR-4516	down	AC064871.3	up	AC007228.9	up	CKMT2	down	RNU12	up	CDKN1A	down
hsa-miR-150-5p	up	ARRDC4	down	hsa-miR-378f	down	AC073254.1	up	AC007228.9	up	CKMT2	down	RNU12	up	CDKN1A	down
hsa-miR-15b-5p	down	ASTN2	up	hsa-miR-6089	down	AC073254.1	up	AC007228.9	up	CKMT2	down	RNU12	up	CDKN1A	down
hsa-miR-16-5p	down	ASTN2	up	hsa-miR-378g	down	AC079586.1	up	AC007228.9	up	CXCL10	down	RNU12	up	CDKN1A	down
hsa-let-7a-5p	up	ATP2A2	down	hsa-miR-6727-5p	down	AC079586.1	up	AC007228.9	up	GADD45G	down	RNU12	up	CKMT2	down
hsa-let-7b-5p	up	ATP2A2	down	hsa-miR-3960	down	AC123886.2	up	AC007228.9	up	HMOX1	down	RNU12	up	CKMT2	down
hsa-let-7c-5p	up	ATP2A2	down	hsa-miR-27b-3p	down	AC123886.2	up	AC007228.9	up	HMOX1	down	RNU12	up	CKMT2	down
hsa-let-7d-5p	up	ATP2A2	down	hsa-miR-3196	down	AC123886.2	up	AC007228.9	up	MYL2	down	RNU12	up	CKMT2	down
hsa-let-7e-5p	up	ATP2A2	down	hsa-miR-7704	down	AC123886.2	up	AC007228.9	up	MYL2	down	RNU12	up	CRYAB	down
hsa-let-7f-5p	up	ATP2A2	down	hsa-miR-195-5p	down	BCYRN1	up	AC007228.9	up	MYL6B	down	RNU12	up	CSRP3	down
hsa-let-7g-5p	up	ATP2A2	down	hsa-miR-15b-5p	down	BCYRN1	up	AC007228.9	up	MYL6B	down	RNU12	up	CSRP3	down
hsa-let-7i-5p	up	ATP2A2	down	hsa-miR-16-5p	down	BCYRN1	up	AC007228.9	up	MYL6B	down	RNU12	up	CTSL	down
hsa-miR-125a-5p	up	ATP2A2	down	hsa-miR-24-3p	down	BCYRN1	up	AC007228.9	up	MYOM3	down	RNU12	up	CXCL10	down
hsa-miR-6089	down	ATP9B	up	hsa-miR-3196	down	BCYRN1	up	AC007228.9	up	NNMT	down	RNU12	up	GADD45G	down
hsa-miR-7847-3p	up	BCL3	down	hsa-miR-378g	down	BCYRN1	up	AC007228.9	up	NNMT	down	RNU12	up	GADD45G	down
hsa-miR-486-3p	up	BHLHE40	down	hsa-miR-4443	down	BCYRN1	up	AC007228.9	up	NNMT	down	RNU12	up	MYL2	down
hsa-miR-4459	up	BTG2	down	hsa-miR-5096	down	BCYRN1	up	AC007228.9	up	NNMT	down	RNU12	up	MYL2	down
hsa-miR-7847-3p	up	BTG2	down	hsa-miR-6089	down	BCYRN1	up	AC007228.9	up	PDLIM1	down	RNU12	up	MYL6B	down
hsa-miR-92a-3p	up	BTG2	down	hsa-miR-638	down	BCYRN1	up	AC007228.9	up	RRAD	down	RNU12	up	MYL6B	down
hsa-miR-6087	down	C1orf229	up	hsa-miR-7977	down	BCYRN1	up	AC007228.9	up	SAT1	down	RNU12	up	MYL6B	down
hsa-miR-6089	down	C1orf229	up	hsa-miR-378g	down	CTC-260E6.6	up	AC007228.9	up	SERPINE1	down	RNU12	up	MYOM3	down
hsa-miR-214-3p	down	C1QA	up	hsa-miR-4734	down	CTC-297N7.7	up	AC007228.9	up	SOCS3	down	RNU12	up	NNMT	down
hsa-miR-6087	down	C7	up	hsa-miR-5100	down	CTC-297N7.7	up	AC007228.9	up	SRGN	down	RNU12	up	NNMT	down
hsa-miR-4516	down	CALM1	up	hsa-miR-7977	down	CTC-297N7.7	up	AC007228.9	up	TAP1	down	RNU12	up	NNMT	down
hsa-miR-214-3p	down	CCDC3	up	hsa-miR-27b-3p	down	CTC-297N7.7	up	AC007228.9	up	THBS1	down	RNU12	up	NNMT	down
hsa-miR-378c	down	CCDC3	up	hsa-miR-4734	down	CTC-297N7.7	up	AC007228.9	up	THBS1	down	RNU12	up	NNMT	down
hsa-miR-378e	down	CCDC3	up	hsa-miR-5100	down	CTC-297N7.7	up	AC007228.9	up	TNFRSF12A	down	RNU12	up	RRAD	down
hsa-miR-378f	down	CCDC3	up	hsa-miR-7977	down	CTC-297N7.7	up	AC007228.9	up	TNFRSF12A	down	RNU12	up	RRAD	down
hsa-miR-378i	down	CCDC3	up	hsa-miR-4734	down	CTC-297N7.7	up	AC007228.9	up	TNFRSF12A	down	RNU12	up	SAT1	down
hsa-miR-6089	down	CCDC3	up	hsa-miR-7977	down	CTC-297N7.7	up	AC007228.9	up	TNFRSF12A	down	RNU12	up	SAT1	down
hsa-miR-26a-5p	down	CCPG1	up	hsa-miR-10b-5p	down	CTC-297N7.7	up	AC007228.9	up	TNFRSF12A	down	RNU12	up	SAT1	down
hsa-miR-26b-5p	down	CCPG1	up	hsa-miR-22-3p	down	CTC-297N7.7	up	AC007228.9	up	TNNC1	down	RNU12	up	SERPINE1	down
hsa-let-7e-5p	up	CDKN1A	down	hsa-miR-3665	down	CTC-297N7.7	up	AC007228.9	up	TNNC1	down	RNU12	up	SOCS3	down
hsa-let-7i-5p	up	CDKN1A	down	hsa-miR-5100	down	CTC-297N7.7	up	AC007228.9	up	TNNI1	down	RNU12	up	THBS1	down

hsa-miR-4459	up	CDKN1A	down	hsa-miR-7847-3p	up	CTD-2033D15.	down	AC007228.9	up	TNNI1	down	RNU12	up	THBS1	down
hsa-miR-378a-3p	up	CISH	down	hsa-miR-4459	up	LINC01405	down	AC007228.9	up	TNNI1	down	RNU12	up	TNFRSF12A	down
hsa-miR-7847-3p	up	CISH	down	hsa-miR-10b-5p	down	MYHAS	up	AC007228.9	up	TNNI1	down	RNU12	up	TNFRSF12A	down
hsa-miR-214-3p	down	CNOT2	up	hsa-miR-214-3p	down	MYHAS	up	AC007228.9	up	TNNI1	down	RNU12	up	TNFRSF12A	down
hsa-miR-22-3p	down	CRTAP	up	hsa-miR-22-3p	down	MYHAS	up	AC007228.9	up	TNNI1	down	RNU12	up	TNNC1	down
hsa-miR-3665	down	CRTAP	up	hsa-miR-3665	down	MYHAS	up	AC007228.9	up	TPM3	down	RNU12	up	TNNC1	down
hsa-miR-4267	down	CRTAP	up	hsa-miR-378c	down	MYHAS	up	AC007228.9	up	TPM3	down	RP11-357D18.1	up	BHLHE40	down
hsa-miR-4516	down	CRTAP	up	hsa-miR-378e	down	MYHAS	up	AC009501.4	up	ANKRD1	down	RP11-357D18.1	up	CASQ2	down
hsa-miR-6087	down	CRTAP	up	hsa-miR-378f	down	MYHAS	up	AC009501.4	up	ANKRD2	down	RP11-357D18.1	up	GADD45G	down
hsa-miR-6089	down	CRTAP	up	hsa-miR-378i	down	MYHAS	up	AC009501.4	up	BHLHE40	down	RP11-357D18.1	up	GADD45G	down
hsa-miR-638	down	CRTAP	up	hsa-miR-4734	down	MYHAS	up	AC009501.4	up	C10orf10	down	RP11-357D18.1	up	NNMT	down
hsa-miR-125a-5p	up	CSRNP1	down	hsa-miR-5100	down	MYHAS	up	AC009501.4	up	CASQ2	down	RP11-357D18.1	up	SOCS3	down
hsa-miR-486-3p	up	CSRNP1	down	hsa-miR-6087	down	MYHAS	up	AC009501.4	up	CCL2	down	RP11-357D18.1	up	SRGN	down
hsa-miR-7847-3p	up	CSRNP1	down	hsa-miR-7704	down	MYHAS	up	AC009501.4	up	CKMT2	down	RP11-357D18.1	up	TNFRSF12A	down
hsa-miR-214-3p	down	CYBRD1	up	hsa-miR-7977	down	MYHAS	up	AC009501.4	up	CKMT2	down	RP11-357D18.1	up	TNFRSF12A	down
hsa-miR-5100	down	CYBRD1	up	hsa-miR-4443	down	RNU12	up	AC009501.4	up	CKMT2	down	RP11-357D18.1	up	TNFRSF12A	down
hsa-miR-214-3p	down	DBNDD1	up	hsa-miR-6089	down	RNU12	up	AC009501.4	up	CKMT2	down	RP11-357D18.1	up	TNFRSF12A	down
hsa-miR-24-3p	down	DBNDD1	up	hsa-miR-1-3p	down	RP11-357D18.	up	AC009501.4	up	CTSL	down	RP11-357D18.1	up	TNFRSF12A	down
hsa-miR-378g	down	DBNDD1	up	hsa-miR-1-3p	down	RP11-357D18.	up	AC009501.4	up	CTSL	down	RP11-357D18.1	up	ACTA1	down
hsa-miR-4508	down	DBNDD1	up	hsa-miR-22-3p	down	RP11-403P17.4	up	AC009501.4	up	CXCL10	down	RP11-357D18.1	up	ANKRD2	down
hsa-miR-6727-5p	down	DBNDD1	up	hsa-miR-4443	down	RP11-403P17.4	up	AC009501.4	up	GADD45G	down	RP11-357D18.1	up	ANKRD2	down
hsa-miR-3196	down	DDIT4	up	hsa-miR-4459	up	RP11-451G4.2	down	AC009501.4	up	GADD45G	down	RP11-357D18.1	up	ANKRD2	down
hsa-miR-3665	down	DDIT4L	up	hsa-miR-29a-3p	up	RP11-480I12.7	down	AC009501.4	up	HMOX1	down	RP11-357D18.1	up	ANKRD2	down
hsa-miR-24-3p	down	DDN	up	hsa-miR-4459	up	RP11-480I12.7	down	AC009501.4	up	HMOX1	down	RP11-357D18.1	up	BHLHE40	down
hsa-miR-4267	down	DDN	up	hsa-miR-4516	down	RP11-521L9.1	up	AC009501.4	up	MYL2	down	RP11-357D18.1	up	C10orf10	down
hsa-miR-150-5p	up	DGKD	down	hsa-miR-6089	down	RP11-521L9.1	up	AC009501.4	up	MYL2	down	RP11-357D18.1	up	CCL8	down
hsa-miR-3197	up	DGKD	down	hsa-miR-150-5p	up	RP11-705C15.5	down	AC009501.4	up	MYL6B	down	RP11-357D18.1	up	CDKN1A	down
hsa-miR-4459	up	DGKD	down	hsa-miR-3665	down	RP11-731F5.2	up	AC009501.4	up	MYL6B	down	RP11-357D18.1	up	CDKN1A	down
hsa-miR-486-3p	up	DGKD	down	hsa-miR-378g	down	RP11-731F5.2	up	AC009501.4	up	MYOM3	down	RP11-357D18.1	up	CDKN1A	down
hsa-miR-7847-3p	up	DGKD	down	hsa-miR-486-5p	up	RP11-91P24.7	down	AC009501.4	up	NNMT	down	RP11-357D18.1	up	CDKN1A	down
hsa-miR-1260b	up	DHCR24	down	hsa-miR-92a-3p	up	RP11-91P24.7	down	AC009501.4	up	NNMT	down	RP11-357D18.1	up	CDKN1A	down
hsa-miR-378a-3p	up	DHCR24	down	hsa-miR-92b-3p	up	RP11-91P24.7	down	AC009501.4	up	NNMT	down	RP11-357D18.1	up	CSRP3	down
hsa-miR-4459	up	DHCR24	down	hsa-miR-5100	down	SNHG12	up	AC009501.4	up	NNMT	down	RP11-357D18.1	up	CSRP3	down
hsa-miR-24-3p	down	DNMT3A	up	hsa-miR-6089	down	SNHG12	up	AC009501.4	up	NNMT	down	RP11-357D18.1	up	CTSL	down
hsa-miR-486-3p	up	DUSP2	down	hsa-miR-378g	down	SNHG12	up	AC009501.4	up	PDLIM1	down	RP11-357D18.1	up	GADD45G	down
hsa-miR-27b-3p	down	EEF1A1	up	hsa-miR-5100	down	SNHG12	up	AC009501.4	up	RRAD	down	RP11-357D18.1	up	MYL2	down
hsa-miR-6089	down	EEF1A1	up	hsa-miR-6089	down	SNHG12	up	AC009501.4	up	RRAD	down	RP11-357D18.1	up	MYL2	down
hsa-miR-150-5p	up	EGR1	down	hsa-miR-7977	down	SNHG12	up	AC009501.4	up	SAT1	down	RP11-357D18.1	up	MYOM3	down
hsa-miR-4508	down	ELOF1	up	hsa-miR-378g	down	SNHG12	up	AC009501.4	up	SAT1	down	RP11-357D18.1	up	NNMT	down
hsa-miR-4516	down	ELOF1	up	hsa-miR-5100	down	SNHG12	up	AC009501.4	up	SAT1	down	RP11-357D18.1	up	NNMT	down
hsa-miR-16-5p	down	ERAP1	up	hsa-miR-6089	down	SNHG12	up	AC009501.4	up	SERPINE1	down	RP11-357D18.1	up	NNMT	down
hsa-miR-4267	down	ERAP1	up	hsa-miR-7977	down	SNHG12	up	AC009501.4	up	SOCS3	down	RP11-357D18.1	up	NNMT	down
hsa-miR-5100	down	ERAP1	up	hsa-miR-7977	down	SNHG12	up	AC009501.4	up	SRGN	down	RP11-357D18.1	up	NNMT	down
hsa-miR-3665	down	F13A1	up	hsa-miR-5100	down	SNHG12	up	AC009501.4	up	TAP1	down	RP11-357D18.1	up	RRAD	down
hsa-miR-6089	down	F13A1	up	hsa-miR-6089	down	SNHG12	up	AC009501.4	up	THBS1	down	RP11-357D18.1	up	RRAD	down
hsa-miR-4324	up	FAM46C	down	hsa-miR-7977	down	SNHG12	up	AC009501.4	up	TNFRSF12A	down	RP11-357D18.1	up	SERPINE1	down
hsa-miR-378g	down	FBLN1	up	hsa-miR-24-3p	down	SNHG8	up	AC009501.4	up	TNFRSF12A	down	RP11-357D18.1	up	SOCS3	down
hsa-miR-4443	down	FBLN1	up	hsa-miR-4443	down	SNHG9	up	AC009501.4	up	TNFRSF12A	down	RP11-357D18.1	up	SRGN	down
hsa-miR-7977	down	FBLN1	up	hsa-miR-195-5p	down	SNORA76C	up	AC009501.4	up	TNFRSF12A	down	RP11-357D18.1	up	TAP1	down
hsa-miR-638	down	FBN1	up	hsa-miR-15b-5p	down	SNORA76C	up	AC009501.4	up	TNNC1	down	RP11-357D18.1	up	TNFRSF12A	down
hsa-miR-4516	down	FMO2	up	hsa-miR-16-5p	down	SNORA76C	up	AC009501.4	up	TNNC1	down	RP11-357D18.1	up	TNFRSF12A	down

hsa-miR-486-3p	up	FOSL2	down
hsa-miR-92a-3p	up	FOSL2	down
hsa-miR-92b-3p	up	FOSL2	down
hsa-miR-3196	down	GAS1	up
hsa-miR-7704	down	GAS1	up
hsa-miR-16-5p	down	GLRX	up
hsa-miR-4516	down	GLRX	up
hsa-miR-3197	up	GPT2	down
hsa-miR-27b-3p	down	GREM1	up
hsa-miR-6727-5p	down	GSG2	up
hsa-let-7a-5p	up	HBEGF	down
hsa-let-7d-5p	up	HBEGF	down
hsa-let-7g-5p	up	HBEGF	down
hsa-let-7i-5p	up	HBEGF	down
hsa-miR-7847-3p	up	HBEGF	down
hsa-miR-214-3p	down	HMG20A	up
hsa-miR-4516	down	HMG20A	up
hsa-miR-128-3p	up	HMGB3	down
hsa-miR-4459	up	HMGB3	down
hsa-miR-22-3p	down	HOMER3	up
hsa-let-7e-5p	up	HSPB7	down
hsa-miR-486-3p	up	HSPB7	down
hsa-miR-133a-5p	up	ICAM1	down
hsa-miR-4459	up	ICAM1	down
hsa-miR-378c	down	IGFBP4	up
hsa-miR-378g	down	IGFBP4	up
hsa-miR-3196	down	IGFBP6	up
hsa-miR-4459	up	IRF1	down
hsa-miR-378g	down	KCTD12	up
hsa-miR-4516	down	KCTD12	up
hsa-let-7e-5p	up	KLF9	down
hsa-miR-30d-5p	up	KLF9	down
hsa-miR-125a-5p	up	LIF	down
hsa-miR-378a-3p	up	LIF	down
hsa-miR-4324	up	LIF	down
hsa-miR-486-3p	up	LIF	down
hsa-miR-4508	down	LRP1	up
hsa-miR-128-3p	up	MB	down
hsa-miR-3197	up	MB	down
hsa-miR-3196	down	MGP	up
hsa-miR-4459	up	MID1IP1	down
hsa-miR-4459	up	MIDN	down
hsa-miR-7847-3p	up	MIDN	down
hsa-miR-26a-5p	down	MKNK2	up
hsa-miR-26b-5p	down	MKNK2	up
hsa-miR-27b-3p	down	MKNK2	up
hsa-miR-4734	down	MKNK2	up
hsa-miR-6089	down	MKNK2	up
hsa-miR-24-3p	down	MMP14	up
hsa-miR-6087	down	MMP14	up
hsa-miR-378c	down	MPZ	up

AC009501.4	up	TNNI1	down	RP11-357D18.1	up	TNFRSF12A	down
AC009501.4	up	TNNI1	down	RP11-357D18.1	up	TNFRSF12A	down
AC009501.4	up	TNNI1	down	RP11-357D18.1	up	TNFRSF12A	down
AC011239.1	up	ACTA1	down	RP11-357D18.1	up	TPM3	down
AC011239.1	up	ACTA1	down	RP11-386J22.3	up	ACTA1	down
AC011239.1	up	ANKRD1	down	RP11-386J22.3	up	ACTA1	down
AC011239.1	up	ANKRD2	down	RP11-386J22.3	up	ANKRD2	down
AC011239.1	up	ANKRD2	down	RP11-386J22.3	up	ANKRD2	down
AC011239.1	up	ANKRD2	down	RP11-386J22.3	up	ANKRD2	down
AC011239.1	up	BHLHE40	down	RP11-386J22.3	up	ANKRD2	down
AC011239.1	up	C10orf10	down	RP11-386J22.3	up	BHLHE40	down
AC011239.1	up	CASQ2	down	RP11-386J22.3	up	C10orf10	down
AC011239.1	up	CCL2	down	RP11-386J22.3	up	CCL8	down
AC011239.1	up	CCL8	down	RP11-386J22.3	up	CDKN1A	down
AC011239.1	up	CDKN1A	down	RP11-386J22.3	up	CKMT2	down
AC011239.1	up	CKMT2	down	RP11-386J22.3	up	CKMT2	down
AC011239.1	up	CKMT2	down	RP11-386J22.3	up	CKMT2	down
AC011239.1	up	CKMT2	down	RP11-386J22.3	up	CKMT2	down
AC011239.1	up	CKMT2	down	RP11-386J22.3	up	HMOX1	down
AC011239.1	up	CRYAB	down	RP11-386J22.3	up	HMOX1	down
AC011239.1	up	CSRP3	down	RP11-386J22.3	up	KLF9	down
AC011239.1	up	CSRP3	down	RP11-386J22.3	up	MYL2	down
AC011239.1	up	CTSL	down	RP11-386J22.3	up	MYL2	down
AC011239.1	up	GADD45G	down	RP11-386J22.3	up	MYOM3	down
AC011239.1	up	GADD45G	down	RP11-386J22.3	up	NNMT	down
AC011239.1	up	HMOX1	down	RP11-386J22.3	up	NNMT	down
AC011239.1	up	HMOX1	down	RP11-386J22.3	up	NNMT	down
AC011239.1	up	MYL6B	down	RP11-386J22.3	up	NNMT	down
AC011239.1	up	MYL6B	down	RP11-386J22.3	up	NNMT	down
AC011239.1	up	MYL6B	down	RP11-386J22.3	up	RRAD	down
AC011239.1	up	MYOM3	down	RP11-386J22.3	up	SAT1	down
AC011239.1	up	NNMT	down	RP11-386J22.3	up	SAT1	down
AC011239.1	up	PDLIM1	down	RP11-386J22.3	up	SAT1	down
AC011239.1	up	RRAD	down	RP11-386J22.3	up	SERPINE1	down
AC011239.1	up	RRAD	down	RP11-386J22.3	up	SOCS3	down
AC011239.1	up	SAT1	down	RP11-386J22.3	up	SRGN	down
AC011239.1	up	SAT1	down	RP11-386J22.3	up	TAP1	down
AC011239.1	up	SAT1	down	RP11-386J22.3	up	THBS1	down
AC011239.1	up	SERPINE1	down	RP11-386J22.3	up	THBS1	down
AC011239.1	up	SOCS3	down	RP11-386J22.3	up	TNFRSF12A	down
AC011239.1	up	SRGN	down	RP11-386J22.3	up	TNFRSF12A	down
AC011239.1	up	TAP1	down	RP11-386J22.3	up	TNFRSF12A	down
AC011239.1	up	THBS1	down	RP11-386J22.3	up	TNNC1	down
AC011239.1	up	THBS1	down	RP11-386J22.3	up	TNNI1	down
AC011239.1	up	TNFRSF12A	down	RP11-386J22.3	up	TNNI1	down
AC011239.1	up	TNFRSF12A	down	RP11-386J22.3	up	TNNI1	down
AC011239.1	up	TNFRSF12A	down	RP11-386J22.3	up	TNNI1	down
AC011239.1	up	TNFRSF12A	down	RP11-386J22.3	up	TNNI1	down
AC011239.1	up	TNNC1	down	RP11-386J22.3	up	TPM3	down
AC011239.1	up	TNNC1	down	RP11-386J22.3	up	TPM3	down
AC011239.1	up	TNNI1	down	RP11-403P17.4	up	ACTA1	down

hsa-miR-378f	down	MPZ	up
hsa-miR-150-5p	up	MYH7	down
hsa-miR-486-3p	up	MYL2	down
hsa-miR-378a-3p	up	MYLK3	down
hsa-miR-7847-3p	up	MYLK3	down
hsa-miR-6089	down	MYOG	up
hsa-miR-145-5p	up	PDE4B	down
hsa-miR-133a-5p	up	PDK4	down
hsa-miR-4459	up	PDK4	down
hsa-miR-150-5p	up	PFKFB3	down
hsa-miR-4443	down	PMP22	up
hsa-miR-133a-5p	up	POPDC2	down
hsa-miR-3665	down	PRKCDBP	up
hsa-miR-378g	down	PRKCDBP	up
hsa-miR-4443	down	PRRG3	up
hsa-miR-125a-5p	up	RASSF5	down
hsa-miR-4459	up	RASSF5	down
hsa-let-7b-5p	up	RGS16	down
hsa-let-7g-5p	up	RGS16	down
hsa-miR-378a-3p	up	RHOU	down
hsa-miR-4459	up	RHOU	down
hsa-miR-128-3p	up	RND3	down
hsa-miR-1260b	up	RNF122	down
hsa-miR-3197	up	RNF122	down
hsa-miR-4459	up	RNF122	down
hsa-miR-22-3p	down	RPL27A	up
hsa-miR-4443	down	RPL27A	up
hsa-miR-6087	down	RPL27A	up
hsa-miR-6089	down	RPL27A	up
hsa-miR-125a-5p	up	RRAD	down
hsa-miR-486-3p	up	RRAD	down
hsa-miR-3665	down	SCARA5	up
hsa-miR-1260b	up	SDC4	down
hsa-miR-4459	up	SDC4	down
hsa-miR-10b-5p	down	SDPR	up
hsa-miR-5096	down	SERINC1	up
hsa-miR-4459	up	SERPINA3	down
hsa-miR-145-5p	up	SERPINE1	down
hsa-miR-23c	up	SERPINE1	down
hsa-miR-30c-5p	up	SERPINE1	down
hsa-miR-486-5p	up	SERPINE1	down
hsa-miR-4459	up	SERTAD1	down
hsa-miR-3196	down	SFRP5	up
hsa-miR-378g	down	SFRP5	up
hsa-miR-4516	down	SFRP5	up
hsa-miR-133a-3p	up	SGMS2	down
hsa-miR-133b	up	SGMS2	down
hsa-miR-4267	down	SHOX	up
hsa-miR-4497	down	SHOX	up
hsa-miR-4516	down	SHOX	up
hsa-miR-6087	down	SHOX	up

AC011239.1	up	TNNI1	down	RP11-403P17.4	up	ANKRD1	down
AC011239.1	up	TNNI1	down	RP11-403P17.4	up	ANKRD2	down
AC011239.1	up	TNNI1	down	RP11-403P17.4	up	BHLHE40	down
AC011239.1	up	TNNI1	down	RP11-403P17.4	up	C10orf10	down
AC011239.1	up	TPM3	down	RP11-403P17.4	up	CASQ2	down
AC011239.1	up	TPM3	down	RP11-403P17.4	up	CCL2	down
AC064871.3	up	ANKRD1	down	RP11-403P17.4	up	CCL8	down
AC064871.3	up	ANKRD2	down	RP11-403P17.4	up	CDKN1A	down
AC064871.3	up	ANKRD2	down	RP11-403P17.4	up	CDKN1A	down
AC064871.3	up	ANKRD2	down	RP11-403P17.4	up	CDKN1A	down
AC064871.3	up	ANKRD2	down	RP11-403P17.4	up	CDKN1A	down
AC064871.3	up	C10orf10	down	RP11-403P17.4	up	CKMT2	down
AC064871.3	up	CASQ2	down	RP11-403P17.4	up	CKMT2	down
AC064871.3	up	CDKN1A	down	RP11-403P17.4	up	CKMT2	down
AC064871.3	up	CDKN1A	down	RP11-403P17.4	up	CKMT2	down
AC064871.3	up	CDKN1A	down	RP11-403P17.4	up	CRYAB	down
AC064871.3	up	CDKN1A	down	RP11-403P17.4	up	CTSL	down
AC064871.3	up	CDKN1A	down	RP11-403P17.4	up	CXCL10	down
AC064871.3	up	CKMT2	down	RP11-403P17.4	up	GADD45G	down
AC064871.3	up	CKMT2	down	RP11-403P17.4	up	GADD45G	down
AC064871.3	up	CKMT2	down	RP11-403P17.4	up	HMOX1	down
AC064871.3	up	CTSL	down	RP11-403P17.4	up	HMOX1	down
AC064871.3	up	CTSL	down	RP11-403P17.4	up	MYL2	down
AC064871.3	up	GADD45G	down	RP11-403P17.4	up	MYL2	down
AC064871.3	up	GADD45G	down	RP11-403P17.4	up	MYL6B	down
AC064871.3	up	HMOX1	down	RP11-403P17.4	up	MYL6B	down
AC064871.3	up	MYL2	down	RP11-403P17.4	up	MYOM3	down
AC064871.3	up	MYL2	down	RP11-403P17.4	up	NNMT	down
AC064871.3	up	MYL6B	down	RP11-403P17.4	up	NNMT	down
AC064871.3	up	MYL6B	down	RP11-403P17.4	up	NNMT	down
AC064871.3	up	MYL6B	down	RP11-403P17.4	up	NNMT	down
AC064871.3	up	MYOM3	down	RP11-403P17.4	up	NNMT	down
AC064871.3	up	NNMT	down	RP11-403P17.4	up	PDLIM1	down
AC064871.3	up	NNMT	down	RP11-403P17.4	up	SAT1	down
AC064871.3	up	NNMT	down	RP11-403P17.4	up	SAT1	down
AC064871.3	up	PDLIM1	down	RP11-403P17.4	up	SAT1	down
AC064871.3	up	RRAD	down	RP11-403P17.4	up	SOCS3	down
AC064871.3	up	RRAD	down	RP11-403P17.4	up	SRGN	down
AC064871.3	up	SERPINE1	down	RP11-403P17.4	up	THBS1	down
AC064871.3	up	SOCS3	down	RP11-403P17.4	up	TNNC1	down
AC064871.3	up	TAP1	down	RP11-403P17.4	up	TNNI1	down
AC064871.3	up	TNFRSF12A	down	RP11-403P17.4	up	TNNI1	down
AC064871.3	up	TNFRSF12A	down	RP11-403P17.4	up	TNNI1	down
AC064871.3	up	TNNC1	down	RP11-403P17.4	up	TNNI1	down
AC064871.3	up	TNNC1	down	RP11-403P17.4	up	TNNI1	down
AC064871.3	up	TNNI1	down	RP11-403P17.4	up	TNNI1	down
AC064871.3	up	TNNI1	down	RP11-403P17.4	up	TPM3	down
AC064871.3	up	TNNI1	down	RP11-403P17.4	up	TPM3	down
AC064871.3	up	TNNI1	down	RP11-442H21.2	up	ACTA1	down
AC064871.3	up	TNNI1	down	RP11-442H21.2	up	BHLHE40	down
AC064871.3	up	TNNI1	down	RP11-442H21.2	up	CASQ2	down

hsa-miR-6089	down	SHOX	up
hsa-miR-6125	down	SHOX	up
hsa-miR-23c	up	SLC39A14	down
hsa-miR-3197	up	SOCS3	down
hsa-miR-4459	up	SOCS3	down
hsa-miR-3196	down	SOD3	up
hsa-miR-4734	down	SOD3	up
hsa-miR-378c	down	SPARC	up
hsa-miR-378g	down	SPARC	up
hsa-miR-26a-5p	down	SPTBN1	up
hsa-miR-26b-5p	down	SPTBN1	up
hsa-let-7a-5p	up	SRGN	down
hsa-let-7f-5p	up	SRGN	down
hsa-miR-1-3p	down	TBC1D8B	up
hsa-miR-7977	down	TBCC	up
hsa-miR-4459	up	TGM2	down
hsa-miR-150-5p	up	THBD	down
hsa-let-7a-5p	up	THBS1	down
hsa-let-7b-5p	up	THBS1	down
hsa-let-7c-5p	up	THBS1	down
hsa-let-7f-5p	up	THBS1	down
hsa-let-7i-5p	up	THBS1	down
hsa-miR-206	up	THBS1	down
hsa-miR-378g	down	TIMP3	up
hsa-miR-4516	down	TIMP3	up
hsa-miR-214-3p	down	TMEM56	up
hsa-miR-4516	down	TMEM56	up
hsa-miR-5096	down	TMEM56	up
hsa-miR-6089	down	TMSB4Y	up
hsa-let-7a-5p	up	TNFSF9	down
hsa-let-7b-5p	up	TNFSF9	down
hsa-let-7c-5p	up	TNFSF9	down
hsa-let-7d-5p	up	TNFSF9	down
hsa-let-7e-5p	up	TNFSF9	down
hsa-let-7f-5p	up	TNFSF9	down
hsa-let-7g-5p	up	TNFSF9	down
hsa-miR-4459	up	TNFSF9	down
hsa-miR-7847-3p	up	TNFSF9	down
hsa-miR-4459	up	TNNI1	down
hsa-miR-7847-3p	up	TNNI1	down
hsa-miR-3665	down	TNS1	up
hsa-miR-378g	down	TNS1	up
hsa-miR-4443	down	TNS1	up
hsa-miR-4516	down	TNS1	up
hsa-miR-6727-5p	down	TNXB	up
hsa-miR-378c	down	TOB2	up
hsa-miR-378f	down	TOB2	up
hsa-miR-378g	down	TOB2	up
hsa-miR-378i	down	TOB2	up
hsa-miR-150-5p	up	TPM3	down
hsa-miR-4459	up	TPM3	down

AC064871.3	up	TPM3	down	RP11-442H21.2	up	CCL2	down
AC064871.3	up	TPM3	down	RP11-442H21.2	up	CDKN1A	down
AC073254.1	up	ACTA1	down	RP11-442H21.2	up	CDKN1A	down
AC073254.1	up	ACTA1	down	RP11-442H21.2	up	CDKN1A	down
AC073254.1	up	ANKRD2	down	RP11-442H21.2	up	CDKN1A	down
AC073254.1	up	BHLHE40	down	RP11-442H21.2	up	CDKN1A	down
AC073254.1	up	C10orf10	down	RP11-442H21.2	up	CKMT2	down
AC073254.1	up	CASQ2	down	RP11-442H21.2	up	CKMT2	down
AC073254.1	up	CCL8	down	RP11-442H21.2	up	CKMT2	down
AC073254.1	up	CDKN1A	down	RP11-442H21.2	up	CKMT2	down
AC073254.1	up	CDKN1A	down	RP11-442H21.2	up	CRYAB	down
AC073254.1	up	CDKN1A	down	RP11-442H21.2	up	CSRP3	down
AC073254.1	up	CKMT2	down	RP11-442H21.2	up	CSRP3	down
AC073254.1	up	CRYAB	down	RP11-442H21.2	up	CTSL	down
AC073254.1	up	CSRP3	down	RP11-442H21.2	up	CTSL	down
AC073254.1	up	CTSL	down	RP11-442H21.2	up	CXCL10	down
AC073254.1	up	CTSL	down	RP11-442H21.2	up	GADD45G	down
AC073254.1	up	CTSL	down	RP11-442H21.2	up	GADD45G	down
AC073254.1	up	CXCL10	down	RP11-442H21.2	up	HMOX1	down
AC073254.1	up	GADD45G	down	RP11-442H21.2	up	HMOX1	down
AC073254.1	up	GADD45G	down	RP11-442H21.2	up	MYL2	down
AC073254.1	up	MYL2	down	RP11-442H21.2	up	MYL2	down
AC073254.1	up	MYL2	down	RP11-442H21.2	up	MYL6B	down
AC073254.1	up	MYOM3	down	RP11-442H21.2	up	MYL6B	down
AC073254.1	up	NNMT	down	RP11-442H21.2	up	MYL6B	down
AC073254.1	up	NNMT	down	RP11-442H21.2	up	MYOM3	down
AC073254.1	up	NNMT	down	RP11-442H21.2	up	NNMT	down
AC073254.1	up	NNMT	down	RP11-442H21.2	up	NNMT	down
AC073254.1	up	NNMT	down	RP11-442H21.2	up	NNMT	down
AC073254.1	up	PDLIM1	down	RP11-442H21.2	up	NNMT	down
AC073254.1	up	RRAD	down	RP11-442H21.2	up	NNMT	down
AC073254.1	up	RRAD	down	RP11-442H21.2	up	RRAD	down
AC073254.1	up	SAT1	down	RP11-442H21.2	up	RRAD	down
AC073254.1	up	SAT1	down	RP11-442H21.2	up	SAT1	down
AC073254.1	up	SAT1	down	RP11-442H21.2	up	SAT1	down
AC073254.1	up	SOCS3	down	RP11-442H21.2	up	TAP1	down
AC073254.1	up	SRGN	down	RP11-442H21.2	up	THBS1	down
AC073254.1	up	TAP1	down	RP11-442H21.2	up	THBS1	down
AC073254.1	up	THBS1	down	RP11-442H21.2	up	TNFRSF12A	down
AC073254.1	up	THBS1	down	RP11-442H21.2	up	TNFRSF12A	down
AC073254.1	up	TNFRSF12A	down	RP11-442H21.2	up	TNFRSF12A	down
AC073254.1	up	TNFRSF12A	down	RP11-442H21.2	up	TNFRSF12A	down
AC073254.1	up	TNFRSF12A	down	RP11-442H21.2	up	TNFRSF12A	down
AC073254.1	up	TNNC1	down	RP11-442H21.2	up	TNNC1	down
AC073254.1	up	TNNI1	down	RP11-442H21.2	up	TNNI1	down
AC073254.1	up	TNNI1	down	RP11-442H21.2	up	TNNI1	down
AC073254.1	up	TNNI1	down	RP11-442H21.2	up	TNNI1	down
AC073254.1	up	TNNI1	down	RP11-442H21.2	up	TNNI1	down
AC073254.1	up	TNNI1	down	RP11-442H21.2	up	TNNI1	down
AC073254.1	up	TNNI1	down	RP11-442H21.2	up	TPM3	down
AC073254.1	up	TPM3	down	RP11-451G4.2	down	ADAM10	up

hsa-miR-486-3p	up	TPM3	down					AC073254.1	up	ANKRD1	down	RP11-451G4.2	down	ASB8	up
hsa-miR-4516	down	TPM4	up					AC073254.1	up	ANKRD2	down	RP11-451G4.2	down	ASB8	up
hsa-miR-3197	up	TRIB1	down					AC073254.1	up	ANKRD2	down	RP11-451G4.2	down	BLOC1S6	up
hsa-miR-5100	down	TRNT1	up					AC073254.1	up	ANKRD2	down	RP11-451G4.2	down	C1orf229	up
hsa-miR-4516	down	TSC22D3	up					AC073254.1	up	ANKRD2	down	RP11-451G4.2	down	C1QA	up
hsa-miR-5096	down	TUBD1	up					AC073254.1	up	BHLHE40	down	RP11-451G4.2	down	C1QA	up
hsa-miR-15b-5p	down	TXNIP	up					AC073254.1	up	C10orf10	down	RP11-451G4.2	down	C1QA	up
hsa-miR-4516	down	UNC45B	up					AC073254.1	up	CCL2	down	RP11-451G4.2	down	C3	up
hsa-miR-4459	up	XIRP1	down					AC073254.1	up	CCL8	down	RP11-451G4.2	down	CEBPZOS	up
hsa-miR-486-3p	up	XIRP1	down					AC073254.1	up	CDKN1A	down	RP11-451G4.2	down	CEBPZOS	up
hsa-miR-7847-3p	up	XIRP1	down					AC073254.1	up	CDKN1A	down	RP11-451G4.2	down	CHRNA1	up
hsa-miR-145-5p	up	ZFP36	down					AC073254.1	up	CDKN1A	down	RP11-451G4.2	down	CHRNA1	up
Potential common target mRNAs of the dysregulated miRNAs and lncRNAs				**Potential common target miRNAs of the dysregulated lncRNAs and mRNAs**				AC073254.1	up	CDKN1A	down	RP11-451G4.2	down	CHRNA1	up
**miRNA/lncRNA**		**target mRNA**		**lncRNA / mRNA**		**target miRNA**		AC073254.1	up	CDKN1A	down	RP11-451G4.2	down	CHRNA1	up
AC007228.9	up	ACTA1	down	CTC-297N7.7	up	hsa-miR-10b-5	down	AC073254.1	up	CKMT2	down	RP11-451G4.2	down	COL1A2	up
AC007228.9	up	ACTA1	down	MYHAS	up	hsa-miR-10b-5	down	AC073254.1	up	CKMT2	down	RP11-451G4.2	down	COL1A2	up
AC011239.1	up	ACTA1	down	SDPR	up	hsa-miR-10b-5	down	AC073254.1	up	CKMT2	down	RP11-451G4.2	down	CTSK	up
AC011239.1	up	ACTA1	down	RP11-357D18.1	up	hsa-miR-1-3p	down	AC073254.1	up	CKMT2	down	RP11-451G4.2	down	CTSK	up
AC073254.1	up	ACTA1	down	RP11-357D18.1	up	hsa-miR-1-3p	down	AC073254.1	up	CRYAB	down	RP11-451G4.2	down	DDIT4	up
AC073254.1	up	ACTA1	down	TBC1D8B	up	hsa-miR-1-3p	down	AC073254.1	up	CTSL	down	RP11-451G4.2	down	DNMT3A	up
AC123886.2	up	ACTA1	down	RP11-705C15.5	down	hsa-miR-150-5	up	AC073254.1	up	CTSL	down	RP11-451G4.2	down	EEF1A1	up
AC123886.2	up	ACTA1	down	ARRDC4	down	hsa-miR-150-5	up	AC073254.1	up	CTSL	down	RP11-451G4.2	down	EEF1A1	up
BCYRN1	up	ACTA1	down	DGKD	down	hsa-miR-150-5	up	AC073254.1	up	CXCL10	down	RP11-451G4.2	down	EEF1A1	up
BCYRN1	up	ACTA1	down	EGR1	down	hsa-miR-150-5	up	AC073254.1	up	GADD45G	down	RP11-451G4.2	down	EEF1A1	up
CTC-260E6.6	up	ACTA1	down	MYH7	down	hsa-miR-150-5	up	AC073254.1	up	HMOX1	down	RP11-451G4.2	down	EEF1A1	up
CTC-297N7.7	up	ACTA1	down	PFKFB3	down	hsa-miR-150-5	up	AC073254.1	up	HMOX1	down	RP11-451G4.2	down	EEF1A1	up
CTC-297N7.7	up	ACTA1	down	THBD	down	hsa-miR-150-5	up	AC073254.1	up	MYL2	down	RP11-451G4.2	down	FAM129A	up
CTC-297N7.7	up	ACTA1	down	TPM3	down	hsa-miR-150-5	up	AC073254.1	up	MYL2	down	RP11-451G4.2	down	GREM1	up
CTC-297N7.7	up	ACTA1	down	BCYRN1	up	hsa-miR-15b-5	down	AC073254.1	up	MYL6B	down	RP11-451G4.2	down	GSG2	up
CTC-297N7.7	up	ACTA1	down	SNORA76C	up	hsa-miR-15b-5	down	AC073254.1	up	MYOM3	down	RP11-451G4.2	down	HBA2	up
MYHAS	up	ACTA1	down	ASTN2	up	hsa-miR-15b-5	down	AC073254.1	up	NNMT	down	RP11-451G4.2	down	HBA2	up
MYHAS	up	ACTA1	down	TXNIP	up	hsa-miR-15b-5	down	AC073254.1	up	NNMT	down	RP11-451G4.2	down	HBB	up
RP11-357D18.1	up	ACTA1	down	BCYRN1	up	hsa-miR-16-5p	down	AC073254.1	up	NNMT	down	RP11-451G4.2	down	HIST1H4J	up
RP11-386J22.3	up	ACTA1	down	SNORA76C	up	hsa-miR-16-5p	down	AC073254.1	up	NNMT	down	RP11-451G4.2	down	HIST1H4K	up
RP11-386J22.3	up	ACTA1	down	ASTN2	up	hsa-miR-16-5p	down	AC073254.1	up	NNMT	down	RP11-451G4.2	down	HTRA1	up
RP11-403P17.4	up	ACTA1	down	ERAP1	up	hsa-miR-16-5p	down	AC073254.1	up	PDLIM1	down	RP11-451G4.2	down	HTRA1	up
RP11-442H21.2	up	ACTA1	down	GLRX	up	hsa-miR-16-5p	down	AC073254.1	up	RRAD	down	RP11-451G4.2	down	IGFBP4	up
SNHG1	up	ACTA1	down	MYHAS	up	hsa-miR-214-3	down	AC073254.1	up	RRAD	down	RP11-451G4.2	down	IMPA2	up
SNHG1	up	ACTA1	down	APOD	up	hsa-miR-214-3	down	AC073254.1	up	SAT1	down	RP11-451G4.2	down	KIAA1328	up
SNHG1	up	ACTA1	down	C1QA	up	hsa-miR-214-3	down	AC073254.1	up	SERPINE1	down	RP11-451G4.2	down	LAMC1	up
SNHG1	up	ACTA1	down	CCDC3	up	hsa-miR-214-3	down	AC073254.1	up	SRGN	down	RP11-451G4.2	down	MGP	up
SNHG8	up	ACTA1	down	CNOT2	up	hsa-miR-214-3	down	AC073254.1	up	THBS1	down	RP11-451G4.2	down	MGP	up
SNHG9	up	ACTA1	down	CYBRD1	up	hsa-miR-214-3	down	AC073254.1	up	TNFRSF12A	down	RP11-451G4.2	down	MGP	up
SNHG9	up	ACTA1	down	DBNDD1	up	hsa-miR-214-3	down	AC073254.1	up	TNFRSF12A	down	RP11-451G4.2	down	MKNK2	up
SNORA76C	up	ACTA1	down	HMG20A	up	hsa-miR-214-3	down	AC073254.1	up	TNFRSF12A	down	RP11-451G4.2	down	MKNK2	up
SNORA76C	up	ACTA1	down	TMEM56	up	hsa-miR-214-3	down	AC073254.1	up	TNFRSF12A	down	RP11-451G4.2	down	MKNK2	up
AC007228.9	up	ACTA1	down	RP11-403P17.4	up	hsa-miR-22-3p	down	AC073254.1	up	TNFRSF12A	down	RP11-451G4.2	down	MKNK2	up
**hsa-let-7a-5p**	**up**	**ACTA1**	**down**	CTC-297N7.7	up	hsa-miR-22-3p	down	AC073254.1	up	TNNC1	down	RP11-451G4.2	down	MKNK2	up
**hsa-let-7b-5p**	**up**	**ACTA1**	**down**	MYHAS	up	hsa-miR-22-3p	down	AC073254.1	up	TNNI1	down	RP11-451G4.2	down	MKNK2	up
**hsa-let-7c-5p**	**up**	**ACTA1**	**down**	CRTAP	up	hsa-miR-22-3p	down	AC073254.1	up	TNNI1	down	RP11-451G4.2	down	MKNK2	up

AC007228.9	up	BHLHE40	down	HOMER3	up	hsa-miR-22-3p	dowr	AC073254.1	up	TNNI1	down	RP11-451G4.2	down	MMP14	up
AC009501.4	up	BHLHE40	down	RPL27A	up	hsa-miR-22-3p	dowr	AC073254.1	up	TNNI1	down	RP11-451G4.2	down	MORC4	up
AC011239.1	up	BHLHE40	down	BCYRN1	up	hsa-miR-24-3p	dowr	AC073254.1	up	TNNI1	down	RP11-451G4.2	down	MSS51	up
AC073254.1	up	BHLHE40	down	SNHG8	up	hsa-miR-24-3p	dowr	AC073254.1	up	TPM3	down	RP11-451G4.2	down	MSS51	up
AC073254.1	up	BHLHE40	down	DBNDD1	up	hsa-miR-24-3p	dowr	AC073254.1	up	TPM3	down	RP11-451G4.2	down	MSS51	up
AC079586.1	up	BHLHE40	down	DDN	up	hsa-miR-24-3p	dowr	AC079586.1	up	ANKRD1	down	RP11-451G4.2	down	MYOC	up
AC123886.2	up	BHLHE40	down	DNMT3A	up	hsa-miR-24-3p	dowr	AC079586.1	up	ANKRD2	down	RP11-451G4.2	down	MYOC	up
BCYRN1	up	BHLHE40	down	MMP14	up	hsa-miR-24-3p	dowr	AC079586.1	up	ANKRD2	down	RP11-451G4.2	down	MYOG	up
CTC-260E6.6	up	BHLHE40	down	AC123886.2	up	hsa-miR-27b-3	dowr	AC079586.1	up	ANKRD2	down	RP11-451G4.2	down	PRRG3	up
CTC-297N7.7	up	BHLHE40	down	CTC-297N7.7	up	hsa-miR-27b-3	dowr	AC079586.1	up	ANKRD2	down	RP11-451G4.2	down	RNASE1	up
CTC-297N7.7	up	BHLHE40	down	EEF1A1	up	hsa-miR-27b-3	dowr	AC079586.1	up	BHLHE40	down	RP11-451G4.2	down	RNASE1	up
CTC-297N7.7	up	BHLHE40	down	GREM1	up	hsa-miR-27b-3	dowr	AC079586.1	up	C10orf10	down	RP11-451G4.2	down	RNASE1	up
MYHAS	up	BHLHE40	down	MKNK2	up	hsa-miR-27b-3	dowr	AC079586.1	up	CASQ2	down	RP11-451G4.2	down	RNASE1	up
RNU12	up	BHLHE40	down	BCYRN1	up	hsa-miR-3196	dowr	AC079586.1	up	CCL2	down	RP11-451G4.2	down	RPL27A	up
RP11-357D18.1	up	BHLHE40	down	AC123886.2	up	hsa-miR-3196	dowr	AC079586.1	up	CCL8	down	RP11-451G4.2	down	RPS3	up
RP11-357D18.1	up	BHLHE40	down	APOE	up	hsa-miR-3196	dowr	AC079586.1	up	CDKN1A	down	RP11-451G4.2	down	SPARC	up
RP11-386J22.3	up	BHLHE40	down	DDIT4	up	hsa-miR-3196	dowr	AC079586.1	up	CDKN1A	down	RP11-451G4.2	down	SPARC	up
RP11-403P17.4	up	BHLHE40	down	GAS1	up	hsa-miR-3196	dowr	AC079586.1	up	CDKN1A	down	RP11-451G4.2	down	SPARC	up
RP11-442H21.2	up	BHLHE40	down	IGFBP6	up	hsa-miR-3196	dowr	AC079586.1	up	CKMT2	down	RP11-451G4.2	down	TBC1D8B	up
RP11-731F5.2	up	BHLHE40	down	MGP	up	hsa-miR-3196	dowr	AC079586.1	up	CKMT2	down	RP11-451G4.2	down	TMEM107	up
SNHG1	up	BHLHE40	down	SFRP5	up	hsa-miR-3196	dowr	AC079586.1	up	CKMT2	down	RP11-451G4.2	down	TMSB4X	up
SNHG1	up	BHLHE40	down	SOD3	up	hsa-miR-3196	dowr	AC079586.1	up	CRYAB	down	RP11-451G4.2	down	TMSB4X	up
SNHG12	up	BHLHE40	down	RP11-731F5.2	up	hsa-miR-3665	dowr	AC079586.1	up	CSRP3	down	RP11-451G4.2	down	TPM4	up
SNHG12	up	BHLHE40	down	CTC-297N7.7	up	hsa-miR-3665	dowr	AC079586.1	up	CSRP3	down	RP11-451G4.2	down	TPM4	up
SNHG12	up	BHLHE40	down	MYHAS	up	hsa-miR-3665	dowr	AC079586.1	up	CTSL	down	RP11-451G4.2	down	TRIM22	up
SNHG9	up	BHLHE40	down	CRTAP	up	hsa-miR-3665	dowr	AC079586.1	up	CTSL	down	RP11-451G4.2	down	TRIM22	up
SNORA76C	up	BHLHE40	down	DDIT4L	up	hsa-miR-3665	dowr	AC079586.1	up	HMOX1	down	RP11-451G4.2	down	TRIM22	up
**hsa-miR-486-3p**	**up**	**BHLHE40**	**down**	F13A1	up	hsa-miR-3665	dowr	AC079586.1	up	HMOX1	down	RP11-451G4.2	down	TRIM22	up
CTD-2033D15.1	down	C1orf229	up	PRKCDBP	up	hsa-miR-3665	dowr	AC079586.1	up	MYL2	down	RP11-451G4.2	down	TUBD1	up
CTD-2033D15.1	down	C1orf229	up	SCARA5	up	hsa-miR-3665	dowr	AC079586.1	up	MYL2	down	RP11-451G4.2	down	TUBD1	up
EAF1-AS1	down	C1orf229	up	TNS1	up	hsa-miR-3665	dowr	AC079586.1	up	MYL6B	down	RP11-451G4.2	down	TUBD1	up
LINC01405	down	C1orf229	up	MYHAS	up	hsa-miR-378c	dowr	AC079586.1	up	MYL6B	down	RP11-451G4.2	down	TUBD1	up
LINC01405	down	C1orf229	up	AC007228.9	up	hsa-miR-378c	dowr	AC079586.1	up	MYL6B	down	RP11-451G4.2	down	TUBD1	up
RP11-451G4.2	down	C1orf229	up	CCDC3	up	hsa-miR-378c	dowr	AC079586.1	up	MYOM3	down	RP11-451G4.2	down	UCP3	up
RP11-705C15.5	down	C1orf229	up	IGFBP4	up	hsa-miR-378c	dowr	AC079586.1	up	NNMT	down	RP11-451G4.2	down	UCP3	up
RP11-91P24.7	down	C1orf229	up	MPZ	up	hsa-miR-378c	dowr	AC079586.1	up	NNMT	down	RP11-451G4.2	down	UCP3	up
**hsa-miR-6087**	**down**	**C1orf229**	**up**	SPARC	up	hsa-miR-378c	dowr	AC079586.1	up	NNMT	down	RP11-451G4.2	down	ZNF302	up
**hsa-miR-6089**	**down**	**C1orf229**	**up**	TOB2	up	hsa-miR-378c	dowr	AC079586.1	up	NNMT	down	RP11-451G4.2	down	ZNF302	up
CTD-2033D15.1	down	C1QA	up	MYHAS	up	hsa-miR-378e	dowr	AC079586.1	up	NNMT	down	RP11-480I12.7	down	A2M	up
CTD-2033D15.1	down	C1QA	up	AC007228.9	up	hsa-miR-378e	dowr	AC079586.1	up	PDLIM1	down	RP11-480I12.7	down	A2M	up
CTD-2033D15.1	down	C1QA	up	CCDC3	up	hsa-miR-378e	dowr	AC079586.1	up	RRAD	down	RP11-480I12.7	down	A2M	up
CTD-2033D15.1	down	C1QA	up	AC073254.1	up	hsa-miR-378f	dowr	AC079586.1	up	RRAD	down	RP11-480I12.7	down	ADAM10	up
EAF1-AS1	down	C1QA	up	MYHAS	up	hsa-miR-378f	dowr	AC079586.1	up	SAT1	down	RP11-480I12.7	down	ASB8	up
EAF1-AS1	down	C1QA	up	AC007228.9	up	hsa-miR-378f	dowr	AC079586.1	up	SAT1	down	RP11-480I12.7	down	ASB8	up
EAF1-AS1	down	C1QA	up	CCDC3	up	hsa-miR-378f	dowr	AC079586.1	up	SAT1	down	RP11-480I12.7	down	BLOC1S6	up
LINC01405	down	C1QA	up	MPZ	up	hsa-miR-378f	dowr	AC079586.1	up	SERPINE1	down	RP11-480I12.7	down	C3	up
LINC01405	down	C1QA	up	TOB2	up	hsa-miR-378f	dowr	AC079586.1	up	SRGN	down	RP11-480I12.7	down	C3	up
LINC01405	down	C1QA	up	AC079586.1	up	hsa-miR-378g	dowr	AC079586.1	up	TAP1	down	RP11-480I12.7	down	CEBPZOS	up
LINC01405	down	C1QA	up	BCYRN1	up	hsa-miR-378g	dowr	AC079586.1	up	THBS1	down	RP11-480I12.7	down	CEBPZOS	up
RP11-451G4.2	down	C1QA	up	AC009501.4	up	hsa-miR-378g	dowr	AC079586.1	up	THBS1	down	RP11-480I12.7	down	CHRNA1	up
RP11-451G4.2	down	C1QA	up	RP11-731F5.2	up	hsa-miR-378g	dowr	AC079586.1	up	TNFRSF12A	down	RP11-480I12.7	down	CHRNA1	up

RP11-451G4.2	down	C1QA	up	SNHG12	up	hsa-miR-378g	dowr	AC079586.1	up	TNFRSF12A	down	RP11-480I12.7	down	CHRNA1	up
RP11-603J24.14	down	C1QA	up	SNHG12	up	hsa-miR-378g	dowr	AC079586.1	up	TNFRSF12A	down	RP11-480I12.7	down	CHRNA1	up
RP11-603J24.14	down	C1QA	up	CTC-260E6.6	up	hsa-miR-378g	dowr	AC079586.1	up	TNFRSF12A	down	RP11-480I12.7	down	CHRNA1	up
RP11-603J24.14	down	C1QA	up	AC007228.9	up	hsa-miR-378g	dowr	AC079586.1	up	TNNC1	down	RP11-480I12.7	down	CHRNA1	up
RP11-705C15.5	down	C1QA	up	DBNDD1	up	hsa-miR-378g	dowr	AC079586.1	up	TNNC1	down	RP11-480I12.7	down	COL1A2	up
RP11-705C15.5	down	C1QA	up	FBLN1	up	hsa-miR-378g	dowr	AC079586.1	up	TNNI1	down	RP11-480I12.7	down	COL1A2	up
RP11-705C15.5	down	C1QA	up	IGFBP4	up	hsa-miR-378g	dowr	AC079586.1	up	TNNI1	down	RP11-480I12.7	down	DDIT4	up
RP11-91P24.7	down	C1QA	up	KCTD12	up	hsa-miR-378g	dowr	AC079586.1	up	TNNI1	down	RP11-480I12.7	down	EEF1A1	up
RP11-91P24.7	down	C1QA	up	PRKCDBP	up	hsa-miR-378g	dowr	AC079586.1	up	TPM3	down	RP11-480I12.7	down	EEF1A1	up
RP11-91P24.7	down	C1QA	up	SFRP5	up	hsa-miR-378g	dowr	AC079586.1	up	TPM3	down	RP11-480I12.7	down	EEF1A1	up
**hsa-miR-214-3p**	**down**	**C1Q A**	**up**	SPARC	up	hsa-miR-378g	dowr	AC123886.2	up	ACTA1	down	RP11-480I12.7	down	EEF1A1	up
AC007228.9	up	CDKN1A	down	TIMP3	up	hsa-miR-378g	dowr	AC123886.2	up	ACTA1	down	RP11-480I12.7	down	EEF1A1	up
AC011239.1	up	CDKN1A	down	TNS1	up	hsa-miR-378g	dowr	AC123886.2	up	ANKRD1	down	RP11-480I12.7	down	EEF1A1	up
AC064871.3	up	CDKN1A	down	TOB2	up	hsa-miR-378g	dowr	AC123886.2	up	ANKRD2	down	RP11-480I12.7	down	EEF1A1	up
AC064871.3	up	CDKN1A	down	MYHAS	up	hsa-miR-378i	dowr	AC123886.2	up	ANKRD2	down	RP11-480I12.7	down	EIF4A2	up
AC064871.3	up	CDKN1A	down	AC007228.9	up	hsa-miR-378i	dowr	AC123886.2	up	ANKRD2	down	RP11-480I12.7	down	EIF4A2	up
AC064871.3	up	CDKN1A	down	CCDC3	up	hsa-miR-378i	dowr	AC123886.2	up	ANKRD2	down	RP11-480I12.7	down	EIF4A2	up
AC064871.3	up	CDKN1A	down	TOB2	up	hsa-miR-378i	dowr	AC123886.2	up	BHLHE40	down	RP11-480I12.7	down	EIF4A2	up
AC073254.1	up	CDKN1A	down	BCYRN1	up	hsa-miR-4443	dowr	AC123886.2	up	C10orf10	down	RP11-480I12.7	down	EIF4A2	up
AC073254.1	up	CDKN1A	down	RP11-403P17.4	up	hsa-miR-4443	dowr	AC123886.2	up	CASQ2	down	RP11-480I12.7	down	EIF4A2	up
AC073254.1	up	CDKN1A	down	SNHG9	up	hsa-miR-4443	dowr	AC123886.2	up	CCL2	down	RP11-480I12.7	down	EIF4A2	up
AC073254.1	up	CDKN1A	down	RNU12	up	hsa-miR-4443	dowr	AC123886.2	up	CCL8	down	RP11-480I12.7	down	GREM1	up
AC073254.1	up	CDKN1A	down	FBLN1	up	hsa-miR-4443	dowr	AC123886.2	up	CDKN1A	down	RP11-480I12.7	down	GSG2	up
AC073254.1	up	CDKN1A	down	PMP22	up	hsa-miR-4443	dowr	AC123886.2	up	CDKN1A	down	RP11-480I12.7	down	HBA1	up
AC073254.1	up	CDKN1A	down	PRRG3	up	hsa-miR-4443	dowr	AC123886.2	up	CDKN1A	down	RP11-480I12.7	down	HBA1	up
AC073254.1	up	CDKN1A	down	RPL27A	up	hsa-miR-4443	dowr	AC123886.2	up	CDKN1A	down	RP11-480I12.7	down	HBA2	up
AC079586.1	up	CDKN1A	down	TNS1	up	hsa-miR-4443	dowr	AC123886.2	up	CDKN1A	down	RP11-480I12.7	down	HBA2	up
AC079586.1	up	CDKN1A	down	RP11-480I12.7	down	hsa-miR-4459	up	AC123886.2	up	CKMT2	down	RP11-480I12.7	down	HBB	up
AC079586.1	up	CDKN1A	down	RP11-451G4.2	down	hsa-miR-4459	up	AC123886.2	up	CKMT2	down	RP11-480I12.7	down	HBD	up
AC123886.2	up	CDKN1A	down	LINC01405	down	hsa-miR-4459	up	AC123886.2	up	CKMT2	down	RP11-480I12.7	down	HBD	up
AC123886.2	up	CDKN1A	down	ADAMTS4	down	hsa-miR-4459	up	AC123886.2	up	CKMT2	down	RP11-480I12.7	down	HBD	up
AC123886.2	up	CDKN1A	down	BTG2	down	hsa-miR-4459	up	AC123886.2	up	CSRP3	down	RP11-480I12.7	down	HIST1H4J	up
AC123886.2	up	CDKN1A	down	CDKN1A	down	hsa-miR-4459	up	AC123886.2	up	CSRP3	down	RP11-480I12.7	down	HIST1H4K	up
AC123886.2	up	CDKN1A	down	DGKD	down	hsa-miR-4459	up	AC123886.2	up	CTSL	down	RP11-480I12.7	down	HTRA1	up
BCYRN1	up	CDKN1A	down	DHCR24	down	hsa-miR-4459	up	AC123886.2	up	CTSL	down	RP11-480I12.7	down	HTRA1	up
BCYRN1	up	CDKN1A	down	HMGB3	down	hsa-miR-4459	up	AC123886.2	up	CTSL	down	RP11-480I12.7	down	IGFBP4	up
BCYRN1	up	CDKN1A	down	ICAM1	down	hsa-miR-4459	up	AC123886.2	up	CXCL10	down	RP11-480I12.7	down	IGFBP7	up
BCYRN1	up	CDKN1A	down	IRF1	down	hsa-miR-4459	up	AC123886.2	up	GADD45G	down	RP11-480I12.7	down	IGKC	up
BCYRN1	up	CDKN1A	down	MID1IP1	down	hsa-miR-4459	up	AC123886.2	up	GADD45G	down	RP11-480I12.7	down	IGKC	up
CTC-260E6.6	up	CDKN1A	down	MIDN	down	hsa-miR-4459	up	AC123886.2	up	HMOX1	down	RP11-480I12.7	down	IGKC	up
CTC-260E6.6	up	CDKN1A	down	PDK4	down	hsa-miR-4459	up	AC123886.2	up	HMOX1	down	RP11-480I12.7	down	IGKC	up
CTC-260E6.6	up	CDKN1A	down	RASSF5	down	hsa-miR-4459	up	AC123886.2	up	MYL2	down	RP11-480I12.7	down	IMPA2	up
CTC-260E6.6	up	CDKN1A	down	RHOU	down	hsa-miR-4459	up	AC123886.2	up	MYL2	down	RP11-480I12.7	down	IMPA2	up
CTC-297N7.7	up	CDKN1A	down	RNF122	down	hsa-miR-4459	up	AC123886.2	up	MYL6B	down	RP11-480I12.7	down	KIAA1328	up
CTC-297N7.7	up	CDKN1A	down	SDC4	down	hsa-miR-4459	up	AC123886.2	up	MYL6B	down	RP11-480I12.7	down	LUM	up
CTC-297N7.7	up	CDKN1A	down	SERPINA3	down	hsa-miR-4459	up	AC123886.2	up	MYOM3	down	RP11-480I12.7	down	MGP	up
CTC-297N7.7	up	CDKN1A	down	SERTAD1	down	hsa-miR-4459	up	AC123886.2	up	NNMT	down	RP11-480I12.7	down	MGP	up
CTC-297N7.7	up	CDKN1A	down	SOCS3	down	hsa-miR-4459	up	AC123886.2	up	NNMT	down	RP11-480I12.7	down	MKNK2	up
CTC-297N7.7	up	CDKN1A	down	TGM2	down	hsa-miR-4459	up	AC123886.2	up	NNMT	down	RP11-480I12.7	down	MKNK2	up
CTC-297N7.7	up	CDKN1A	down	TNFSF9	down	hsa-miR-4459	up	AC123886.2	up	NNMT	down	RP11-480I12.7	down	MKNK2	up
CTC-297N7.7	up	CDKN1A	down	TNNI1	down	hsa-miR-4459	up	AC123886.2	up	NNMT	down	RP11-480I12.7	down	MKNK2	up

CTC-297N7.7	up	CDKN1A	down	TPM3	down	hsa-miR-4459	up	AC123886.2	up	PDLIM1	down	RP11-480I12.7	down	MKNK2	up
CTC-297N7.7	up	CDKN1A	down	XIRP1	down	hsa-miR-4459	up	AC123886.2	up	RRAD	down	RP11-480I12.7	down	MKNK2	up
CTC-297N7.7	up	CDKN1A	down	AC064871.3	up	hsa-miR-4516	dowr	AC123886.2	up	SAT1	down	RP11-480I12.7	down	MMP14	up
CTC-297N7.7	up	CDKN1A	down	RP11-521L9.1	up	hsa-miR-4516	dowr	AC123886.2	up	SAT1	down	RP11-480I12.7	down	MORC4	up
CTC-297N7.7	up	CDKN1A	down	CALM1	up	hsa-miR-4516	dowr	AC123886.2	up	SAT1	down	RP11-480I12.7	down	MSS51	up
CTC-297N7.7	up	CDKN1A	down	CRTAP	up	hsa-miR-4516	dowr	AC123886.2	up	SERPINE1	down	RP11-480I12.7	down	MSS51	up
CTC-297N7.7	up	CDKN1A	down	ELOF1	up	hsa-miR-4516	dowr	AC123886.2	up	SRGN	down	RP11-480I12.7	down	MYH1	up
CTC-297N7.7	up	CDKN1A	down	FMO2	up	hsa-miR-4516	dowr	AC123886.2	up	TAP1	down	RP11-480I12.7	down	MYH4	up
CTC-297N7.7	up	CDKN1A	down	GLRX	up	hsa-miR-4516	dowr	AC123886.2	up	THBS1	down	RP11-480I12.7	down	PRRG3	up
CTC-297N7.7	up	CDKN1A	down	HMG20A	up	hsa-miR-4516	dowr	AC123886.2	up	THBS1	down	RP11-480I12.7	down	RNASE1	up
CTC-297N7.7	up	CDKN1A	down	KCTD12	up	hsa-miR-4516	dowr	AC123886.2	up	TNFRSF12A	down	RP11-480I12.7	down	RNASE1	up
MYHAS	up	CDKN1A	down	SFRP5	up	hsa-miR-4516	dowr	AC123886.2	up	TNFRSF12A	down	RP11-480I12.7	down	RNASE1	up
MYHAS	up	CDKN1A	down	SHOX	up	hsa-miR-4516	dowr	AC123886.2	up	TNFRSF12A	down	RP11-480I12.7	down	RNASE1	up
MYHAS	up	CDKN1A	down	TIMP3	up	hsa-miR-4516	dowr	AC123886.2	up	TNFRSF12A	down	RP11-480I12.7	down	RPL27A	up
MYHAS	up	CDKN1A	down	TMEM56	up	hsa-miR-4516	dowr	AC123886.2	up	TNFRSF12A	down	RP11-480I12.7	down	RPS3	up
RNU12	up	CDKN1A	down	TNS1	up	hsa-miR-4516	dowr	AC123886.2	up	TNNC1	down	RP11-480I12.7	down	SPARC	up
RNU12	up	CDKN1A	down	TPM4	up	hsa-miR-4516	dowr	AC123886.2	up	TNNC1	down	RP11-480I12.7	down	SPARC	up
RNU12	up	CDKN1A	down	TSC22D3	up	hsa-miR-4516	dowr	AC123886.2	up	TNNI1	down	RP11-480I12.7	down	SPARC	up
RNU12	up	CDKN1A	down	UNC45B	up	hsa-miR-4516	dowr	AC123886.2	up	TNNI1	down	RP11-480I12.7	down	SPARC	up
RNU12	up	CDKN1A	down	CTC-297N7.7	up	hsa-miR-4734	dowr	AC123886.2	up	TPM3	down	RP11-480I12.7	down	SPARC	up
RP11-357D18.1	up	CDKN1A	down	CTC-297N7.7	up	hsa-miR-4734	dowr	BCYRN1	up	ACTA1	down	RP11-480I12.7	down	STAG2	up
RP11-357D18.1	up	CDKN1A	down	CTC-297N7.7	up	hsa-miR-4734	dowr	BCYRN1	up	ACTA1	down	RP11-480I12.7	down	STAG2	up
RP11-357D18.1	up	CDKN1A	down	MYHAS	up	hsa-miR-4734	dowr	BCYRN1	up	ANKRD1	down	RP11-480I12.7	down	TBC1D8B	up
RP11-357D18.1	up	CDKN1A	down	MKNK2	up	hsa-miR-4734	dowr	BCYRN1	up	ANKRD2	down	RP11-480I12.7	down	TMEM107	up
RP11-357D18.1	up	CDKN1A	down	SOD3	up	hsa-miR-4734	dowr	BCYRN1	up	BHLHE40	down	RP11-480I12.7	down	TMSB4X	up
RP11-386J22.3	up	CDKN1A	down	RP11-91P24.7	down	hsa-miR-486-5	up	BCYRN1	up	CASQ2	down	RP11-480I12.7	down	TMSB4X	up
RP11-403P17.4	up	CDKN1A	down	SERPINE1	down	hsa-miR-486-5	up	BCYRN1	up	CCL2	down	RP11-480I12.7	down	TMSB4X	up
RP11-403P17.4	up	CDKN1A	down	BCYRN1	up	hsa-miR-5096	dowr	BCYRN1	up	CCL8	down	RP11-480I12.7	down	TRIM22	up
RP11-403P17.4	up	CDKN1A	down	SERINC1	up	hsa-miR-5096	dowr	BCYRN1	up	CDKN1A	down	RP11-480I12.7	down	TRIM22	up
RP11-403P17.4	up	CDKN1A	down	TMEM56	up	hsa-miR-5096	dowr	BCYRN1	up	CDKN1A	down	RP11-480I12.7	down	TRIM22	up
RP11-442H21.2	up	CDKN1A	down	TUBD1	up	hsa-miR-5096	dowr	BCYRN1	up	CDKN1A	down	RP11-480I12.7	down	TRIM22	up
RP11-442H21.2	up	CDKN1A	down	SNHG12	up	hsa-miR-5100	dowr	BCYRN1	up	CDKN1A	down	RP11-480I12.7	down	TRIM22	up
RP11-442H21.2	up	CDKN1A	down	SNHG12	up	hsa-miR-5100	dowr	BCYRN1	up	CDKN1A	down	RP11-480I12.7	down	TUBD1	up
RP11-442H21.2	up	CDKN1A	down	SNHG12	up	hsa-miR-5100	dowr	BCYRN1	up	CKMT2	down	RP11-480I12.7	down	TUBD1	up
RP11-442H21.2	up	CDKN1A	down	SNHG12	up	hsa-miR-5100	dowr	BCYRN1	up	CKMT2	down	RP11-480I12.7	down	TUBD1	up
RP11-521L9.1	up	CDKN1A	down	CTC-297N7.7	up	hsa-miR-5100	dowr	BCYRN1	up	CKMT2	down	RP11-480I12.7	down	TUBD1	up
RP11-521L9.1	up	CDKN1A	down	CTC-297N7.7	up	hsa-miR-5100	dowr	BCYRN1	up	CKMT2	down	RP11-480I12.7	down	TUBD1	up
RP11-521L9.1	up	CDKN1A	down	CTC-297N7.7	up	hsa-miR-5100	dowr	BCYRN1	up	CRYAB	down	RP11-480I12.7	down	UCP3	up
RP11-521L9.1	up	CDKN1A	down	MYHAS	up	hsa-miR-5100	dowr	BCYRN1	up	CSRP3	down	RP11-480I12.7	down	UCP3	up
RP11-521L9.1	up	CDKN1A	down	CYBRD1	up	hsa-miR-5100	dowr	BCYRN1	up	CTSL	down	RP11-480I12.7	down	UCP3	up
RP11-731F5.2	up	CDKN1A	down	ERAP1	up	hsa-miR-5100	dowr	BCYRN1	up	CTSL	down	RP11-480I12.7	down	ZNF302	up
RP11-731F5.2	up	CDKN1A	down	TRNT1	up	hsa-miR-5100	dowr	BCYRN1	up	CTSL	down	RP11-480I12.7	down	ZNF302	up
RP11-731F5.2	up	CDKN1A	down	AC009501.4	up	hsa-miR-6087	dowr	BCYRN1	up	CXCL10	down	RP11-480I12.7	down	ZNF354B	up
RP11-731F5.2	up	CDKN1A	down	MYHAS	up	hsa-miR-6087	dowr	BCYRN1	up	HMOX1	down	RP11-521L9.1	up	ANKRD1	down
RP11-731F5.2	up	CDKN1A	down	C1orf229	up	hsa-miR-6087	dowr	BCYRN1	up	HMOX1	down	RP11-521L9.1	up	ANKRD2	down
SNHG1	up	CDKN1A	down	C7	up	hsa-miR-6087	dowr	BCYRN1	up	MYL2	down	RP11-521L9.1	up	ANKRD2	down
SNHG1	up	CDKN1A	down	CRTAP	up	hsa-miR-6087	dowr	BCYRN1	up	MYL2	down	RP11-521L9.1	up	ANKRD2	down
SNHG1	up	CDKN1A	down	MMP14	up	hsa-miR-6087	dowr	BCYRN1	up	MYL6B	down	RP11-521L9.1	up	ANKRD2	down
SNHG1	up	CDKN1A	down	RPL27A	up	hsa-miR-6087	dowr	BCYRN1	up	MYL6B	down	RP11-521L9.1	up	C10orf10	down
SNHG1	up	CDKN1A	down	SHOX	up	hsa-miR-6087	dowr	BCYRN1	up	MYOM3	down	RP11-521L9.1	up	CASQ2	down
SNHG1	up	CDKN1A	down	AC073254.1	up	hsa-miR-6089	dowr	BCYRN1	up	NNMT	down	RP11-521L9.1	up	CCL8	down

SNHG1	up	CDKN1A	down	BCYRN1	up	hsa-miR-6089	down	BCYRN1	up	NNMT	down	RP11-521L9.1	up	CDKN1A	down
SNHG1	up	CDKN1A	down	SNHG12	up	hsa-miR-6089	down	BCYRN1	up	NNMT	down	RP11-521L9.1	up	CDKN1A	down
SNHG1	up	CDKN1A	down	SNHG12	up	hsa-miR-6089	down	BCYRN1	up	NNMT	down	RP11-521L9.1	up	CDKN1A	down
SNHG1	up	CDKN1A	down	SNHG12	up	hsa-miR-6089	down	BCYRN1	up	NNMT	down	RP11-521L9.1	up	CDKN1A	down
SNHG12	up	CDKN1A	down	SNHG12	up	hsa-miR-6089	down	BCYRN1	up	PDLIM1	down	RP11-521L9.1	up	CDKN1A	down
SNHG12	up	CDKN1A	down	RP11-521L9.1	up	hsa-miR-6089	down	BCYRN1	up	RRAD	down	RP11-521L9.1	up	CKMT2	down
SNHG12	up	CDKN1A	down	RNU12	up	hsa-miR-6089	down	BCYRN1	up	RRAD	down	RP11-521L9.1	up	CKMT2	down
SNHG12	up	CDKN1A	down	ATP9B	up	hsa-miR-6089	down	BCYRN1	up	SAT1	down	RP11-521L9.1	up	CKMT2	down
SNHG12	up	CDKN1A	down	C1orf229	up	hsa-miR-6089	down	BCYRN1	up	SAT1	down	RP11-521L9.1	up	CKMT2	down
SNHG12	up	CDKN1A	down	CCDC3	up	hsa-miR-6089	down	BCYRN1	up	SAT1	down	RP11-521L9.1	up	CRYAB	down
SNHG12	up	CDKN1A	down	CRTAP	up	hsa-miR-6089	down	BCYRN1	up	SERPINE1	down	RP11-521L9.1	up	CSRP3	down
SNHG12	up	CDKN1A	down	EEF1A1	up	hsa-miR-6089	down	BCYRN1	up	SRGN	down	RP11-521L9.1	up	CSRP3	down
SNHG12	up	CDKN1A	down	F13A1	up	hsa-miR-6089	down	BCYRN1	up	TAP1	down	RP11-521L9.1	up	CTSL	down
SNHG12	up	CDKN1A	down	MKNK2	up	hsa-miR-6089	down	BCYRN1	up	THBS1	down	RP11-521L9.1	up	HMOX1	down
SNHG12	up	CDKN1A	down	MYOG	up	hsa-miR-6089	down	BCYRN1	up	THBS1	down	RP11-521L9.1	up	HMOX1	down
SNHG12	up	CDKN1A	down	RPL27A	up	hsa-miR-6089	down	BCYRN1	up	TNFRSF12A	down	RP11-521L9.1	up	MYL6B	down
SNHG12	up	CDKN1A	down	SHOX	up	hsa-miR-6089	down	BCYRN1	up	TNFRSF12A	down	RP11-521L9.1	up	MYL6B	down
SNHG12	up	CDKN1A	down	TMSB4Y	up	hsa-miR-6089	down	BCYRN1	up	TNFRSF12A	down	RP11-521L9.1	up	MYL6B	down
SNHG12	up	CDKN1A	down	BCYRN1	up	hsa-miR-638	down	BCYRN1	up	TNFRSF12A	down	RP11-521L9.1	up	MYOM3	down
SNHG12	up	CDKN1A	down	CRTAP	up	hsa-miR-638	down	BCYRN1	up	TNNC1	down	RP11-521L9.1	up	NNMT	down
SNHG12	up	CDKN1A	down	FBN1	up	hsa-miR-638	down	BCYRN1	up	TNNC1	down	RP11-521L9.1	up	NNMT	down
SNHG12	up	CDKN1A	down	AC079586.1	up	hsa-miR-6727-	down	BCYRN1	up	TNNI1	down	RP11-521L9.1	up	NNMT	down
SNHG12	up	CDKN1A	down	DBNDD1	up	hsa-miR-6727-	down	BCYRN1	up	TNNI1	down	RP11-521L9.1	up	NNMT	down
SNHG8	up	CDKN1A	down	GSG2	up	hsa-miR-6727-	down	BCYRN1	up	TNNI1	down	RP11-521L9.1	up	NNMT	down
SNHG8	up	CDKN1A	down	TNXB	up	hsa-miR-6727-	down	BCYRN1	up	TNNI1	down	RP11-521L9.1	up	SAT1	down
SNHG8	up	CDKN1A	down	AC009501.4	up	hsa-miR-7704	down	BCYRN1	up	TNNI1	down	RP11-521L9.1	up	SAT1	down
SNHG8	up	CDKN1A	down	AC123886.2	up	hsa-miR-7704	down	BCYRN1	up	TPM3	down	RP11-521L9.1	up	SAT1	down
SNHG8	up	CDKN1A	down	MYHAS	up	hsa-miR-7704	down	BCYRN1	up	TPM3	down	RP11-521L9.1	up	SERPINE1	down
SNHG9	up	CDKN1A	down	APOE	up	hsa-miR-7704	down	CTA-14H9.5	up	ANKRD2	down	RP11-521L9.1	up	SRGN	down
SNHG9	up	CDKN1A	down	GAS1	up	hsa-miR-7704	down	CTA-14H9.5	up	ANKRD2	down	RP11-521L9.1	up	TAP1	down
SNHG9	up	CDKN1A	down	CTD-2033D15.3	down	hsa-miR-7847-	up	CTA-14H9.5	up	ANKRD2	down	RP11-521L9.1	up	THBS1	down
SNORA76C	up	CDKN1A	down	BCL3	down	hsa-miR-7847-	up	CTA-14H9.5	up	ANKRD2	down	RP11-521L9.1	up	THBS1	down
SNORA76C	up	CDKN1A	down	BTG2	down	hsa-miR-7847-	up	CTA-14H9.5	up	C10orf10	down	RP11-521L9.1	up	TNNC1	down
SNORA76C	up	CDKN1A	down	CISH	down	hsa-miR-7847-	up	CTA-14H9.5	up	SOCS3	down	RP11-521L9.1	up	TNNC1	down
SNORA76C	up	CDKN1A	down	CSRNP1	down	hsa-miR-7847-	up	CTA-14H9.5	up	THBS1	down	RP11-521L9.1	up	TNNI1	down
SNORA76C	up	CDKN1A	down	DGKD	down	hsa-miR-7847-	up	CTA-14H9.5	up	THBS1	down	RP11-521L9.1	up	TNNI1	down
**hsa-let-7e-5p**	**up**	**CDKN1A**	**down**	HBEGF	down	hsa-miR-7847-	up	CTA-14H9.5	up	TNNI1	down	RP11-521L9.1	up	TNNI1	down
**hsa-let-7i-5p**	**up**	**CDKN1A**	**down**	MIDN	down	hsa-miR-7847-	up	CTA-14H9.5	up	TNNI1	down	RP11-521L9.1	up	TNNI1	down
**hsa-miR-4459**	**up**	**CDKN1A**	**down**	MYLK3	down	hsa-miR-7847-	up	CTA-14H9.5	up	TNNI1	down	RP11-521L9.1	up	TPM3	down
CTD-2033D15.1	down	DDIT4	up	TNFSF9	down	hsa-miR-7847-	up	CTA-14H9.5	up	TNNI1	down	RP11-521L9.1	up	TPM3	down
CTD-2033D15.1	down	DDIT4	up	TNNI1	down	hsa-miR-7847-	up	CTA-14H9.5	up	TNNI1	down	RP11-603J24.14	down	BLOC1S6	up
EAF1-AS1	down	DDIT4	up	XIRP1	down	hsa-miR-7847-	up	CTC-260E6.6	up	ACTA1	down	RP11-603J24.14	down	C1QA	up
LINC01405	down	DDIT4	up	BCYRN1	up	hsa-miR-7977	down	CTC-260E6.6	up	ANKRD1	down	RP11-603J24.14	down	C1QA	up
LINC01405	down	DDIT4	up	SNHG12	up	hsa-miR-7977	down	CTC-260E6.6	up	ANKRD2	down	RP11-603J24.14	down	C1QA	up
RP11-451G4.2	down	DDIT4	up	SNHG12	up	hsa-miR-7977	down	CTC-260E6.6	up	ANKRD2	down	RP11-603J24.14	down	C3	up
RP11-480I12.7	down	DDIT4	up	SNHG12	up	hsa-miR-7977	down	CTC-260E6.6	up	ANKRD2	down	RP11-603J24.14	down	CEBPZOS	up
RP11-91P24.7	down	DDIT4	up	SNHG12	up	hsa-miR-7977	down	CTC-260E6.6	up	BHLHE40	down	RP11-603J24.14	down	CEBPZOS	up
**hsa-miR-3196**	**down**	**DDIT4**	**up**	CTC-297N7.7	up	hsa-miR-7977	down	CTC-260E6.6	up	CASQ2	down	RP11-603J24.14	down	CHRNA1	up
CTD-2033D15.1	down	DNMT3A	up	CTC-297N7.7	up	hsa-miR-7977	down	CTC-260E6.6	up	CCL8	down	RP11-603J24.14	down	CHRNA1	up
CTD-2033D15.1	down	DNMT3A	up	CTC-297N7.7	up	hsa-miR-7977	down	CTC-260E6.6	up	CDKN1A	down	RP11-603J24.14	down	CHRNA1	up
EAF1-AS1	down	DNMT3A	up	MYHAS	up	hsa-miR-7977	down	CTC-260E6.6	up	CDKN1A	down	RP11-603J24.14	down	CHRNA1	up

LINC01405	down	DNMT3A	up	FBLN1	up	hsa-miR-7977	down	CTC-260E6.6	up	CDKN1A	down	RP11-603J24.14	down	CHRNA1	up
LINC01405	down	DNMT3A	up	TBCC	up	hsa-miR-7977	down	CTC-260E6.6	up	CDKN1A	down	RP11-603J24.14	down	CHRNA1	up
RP11-451G4.2	down	DNMT3A	up	RP11-91P24.7	down	hsa-miR-92a-3	up	CTC-260E6.6	up	CKMT2	down	RP11-603J24.14	down	COL1A2	up
RP11-705C15.5	down	DNMT3A	up	BTG2	down	hsa-miR-92a-3	up	CTC-260E6.6	up	CKMT2	down	RP11-603J24.14	down	COL1A2	up
RP11-91P24.7	down	DNMT3A	up	FOSL2	down	hsa-miR-92a-3	up	CTC-260E6.6	up	CKMT2	down	RP11-603J24.14	down	CTSK	up
**hsa-miR-24-3p**	**down**	**DNMT3A**	**up**	RP11-91P24.7	down	hsa-miR-92b-3	up	CTC-260E6.6	up	CKMT2	down	RP11-603J24.14	down	CTSK	up
CTD-2033D15.1	down	EEF1A1	up	FOSL2	down	hsa-miR-92b-3	up	CTC-260E6.6	up	CSRP3	down	RP11-603J24.14	down	EEF1A1	up
CTD-2033D15.1	down	EEF1A1	up					CTC-260E6.6	up	CSRP3	down	RP11-603J24.14	down	EEF1A1	up
CTD-2033D15.1	down	EEF1A1	up					CTC-260E6.6	up	CTSL	down	RP11-603J24.14	down	EEF1A1	up
CTD-2033D15.1	down	EEF1A1	up					CTC-260E6.6	up	CTSL	down	RP11-603J24.14	down	EEF1A1	up
CTD-2033D15.1	down	EEF1A1	up					CTC-260E6.6	up	CTSL	down	RP11-603J24.14	down	EEF1A1	up
CTD-2033D15.1	down	EEF1A1	up					CTC-260E6.6	up	GADD45G	down	RP11-603J24.14	down	EEF1A1	up
CTD-2033D15.1	down	EEF1A1	up					CTC-260E6.6	up	GADD45G	down	RP11-603J24.14	down	EIF4A2	up
CTD-2033D15.1	down	EEF1A1	up					CTC-260E6.6	up	HMOX1	down	RP11-603J24.14	down	EIF4A2	up
CTD-2033D15.1	down	EEF1A1	up					CTC-260E6.6	up	HMOX1	down	RP11-603J24.14	down	EIF4A2	up
CTD-2033D15.1	down	EEF1A1	up					CTC-260E6.6	up	MYL2	down	RP11-603J24.14	down	EIF4A2	up
EAF1-AS1	down	EEF1A1	up					CTC-260E6.6	up	MYL2	down	RP11-603J24.14	down	EIF4A2	up
EAF1-AS1	down	EEF1A1	up					CTC-260E6.6	up	MYL6B	down	RP11-603J24.14	down	FAM129A	up
EAF1-AS1	down	EEF1A1	up					CTC-260E6.6	up	MYL6B	down	RP11-603J24.14	down	GREM1	up
LINC01405	down	EEF1A1	up					CTC-260E6.6	up	MYL6B	down	RP11-603J24.14	down	HBA1	up
LINC01405	down	EEF1A1	up					CTC-260E6.6	up	MYOM3	down	RP11-603J24.14	down	HBA1	up
LINC01405	down	EEF1A1	up					CTC-260E6.6	up	NNMT	down	RP11-603J24.14	down	HBA2	up
LINC01405	down	EEF1A1	up					CTC-260E6.6	up	NNMT	down	RP11-603J24.14	down	HBA2	up
LINC01405	down	EEF1A1	up					CTC-260E6.6	up	NNMT	down	RP11-603J24.14	down	HBB	up
LINC01405	down	EEF1A1	up					CTC-260E6.6	up	PDLIM1	down	RP11-603J24.14	down	HBD	up
LINC01405	down	EEF1A1	up					CTC-260E6.6	up	SERPINE1	down	RP11-603J24.14	down	HBD	up
LINC01405	down	EEF1A1	up					CTC-260E6.6	up	TAP1	down	RP11-603J24.14	down	HBD	up
LINC01405	down	EEF1A1	up					CTC-260E6.6	up	TNFRSF12A	down	RP11-603J24.14	down	HIST1H4J	up
LINC01405	down	EEF1A1	up					CTC-260E6.6	up	TNFRSF12A	down	RP11-603J24.14	down	HIST1H4K	up
LINC01405	down	EEF1A1	up					CTC-260E6.6	up	TNFRSF12A	down	RP11-603J24.14	down	HTRA1	up
RP11-451G4.2	down	EEF1A1	up					CTC-260E6.6	up	TNFRSF12A	down	RP11-603J24.14	down	IGFBP4	up
RP11-451G4.2	down	EEF1A1	up					CTC-260E6.6	up	TNNC1	down	RP11-603J24.14	down	IGFBP7	up
RP11-451G4.2	down	EEF1A1	up					CTC-260E6.6	up	TNNC1	down	RP11-603J24.14	down	IGKC	up
RP11-451G4.2	down	EEF1A1	up					CTC-260E6.6	up	TNNI1	down	RP11-603J24.14	down	IGKC	up
RP11-451G4.2	down	EEF1A1	up					CTC-260E6.6	up	TNNI1	down	RP11-603J24.14	down	IGKC	up
RP11-451G4.2	down	EEF1A1	up					CTC-260E6.6	up	TNNI1	down	RP11-603J24.14	down	IMPA2	up
RP11-480I12.7	down	EEF1A1	up					CTC-260E6.6	up	TNNI1	down	RP11-603J24.14	down	KIAA1328	up
RP11-480I12.7	down	EEF1A1	up					CTC-260E6.6	up	TNNI1	down	RP11-603J24.14	down	LAMC1	up
RP11-480I12.7	down	EEF1A1	up					CTC-260E6.6	up	TNNI1	down	RP11-603J24.14	down	MKNK2	up
RP11-480I12.7	down	EEF1A1	up					CTC-260E6.6	up	TPM3	down	RP11-603J24.14	down	MKNK2	up
RP11-480I12.7	down	EEF1A1	up					CTC-297N7.7	up	ACTA1	down	RP11-603J24.14	down	MKNK2	up
RP11-480I12.7	down	EEF1A1	up					CTC-297N7.7	up	ACTA1	down	RP11-603J24.14	down	MKNK2	up
RP11-480I12.7	down	EEF1A1	up					CTC-297N7.7	up	ANKRD2	down	RP11-603J24.14	down	MKNK2	up
RP11-603J24.14	down	EEF1A1	up					CTC-297N7.7	up	ANKRD2	down	RP11-603J24.14	down	MKNK2	up
RP11-603J24.14	down	EEF1A1	up					CTC-297N7.7	up	ANKRD2	down	RP11-603J24.14	down	MMP14	up
RP11-603J24.14	down	EEF1A1	up					CTC-297N7.7	up	ANKRD2	down	RP11-603J24.14	down	MORC4	up
RP11-603J24.14	down	EEF1A1	up					CTC-297N7.7	up	BHLHE40	down	RP11-603J24.14	down	MSS51	up
RP11-603J24.14	down	EEF1A1	up					CTC-297N7.7	up	C10orf10	down	RP11-603J24.14	down	MSS51	up
RP11-603J24.14	down	EEF1A1	up					CTC-297N7.7	up	CDKN1A	down	RP11-603J24.14	down	MSS51	up
RP11-705C15.5	down	EEF1A1	up					CTC-297N7.7	up	CDKN1A	down	RP11-603J24.14	down	MYH1	up
RP11-705C15.5	down	EEF1A1	up					CTC-297N7.7	up	CDKN1A	down	RP11-603J24.14	down	MYH4	up

RP11-705C15.5	down	EEF1A1	up
RP11-705C15.5	down	EEF1A1	up
RP11-91P24.7	down	EEF1A1	up
RP11-91P24.7	down	EEF1A1	up
RP11-91P24.7	down	EEF1A1	up
RP11-91P24.7	down	EEF1A1	up
RP11-91P24.7	down	EEF1A1	up
**hsa-miR-27b-3p**	**down**	**EEF1A1**	**up**
**hsa-miR-6089**	**down**	**EEF1A1**	**up**
EAF1-AS1	down	GREM1	up
LINC01405	down	GREM1	up
LINC01405	down	GREM1	up
RP11-451G4.2	down	GREM1	up
RP11-480I12.7	down	GREM1	up
RP11-603J24.14	down	GREM1	up
RP11-705C15.5	down	GREM1	up
RP11-91P24.7	down	GREM1	up
**hsa-miR-27b-3p**	**down**	**GREM1**	**up**
CTD-2033D15.1	down	GSG2	up
CTD-2033D15.1	down	GSG2	up
LINC01405	down	GSG2	up
LINC01405	down	GSG2	up
RP11-451G4.2	down	GSG2	up
RP11-480I12.7	down	GSG2	up
RP11-705C15.5	down	GSG2	up
RP11-91P24.7	down	GSG2	up
**hsa-miR-6727-5p**	**down**	**GSG2**	**up**
CTD-2033D15.1	down	IGFBP4	up
CTD-2033D15.1	down	IGFBP4	up
EAF1-AS1	down	IGFBP4	up
LINC01405	down	IGFBP4	up
LINC01405	down	IGFBP4	up
RP11-451G4.2	down	IGFBP4	up
RP11-480I12.7	down	IGFBP4	up
RP11-603J24.14	down	IGFBP4	up
RP11-705C15.5	down	IGFBP4	up
**hsa-miR-378c**	**down**	**IGFBP4**	**up**
**hsa-miR-378g**	**down**	**IGFBP4**	**up**
RP11-386J22.3	up	KLF9	down
**hsa-let-7e-5p**	**up**	**KLF9**	**down**
**hsa-miR-30d-5p**	**up**	**KLF9**	**down**
RP11-451G4.2	down	MGP	up
RP11-451G4.2	down	MGP	up
RP11-451G4.2	down	MGP	up
RP11-480I12.7	down	MGP	up
RP11-480I12.7	down	MGP	up
RP11-91P24.7	down	MGP	up
**hsa-miR-3196**	**down**	**MGP**	**up**
CTD-2033D15.1	down	MKNK2	up
CTD-2033D15.1	down	MKNK2	up
CTD-2033D15.1	down	MKNK2	up

CTC-297N7.7	up	CDKN1A	down	RP11-603J24.14	down	MYOC	up
CTC-297N7.7	up	CDKN1A	down	RP11-603J24.14	down	MYOC	up
CTC-297N7.7	up	CKMT2	down	RP11-603J24.14	down	MYOG	up
CTC-297N7.7	up	CKMT2	down	RP11-603J24.14	down	PRRG3	up
CTC-297N7.7	up	CKMT2	down	RP11-603J24.14	down	RNASE1	up
CTC-297N7.7	up	CKMT2	down	RP11-603J24.14	down	RNASE1	up
CTC-297N7.7	up	CTSL	down	RP11-603J24.14	down	RNASE1	up
CTC-297N7.7	up	CTSL	down	RP11-603J24.14	down	RNASE1	up
CTC-297N7.7	up	CTSL	down	RP11-603J24.14	down	RPL27A	up
CTC-297N7.7	up	GADD45G	down	RP11-603J24.14	down	RPS3	up
CTC-297N7.7	up	GADD45G	down	RP11-603J24.14	down	S100A10	up
CTC-297N7.7	up	HMOX1	down	RP11-603J24.14	down	SPARC	up
CTC-297N7.7	up	HMOX1	down	RP11-603J24.14	down	SPARC	up
CTC-297N7.7	up	MYL2	down	RP11-603J24.14	down	SPARC	up
CTC-297N7.7	up	MYL2	down	RP11-603J24.14	down	SPARC	up
CTC-297N7.7	up	MYL6B	down	RP11-603J24.14	down	SPARC	up
CTC-297N7.7	up	MYL6B	down	RP11-603J24.14	down	SPARC	up
CTC-297N7.7	up	MYL6B	down	RP11-603J24.14	down	STAG2	up
CTC-297N7.7	up	MYOM3	down	RP11-603J24.14	down	STAG2	up
CTC-297N7.7	up	NNMT	down	RP11-603J24.14	down	TBC1D8B	up
CTC-297N7.7	up	NNMT	down	RP11-603J24.14	down	TMEM107	up
CTC-297N7.7	up	NNMT	down	RP11-603J24.14	down	TMSB4X	up
CTC-297N7.7	up	NNMT	down	RP11-603J24.14	down	TMSB4X	up
CTC-297N7.7	up	NNMT	down	RP11-603J24.14	down	TMSB4X	up
CTC-297N7.7	up	PDLIM1	down	RP11-603J24.14	down	TMSB4X	up
CTC-297N7.7	up	RRAD	down	RP11-603J24.14	down	TPM4	up
CTC-297N7.7	up	RRAD	down	RP11-603J24.14	down	TPM4	up
CTC-297N7.7	up	SAT1	down	RP11-603J24.14	down	TRIM22	up
CTC-297N7.7	up	SAT1	down	RP11-603J24.14	down	TRIM22	up
CTC-297N7.7	up	SAT1	down	RP11-603J24.14	down	TRIM22	up
CTC-297N7.7	up	SERPINE1	down	RP11-603J24.14	down	TRIM22	up
CTC-297N7.7	up	SRGN	down	RP11-603J24.14	down	TUBD1	up
CTC-297N7.7	up	TAP1	down	RP11-603J24.14	down	TUBD1	up
CTC-297N7.7	up	THBS1	down	RP11-603J24.14	down	TUBD1	up
CTC-297N7.7	up	THBS1	down	RP11-603J24.14	down	TUBD1	up
CTC-297N7.7	up	TNFRSF12A	down	RP11-603J24.14	down	TUBD1	up
CTC-297N7.7	up	TNFRSF12A	down	RP11-603J24.14	down	ZNF302	up
CTC-297N7.7	up	TNFRSF12A	down	RP11-603J24.14	down	ZNF302	up
CTC-297N7.7	up	TNFRSF12A	down	RP11-603J24.14	down	ZNF354B	up
CTC-297N7.7	up	TNFRSF12A	down	RP11-701H24.7	up	GADD45G	down
CTC-297N7.7	up	TNNC1	down	RP11-701H24.7	up	TAP1	down
CTC-297N7.7	up	TNNC1	down	RP11-705C15.5	down	ADAM10	up
CTC-297N7.7	up	TNNI1	down	RP11-705C15.5	down	BLOC1S6	up
CTC-297N7.7	up	TNNI1	down	RP11-705C15.5	down	C1orf229	up
CTC-297N7.7	up	TPM3	down	RP11-705C15.5	down	C1QA	up
CTC-297N7.7	up	ANKRD1	down	RP11-705C15.5	down	C1QA	up
CTC-297N7.7	up	ANKRD2	down	RP11-705C15.5	down	C1QA	up
CTC-297N7.7	up	ANKRD2	down	RP11-705C15.5	down	C3	up
CTC-297N7.7	up	ANKRD2	down	RP11-705C15.5	down	C3	up
CTC-297N7.7	up	C10orf10	down	RP11-705C15.5	down	CEBPZOS	up
CTC-297N7.7	up	CDKN1A	down	RP11-705C15.5	down	CEBPZOS	up

CTD-2033D15.1	down	MKNK2	up
CTD-2033D15.1	down	MKNK2	up
CTD-2033D15.1	down	MKNK2	up
CTD-2033D15.1	down	MKNK2	up
CTD-2033D15.1	down	MKNK2	up
CTD-2033D15.1	down	MKNK2	up
EAF1-AS1	down	MKNK2	up
EAF1-AS1	down	MKNK2	up
EAF1-AS1	down	MKNK2	up
EAF1-AS1	down	MKNK2	up
EAF1-AS1	down	MKNK2	up
EAF1-AS1	down	MKNK2	up
EAF1-AS1	down	MKNK2	up
LINC01405	down	MKNK2	up
LINC01405	down	MKNK2	up
LINC01405	down	MKNK2	up
LINC01405	down	MKNK2	up
LINC01405	down	MKNK2	up
LINC01405	down	MKNK2	up
LINC01405	down	MKNK2	up
LINC01405	down	MKNK2	up
LINC01405	down	MKNK2	up
RP11-451G4.2	down	MKNK2	up
RP11-451G4.2	down	MKNK2	up
RP11-451G4.2	down	MKNK2	up
RP11-451G4.2	down	MKNK2	up
RP11-451G4.2	down	MKNK2	up
RP11-451G4.2	down	MKNK2	up
RP11-451G4.2	down	MKNK2	up
RP11-480I12.7	down	MKNK2	up
RP11-480I12.7	down	MKNK2	up
RP11-480I12.7	down	MKNK2	up
RP11-480I12.7	down	MKNK2	up
RP11-480I12.7	down	MKNK2	up
RP11-480I12.7	down	MKNK2	up
RP11-603J24.14	down	MKNK2	up
RP11-603J24.14	down	MKNK2	up
RP11-603J24.14	down	MKNK2	up
RP11-603J24.14	down	MKNK2	up
RP11-603J24.14	down	MKNK2	up
RP11-603J24.14	down	MKNK2	up
RP11-705C15.5	down	MKNK2	up
RP11-705C15.5	down	MKNK2	up
RP11-705C15.5	down	MKNK2	up
RP11-705C15.5	down	MKNK2	up
RP11-705C15.5	down	MKNK2	up
RP11-705C15.5	down	MKNK2	up
RP11-705C15.5	down	MKNK2	up
RP11-91P24.7	down	MKNK2	up
RP11-91P24.7	down	MKNK2	up
RP11-91P24.7	down	MKNK2	up

CTC-297N7.7	up	CDKN1A	down	RP11-705C15.5	down	CHRNA1	up
CTC-297N7.7	up	CDKN1A	down	RP11-705C15.5	down	CHRNA1	up
CTC-297N7.7	up	CDKN1A	down	RP11-705C15.5	down	CHRNA1	up
CTC-297N7.7	up	CDKN1A	down	RP11-705C15.5	down	CHRNA1	up
CTC-297N7.7	up	MYL6B	down	RP11-705C15.5	down	CHRNA1	up
CTC-297N7.7	up	MYOM3	down	RP11-705C15.5	down	CHRNA1	up
CTC-297N7.7	up	NNMT	down	RP11-705C15.5	down	CTSK	up
CTC-297N7.7	up	NNMT	down	RP11-705C15.5	down	CTSK	up
CTC-297N7.7	up	NNMT	down	RP11-705C15.5	down	DNMT3A	up
CTC-297N7.7	up	NNMT	down	RP11-705C15.5	down	EEF1A1	up
CTC-297N7.7	up	NNMT	down	RP11-705C15.5	down	EEF1A1	up
CTC-297N7.7	up	PDLIM1	down	RP11-705C15.5	down	EEF1A1	up
CTC-297N7.7	up	RRAD	down	RP11-705C15.5	down	EEF1A1	up
CTC-297N7.7	up	RRAD	down	RP11-705C15.5	down	EIF4A2	up
CTC-297N7.7	up	SAT1	down	RP11-705C15.5	down	EIF4A2	up
CTC-297N7.7	up	SAT1	down	RP11-705C15.5	down	EIF4A2	up
CTC-297N7.7	up	SAT1	down	RP11-705C15.5	down	EIF4A2	up
CTC-297N7.7	up	SERPINE1	down	RP11-705C15.5	down	EIF4A2	up
CTC-297N7.7	up	SRGN	down	RP11-705C15.5	down	EIF4A2	up
CTC-297N7.7	up	THBS1	down	RP11-705C15.5	down	EIF4A2	up
CTC-297N7.7	up	TNFRSF12A	down	RP11-705C15.5	down	FAM129A	up
CTC-297N7.7	up	TNFRSF12A	down	RP11-705C15.5	down	GREM1	up
CTC-297N7.7	up	TNFRSF12A	down	RP11-705C15.5	down	GSG2	up
CTC-297N7.7	up	TNFRSF12A	down	RP11-705C15.5	down	HBA1	up
CTC-297N7.7	up	TNFRSF12A	down	RP11-705C15.5	down	HBA1	up
CTC-297N7.7	up	TNNC1	down	RP11-705C15.5	down	HBA2	up
CTC-297N7.7	up	TNNC1	down	RP11-705C15.5	down	HBA2	up
CTC-297N7.7	up	TNNI1	down	RP11-705C15.5	down	HBB	up
CTC-297N7.7	up	TNNI1	down	RP11-705C15.5	down	HBD	up
CTC-297N7.7	up	TNNI1	down	RP11-705C15.5	down	HBD	up
CTC-297N7.7	up	TNNI1	down	RP11-705C15.5	down	HBD	up
CTC-297N7.7	up	TNNI1	down	RP11-705C15.5	down	HIST1H4J	up
CTC-297N7.7	up	TPM3	down	RP11-705C15.5	down	HIST1H4K	up
CTC-297N7.7	up	ACTA1	down	RP11-705C15.5	down	HTRA1	up
CTC-297N7.7	up	ACTA1	down	RP11-705C15.5	down	HTRA1	up
CTC-297N7.7	up	ANKRD2	down	RP11-705C15.5	down	IGFBP4	up
CTC-297N7.7	up	ANKRD2	down	RP11-705C15.5	down	IGFBP7	up
CTC-297N7.7	up	ANKRD2	down	RP11-705C15.5	down	IGKC	up
CTC-297N7.7	up	ANKRD2	down	RP11-705C15.5	down	IGKC	up
CTC-297N7.7	up	BHLHE40	down	RP11-705C15.5	down	IGKC	up
CTC-297N7.7	up	CASQ2	down	RP11-705C15.5	down	IMPA2	up
CTC-297N7.7	up	CDKN1A	down	RP11-705C15.5	down	IMPA2	up
CTC-297N7.7	up	CDKN1A	down	RP11-705C15.5	down	KIAA1328	up
CTC-297N7.7	up	CDKN1A	down	RP11-705C15.5	down	LAMC1	up
CTC-297N7.7	up	CDKN1A	down	RP11-705C15.5	down	MKNK2	up
CTC-297N7.7	up	CDKN1A	down	RP11-705C15.5	down	MKNK2	up
CTC-297N7.7	up	CKMT2	down	RP11-705C15.5	down	MKNK2	up
CTC-297N7.7	up	CKMT2	down	RP11-705C15.5	down	MKNK2	up
CTC-297N7.7	up	CKMT2	down	RP11-705C15.5	down	MKNK2	up
CTC-297N7.7	up	CKMT2	down	RP11-705C15.5	down	MKNK2	up
CTC-297N7.7	up	CSRP3	down	RP11-705C15.5	down	MKNK2	up

RP11-91P24.7	down	MKNK2	up
RP11-91P24.7	down	MKNK2	up
RP11-91P24.7	down	MKNK2	up
RP11-91P24.7	down	MKNK2	up
**hsa-miR-26a-5p**	**down**	**MKNK2**	**up**
**hsa-miR-26b-5p**	**down**	**MKNK2**	**up**
**hsa-miR-27b-3p**	**down**	**MKNK2**	**up**
**hsa-miR-4734**	**down**	**MKNK2**	**up**
**hsa-miR-6089**	**down**	**MKNK2**	**up**
CTD-2033D15.1	down	MMP14	up
EAF1-AS1	down	MMP14	up
LINC01405	down	MMP14	up
LINC01405	down	MMP14	up
RP11-451G4.2	down	MMP14	up
RP11-480I12.7	down	MMP14	up
RP11-603J24.14	down	MMP14	up
RP11-705C15.5	down	MMP14	up
RP11-91P24.7	down	MMP14	up
**hsa-miR-24-3p**	**down**	**MMP14**	**up**
**hsa-miR-6087**	**down**	**MMP14**	**up**
AC007228.9	up	MYL2	down
AC007228.9	up	MYL2	down
AC009501.4	up	MYL2	down
AC009501.4	up	MYL2	down
AC064871.3	up	MYL2	down
AC064871.3	up	MYL2	down
AC073254.1	up	MYL2	down
AC073254.1	up	MYL2	down
AC073254.1	up	MYL2	down
AC073254.1	up	MYL2	down
AC079586.1	up	MYL2	down
AC079586.1	up	MYL2	down
AC123886.2	up	MYL2	down
AC123886.2	up	MYL2	down
BCYRN1	up	MYL2	down
BCYRN1	up	MYL2	down
CTC-260E6.6	up	MYL2	down
CTC-260E6.6	up	MYL2	down
CTC-297N7.7	up	MYL2	down
CTC-297N7.7	up	MYL2	down
CTC-297N7.7	up	MYL2	down
CTC-297N7.7	up	MYL2	down
CTC-537E7.2	up	MYL2	down
CTC-537E7.2	up	MYL2	down
MYHAS	up	MYL2	down
MYHAS	up	MYL2	down
RNU12	up	MYL2	down
RNU12	up	MYL2	down
RP11-357D18.1	up	MYL2	down
RP11-357D18.1	up	MYL2	down
RP11-386J22.3	up	MYL2	down

CTC-297N7.7	up	CSRP3	down	RP11-705C15.5	down	MMP14	up
CTC-297N7.7	up	CTSL	down	RP11-705C15.5	down	MORC4	up
CTC-297N7.7	up	CTSL	down	RP11-705C15.5	down	MSS51	up
CTC-297N7.7	up	CTSL	down	RP11-705C15.5	down	MSS51	up
CTC-297N7.7	up	GADD45G	down	RP11-705C15.5	down	MSS51	up
CTC-297N7.7	up	GADD45G	down	RP11-705C15.5	down	MYH1	up
CTC-297N7.7	up	HMOX1	down	RP11-705C15.5	down	MYH4	up
CTC-297N7.7	up	HMOX1	down	RP11-705C15.5	down	MYOC	up
CTC-297N7.7	up	MYL2	down	RP11-705C15.5	down	MYOC	up
CTC-297N7.7	up	MYL2	down	RP11-705C15.5	down	MYOG	up
CTC-297N7.7	up	MYL6B	down	RP11-705C15.5	down	PRRG3	up
CTC-297N7.7	up	NNMT	down	RP11-705C15.5	down	RNASE1	up
CTC-297N7.7	up	PDLIM1	down	RP11-705C15.5	down	RPL27A	up
CTC-297N7.7	up	RRAD	down	RP11-705C15.5	down	RPS3	up
CTC-297N7.7	up	RRAD	down	RP11-705C15.5	down	SPARC	up
CTC-297N7.7	up	SERPINE1	down	RP11-705C15.5	down	SPARC	up
CTC-297N7.7	up	SRGN	down	RP11-705C15.5	down	SPARC	up
CTC-297N7.7	up	TAP1	down	RP11-705C15.5	down	SPARC	up
CTC-297N7.7	up	THBS1	down	RP11-705C15.5	down	STAG2	up
CTC-297N7.7	up	THBS1	down	RP11-705C15.5	down	STAG2	up
CTC-297N7.7	up	TNFRSF12A	down	RP11-705C15.5	down	TMSB4X	up
CTC-297N7.7	up	TNFRSF12A	down	RP11-705C15.5	down	TMSB4X	up
CTC-297N7.7	up	TNFRSF12A	down	RP11-705C15.5	down	TMSB4X	up
CTC-297N7.7	up	TNFRSF12A	down	RP11-705C15.5	down	TPM4	up
CTC-297N7.7	up	TNNC1	down	RP11-705C15.5	down	TPM4	up
CTC-297N7.7	up	TNNC1	down	RP11-705C15.5	down	TRIM22	up
CTC-297N7.7	up	TNNI1	down	RP11-705C15.5	down	TRIM22	up
CTC-297N7.7	up	TNNI1	down	RP11-705C15.5	down	TRIM22	up
CTC-297N7.7	up	TNNI1	down	RP11-705C15.5	down	TRIM22	up
CTC-297N7.7	up	TNNI1	down	RP11-705C15.5	down	TRIM22	up
CTC-297N7.7	up	TNNI1	down	RP11-705C15.5	down	TUBD1	up
CTC-297N7.7	up	TPM3	down	RP11-705C15.5	down	TUBD1	up
CTC-297N7.7	up	ACTA1	down	RP11-705C15.5	down	TUBD1	up
CTC-297N7.7	up	BHLHE40	down	RP11-705C15.5	down	TUBD1	up
CTC-297N7.7	up	C10orf10	down	RP11-705C15.5	down	TUBD1	up
CTC-297N7.7	up	CASQ2	down	RP11-705C15.5	down	UCP3	up
CTC-297N7.7	up	CDKN1A	down	RP11-705C15.5	down	UCP3	up
CTC-297N7.7	up	CDKN1A	down	RP11-705C15.5	down	UCP3	up
CTC-297N7.7	up	CDKN1A	down	RP11-705C15.5	down	ZNF302	up
CTC-297N7.7	up	CDKN1A	down	RP11-705C15.5	down	ZNF302	up
CTC-297N7.7	up	CKMT2	down	RP11-705C15.5	down	ZNF354B	up
CTC-297N7.7	up	CKMT2	down	RP11-731F5.2	up	ANKRD1	down
CTC-297N7.7	up	CKMT2	down	RP11-731F5.2	up	ANKRD2	down
CTC-297N7.7	up	CKMT2	down	RP11-731F5.2	up	BHLHE40	down
CTC-297N7.7	up	CSRP3	down	RP11-731F5.2	up	C10orf10	down
CTC-297N7.7	up	CSRP3	down	RP11-731F5.2	up	CASQ2	down
CTC-297N7.7	up	CTSL	down	RP11-731F5.2	up	CCL2	down
CTC-297N7.7	up	CTSL	down	RP11-731F5.2	up	CDKN1A	down
CTC-297N7.7	up	CTSL	down	RP11-731F5.2	up	CDKN1A	down
CTC-297N7.7	up	GADD45G	down	RP11-731F5.2	up	CDKN1A	down
CTC-297N7.7	up	GADD45G	down	RP11-731F5.2	up	CDKN1A	down

RP11-386J22.3	up	MYL2	down
RP11-403P17.4	up	MYL2	down
RP11-403P17.4	up	MYL2	down
RP11-442H21.2	up	MYL2	down
RP11-442H21.2	up	MYL2	down
RP11-731F5.2	up	MYL2	down
RP11-731F5.2	up	MYL2	down
SNHG1	up	MYL2	down
SNHG1	up	MYL2	down
SNHG1	up	MYL2	down
SNHG1	up	MYL2	down
SNHG12	up	MYL2	down
SNHG12	up	MYL2	down
SNHG12	up	MYL2	down
SNHG12	up	MYL2	down
SNHG12	up	MYL2	down
SNHG12	up	MYL2	down
SNHG12	up	MYL2	down
SNHG12	up	MYL2	down
SNHG12	up	MYL2	down
SNHG8	up	MYL2	down
SNHG8	up	MYL2	down
SNHG9	up	MYL2	down
SNHG9	up	MYL2	down
SNORA76C	up	MYL2	down
SNORA76C	up	MYL2	down
**hsa-miR-486-3p**	**up**	**MYL2**	**down**
CTD-2033D15.1	down	MYOG	up
CTD-2033D15.1	down	MYOG	up
LINC01405	down	MYOG	up
LINC01405	down	MYOG	up
RP11-451G4.2	down	MYOG	up
RP11-603J24.14	down	MYOG	up
RP11-705C15.5	down	MYOG	up
RP11-91P24.7	down	MYOG	up
**hsa-miR-6089**	**down**	**MYOG**	**up**
CTD-2033D15.1	down	PRRG3	up
CTD-2033D15.1	down	PRRG3	up
EAF1-AS1	down	PRRG3	up
LINC01405	down	PRRG3	up
LINC01405	down	PRRG3	up
RP11-451G4.2	down	PRRG3	up
RP11-480I12.7	down	PRRG3	up
RP11-603J24.14	down	PRRG3	up
RP11-705C15.5	down	PRRG3	up
RP11-91P24.7	down	PRRG3	up
**hsa-miR-4443**	**down**	**PRRG3**	**up**
CTD-2033D15.1	down	RPL27A	up
EAF1-AS1	down	RPL27A	up
LINC01405	down	RPL27A	up
RP11-451G4.2	down	RPL27A	up

CTC-297N7.7	up	HMOX1	down	RP11-731F5.2	up	CDKN1A	down
CTC-297N7.7	up	HMOX1	down	RP11-731F5.2	up	CKMT2	down
CTC-297N7.7	up	MYL6B	down	RP11-731F5.2	up	CKMT2	down
CTC-297N7.7	up	MYL6B	down	RP11-731F5.2	up	CKMT2	down
CTC-297N7.7	up	MYL6B	down	RP11-731F5.2	up	CKMT2	down
CTC-297N7.7	up	NNMT	down	RP11-731F5.2	up	CRYAB	down
CTC-297N7.7	up	NNMT	down	RP11-731F5.2	up	CSRP3	down
CTC-297N7.7	up	PDLIM1	down	RP11-731F5.2	up	CSRP3	down
CTC-297N7.7	up	RRAD	down	RP11-731F5.2	up	CTSL	down
CTC-297N7.7	up	RRAD	down	RP11-731F5.2	up	CTSL	down
CTC-297N7.7	up	SAT1	down	RP11-731F5.2	up	CTSL	down
CTC-297N7.7	up	SAT1	down	RP11-731F5.2	up	GADD45G	down
CTC-297N7.7	up	SAT1	down	RP11-731F5.2	up	MYL2	down
CTC-297N7.7	up	SERPINE1	down	RP11-731F5.2	up	MYL2	down
CTC-297N7.7	up	THBS1	down	RP11-731F5.2	up	MYL6B	down
CTC-297N7.7	up	TNFRSF12A	down	RP11-731F5.2	up	MYL6B	down
CTC-297N7.7	up	TNFRSF12A	down	RP11-731F5.2	up	MYL6B	down
CTC-297N7.7	up	TNFRSF12A	down	RP11-731F5.2	up	NNMT	down
CTC-297N7.7	up	TNFRSF12A	down	RP11-731F5.2	up	NNMT	down
CTC-297N7.7	up	TNFRSF12A	down	RP11-731F5.2	up	NNMT	down
CTC-297N7.7	up	TNNC1	down	RP11-731F5.2	up	NNMT	down
CTC-297N7.7	up	TNNC1	down	RP11-731F5.2	up	NNMT	down
CTC-297N7.7	up	TNNI1	down	RP11-731F5.2	up	PDLIM1	down
CTC-297N7.7	up	TNNI1	down	RP11-731F5.2	up	RRAD	down
CTC-297N7.7	up	TNNI1	down	RP11-731F5.2	up	RRAD	down
CTC-297N7.7	up	TNNI1	down	RP11-731F5.2	up	SERPINE1	down
CTC-297N7.7	up	TNNI1	down	RP11-731F5.2	up	SOCS3	down
CTC-297N7.7	up	TNNI1	down	RP11-731F5.2	up	SRGN	down
CTC-297N7.7	up	TPM3	down	RP11-731F5.2	up	THBS1	down
CTC-297N7.7	up	TPM3	down	RP11-731F5.2	up	THBS1	down
CTC-537E7.2	up	ANKRD2	down	RP11-731F5.2	up	TNFRSF12A	down
CTC-537E7.2	up	ANKRD2	down	RP11-731F5.2	up	TNFRSF12A	down
CTC-537E7.2	up	ANKRD2	down	RP11-731F5.2	up	TNFRSF12A	down
CTC-537E7.2	up	C10orf10	down	RP11-731F5.2	up	TNFRSF12A	down
CTC-537E7.2	up	GADD45G	down	RP11-731F5.2	up	TNNC1	down
CTC-537E7.2	up	MYL2	down	RP11-731F5.2	up	TNNC1	down
CTC-537E7.2	up	MYL2	down	RP11-731F5.2	up	TNNI1	down
CTC-537E7.2	up	PDLIM1	down	RP11-731F5.2	up	TNNI1	down
CTC-537E7.2	up	RRAD	down	RP11-731F5.2	up	TNNI1	down
CTC-537E7.2	up	RRAD	down	RP11-731F5.2	up	TNNI1	down
CTC-537E7.2	up	THBS1	down	RP11-731F5.2	up	TNNI1	down
CTC-537E7.2	up	THBS1	down	RP11-731F5.2	up	TNNI1	down
CTC-537E7.2	up	TNNC1	down	RP11-731F5.2	up	TPM3	down
CTC-537E7.2	up	TNNI1	down	RP11-731F5.2	up	TPM3	down
CTC-537E7.2	up	TNNI1	down	RP11-91P24.7	down	A2M	up
CTD-2033D15.1	down	A2M	up	RP11-91P24.7	down	A2M	up
CTD-2033D15.1	down	A2M	up	RP11-91P24.7	down	A2M	up
CTD-2033D15.1	down	ASB8	up	RP11-91P24.7	down	ADAM10	up
CTD-2033D15.1	down	ASB8	up	RP11-91P24.7	down	ASB8	up
CTD-2033D15.1	down	C1orf229	up	RP11-91P24.7	down	ASB8	up
CTD-2033D15.1	down	C1QA	up	RP11-91P24.7	down	BLOC1S6	up

RP11-480I12.7	down	RPL27A	up
RP11-603J24.14	down	RPL27A	up
RP11-705C15.5	down	RPL27A	up
**hsa-miR-22-3p**	**down**	**RPL27A**	**up**
**hsa-miR-4443**	**down**	**RPL27A**	**up**
**hsa-miR-6087**	**down**	**RPL27A**	**up**
**hsa-miR-6089**	**down**	**RPL27A**	**up**
AC007228.9	up	RRAD	down
AC009501.4	up	RRAD	down
AC009501.4	up	RRAD	down
AC011239.1	up	RRAD	down
AC011239.1	up	RRAD	down
AC064871.3	up	RRAD	down
AC064871.3	up	RRAD	down
AC073254.1	up	RRAD	down
AC073254.1	up	RRAD	down
AC073254.1	up	RRAD	down
AC073254.1	up	RRAD	down
AC079586.1	up	RRAD	down
AC079586.1	up	RRAD	down
AC123886.2	up	RRAD	down
BCYRN1	up	RRAD	down
BCYRN1	up	RRAD	down
CTC-297N7.7	up	RRAD	down
CTC-297N7.7	up	RRAD	down
CTC-297N7.7	up	RRAD	down
CTC-297N7.7	up	RRAD	down
CTC-297N7.7	up	RRAD	down
CTC-297N7.7	up	RRAD	down
CTC-297N7.7	up	RRAD	down
CTC-297N7.7	up	RRAD	down
CTC-537E7.2	up	RRAD	down
CTC-537E7.2	up	RRAD	down
MYHAS	up	RRAD	down
MYHAS	up	RRAD	down
RNU12	up	RRAD	down
RNU12	up	RRAD	down
RP11-357D18.1	up	RRAD	down
RP11-357D18.1	up	RRAD	down
RP11-386J22.3	up	RRAD	down
RP11-442H21.2	up	RRAD	down
RP11-442H21.2	up	RRAD	down
RP11-731F5.2	up	RRAD	down
RP11-731F5.2	up	RRAD	down
SNHG1	up	RRAD	down
SNHG1	up	RRAD	down
SNHG1	up	RRAD	down
SNHG1	up	RRAD	down
SNHG12	up	RRAD	down
SNHG12	up	RRAD	down
SNHG12	up	RRAD	down

CTD-2033D15.1	down	C1QA	up	RP11-91P24.7	down	C1orf229	up
CTD-2033D15.1	down	C1QA	up	RP11-91P24.7	down	C1QA	up
CTD-2033D15.1	down	C3	up	RP11-91P24.7	down	C1QA	up
CTD-2033D15.1	down	CEBPZOS	up	RP11-91P24.7	down	C1QA	up
CTD-2033D15.1	down	CEBPZOS	up	RP11-91P24.7	down	C3	up
CTD-2033D15.1	down	CHRNA1	up	RP11-91P24.7	down	CEBPZOS	up
CTD-2033D15.1	down	CHRNA1	up	RP11-91P24.7	down	CEBPZOS	up
CTD-2033D15.1	down	CHRNA1	up	RP11-91P24.7	down	CHRNA1	up
CTD-2033D15.1	down	CHRNA1	up	RP11-91P24.7	down	CHRNA1	up
CTD-2033D15.1	down	CHRNA1	up	RP11-91P24.7	down	CHRNA1	up
CTD-2033D15.1	down	COL1A2	up	RP11-91P24.7	down	CHRNA1	up
CTD-2033D15.1	down	COL1A2	up	RP11-91P24.7	down	CHRNA1	up
CTD-2033D15.1	down	CTSK	up	RP11-91P24.7	down	CHRNA1	up
CTD-2033D15.1	down	CTSK	up	RP11-91P24.7	down	COL1A2	up
CTD-2033D15.1	down	DDIT4	up	RP11-91P24.7	down	COL1A2	up
CTD-2033D15.1	down	DNMT3A	up	RP11-91P24.7	down	CTSK	up
CTD-2033D15.1	down	EEF1A1	up	RP11-91P24.7	down	CTSK	up
CTD-2033D15.1	down	EEF1A1	up	RP11-91P24.7	down	DDIT4	up
CTD-2033D15.1	down	EEF1A1	up	RP11-91P24.7	down	DNMT3A	up
CTD-2033D15.1	down	EEF1A1	up	RP11-91P24.7	down	EEF1A1	up
CTD-2033D15.1	down	FAM129A	up	RP11-91P24.7	down	EEF1A1	up
CTD-2033D15.1	down	GSG2	up	RP11-91P24.7	down	EEF1A1	up
CTD-2033D15.1	down	HBA1	up	RP11-91P24.7	down	EEF1A1	up
CTD-2033D15.1	down	HBA1	up	RP11-91P24.7	down	EEF1A1	up
CTD-2033D15.1	down	HBA2	up	RP11-91P24.7	down	EIF4A2	up
CTD-2033D15.1	down	HBA2	up	RP11-91P24.7	down	EIF4A2	up
CTD-2033D15.1	down	HBB	up	RP11-91P24.7	down	EIF4A2	up
CTD-2033D15.1	down	HBD	up	RP11-91P24.7	down	EIF4A2	up
CTD-2033D15.1	down	HBD	up	RP11-91P24.7	down	EIF4A2	up
CTD-2033D15.1	down	HBD	up	RP11-91P24.7	down	EIF4A2	up
CTD-2033D15.1	down	HIST1H4J	up	RP11-91P24.7	down	EIF4A2	up
CTD-2033D15.1	down	HIST1H4K	up	RP11-91P24.7	down	FAM129A	up
CTD-2033D15.1	down	HTRA1	up	RP11-91P24.7	down	GREM1	up
CTD-2033D15.1	down	IGFBP4	up	RP11-91P24.7	down	GSG2	up
CTD-2033D15.1	down	IGFBP7	up	RP11-91P24.7	down	HBA1	up
CTD-2033D15.1	down	IGKC	up	RP11-91P24.7	down	HBA2	up
CTD-2033D15.1	down	IGKC	up	RP11-91P24.7	down	HBA2	up
CTD-2033D15.1	down	IGKC	up	RP11-91P24.7	down	HIST1H4J	up
CTD-2033D15.1	down	IGKC	up	RP11-91P24.7	down	HIST1H4K	up
CTD-2033D15.1	down	IMPA2	up	RP11-91P24.7	down	HTRA1	up
CTD-2033D15.1	down	IMPA2	up	RP11-91P24.7	down	HTRA1	up
CTD-2033D15.1	down	KIAA1328	up	RP11-91P24.7	down	MGP	up
CTD-2033D15.1	down	LAMC1	up	RP11-91P24.7	down	MKNK2	up
CTD-2033D15.1	down	LUM	up	RP11-91P24.7	down	MKNK2	up
CTD-2033D15.1	down	MKNK2	up	RP11-91P24.7	down	MKNK2	up
CTD-2033D15.1	down	MKNK2	up	RP11-91P24.7	down	MKNK2	up
CTD-2033D15.1	down	MKNK2	up	RP11-91P24.7	down	MKNK2	up
CTD-2033D15.1	down	MKNK2	up	RP11-91P24.7	down	MKNK2	up
CTD-2033D15.1	down	MKNK2	up	RP11-91P24.7	down	MKNK2	up
CTD-2033D15.1	down	MKNK2	up	RP11-91P24.7	down	MMP14	up
CTD-2033D15.1	down	MKNK2	up	RP11-91P24.7	down	MORC4	up

SNHG12	up	RRAD	down
SNHG12	up	RRAD	down
SNHG12	up	RRAD	down
SNHG12	up	RRAD	down
SNHG12	up	RRAD	down
SNORA76C	up	RRAD	down
SNORA76C	up	RRAD	down
**hsa-miR-125a-5p**	**up**	**RRAD**	**down**
**hsa-miR-486-3p**	**up**	**RRAD**	**down**
AC007228.9	up	SERPINE1	down
AC009501.4	up	SERPINE1	down
AC011239.1	up	SERPINE1	down
AC064871.3	up	SERPINE1	down
AC073254.1	up	SERPINE1	down
AC079586.1	up	SERPINE1	down
AC123886.2	up	SERPINE1	down
BCYRN1	up	SERPINE1	down
CTC-260E6.6	up	SERPINE1	down
CTC-297N7.7	up	SERPINE1	down
CTC-297N7.7	up	SERPINE1	down
CTC-297N7.7	up	SERPINE1	down
CTC-297N7.7	up	SERPINE1	down
MYHAS	up	SERPINE1	down
RNU12	up	SERPINE1	down
RP11-357D18.1	up	SERPINE1	down
RP11-386J22.3	up	SERPINE1	down
RP11-521L9.1	up	SERPINE1	down
RP11-731F5.2	up	SERPINE1	down
SNHG1	up	SERPINE1	down
SNHG1	up	SERPINE1	down
SNHG12	up	SERPINE1	down
SNHG12	up	SERPINE1	down
SNHG12	up	SERPINE1	down
SNHG8	up	SERPINE1	down
SNHG9	up	SERPINE1	down
**hsa-miR-145-5p**	**up**	**SERPINE1**	**down**
**hsa-miR-23c**	**up**	**SERPINE1**	**down**
**hsa-miR-30c-5p**	**up**	**SERPINE1**	**down**
**hsa-miR-486-5p**	**up**	**SERPINE1**	**down**
AC007228.9	up	SOCS3	down
AC009501.4	up	SOCS3	down
AC011239.1	up	SOCS3	down
AC064871.3	up	SOCS3	down
AC073254.1	up	SOCS3	down
CTA-14H9.5	up	SOCS3	down
RNU12	up	SOCS3	down
RP11-357D18.1	up	SOCS3	down
RP11-357D18.1	up	SOCS3	down
RP11-386J22.3	up	SOCS3	down
RP11-403P17.4	up	SOCS3	down
RP11-731F5.2	up	SOCS3	down

CTD-2033D15.1	down	MMP14	up	RP11-91P24.7	down	MSS51	up
CTD-2033D15.1	down	MORC4	up	RP11-91P24.7	down	MSS51	up
CTD-2033D15.1	down	MSS51	up	RP11-91P24.7	down	MSS51	up
CTD-2033D15.1	down	MSS51	up	RP11-91P24.7	down	MYH1	up
CTD-2033D15.1	down	MYH1	up	RP11-91P24.7	down	MYH4	up
CTD-2033D15.1	down	MYH4	up	RP11-91P24.7	down	MYOC	up
CTD-2033D15.1	down	MYOC	up	RP11-91P24.7	down	MYOC	up
CTD-2033D15.1	down	MYOC	up	RP11-91P24.7	down	MYOG	up
CTD-2033D15.1	down	MYOG	up	RP11-91P24.7	down	PRRG3	up
CTD-2033D15.1	down	PRRG3	up	RP11-91P24.7	down	RNASE1	up
CTD-2033D15.1	down	RNASE1	up	RP11-91P24.7	down	RNASE1	up
CTD-2033D15.1	down	RNASE1	up	RP11-91P24.7	down	RNASE1	up
CTD-2033D15.1	down	RNASE1	up	RP11-91P24.7	down	RNASE1	up
CTD-2033D15.1	down	RNASE1	up	RP11-91P24.7	down	SPARC	up
CTD-2033D15.1	down	RPS3	up	RP11-91P24.7	down	SPARC	up
CTD-2033D15.1	down	SPARC	up	RP11-91P24.7	down	SPARC	up
CTD-2033D15.1	down	SPARC	up	RP11-91P24.7	down	SPARC	up
CTD-2033D15.1	down	STAG2	up	RP11-91P24.7	down	SPARC	up
CTD-2033D15.1	down	STAG2	up	RP11-91P24.7	down	SPARC	up
CTD-2033D15.1	down	TBC1D8B	up	RP11-91P24.7	down	STAG2	up
CTD-2033D15.1	down	TMEM107	up	RP11-91P24.7	down	STAG2	up
CTD-2033D15.1	down	TMSB4X	up	RP11-91P24.7	down	TBC1D8B	up
CTD-2033D15.1	down	TMSB4X	up	RP11-91P24.7	down	TMEM107	up
CTD-2033D15.1	down	TMSB4X	up	RP11-91P24.7	down	TMSB4X	up
CTD-2033D15.1	down	TPM4	up	RP11-91P24.7	down	TPM4	up
CTD-2033D15.1	down	TRIM22	up	RP11-91P24.7	down	TPM4	up
CTD-2033D15.1	down	TRIM22	up	RP11-91P24.7	down	TUBD1	up
CTD-2033D15.1	down	TRIM22	up	RP11-91P24.7	down	TUBD1	up
CTD-2033D15.1	down	TRIM22	up	RP11-91P24.7	down	TUBD1	up
CTD-2033D15.1	down	TRIM22	up	RP11-91P24.7	down	TUBD1	up
CTD-2033D15.1	down	TUBD1	up	RP11-91P24.7	down	TUBD1	up
CTD-2033D15.1	down	TUBD1	up	RP11-91P24.7	down	ZNF302	up
CTD-2033D15.1	down	TUBD1	up	RP11-91P24.7	down	ZNF302	up
CTD-2033D15.1	down	TUBD1	up	SNHG1	up	ACTA1	down
CTD-2033D15.1	down	TUBD1	up	SNHG1	up	ACTA1	down
CTD-2033D15.1	down	UCP3	up	SNHG1	up	ANKRD1	down
CTD-2033D15.1	down	UCP3	up	SNHG1	up	ANKRD2	down
CTD-2033D15.1	down	UCP3	up	SNHG1	up	ANKRD2	down
CTD-2033D15.1	down	ZNF302	up	SNHG1	up	ANKRD2	down
CTD-2033D15.1	down	ZNF302	up	SNHG1	up	BHLHE40	down
CTD-2033D15.1	down	ZNF354B	up	SNHG1	up	C10orf10	down
CTD-2033D15.1	down	A2M	up	SNHG1	up	CCL2	down
CTD-2033D15.1	down	A2M	up	SNHG1	up	CCL8	down
CTD-2033D15.1	down	A2M	up	SNHG1	up	CDKN1A	down
CTD-2033D15.1	down	ASB8	up	SNHG1	up	CDKN1A	down
CTD-2033D15.1	down	ASB8	up	SNHG1	up	CDKN1A	down
CTD-2033D15.1	down	C1orf229	up	SNHG1	up	CDKN1A	down
CTD-2033D15.1	down	C1QA	up	SNHG1	up	CDKN1A	down
CTD-2033D15.1	down	C3	up	SNHG1	up	CKMT2	down
CTD-2033D15.1	down	C3	up	SNHG1	up	CKMT2	down
CTD-2033D15.1	down	CEBPZOS	up	SNHG1	up	CKMT2	down

SNHG1	up	SOCS3	down
SNHG1	up	SOCS3	down
SNHG12	up	SOCS3	down
SNHG12	up	SOCS3	down
SNHG12	up	SOCS3	down
SNHG8	up	SOCS3	down
SNHG9	up	SOCS3	down
**hsa-miR-3197**	**up**	**SOCS3**	**down**
**hsa-miR-4459**	**up**	**SOCS3**	**down**
CTD-2033D15.1	down	SPARC	up
CTD-2033D15.1	down	SPARC	up
EAF1-AS1	down	SPARC	up
LINC01405	down	SPARC	up
LINC01405	down	SPARC	up
LINC01405	down	SPARC	up
LINC01405	down	SPARC	up
LINC01405	down	SPARC	up
LINC01405	down	SPARC	up
LINC01405	down	SPARC	up
LINC01405	down	SPARC	up
LINC01405	down	SPARC	up
LINC01405	down	SPARC	up
RP11-451G4.2	down	SPARC	up
RP11-451G4.2	down	SPARC	up
RP11-451G4.2	down	SPARC	up
RP11-480I12.7	down	SPARC	up
RP11-480I12.7	down	SPARC	up
RP11-480I12.7	down	SPARC	up
RP11-480I12.7	down	SPARC	up
RP11-480I12.7	down	SPARC	up
RP11-603J24.14	down	SPARC	up
RP11-603J24.14	down	SPARC	up
RP11-603J24.14	down	SPARC	up
RP11-603J24.14	down	SPARC	up
RP11-603J24.14	down	SPARC	up
RP11-603J24.14	down	SPARC	up
RP11-705C15.5	down	SPARC	up
RP11-705C15.5	down	SPARC	up
RP11-705C15.5	down	SPARC	up
RP11-705C15.5	down	SPARC	up
RP11-91P24.7	down	SPARC	up
RP11-91P24.7	down	SPARC	up
RP11-91P24.7	down	SPARC	up
RP11-91P24.7	down	SPARC	up
RP11-91P24.7	down	SPARC	up
RP11-91P24.7	down	SPARC	up
**hsa-miR-378c**	**down**	**SPARC**	**up**
**hsa-miR-378g**	**down**	**SPARC**	**up**
AC007228.9	up	SRGN	down
AC009501.4	up	SRGN	down
AC011239.1	up	SRGN	down

CTD-2033D15.1	down	COL1A2	up	SNHG1	up	CKMT2	down
CTD-2033D15.1	down	COL1A2	up	SNHG1	up	CTSL	down
CTD-2033D15.1	down	DDIT4	up	SNHG1	up	CTSL	down
CTD-2033D15.1	down	DNMT3A	up	SNHG1	up	CTSL	down
CTD-2033D15.1	down	EEF1A1	up	SNHG1	up	GADD45G	down
CTD-2033D15.1	down	EEF1A1	up	SNHG1	up	GADD45G	down
CTD-2033D15.1	down	EEF1A1	up	SNHG1	up	HMOX1	down
CTD-2033D15.1	down	EEF1A1	up	SNHG1	up	HMOX1	down
CTD-2033D15.1	down	EEF1A1	up	SNHG1	up	MYL2	down
CTD-2033D15.1	down	EEF1A1	up	SNHG1	up	MYL2	down
CTD-2033D15.1	down	EIF4A2	up	SNHG1	up	MYL6B	down
CTD-2033D15.1	down	FAM129A	up	SNHG1	up	MYL6B	down
CTD-2033D15.1	down	GSG2	up	SNHG1	up	MYL6B	down
CTD-2033D15.1	down	HBA1	up	SNHG1	up	MYOM3	down
CTD-2033D15.1	down	HBA1	up	SNHG1	up	PDLIM1	down
CTD-2033D15.1	down	HBA2	up	SNHG1	up	RRAD	down
CTD-2033D15.1	down	HBA2	up	SNHG1	up	RRAD	down
CTD-2033D15.1	down	HBB	up	SNHG1	up	SERPINE1	down
CTD-2033D15.1	down	HBD	up	SNHG1	up	SOCS3	down
CTD-2033D15.1	down	HBD	up	SNHG1	up	TAP1	down
CTD-2033D15.1	down	HBD	up	SNHG1	up	THBS1	down
CTD-2033D15.1	down	HIST1H4J	up	SNHG1	up	THBS1	down
CTD-2033D15.1	down	HIST1H4K	up	SNHG1	up	TNFRSF12A	down
CTD-2033D15.1	down	HTRA1	up	SNHG1	up	TNFRSF12A	down
CTD-2033D15.1	down	HTRA1	up	SNHG1	up	TNFRSF12A	down
CTD-2033D15.1	down	IGFBP4	up	SNHG1	up	TNFRSF12A	down
CTD-2033D15.1	down	IGKC	up	SNHG1	up	TNFRSF12A	down
CTD-2033D15.1	down	IGKC	up	SNHG1	up	TNNC1	down
CTD-2033D15.1	down	IGKC	up	SNHG1	up	TNNC1	down
CTD-2033D15.1	down	IGKC	up	SNHG1	up	TNNI1	down
CTD-2033D15.1	down	IMPA2	up	SNHG1	up	TNNI1	down
CTD-2033D15.1	down	IMPA2	up	SNHG1	up	TNNI1	down
CTD-2033D15.1	down	KIAA1328	up	SNHG1	up	TNNI1	down
CTD-2033D15.1	down	LAMC1	up	SNHG1	up	TNNI1	down
CTD-2033D15.1	down	MKNK2	up	SNHG1	up	TPM3	down
CTD-2033D15.1	down	MKNK2	up	SNHG1	up	ACTA1	down
CTD-2033D15.1	down	MORC4	up	SNHG1	up	ACTA1	down
CTD-2033D15.1	down	MSS51	up	SNHG1	up	ANKRD1	down
CTD-2033D15.1	down	MSS51	up	SNHG1	up	ANKRD2	down
CTD-2033D15.1	down	MSS51	up	SNHG1	up	ANKRD2	down
CTD-2033D15.1	down	MYH1	up	SNHG1	up	ANKRD2	down
CTD-2033D15.1	down	MYH4	up	SNHG1	up	BHLHE40	down
CTD-2033D15.1	down	MYOC	up	SNHG1	up	C10orf10	down
CTD-2033D15.1	down	MYOC	up	SNHG1	up	CCL2	down
CTD-2033D15.1	down	MYOG	up	SNHG1	up	CCL8	down
CTD-2033D15.1	down	PRRG3	up	SNHG1	up	CDKN1A	down
CTD-2033D15.1	down	RNASE1	up	SNHG1	up	CDKN1A	down
CTD-2033D15.1	down	RNASE1	up	SNHG1	up	CDKN1A	down
CTD-2033D15.1	down	RNASE1	up	SNHG1	up	CDKN1A	down
CTD-2033D15.1	down	RPL27A	up	SNHG1	up	CDKN1A	down
CTD-2033D15.1	down	STAG2	up	SNHG1	up	CKMT2	down

AC073254.1	up	SRGN	down
AC073254.1	up	SRGN	down
AC079586.1	up	SRGN	down
AC123886.2	up	SRGN	down
BCYRN1	up	SRGN	down
CTC-297N7.7	up	SRGN	down
CTC-297N7.7	up	SRGN	down
CTC-297N7.7	up	SRGN	down
RP11-357D18.1	up	SRGN	down
RP11-357D18.1	up	SRGN	down
RP11-386J22.3	up	SRGN	down
RP11-403P17.4	up	SRGN	down
RP11-521L9.1	up	SRGN	down
RP11-731F5.2	up	SRGN	down
SNHG12	up	SRGN	down
SNHG12	up	SRGN	down
SNHG12	up	SRGN	down
SNHG12	up	SRGN	down
SNHG12	up	SRGN	down
SNHG8	up	SRGN	down
SNHG9	up	SRGN	down
SNORA76C	up	SRGN	down
**hsa-let-7a-5p**	**up**	**SRGN**	**down**
**hsa-let-7f-5p**	**up**	**SRGN**	**down**
CTD-2033D15.1	down	TBC1D8B	up
CTD-2033D15.1	down	TBC1D8B	up
EAF1-AS1	down	TBC1D8B	up
LINC01405	down	TBC1D8B	up
LINC01405	down	TBC1D8B	up
RP11-451G4.2	down	TBC1D8B	up
RP11-480I12.7	down	TBC1D8B	up
RP11-603J24.14	down	TBC1D8B	up
RP11-91P24.7	down	TBC1D8B	up
**hsa-miR-1-3p**	**down**	**TBC1D8B**	**up**
AC007228.9	up	THBS1	down
AC007228.9	up	THBS1	down
AC009501.4	up	THBS1	down
AC011239.1	up	THBS1	down
AC011239.1	up	THBS1	down
AC073254.1	up	THBS1	down
AC073254.1	up	THBS1	down
AC073254.1	up	THBS1	down
AC079586.1	up	THBS1	down
AC079586.1	up	THBS1	down
AC123886.2	up	THBS1	down
AC123886.2	up	THBS1	down
BCYRN1	up	THBS1	down
BCYRN1	up	THBS1	down
CTA-14H9.5	up	THBS1	down
CTA-14H9.5	up	THBS1	down
CTC-297N7.7	up	THBS1	down

CTD-2033D15.1	down	STAG2	up
CTD-2033D15.1	down	TBC1D8B	up
CTD-2033D15.1	down	TMEM107	up
CTD-2033D15.1	down	TMSB4X	up
CTD-2033D15.1	down	TPM4	up
CTD-2033D15.1	down	TPM4	up
CTD-2033D15.1	down	TRIM22	up
CTD-2033D15.1	down	TRIM22	up
CTD-2033D15.1	down	TRIM22	up
CTD-2033D15.1	down	TRIM22	up
CTD-2033D15.1	down	TRIM22	up
CTD-2033D15.1	down	UCP3	up
CTD-2033D15.1	down	UCP3	up
CTD-2033D15.1	down	UCP3	up
CTD-2033D15.1	down	ZNF302	up
CTD-2033D15.1	down	ZNF302	up
CTD-2033D15.1	down	ZNF354B	up
EAF1-AS1	down	A2M	up
EAF1-AS1	down	A2M	up
EAF1-AS1	down	ADAM10	up
EAF1-AS1	down	ASB8	up
EAF1-AS1	down	ASB8	up
EAF1-AS1	down	BLOC1S6	up
EAF1-AS1	down	C1orf229	up
EAF1-AS1	down	C1QA	up
EAF1-AS1	down	C1QA	up
EAF1-AS1	down	C1QA	up
EAF1-AS1	down	C3	up
EAF1-AS1	down	CEBPZOS	up
EAF1-AS1	down	CEBPZOS	up
EAF1-AS1	down	COL1A2	up
EAF1-AS1	down	COL1A2	up
EAF1-AS1	down	DDIT4	up
EAF1-AS1	down	DNMT3A	up
EAF1-AS1	down	EEF1A1	up
EAF1-AS1	down	EEF1A1	up
EAF1-AS1	down	EEF1A1	up
EAF1-AS1	down	FAM129A	up
EAF1-AS1	down	GREM1	up
EAF1-AS1	down	HBB	up
EAF1-AS1	down	HBD	up
EAF1-AS1	down	HBD	up
EAF1-AS1	down	HBD	up
EAF1-AS1	down	HTRA1	up
EAF1-AS1	down	HTRA1	up
EAF1-AS1	down	IGFBP4	up
EAF1-AS1	down	IGFBP7	up
EAF1-AS1	down	IMPA2	up
EAF1-AS1	down	IMPA2	up
EAF1-AS1	down	LAMC1	up
EAF1-AS1	down	MKNK2	up

SNHG1	up	CKMT2	down
SNHG1	up	CKMT2	down
SNHG1	up	CKMT2	down
SNHG1	up	CTSL	down
SNHG1	up	CTSL	down
SNHG1	up	CTSL	down
SNHG1	up	GADD45G	down
SNHG1	up	HMOX1	down
SNHG1	up	HMOX1	down
SNHG1	up	MYL2	down
SNHG1	up	MYL2	down
SNHG1	up	MYL6B	down
SNHG1	up	MYL6B	down
SNHG1	up	MYL6B	down
SNHG1	up	MYOM3	down
SNHG1	up	PDLIM1	down
SNHG1	up	RRAD	down
SNHG1	up	RRAD	down
SNHG1	up	SERPINE1	down
SNHG1	up	SOCS3	down
SNHG1	up	TAP1	down
SNHG1	up	THBS1	down
SNHG1	up	THBS1	down
SNHG1	up	TNFRSF12A	down
SNHG1	up	TNFRSF12A	down
SNHG1	up	TNFRSF12A	down
SNHG1	up	TNFRSF12A	down
SNHG1	up	TNFRSF12A	down
SNHG1	up	TNNC1	down
SNHG1	up	TNNC1	down
SNHG1	up	TNNI1	down
SNHG1	up	TNNI1	down
SNHG1	up	TNNI1	down
SNHG1	up	TNNI1	down
SNHG1	up	TNNI1	down
SNHG1	up	TNNI1	down
SNHG1	up	TPM3	down
SNHG12	up	ANKRD1	down
SNHG12	up	ANKRD2	down
SNHG12	up	ANKRD2	down
SNHG12	up	ANKRD2	down
SNHG12	up	C10orf10	down
SNHG12	up	CCL2	down
SNHG12	up	CCL8	down
SNHG12	up	CDKN1A	down
SNHG12	up	CDKN1A	down
SNHG12	up	CDKN1A	down
SNHG12	up	CDKN1A	down
SNHG12	up	CDKN1A	down
SNHG12	up	CKMT2	down
SNHG12	up	CKMT2	down

CTC-297N7.7	up	THBS1	down
CTC-297N7.7	up	THBS1	down
CTC-297N7.7	up	THBS1	down
CTC-297N7.7	up	THBS1	down
CTC-297N7.7	up	THBS1	down
CTC-537E7.2	up	THBS1	down
CTC-537E7.2	up	THBS1	down
MYHAS	up	THBS1	down
MYHAS	up	THBS1	down
RNU12	up	THBS1	down
RNU12	up	THBS1	down
RP11-386J22.3	up	THBS1	down
RP11-386J22.3	up	THBS1	down
RP11-403P17.4	up	THBS1	down
RP11-442H21.2	up	THBS1	down
RP11-442H21.2	up	THBS1	down
RP11-521L9.1	up	THBS1	down
RP11-521L9.1	up	THBS1	down
RP11-731F5.2	up	THBS1	down
RP11-731F5.2	up	THBS1	down
SNHG1	up	THBS1	down
SNHG1	up	THBS1	down
SNHG1	up	THBS1	down
SNHG1	up	THBS1	down
SNHG12	up	THBS1	down
SNHG12	up	THBS1	down
SNHG12	up	THBS1	down
SNHG12	up	THBS1	down
SNHG12	up	THBS1	down
SNHG12	up	THBS1	down
SNHG12	up	THBS1	down
SNHG12	up	THBS1	down
SNHG8	up	THBS1	down
SNHG8	up	THBS1	down
SNHG9	up	THBS1	down
SNORA76C	up	THBS1	down
SNORA76C	up	THBS1	down
**hsa-let-7a-5p**	**up**	**THBS1**	**down**
**hsa-let-7b-5p**	**up**	**THBS1**	**down**
**hsa-let-7c-5p**	**up**	**THBS1**	**down**
**hsa-let-7f-5p**	**up**	**THBS1**	**down**
**hsa-let-7i-5p**	**up**	**THBS1**	**down**
**hsa-miR-206**	**up**	**THBS1**	**down**
AC007228.9	up	TNNI1	down
AC007228.9	up	TNNI1	down
AC007228.9	up	TNNI1	down
AC007228.9	up	TNNI1	down
AC007228.9	up	TNNI1	down
AC007228.9	up	TNNI1	down
AC009501.4	up	TNNI1	down
AC009501.4	up	TNNI1	down

EAF1-AS1	down	MKNK2	up
EAF1-AS1	down	MKNK2	up
EAF1-AS1	down	MKNK2	up
EAF1-AS1	down	MKNK2	up
EAF1-AS1	down	MKNK2	up
EAF1-AS1	down	MKNK2	up
EAF1-AS1	down	MMP14	up
EAF1-AS1	down	MYH1	up
EAF1-AS1	down	MYH4	up
EAF1-AS1	down	MYOC	up
EAF1-AS1	down	MYOC	up
EAF1-AS1	down	PRRG3	up
EAF1-AS1	down	RNASE1	up
EAF1-AS1	down	RNASE1	up
EAF1-AS1	down	RNASE1	up
EAF1-AS1	down	RPL27A	up
EAF1-AS1	down	SPARC	up
EAF1-AS1	down	STAG2	up
EAF1-AS1	down	STAG2	up
EAF1-AS1	down	TBC1D8B	up
EAF1-AS1	down	TMSB4X	up
EAF1-AS1	down	TMSB4X	up
EAF1-AS1	down	TPM4	up
EAF1-AS1	down	TRIM22	up
EAF1-AS1	down	TRIM22	up
EAF1-AS1	down	TRIM22	up
EAF1-AS1	down	UCP3	up
EAF1-AS1	down	ZNF302	up
LINC01405	down	ADAM10	up
LINC01405	down	BLOC1S6	up
LINC01405	down	C1orf229	up
LINC01405	down	C1QA	up
LINC01405	down	C1QA	up
LINC01405	down	C1QA	up
LINC01405	down	C3	up
LINC01405	down	CEBPZOS	up
LINC01405	down	CEBPZOS	up
LINC01405	down	CHRNA1	up
LINC01405	down	CHRNA1	up
LINC01405	down	CHRNA1	up
LINC01405	down	CHRNA1	up
LINC01405	down	CHRNA1	up
LINC01405	down	CHRNA1	up
LINC01405	down	COL1A2	up
LINC01405	down	COL1A2	up
LINC01405	down	DDIT4	up
LINC01405	down	DNMT3A	up
LINC01405	down	EEF1A1	up
LINC01405	down	EEF1A1	up
LINC01405	down	EEF1A1	up
LINC01405	down	EEF1A1	up

SNHG12	up	CKMT2	down
SNHG12	up	CKMT2	down
SNHG12	up	CSRP3	down
SNHG12	up	CSRP3	down
SNHG12	up	CTSL	down
SNHG12	up	CTSL	down
SNHG12	up	CTSL	down
SNHG12	up	GADD45G	down
SNHG12	up	HMOX1	down
SNHG12	up	HMOX1	down
SNHG12	up	MYL2	down
SNHG12	up	MYL2	down
SNHG12	up	MYL6B	down
SNHG12	up	MYL6B	down
SNHG12	up	MYL6B	down
SNHG12	up	PDLIM1	down
SNHG12	up	RRAD	down
SNHG12	up	RRAD	down
SNHG12	up	SAT1	down
SNHG12	up	SAT1	down
SNHG12	up	SAT1	down
SNHG12	up	SRGN	down
SNHG12	up	TAP1	down
SNHG12	up	THBS1	down
SNHG12	up	THBS1	down
SNHG12	up	TNFRSF12A	down
SNHG12	up	TNFRSF12A	down
SNHG12	up	TNFRSF12A	down
SNHG12	up	TNFRSF12A	down
SNHG12	up	TNNC1	down
SNHG12	up	ANKRD1	down
SNHG12	up	BHLHE40	down
SNHG12	up	C10orf10	down
SNHG12	up	CCL8	down
SNHG12	up	CDKN1A	down
SNHG12	up	CDKN1A	down
SNHG12	up	CKMT2	down
SNHG12	up	CKMT2	down
SNHG12	up	CKMT2	down
SNHG12	up	CRYAB	down
SNHG12	up	CTSL	down
SNHG12	up	CTSL	down
SNHG12	up	CTSL	down
SNHG12	up	GADD45G	down
SNHG12	up	HMOX1	down
SNHG12	up	HMOX1	down
SNHG12	up	MYL2	down
SNHG12	up	MYL2	down
SNHG12	up	MYL6B	down
SNHG12	up	MYL6B	down
SNHG12	up	NNMT	down

AC009501.4	up	TNNI1	down
AC011239.1	up	TNNI1	down
AC011239.1	up	TNNI1	down
AC011239.1	up	TNNI1	down
AC011239.1	up	TNNI1	down
AC011239.1	up	TNNI1	down
AC064871.3	up	TNNI1	down
AC064871.3	up	TNNI1	down
AC064871.3	up	TNNI1	down
AC064871.3	up	TNNI1	down
AC064871.3	up	TNNI1	down
AC064871.3	up	TNNI1	down
AC073254.1	up	TNNI1	down
AC073254.1	up	TNNI1	down
AC073254.1	up	TNNI1	down
AC073254.1	up	TNNI1	down
AC073254.1	up	TNNI1	down
AC073254.1	up	TNNI1	down
AC073254.1	up	TNNI1	down
AC073254.1	up	TNNI1	down
AC073254.1	up	TNNI1	down
AC073254.1	up	TNNI1	down
AC073254.1	up	TNNI1	down
AC079586.1	up	TNNI1	down
AC079586.1	up	TNNI1	down
AC079586.1	up	TNNI1	down
AC123886.2	up	TNNI1	down
AC123886.2	up	TNNI1	down
BCYRN1	up	TNNI1	down
BCYRN1	up	TNNI1	down
BCYRN1	up	TNNI1	down
BCYRN1	up	TNNI1	down
BCYRN1	up	TNNI1	down
CTA-14H9.5	up	TNNI1	down
CTA-14H9.5	up	TNNI1	down
CTA-14H9.5	up	TNNI1	down
CTA-14H9.5	up	TNNI1	down
CTA-14H9.5	up	TNNI1	down
CTC-260E6.6	up	TNNI1	down
CTC-260E6.6	up	TNNI1	down
CTC-260E6.6	up	TNNI1	down
CTC-260E6.6	up	TNNI1	down
CTC-260E6.6	up	TNNI1	down
CTC-260E6.6	up	TNNI1	down
CTC-297N7.7	up	TNNI1	down
CTC-297N7.7	up	TNNI1	down
CTC-297N7.7	up	TNNI1	down
CTC-297N7.7	up	TNNI1	down
CTC-297N7.7	up	TNNI1	down
CTC-297N7.7	up	TNNI1	down
CTC-297N7.7	up	TNNI1	down

LINC01405	down	EEF1A1	up	SNHG12	up	NNMT	down
LINC01405	down	EEF1A1	up	SNHG12	up	NNMT	down
LINC01405	down	EEF1A1	up	SNHG12	up	NNMT	down
LINC01405	down	EIF4A2	up	SNHG12	up	PDLIM1	down
LINC01405	down	EIF4A2	up	SNHG12	up	SAT1	down
LINC01405	down	EIF4A2	up	SNHG12	up	SAT1	down
LINC01405	down	EIF4A2	up	SNHG12	up	SAT1	down
LINC01405	down	EIF4A2	up	SNHG12	up	SOCS3	down
LINC01405	down	EIF4A2	up	SNHG12	up	SRGN	down
LINC01405	down	EIF4A2	up	SNHG12	up	TAP1	down
LINC01405	down	FAM129A	up	SNHG12	up	THBS1	down
LINC01405	down	GREM1	up	SNHG12	up	TNFRSF12A	down
LINC01405	down	GSG2	up	SNHG12	up	TNFRSF12A	down
LINC01405	down	HBA1	up	SNHG12	up	TNFRSF12A	down
LINC01405	down	HBA1	up	SNHG12	up	TNFRSF12A	down
LINC01405	down	HBA2	up	SNHG12	up	TNNC1	down
LINC01405	down	HBA2	up	SNHG12	up	TNNC1	down
LINC01405	down	HBB	up	SNHG12	up	ANKRD1	down
LINC01405	down	HBD	up	SNHG12	up	ANKRD2	down
LINC01405	down	HBD	up	SNHG12	up	ANKRD2	down
LINC01405	down	HBD	up	SNHG12	up	ANKRD2	down
LINC01405	down	HIST1H4J	up	SNHG12	up	ANKRD2	down
LINC01405	down	HIST1H4K	up	SNHG12	up	BHLHE40	down
LINC01405	down	HTRA1	up	SNHG12	up	CCL2	down
LINC01405	down	HTRA1	up	SNHG12	up	CCL8	down
LINC01405	down	IGFBP4	up	SNHG12	up	CDKN1A	down
LINC01405	down	IGFBP7	up	SNHG12	up	CDKN1A	down
LINC01405	down	IGKC	up	SNHG12	up	CKMT2	down
LINC01405	down	IGKC	up	SNHG12	up	CKMT2	down
LINC01405	down	IGKC	up	SNHG12	up	CKMT2	down
LINC01405	down	IGKC	up	SNHG12	up	CKMT2	down
LINC01405	down	IMPA2	up	SNHG12	up	CSRP3	down
LINC01405	down	IMPA2	up	SNHG12	up	CSRP3	down
LINC01405	down	KIAA1328	up	SNHG12	up	CTSL	down
LINC01405	down	LAMC1	up	SNHG12	up	CTSL	down
LINC01405	down	MKNK2	up	SNHG12	up	CTSL	down
LINC01405	down	MKNK2	up	SNHG12	up	CXCL10	down
LINC01405	down	MKNK2	up	SNHG12	up	GADD45G	down
LINC01405	down	MKNK2	up	SNHG12	up	HMOX1	down
LINC01405	down	MKNK2	up	SNHG12	up	HMOX1	down
LINC01405	down	MKNK2	up	SNHG12	up	MYL2	down
LINC01405	down	MKNK2	up	SNHG12	up	MYL2	down
LINC01405	down	MMP14	up	SNHG12	up	MYL6B	down
LINC01405	down	MORC4	up	SNHG12	up	MYL6B	down
LINC01405	down	MSS51	up	SNHG12	up	MYL6B	down
LINC01405	down	MSS51	up	SNHG12	up	NNMT	down
LINC01405	down	MSS51	up	SNHG12	up	NNMT	down
LINC01405	down	MYH1	up	SNHG12	up	NNMT	down
LINC01405	down	MYH4	up	SNHG12	up	NNMT	down
LINC01405	down	MYOG	up	SNHG12	up	PDLIM1	down
LINC01405	down	PRRG3	up	SNHG12	up	RRAD	down

CTC-297N7.7	up	TNNI1	down
CTC-297N7.7	up	TNNI1	down
CTC-297N7.7	up	TNNI1	down
CTC-297N7.7	up	TNNI1	down
CTC-297N7.7	up	TNNI1	down
CTC-297N7.7	up	TNNI1	down
CTC-297N7.7	up	TNNI1	down
CTC-297N7.7	up	TNNI1	down
CTC-297N7.7	up	TNNI1	down
CTC-297N7.7	up	TNNI1	down
CTC-297N7.7	up	TNNI1	down
CTC-537E7.2	up	TNNI1	down
CTC-537E7.2	up	TNNI1	down
MYHAS	up	TNNI1	down
MYHAS	up	TNNI1	down
MYHAS	up	TNNI1	down
MYHAS	up	TNNI1	down
RP11-386J22.3	up	TNNI1	down
RP11-386J22.3	up	TNNI1	down
RP11-386J22.3	up	TNNI1	down
RP11-386J22.3	up	TNNI1	down
RP11-386J22.3	up	TNNI1	down
RP11-403P17.4	up	TNNI1	down
RP11-403P17.4	up	TNNI1	down
RP11-403P17.4	up	TNNI1	down
RP11-403P17.4	up	TNNI1	down
RP11-403P17.4	up	TNNI1	down
RP11-403P17.4	up	TNNI1	down
RP11-442H21.2	up	TNNI1	down
RP11-442H21.2	up	TNNI1	down
RP11-442H21.2	up	TNNI1	down
RP11-442H21.2	up	TNNI1	down
RP11-442H21.2	up	TNNI1	down
RP11-521L9.1	up	TNNI1	down
RP11-521L9.1	up	TNNI1	down
RP11-521L9.1	up	TNNI1	down
RP11-521L9.1	up	TNNI1	down
RP11-731F5.2	up	TNNI1	down
RP11-731F5.2	up	TNNI1	down
RP11-731F5.2	up	TNNI1	down
RP11-731F5.2	up	TNNI1	down
RP11-731F5.2	up	TNNI1	down
RP11-731F5.2	up	TNNI1	down
SNHG1	up	TNNI1	down
SNHG1	up	TNNI1	down
SNHG1	up	TNNI1	down
SNHG1	up	TNNI1	down
SNHG1	up	TNNI1	down
SNHG1	up	TNNI1	down
SNHG1	up	TNNI1	down
SNHG1	up	TNNI1	down

LINC01405	down	RNASE1	up	SNHG12	up	RRAD	down
LINC01405	down	RNASE1	up	SNHG12	up	SAT1	down
LINC01405	down	RNASE1	up	SNHG12	up	SAT1	down
LINC01405	down	RNASE1	up	SNHG12	up	SAT1	down
LINC01405	down	RPS3	up	SNHG12	up	SERPINE1	down
LINC01405	down	SPARC	up	SNHG12	up	SOCS3	down
LINC01405	down	SPARC	up	SNHG12	up	SRGN	down
LINC01405	down	SPARC	up	SNHG12	up	TAP1	down
LINC01405	down	SPARC	up	SNHG12	up	THBS1	down
LINC01405	down	SPARC	up	SNHG12	up	TNFRSF12A	down
LINC01405	down	TBC1D8B	up	SNHG12	up	TNFRSF12A	down
LINC01405	down	TMEM107	up	SNHG12	up	TNFRSF12A	down
LINC01405	down	TMSB4X	up	SNHG12	up	TNFRSF12A	down
LINC01405	down	TMSB4X	up	SNHG12	up	TNFRSF12A	down
LINC01405	down	TMSB4X	up	SNHG12	up	TNNC1	down
LINC01405	down	TMSB4X	up	SNHG12	up	ANKRD1	down
LINC01405	down	TPM4	up	SNHG12	up	ANKRD2	down
LINC01405	down	TPM4	up	SNHG12	up	ANKRD2	down
LINC01405	down	TRIM22	up	SNHG12	up	ANKRD2	down
LINC01405	down	TRIM22	up	SNHG12	up	BHLHE40	down
LINC01405	down	TRIM22	up	SNHG12	up	C10orf10	down
LINC01405	down	TRIM22	up	SNHG12	up	CDKN1A	down
LINC01405	down	TRIM22	up	SNHG12	up	CDKN1A	down
LINC01405	down	TUBD1	up	SNHG12	up	CDKN1A	down
LINC01405	down	TUBD1	up	SNHG12	up	CDKN1A	down
LINC01405	down	TUBD1	up	SNHG12	up	CDKN1A	down
LINC01405	down	TUBD1	up	SNHG12	up	CKMT2	down
LINC01405	down	TUBD1	up	SNHG12	up	CTSL	down
LINC01405	down	UCP3	up	SNHG12	up	CTSL	down
LINC01405	down	UCP3	up	SNHG12	up	CTSL	down
LINC01405	down	UCP3	up	SNHG12	up	CXCL10	down
LINC01405	down	ZNF302	up	SNHG12	up	GADD45G	down
LINC01405	down	ZNF302	up	SNHG12	up	GADD45G	down
LINC01405	down	ZNF354B	up	SNHG12	up	HMOX1	down
LINC01405	down	ADAM10	up	SNHG12	up	HMOX1	down
LINC01405	down	BLOC1S6	up	SNHG12	up	MYL2	down
LINC01405	down	C1orf229	up	SNHG12	up	NNMT	down
LINC01405	down	C1QA	up	SNHG12	up	NNMT	down
LINC01405	down	C3	up	SNHG12	up	NNMT	down
LINC01405	down	CEBPZOS	up	SNHG12	up	NNMT	down
LINC01405	down	CEBPZOS	up	SNHG12	up	NNMT	down
LINC01405	down	CHRNA1	up	SNHG12	up	PDLIM1	down
LINC01405	down	CHRNA1	up	SNHG12	up	RRAD	down
LINC01405	down	CHRNA1	up	SNHG12	up	RRAD	down
LINC01405	down	CHRNA1	up	SNHG12	up	SERPINE1	down
LINC01405	down	CHRNA1	up	SNHG12	up	SOCS3	down
LINC01405	down	CHRNA1	up	SNHG12	up	SRGN	down
LINC01405	down	COL1A2	up	SNHG12	up	TAP1	down
LINC01405	down	COL1A2	up	SNHG12	up	THBS1	down
LINC01405	down	CTSK	up	SNHG12	up	THBS1	down
LINC01405	down	CTSK	up	SNHG12	up	TNFRSF12A	down

SNHG1	up	TNNI1	down
SNHG1	up	TNNI1	down
SNHG1	up	TNNI1	down
SNHG8	up	TNNI1	down
SNHG8	up	TNNI1	down
SNHG8	up	TNNI1	down
SNHG8	up	TNNI1	down
SNHG8	up	TNNI1	down
SNHG8	up	TNNI1	down
SNHG9	up	TNNI1	down
SNHG9	up	TNNI1	down
SNHG9	up	TNNI1	down
SNHG9	up	TNNI1	down
SNHG9	up	TNNI1	down
SNORA76C	up	TNNI1	down
SNORA76C	up	TNNI1	down
SNORA76C	up	TNNI1	down
SNORA76C	up	TNNI1	down
SNORA76C	up	TNNI1	down
SNORA76C	up	TNNI1	down
**hsa-miR-4459**	**up**	**TNNI1**	**down**
**hsa-miR-7847-3p**	**up**	**TNNI1**	**down**
AC007228.9	up	TPM3	down
AC007228.9	up	TPM3	down
AC011239.1	up	TPM3	down
AC011239.1	up	TPM3	down
AC064871.3	up	TPM3	down
AC064871.3	up	TPM3	down
AC073254.1	up	TPM3	down
AC073254.1	up	TPM3	down
AC073254.1	up	TPM3	down
AC079586.1	up	TPM3	down
AC079586.1	up	TPM3	down
AC123886.2	up	TPM3	down
BCYRN1	up	TPM3	down
BCYRN1	up	TPM3	down
CTC-260E6.6	up	TPM3	down
CTC-297N7.7	up	TPM3	down
CTC-297N7.7	up	TPM3	down
CTC-297N7.7	up	TPM3	down
CTC-297N7.7	up	TPM3	down
CTC-297N7.7	up	TPM3	down
RP11-357D18.1	up	TPM3	down
RP11-386J22.3	up	TPM3	down
RP11-386J22.3	up	TPM3	down
RP11-403P17.4	up	TPM3	down
RP11-403P17.4	up	TPM3	down
RP11-442H21.2	up	TPM3	down
RP11-521L9.1	up	TPM3	down
RP11-521L9.1	up	TPM3	down
RP11-731F5.2	up	TPM3	down

LINC01405	down	DDIT4	up	SNHG12	up	TNFRSF12A	down
LINC01405	down	DNMT3A	up	SNHG12	up	TNNC1	down
LINC01405	down	EEF1A1	up	SNHG12	up	ANKRD1	down
LINC01405	down	EEF1A1	up	SNHG12	up	ANKRD2	down
LINC01405	down	EEF1A1	up	SNHG12	up	ANKRD2	down
LINC01405	down	EEF1A1	up	SNHG12	up	ANKRD2	down
LINC01405	down	EIF4A2	up	SNHG12	up	C10orf10	down
LINC01405	down	EIF4A2	up	SNHG12	up	CCL2	down
LINC01405	down	EIF4A2	up	SNHG12	up	CCL8	down
LINC01405	down	EIF4A2	up	SNHG12	up	CDKN1A	down
LINC01405	down	EIF4A2	up	SNHG12	up	CDKN1A	down
LINC01405	down	EIF4A2	up	SNHG12	up	CDKN1A	down
LINC01405	down	EIF4A2	up	SNHG12	up	CDKN1A	down
LINC01405	down	FAM129A	up	SNHG12	up	CDKN1A	down
LINC01405	down	GREM1	up	SNHG12	up	CKMT2	down
LINC01405	down	GSG2	up	SNHG12	up	CKMT2	down
LINC01405	down	HBA1	up	SNHG12	up	CKMT2	down
LINC01405	down	HBA1	up	SNHG12	up	CKMT2	down
LINC01405	down	HBA2	up	SNHG12	up	CSRP3	down
LINC01405	down	HBA2	up	SNHG12	up	CSRP3	down
LINC01405	down	HBB	up	SNHG12	up	CTSL	down
LINC01405	down	HBD	up	SNHG12	up	CTSL	down
LINC01405	down	HBD	up	SNHG12	up	CTSL	down
LINC01405	down	HIST1H4J	up	SNHG12	up	GADD45G	down
LINC01405	down	HIST1H4K	up	SNHG12	up	HMOX1	down
LINC01405	down	HTRA1	up	SNHG12	up	HMOX1	down
LINC01405	down	IGFBP4	up	SNHG12	up	MYL2	down
LINC01405	down	IGKC	up	SNHG12	up	MYL2	down
LINC01405	down	IGKC	up	SNHG12	up	MYL6B	down
LINC01405	down	IGKC	up	SNHG12	up	MYL6B	down
LINC01405	down	IMPA2	up	SNHG12	up	MYL6B	down
LINC01405	down	IMPA2	up	SNHG12	up	NNMT	down
LINC01405	down	KIAA1328	up	SNHG12	up	PDLIM1	down
LINC01405	down	MKNK2	up	SNHG12	up	RRAD	down
LINC01405	down	MKNK2	up	SNHG12	up	RRAD	down
LINC01405	down	MMP14	up	SNHG12	up	SAT1	down
LINC01405	down	MORC4	up	SNHG12	up	SAT1	down
LINC01405	down	MSS51	up	SNHG12	up	SAT1	down
LINC01405	down	MSS51	up	SNHG12	up	SERPINE1	down
LINC01405	down	MSS51	up	SNHG12	up	SRGN	down
LINC01405	down	MYH1	up	SNHG12	up	TAP1	down
LINC01405	down	MYH4	up	SNHG12	up	THBS1	down
LINC01405	down	MYOC	up	SNHG12	up	THBS1	down
LINC01405	down	MYOC	up	SNHG12	up	TNFRSF12A	down
LINC01405	down	MYOG	up	SNHG12	up	TNFRSF12A	down
LINC01405	down	PRRG3	up	SNHG12	up	TNFRSF12A	down
LINC01405	down	RNASE1	up	SNHG12	up	TNFRSF12A	down
LINC01405	down	RPL27A	up	SNHG12	up	TNNC1	down
LINC01405	down	SPARC	up	SNHG8	up	ACTA1	down
LINC01405	down	SPARC	up	SNHG8	up	C10orf10	down
LINC01405	down	SPARC	up	SNHG8	up	CASQ2	down

RP11-731F5.2	up	TPM3	down
SNHG1	up	TPM3	down
SNHG1	up	TPM3	down
SNHG9	up	TPM3	down
SNHG9	up	TPM3	down
SNORA76C	up	TPM3	down
SNORA76C	up	TPM3	down
**hsa-miR-150-5p**	**up**	**TPM3**	**down**
**hsa-miR-4459**	**up**	**TPM3**	**down**
**hsa-miR-486-3p**	**up**	**TPM3**	**down**
CTD-2033D15.1	down	TPM4	up
CTD-2033D15.1	down	TPM4	up
CTD-2033D15.1	down	TPM4	up
EAF1-AS1	down	TPM4	up
LINC01405	down	TPM4	up
LINC01405	down	TPM4	up
LINC01405	down	TPM4	up
LINC01405	down	TPM4	up
RP11-451G4.2	down	TPM4	up
RP11-451G4.2	down	TPM4	up
RP11-603J24.14	down	TPM4	up
RP11-603J24.14	down	TPM4	up
RP11-705C15.5	down	TPM4	up
RP11-705C15.5	down	TPM4	up
RP11-91P24.7	down	TPM4	up
RP11-91P24.7	down	TPM4	up
**hsa-miR-4516**	**down**	**TPM4**	**up**
CTD-2033D15.1	down	TUBD1	up
CTD-2033D15.1	down	TUBD1	up
CTD-2033D15.1	down	TUBD1	up
CTD-2033D15.1	down	TUBD1	up
CTD-2033D15.1	down	TUBD1	up
LINC01405	down	TUBD1	up
LINC01405	down	TUBD1	up
LINC01405	down	TUBD1	up
LINC01405	down	TUBD1	up
LINC01405	down	TUBD1	up
LINC01405	down	TUBD1	up
LINC01405	down	TUBD1	up
LINC01405	down	TUBD1	up
LINC01405	down	TUBD1	up
LINC01405	down	TUBD1	up
RP11-451G4.2	down	TUBD1	up
RP11-451G4.2	down	TUBD1	up
RP11-451G4.2	down	TUBD1	up
RP11-451G4.2	down	TUBD1	up
RP11-451G4.2	down	TUBD1	up
RP11-480I12.7	down	TUBD1	up
RP11-480I12.7	down	TUBD1	up
RP11-480I12.7	down	TUBD1	up
RP11-480I12.7	down	TUBD1	up

LINC01405	down	SPARC	up	SNHG8	up	CCL8	down
LINC01405	down	SPARC	up	SNHG8	up	CDKN1A	down
LINC01405	down	TBC1D8B	up	SNHG8	up	CDKN1A	down
LINC01405	down	TMEM107	up	SNHG8	up	CDKN1A	down
LINC01405	down	TMSB4X	up	SNHG8	up	CDKN1A	down
LINC01405	down	TMSB4X	up	SNHG8	up	CDKN1A	down
LINC01405	down	TMSB4X	up	SNHG8	up	CKMT2	down
LINC01405	down	TPM4	up	SNHG8	up	CKMT2	down
LINC01405	down	TPM4	up	SNHG8	up	CKMT2	down
LINC01405	down	TRIM22	up	SNHG8	up	CTSL	down
LINC01405	down	TRIM22	up	SNHG8	up	CTSL	down
LINC01405	down	TRIM22	up	SNHG8	up	CTSL	down
LINC01405	down	TRIM22	up	SNHG8	up	GADD45G	down
LINC01405	down	TRIM22	up	SNHG8	up	GADD45G	down
LINC01405	down	TUBD1	up	SNHG8	up	HMOX1	down
LINC01405	down	TUBD1	up	SNHG8	up	HMOX1	down
LINC01405	down	TUBD1	up	SNHG8	up	MYL2	down
LINC01405	down	TUBD1	up	SNHG8	up	MYL2	down
LINC01405	down	TUBD1	up	SNHG8	up	MYL6B	down
LINC01405	down	UCP3	up	SNHG8	up	MYL6B	down
LINC01405	down	ZNF302	up	SNHG8	up	MYL6B	down
LINC01405	down	ZNF302	up	SNHG8	up	MYOM3	down
LINC01405	down	ZNF354B	up	SNHG8	up	NNMT	down
MYHAS	up	ACTA1	down	SNHG8	up	NNMT	down
MYHAS	up	ACTA1	down	SNHG8	up	NNMT	down
MYHAS	up	ANKRD1	down	SNHG8	up	NNMT	down
MYHAS	up	ANKRD2	down	SNHG8	up	NNMT	down
MYHAS	up	ANKRD2	down	SNHG8	up	PDLIM1	down
MYHAS	up	ANKRD2	down	SNHG8	up	SAT1	down
MYHAS	up	ANKRD2	down	SNHG8	up	SAT1	down
MYHAS	up	BHLHE40	down	SNHG8	up	SAT1	down
MYHAS	up	C10orf10	down	SNHG8	up	SERPINE1	down
MYHAS	up	CASQ2	down	SNHG8	up	SOCS3	down
MYHAS	up	CDKN1A	down	SNHG8	up	SRGN	down
MYHAS	up	CDKN1A	down	SNHG8	up	THBS1	down
MYHAS	up	CDKN1A	down	SNHG8	up	THBS1	down
MYHAS	up	CDKN1A	down	SNHG8	up	TNFRSF12A	down
MYHAS	up	CKMT2	down	SNHG8	up	TNFRSF12A	down
MYHAS	up	CKMT2	down	SNHG8	up	TNFRSF12A	down
MYHAS	up	CKMT2	down	SNHG8	up	TNFRSF12A	down
MYHAS	up	CKMT2	down	SNHG8	up	TNFRSF12A	down
MYHAS	up	CRYAB	down	SNHG8	up	TNNC1	down
MYHAS	up	CTSL	down	SNHG8	up	TNNC1	down
MYHAS	up	CTSL	down	SNHG8	up	TNNI1	down
MYHAS	up	CTSL	down	SNHG8	up	TNNI1	down
MYHAS	up	GADD45G	down	SNHG8	up	TNNI1	down
MYHAS	up	GADD45G	down	SNHG8	up	TNNI1	down
MYHAS	up	HMOX1	down	SNHG8	up	TNNI1	down
MYHAS	up	HMOX1	down	SNHG8	up	TNNI1	down
MYHAS	up	MYL2	down	SNHG9	up	ACTA1	down
MYHAS	up	MYL2	down	SNHG9	up	ACTA1	down

RP11-480I12.7	down	TUBD1	up
RP11-603J24.14	down	TUBD1	up
RP11-603J24.14	down	TUBD1	up
RP11-603J24.14	down	TUBD1	up
RP11-603J24.14	down	TUBD1	up
RP11-603J24.14	down	TUBD1	up
RP11-705C15.5	down	TUBD1	up
RP11-705C15.5	down	TUBD1	up
RP11-705C15.5	down	TUBD1	up
RP11-705C15.5	down	TUBD1	up
RP11-705C15.5	down	TUBD1	up
RP11-91P24.7	down	TUBD1	up
RP11-91P24.7	down	TUBD1	up
RP11-91P24.7	down	TUBD1	up
RP11-91P24.7	down	TUBD1	up
RP11-91P24.7	down	TUBD1	up
**hsa-miR-5096**	**down**	**TUBD1**	**up**

MYHAS	up	MYL6B	down	SNHG9	up	ANKRD1	down
MYHAS	up	MYL6B	down	SNHG9	up	ANKRD2	down
MYHAS	up	MYL6B	down	SNHG9	up	ANKRD2	down
MYHAS	up	MYOM3	down	SNHG9	up	ANKRD2	down
MYHAS	up	NNMT	down	SNHG9	up	ANKRD2	down
MYHAS	up	NNMT	down	SNHG9	up	BHLHE40	down
MYHAS	up	NNMT	down	SNHG9	up	C10orf10	down
MYHAS	up	NNMT	down	SNHG9	up	CASQ2	down
MYHAS	up	NNMT	down	SNHG9	up	CCL8	down
MYHAS	up	PDLIM1	down	SNHG9	up	CDKN1A	down
MYHAS	up	RRAD	down	SNHG9	up	CDKN1A	down
MYHAS	up	RRAD	down	SNHG9	up	CDKN1A	down
MYHAS	up	SAT1	down	SNHG9	up	CKMT2	down
MYHAS	up	SAT1	down	SNHG9	up	CKMT2	down
MYHAS	up	SAT1	down	SNHG9	up	CKMT2	down
MYHAS	up	SERPINE1	down	SNHG9	up	CKMT2	down
MYHAS	up	TAP1	down	SNHG9	up	CSRP3	down
MYHAS	up	THBS1	down	SNHG9	up	CSRP3	down
MYHAS	up	THBS1	down	SNHG9	up	CTSL	down
MYHAS	up	TNFRSF12A	down	SNHG9	up	CTSL	down
MYHAS	up	TNFRSF12A	down	SNHG9	up	CTSL	down
MYHAS	up	TNFRSF12A	down	SNHG9	up	GADD45G	down
MYHAS	up	TNFRSF12A	down	SNHG9	up	GADD45G	down
MYHAS	up	TNFRSF12A	down	SNHG9	up	HMOX1	down
MYHAS	up	TNNC1	down	SNHG9	up	HMOX1	down
MYHAS	up	TNNC1	down	SNHG9	up	MYL2	down
MYHAS	up	TNNI1	down	SNHG9	up	MYL2	down
MYHAS	up	TNNI1	down	SNHG9	up	MYL6B	down
MYHAS	up	TNNI1	down	SNHG9	up	MYL6B	down
MYHAS	up	TNNI1	down	SNHG9	up	MYL6B	down
SNORA76C	up	ACTA1	down	SNHG9	up	MYOM3	down
SNORA76C	up	ACTA1	down	SNHG9	up	NNMT	down
SNORA76C	up	ANKRD1	down	SNHG9	up	NNMT	down
SNORA76C	up	ANKRD2	down	SNHG9	up	NNMT	down
SNORA76C	up	ANKRD2	down	SNHG9	up	NNMT	down
SNORA76C	up	ANKRD2	down	SNHG9	up	NNMT	down
SNORA76C	up	ANKRD2	down	SNHG9	up	PDLIM1	down
SNORA76C	up	BHLHE40	down	SNHG9	up	SAT1	down
SNORA76C	up	C10orf10	down	SNHG9	up	SAT1	down
SNORA76C	up	CASQ2	down	SNHG9	up	SAT1	down
SNORA76C	up	CDKN1A	down	SNHG9	up	SERPINE1	down
SNORA76C	up	CDKN1A	down	SNHG9	up	SOCS3	down
SNORA76C	up	CDKN1A	down	SNHG9	up	SRGN	down
SNORA76C	up	CDKN1A	down	SNHG9	up	TAP1	down
SNORA76C	up	CDKN1A	down	SNHG9	up	THBS1	down
SNORA76C	up	CKMT2	down	SNHG9	up	TNNC1	down
SNORA76C	up	CKMT2	down	SNHG9	up	TNNC1	down
SNORA76C	up	CKMT2	down	SNHG9	up	TNNI1	down
SNORA76C	up	CKMT2	down	SNHG9	up	TNNI1	down
SNORA76C	up	CTSL	down	SNHG9	up	TNNI1	down
SNORA76C	up	CTSL	down	SNHG9	up	TNNI1	down

SNORA76C	up	CTSL	down	SNHG9	up	TNNI1	down
SNORA76C	up	GADD45G	down	SNHG9	up	TPM3	down
SNORA76C	up	GADD45G	down	SNHG9	up	TPM3	down
SNORA76C	up	HMOX1	down				
SNORA76C	up	HMOX1	down				
SNORA76C	up	MYL2	down				
SNORA76C	up	MYL2	down				
SNORA76C	up	MYL6B	down				
SNORA76C	up	MYOM3	down				
SNORA76C	up	NNMT	down				
SNORA76C	up	NNMT	down				
SNORA76C	up	NNMT	down				
SNORA76C	up	NNMT	down				
SNORA76C	up	NNMT	down				
SNORA76C	up	PDLIM1	down				
SNORA76C	up	RRAD	down				
SNORA76C	up	RRAD	down				
SNORA76C	up	SRGN	down				
SNORA76C	up	THBS1	down				
SNORA76C	up	THBS1	down				
SNORA76C	up	TNFRSF12A	down				
SNORA76C	up	TNFRSF12A	down				
SNORA76C	up	TNFRSF12A	down				
SNORA76C	up	TNFRSF12A	down				
SNORA76C	up	TNFRSF12A	down				
SNORA76C	up	TNNC1	down				
SNORA76C	up	TNNI1	down				
SNORA76C	up	TNNI1	down				
SNORA76C	up	TNNI1	down				
SNORA76C	up	TNNI1	down				
SNORA76C	up	TNNI1	down				
SNORA76C	up	TNNI1	down				
SNORA76C	up	TPM3	down				
SNORA76C	up	TPM3	down				

Supplementary Table S5A.

GO analyses of the dysregulated mRNAs, miRNA targets and lncRNA targets

GO analyses of the dysregulated mRNAs

GO_ID	GO_Term	S.gene. number	TS.gene. number	B.gene. number	TB.gene. number	pvalue
**GO:0005615**	extracellular space	43	172	661	11484	2.22E-16
**GO:0031012**	extracellular matrix	16	172	122	11484	3.31E-11
**GO:0005576**	extracellular region	42	172	935	11484	5.77E-11
**GO:0030049**	muscle filament sliding	8	172	19	11484	1.41E-10
**GO:0006936**	muscle contraction	11	172	52	11484	2.19E-10
**GO:0031093**	platelet alpha granule lumen	8	172	21	11484	3.71E-10
**GO:0070062**	extracellular vesicular exosome	52	172	1439	11484	5.26E-10
**GO:0002576**	platelet degranulation	9	172	40	11484	5.64E-09
**GO:0030017**	sarcomere	7	172	23	11484	3.00E-08
**GO:0072562**	blood microparticle	11	172	88	11484	7.29E-08
**GO:0071276**	cellular response to cadmium ion	5	172	9	11484	8.54E-08
**GO:0071294**	cellular response to zinc ion	5	172	11	11484	3.05E-07
**GO:0030168**	platelet activation	11	172	111	11484	8.00E-07
**GO:0045926**	negative regulation of growth	5	172	13	11484	8.30E-07
**GO:0030198**	extracellular matrix organization	13	172	172	11484	1.79E-06
**GO:0008285**	negative regulation of cell proliferation	15	172	238	11484	2.78E-06
**GO:0071682**	endocytic vesicle lumen	4	172	8	11484	3.25E-06
**GO:0015671**	oxygen transport	4	172	9	11484	5.77E-06
**GO:0008016**	regulation of heart contraction	5	172	20	11484	9.19E-06
**GO:0022617**	extracellular matrix disassembly	8	172	72	11484	1.14E-05
**GO:0008009**	chemokine activity	6	172	38	11484	1.92E-05
**GO:0030016**	myofibril	5	172	23	11484	1.92E-05
**GO:0005829**	cytosol	43	172	1536	11484	2.67E-05
**GO:0005578**	proteinaceous extracellular matrix	10	172	136	11484	3.66E-05
**GO:0055010**	ventricular cardiac muscle tissue morphogenesis	4	172	14	11484	4.33E-05
**GO:0005604**	basement membrane	6	172	44	11484	4.56E-05
**GO:0015629**	actin cytoskeleton	9	172	115	11484	5.55E-05
**GO:0001725**	stress fiber	5	172	29	11484	6.31E-05
**GO:0008201**	heparin binding	7	172	74	11484	0.000116
**GO:0030308**	negative regulation of cell growth	7	172	75	11484	0.000126
**GO:0007050**	cell cycle arrest	7	172	77	11484	0.000149
**GO:0005344**	oxygen transporter activity	3	172	8	11484	0.000175
**GO:0031672**	A band	3	172	8	11484	0.000175
**GO:0045653**	negative regulation of megakaryocyte differentiation	3	172	8	11484	0.000175

**GO:0010951**	negative regulation of endopeptidase activity	7	172	79	11484	0.000175
**GO:0004867**	serine-type endopeptidase inhibitor activity	6	172	57	11484	0.000199
**GO:0031720**	haptoglobin binding	2	172	2	11484	0.000223
**GO:0031838**	haptoglobin-hemoglobin complex	2	172	2	11484	0.000223
**GO:0036018**	cellular response to erythropoietin	2	172	2	11484	0.000223
**GO:0045651**	positive regulation of macrophage differentiation	2	172	2	11484	0.000223
**GO:2000098**	negative regulation of smooth muscle cell-matrix adhesion	2	172	2	11484	0.000223
**GO:0007596**	blood coagulation	13	172	273	11484	0.000233
**GO:0005212**	structural constituent of eye lens	3	172	9	11484	0.00026
**GO:0007512**	adult heart development	3	172	9	11484	0.00026
**GO:0005737**	cytoplasm	62	172	2791	11484	0.000346
**GO:0034605**	cellular response to heat	4	172	24	11484	0.000409
**GO:0050679**	positive regulation of epithelial cell proliferation	5	172	43	11484	0.000432
**GO:0001618**	virus receptor activity	4	172	25	11484	0.000481
**GO:0042542**	response to hydrogen peroxide	4	172	25	11484	0.000481
**GO:0003779**	actin binding	10	172	186	11484	0.000484
**GO:0048471**	perinuclear region of cytoplasm	14	172	342	11484	0.000625
**GO:0010744**	positive regulation of macrophage derived foam cell differentiation	3	172	12	11484	0.000658
**GO:0031674**	I band	3	172	12	11484	0.000658
**GO:0045214**	sarcomere organization	3	172	12	11484	0.000658
**GO:0046688**	response to copper ion	3	172	12	11484	0.000658
**GO:0000791**	euchromatin	2	172	3	11484	0.000663
**GO:0005862**	muscle thin filament tropomyosin	2	172	3	11484	0.000663
**GO:0007568**	aging	6	172	72	11484	0.000714
**GO:0009615**	response to virus	7	172	100	11484	0.000744
**GO:2001240**	negative regulation of extrinsic apoptotic signaling pathway in absence of ligand	4	172	29	11484	0.000861
**GO:0045429**	positive regulation of nitric oxide biosynthetic process	4	172	31	11484	0.001115
**GO:0007267**	cell-cell signaling	8	172	140	11484	0.001202
**GO:0006942**	regulation of striated muscle contraction	2	172	4	11484	0.001312
**GO:0030195**	negative regulation of blood coagulation	2	172	4	11484	0.001312

**GO:1900745**	positive regulation of p38MAPK cascade	2	172	4	11484	0.001312
**GO:0009612**	response to mechanical stimulus	4	172	33	11484	0.001417
**GO:0019825**	oxygen binding	3	172	16	11484	0.001602
**GO:2000379**	positive regulation of reactive oxygen species metabolic process	3	172	16	11484	0.001602
**GO:0005509**	calcium ion binding	15	172	423	11484	0.00172
**GO:0030155**	regulation of cell adhesion	3	172	17	11484	0.001924
**GO:0008217**	regulation of blood pressure	4	172	36	11484	0.001969
**GO:0071260**	cellular response to mechanical stimulus	4	172	36	11484	0.001969
**GO:0009629**	response to gravity	2	172	5	11484	0.002165
**GO:0042117**	monocyte activation	2	172	5	11484	0.002165
**GO:0045820**	negative regulation of glycolytic process	2	172	5	11484	0.002165
**GO:0048711**	positive regulation of astrocyte differentiation	2	172	5	11484	0.002165
**GO:0051918**	negative regulation of fibrinolysis	2	172	5	11484	0.002165
**GO:1900027**	regulation of ruffle assembly	2	172	5	11484	0.002165
**GO:0042026**	protein refolding	3	172	18	11484	0.002283
**GO:0005518**	collagen binding	4	172	38	11484	0.002411
**GO:0043010**	camera-type eye development	4	172	38	11484	0.002411
**GO:0006986**	response to unfolded protein	4	172	39	11484	0.002656
**GO:0002027**	regulation of heart rate	3	172	19	11484	0.002682
**GO:0007566**	embryo implantation	3	172	20	11484	0.00312
**GO:0009409**	response to cold	3	172	20	11484	0.00312
**GO:0010243**	response to organonitrogen compound	3	172	20	11484	0.00312
**GO:0042733**	embryonic digit morphogenesis	4	172	41	11484	0.003195
**GO:0019800**	peptide cross-linking via chondroitin 4-sulfate glycosaminoglycan	2	172	6	11484	0.003216
**GO:0050786**	RAGE receptor binding	2	172	6	11484	0.003216
**GO:0061045**	negative regulation of wound healing	2	172	6	11484	0.003216
**GO:0008307**	structural constituent of muscle	3	172	22	11484	0.004124
**GO:0030593**	neutrophil chemotaxis	3	172	22	11484	0.004124
**GO:0006954**	inflammatory response	9	172	210	11484	0.004343
**GO:0005833**	hemoglobin complex	2	172	7	11484	0.004458
**GO:0005859**	muscle myosin complex	2	172	7	11484	0.004458
**GO:0010165**	response to X-ray	2	172	7	11484	0.004458
**GO:0030898**	actin-dependent ATPase activity	2	172	7	11484	0.004458
**GO:0050663**	cytokine secretion	2	172	7	11484	0.004458

**GO:0055003**	cardiac myofibril assembly	2	172	7	11484	0.004458
**GO:0097067**	cellular response to thyroid hormone stimulus	2	172	7	11484	0.004458
**GO:0032496**	response to lipopolysaccharide	5	172	73	11484	0.004701
**GO:0005856**	cytoskeleton	9	172	214	11484	0.004909
**GO:0014070**	response to organic cyclic compound	5	172	75	11484	0.005274
**GO:0048015**	phosphatidylinositol-mediated signaling	5	172	75	11484	0.005274
**GO:0035914**	skeletal muscle cell differentiation	3	172	24	11484	0.005302
**GO:0005634**	nucleus	66	172	3346	11484	0.00544
**GO:0006950**	response to stress	4	172	48	11484	0.005661
**GO:0007565**	female pregnancy	4	172	48	11484	0.005661
**GO:0000185**	activation of MAPKKK activity	2	172	8	11484	0.005885
**GO:0006882**	cellular zinc ion homeostasis	2	172	8	11484	0.005885
**GO:0044548**	S100 protein binding	2	172	8	11484	0.005885
**GO:0005791**	rough endoplasmic reticulum	3	172	25	11484	0.00596
**GO:0048146**	positive regulation of fibroblast proliferation	3	172	25	11484	0.00596
**GO:0016887**	ATPase activity	6	172	110	11484	0.006144
**GO:0008083**	growth factor activity	5	172	78	11484	0.006224
**GO:0048306**	calcium-dependent protein binding	3	172	26	11484	0.006664
**GO:0071347**	cellular response to interleukin-1	3	172	26	11484	0.006664
**GO:0042493**	response to drug	8	172	186	11484	0.006892
**GO:0007603**	phototransduction, visible light	4	172	51	11484	0.007024
**GO:0060021**	palate development	4	172	51	11484	0.007024
**GO:0030162**	regulation of proteolysis	3	172	27	11484	0.007416
**GO:0005504**	fatty acid binding	2	172	9	11484	0.007493
**GO:0005916**	fascia adherens	2	172	9	11484	0.007493
**GO:0006941**	striated muscle contraction	2	172	9	11484	0.007493
**GO:0019433**	triglyceride catabolic process	2	172	9	11484	0.007493
**GO:0031432**	titin binding	2	172	9	11484	0.007493
**GO:0035574**	histone H4-K20 demethylation	2	172	9	11484	0.007493
**GO:0035575**	histone demethylase activity (H4-K20 specific)	2	172	9	11484	0.007493
**GO:0043292**	contractile fiber	2	172	9	11484	0.007493
**GO:0051412**	response to corticosterone	2	172	9	11484	0.007493
**GO:0060213**	positive regulation of nuclear-transcribed mRNA poly(A) tail shortening	2	172	9	11484	0.007493
**GO:0070370**	cellular heat acclimation	2	172	9	11484	0.007493
**GO:0043234**	protein complex	9	172	232	11484	0.008201
**GO:0008180**	COP9 signalosome	3	172	28	11484	0.008215

**GO:0050729**	positive regulation of inflammatory response	3	172	28	11484	0.008215
**GO:0051591**	response to cAMP	3	172	28	11484	0.008215
**GO:0001525**	angiogenesis	6	172	117	11484	0.008239
**GO:0002026**	regulation of the force of heart contraction	2	172	10	11484	0.009274
**GO:0032982**	myosin filament	2	172	10	11484	0.009274
**GO:0035924**	cellular response to vascular endothelial growth factor stimulus	2	172	10	11484	0.009274
**GO:0042246**	tissue regeneration	2	172	10	11484	0.009274
**GO:0042629**	mast cell granule	2	172	10	11484	0.009274
**GO:0030036**	actin cytoskeleton organization	5	172	86	11484	0.009336
**GO:0030018**	Z disc	4	172	57	11484	0.010361
**GO:0001666**	response to hypoxia	5	172	89	11484	0.010737
**GO:0002102**	podosome	2	172	11	11484	0.011224
**GO:0044183**	protein binding involved in protein folding	2	172	11	11484	0.011224
**GO:0090084**	negative regulation of inclusion body assembly	2	172	11	11484	0.011224
**GO:0016459**	myosin complex	3	172	32	11484	0.011908
**GO:0051403**	stress-activated MAPK cascade	3	172	32	11484	0.011908
**GO:0001523**	retinoid metabolic process	3	172	33	11484	0.012957
**GO:0005201**	extracellular matrix structural constituent	3	172	33	11484	0.012957
**GO:0006928**	cellular component movement	4	172	61	11484	0.013074
**GO:0005539**	glycosaminoglycan binding	2	172	12	11484	0.013337
**GO:0005605**	basal lamina	2	172	12	11484	0.013337
**GO:0031668**	cellular response to extracellular stimulus	2	172	12	11484	0.013337
**GO:0042623**	ATPase activity, coupled	2	172	12	11484	0.013337
**GO:0051602**	response to electrical stimulus	2	172	12	11484	0.013337
**GO:2000352**	negative regulation of endothelial cell apoptotic process	2	172	12	11484	0.013337
**GO:0001649**	osteoblast differentiation	4	172	62	11484	0.013817
**GO:0044281**	small molecule metabolic process	21	172	839	11484	0.013877
**GO:0003725**	double-stranded RNA binding	3	172	34	11484	0.014057
**GO:0043434**	response to peptide hormone	3	172	34	11484	0.014057
**GO:0001135**	RNA polymerase II transcription factor recruiting transcription factor activity	1	172	1	11484	0.014977

**GO:0001226**	RNA polymerase II transcription corepressor binding	1	172	1	11484	0.014977
**GO:0001680**	tRNA 3'-terminal CCA addition	1	172	1	11484	0.014977
**GO:0001869**	negative regulation of complement activation, lectin pathway	1	172	1	11484	0.014977
**GO:0001872**	(1->3)-beta-D-glucan binding	1	172	1	11484	0.014977
**GO:0001910**	regulation of leukocyte mediated cytotoxicity	1	172	1	11484	0.014977
**GO:0001998**	angiotensin mediated vasoconstriction involved in regulation of systemic arterial blood pressure	1	172	1	11484	0.014977
**GO:0001999**	renal response to blood flow involved in circulatory renin-angiotensin regulation of systemic arterial blood pressure	1	172	1	11484	0.014977
**GO:0002019**	regulation of renal output by angiotensin	1	172	1	11484	0.014977
**GO:0002060**	purine nucleobase binding	1	172	1	11484	0.014977
**GO:0002291**	T cell activation via T cell receptor contact with antigen bound to MHC molecule on antigen presenting cell	1	172	1	11484	0.014977
**GO:0002457**	T cell antigen processing and presentation	1	172	1	11484	0.014977
**GO:0002528**	regulation of vascular permeability involved in acute inflammatory response	1	172	1	11484	0.014977
**GO:0003294**	atrial ventricular junction remodeling	1	172	1	11484	0.014977
**GO:0004465**	lipoprotein lipase activity	1	172	1	11484	0.014977
**GO:0005602**	complement component C1 complex	1	172	1	11484	0.014977
**GO:0006148**	inosine catabolic process	1	172	1	11484	0.014977
**GO:0006738**	nicotinamide riboside catabolic process	1	172	1	11484	0.014977
**GO:0007522**	visceral muscle development	1	172	1	11484	0.014977
**GO:0008112**	nicotinamide N-methyltransferase activity	1	172	1	11484	0.014977
**GO:0008934**	inositol monophosphate 1-phosphatase activity	1	172	1	11484	0.014977
**GO:0009991**	response to extracellular stimulus	1	172	1	11484	0.014977
**GO:0010232**	vascular transport	1	172	1	11484	0.014977

**GO:0010273**	detoxification of copper ion	1	172	1	11484	0.014977
**GO:0010477**	response to sulfur dioxide	1	172	1	11484	0.014977
**GO:0010643**	cell communication by chemical coupling	1	172	1	11484	0.014977
**GO:0010644**	cell communication by electrical coupling	1	172	1	11484	0.014977
**GO:0010652**	positive regulation of cell communication by chemical coupling	1	172	1	11484	0.014977
**GO:0014734**	skeletal muscle hypertrophy	1	172	1	11484	0.014977
**GO:0014829**	vascular smooth muscle contraction	1	172	1	11484	0.014977
**GO:0014873**	response to muscle activity involved in regulation of muscle adaptation	1	172	1	11484	0.014977
**GO:0017129**	triglyceride binding	1	172	1	11484	0.014977
**GO:0019912**	cyclin-dependent protein kinase activating kinase activity	1	172	1	11484	0.014977
**GO:0021555**	midbrain-hindbrain boundary morphogenesis	1	172	1	11484	0.014977
**GO:0021557**	oculomotor nerve development	1	172	1	11484	0.014977
**GO:0021558**	trochlear nerve development	1	172	1	11484	0.014977
**GO:0030682**	evasion or tolerance of host defense response	1	172	1	11484	0.014977
**GO:0031109**	microtubule polymerization or depolymerization	1	172	1	11484	0.014977
**GO:0031444**	slow-twitch skeletal muscle fiber contraction	1	172	1	11484	0.014977
**GO:0031703**	type 2 angiotensin receptor binding	1	172	1	11484	0.014977
**GO:0032185**	septin cytoskeleton organization	1	172	1	11484	0.014977
**GO:0032217**	riboflavin transporter activity	1	172	1	11484	0.014977
**GO:0032218**	riboflavin transport	1	172	1	11484	0.014977
**GO:0033371**	T cell secretory granule organization	1	172	1	11484	0.014977
**GO:0033373**	maintenance of protease location in mast cell secretory granule	1	172	1	11484	0.014977
**GO:0033382**	maintenance of granzyme B location in T cell secretory granule	1	172	1	11484	0.014977
**GO:0034104**	negative regulation of tissue remodeling	1	172	1	11484	0.014977
**GO:0034356**	NAD biosynthesis via nicotinamide riboside salvage pathway	1	172	1	11484	0.014977

**GO:0035491**	positive regulation of leukotriene production involved in inflammatory response	1	172	1	11484	0.014977
**GO:0035583**	sequestering of TGFbeta in extracellular matrix	1	172	1	11484	0.014977
**GO:0038133**	ERBB2-ERBB3 signaling pathway	1	172	1	11484	0.014977
**GO:0042694**	muscle cell fate specification	1	172	1	11484	0.014977
**GO:0043120**	tumor necrosis factor binding	1	172	1	11484	0.014977
**GO:0043241**	protein complex disassembly	1	172	1	11484	0.014977
**GO:0044027**	hypermethylation of CpG island	1	172	1	11484	0.014977
**GO:0044346**	fibroblast apoptotic process	1	172	1	11484	0.014977
**GO:0044406**	adhesion of symbiont to host	1	172	1	11484	0.014977
**GO:0045091**	regulation of single stranded viral RNA replication via double stranded DNA intermediate	1	172	1	11484	0.014977
**GO:0045203**	integral component of cell outer membrane	1	172	1	11484	0.014977
**GO:0045503**	dynein light chain binding	1	172	1	11484	0.014977
**GO:0045608**	negative regulation of auditory receptor cell differentiation	1	172	1	11484	0.014977
**GO:0045977**	positive regulation of mitotic cell cycle, embryonic	1	172	1	11484	0.014977
**GO:0046331**	lateral inhibition	1	172	1	11484	0.014977
**GO:0046813**	receptor-mediated virion attachment to host cell	1	172	1	11484	0.014977
**GO:0047127**	thiomorpholine-carboxylate dehydrogenase activity	1	172	1	11484	0.014977
**GO:0047280**	nicotinamide phosphoribosyltransferase activity	1	172	1	11484	0.014977
**GO:0047992**	hydroxylysine kinase activity	1	172	1	11484	0.014977
**GO:0048248**	CXCR3 chemokine receptor binding	1	172	1	11484	0.014977
**GO:0048269**	methionine adenosyltransferase complex	1	172	1	11484	0.014977
**GO:0050614**	delta24-sterol reductase activity	1	172	1	11484	0.014977
**GO:0050703**	interleukin-1 alpha secretion	1	172	1	11484	0.014977
**GO:0050716**	positive regulation of interleukin-1 secretion	1	172	1	11484	0.014977
**GO:0051014**	actin filament severing	1	172	1	11484	0.014977
**GO:0051089**	constitutive protein ectodomain proteolysis	1	172	1	11484	0.014977
**GO:0051545**	negative regulation of elastin biosynthetic process	1	172	1	11484	0.014977

**GO:0051969**	regulation of transmission of nerve impulse	1	172	1	11484	0.014977
**GO:0052591**	sn-glycerol-3-phosphate:ubiquinone-8 oxidoreductase activity	1	172	1	11484	0.014977
**GO:0052832**	inositol monophosphate 3-phosphatase activity	1	172	1	11484	0.014977
**GO:0052833**	inositol monophosphate 4-phosphatase activity	1	172	1	11484	0.014977
**GO:0052927**	CTP:tRNA cytidylyltransferase activity	1	172	1	11484	0.014977
**GO:0052928**	CTP:3'-cytidine-tRNA cytidylyltransferase activity	1	172	1	11484	0.014977
**GO:0052929**	ATP:3'-cytidine-cytidine-tRNA adenylyltransferase activity	1	172	1	11484	0.014977
**GO:0060156**	milk ejection	1	172	1	11484	0.014977
**GO:0060212**	negative regulation of nuclear-transcribed mRNA poly(A) tail shortening	1	172	1	11484	0.014977
**GO:0060420**	regulation of heart growth	1	172	1	11484	0.014977
**GO:0060588**	negative regulation of lipoprotein lipid oxidation	1	172	1	11484	0.014977
**GO:0060675**	ureteric bud morphogenesis	1	172	1	11484	0.014977
**GO:0061009**	common bile duct development	1	172	1	11484	0.014977
**GO:0061041**	regulation of wound healing	1	172	1	11484	0.014977
**GO:0061044**	negative regulation of vascular wound healing	1	172	1	11484	0.014977
**GO:0061106**	negative regulation of stomach neuroendocrine cell differentiation	1	172	1	11484	0.014977
**GO:0061626**	pharyngeal arch artery morphogenesis	1	172	1	11484	0.014977
**GO:0070236**	negative regulation of activation-induced cell death of T cells	1	172	1	11484	0.014977
**GO:0070287**	ferritin receptor activity	1	172	1	11484	0.014977
**GO:0070471**	uterine smooth muscle contraction	1	172	1	11484	0.014977
**GO:0070557**	PCNA-p21 complex	1	172	1	11484	0.014977
**GO:0070970**	interleukin-2 secretion	1	172	1	11484	0.014977
**GO:0071820**	N-box binding	1	172	1	11484	0.014977
**GO:0071888**	macrophage apoptotic process	1	172	1	11484	0.014977
**GO:0072141**	renal interstitial cell development	1	172	1	11484	0.014977
**GO:0072600**	establishment of protein localization to Golgi	1	172	1	11484	0.014977

**GO:0090096**	positive regulation of metanephric cap mesenchymal cell proliferation	1	172	1	11484	0.014977
**GO:0090521**	glomerular visceral epithelial cell migration	1	172	1	11484	0.014977
**GO:0097368**	establishment of Sertoli cell barrier	1	172	1	11484	0.014977
**GO:0097493**	structural molecule activity conferring elasticity	1	172	1	11484	0.014977
**GO:0097541**	axonemal basal plate	1	172	1	11484	0.014977
**GO:1901003**	negative regulation of fermentation	1	172	1	11484	0.014977
**GO:1901387**	positive regulation of voltage-gated calcium channel activity	1	172	1	11484	0.014977
**GO:1901676**	positive regulation of histone H3-K27 acetylation	1	172	1	11484	0.014977
**GO:2000227**	negative regulation of pancreatic A cell differentiation	1	172	1	11484	0.014977
**GO:2000405**	negative regulation of T cell migration	1	172	1	11484	0.014977
**GO:2000564**	regulation of CD8-positive, alpha-beta T cell proliferation	1	172	1	11484	0.014977
**GO:2000751**	histone H3-T3 phosphorylation involved in chromosome passenger complex localization to kinetochore	1	172	1	11484	0.014977
**GO:0000146**	microfilament motor activity	2	172	13	11484	0.015609
**GO:0001968**	fibronectin binding	2	172	13	11484	0.015609
**GO:0042744**	hydrogen peroxide catabolic process	2	172	13	11484	0.015609
**GO:0045909**	positive regulation of vasodilation	2	172	13	11484	0.015609
**GO:0007173**	epidermal growth factor receptor signaling pathway	5	172	98	11484	0.01579
**GO:0000079**	regulation of cyclin-dependent protein serine/threonine kinase activity	3	172	36	11484	0.016413
**GO:0050731**	positive regulation of peptidyl-tyrosine phosphorylation	3	172	37	11484	0.017669
**GO:0005524**	ATP binding	22	172	913	11484	0.017744
**GO:0006336**	DNA replication-independent nucleosome assembly	2	172	14	11484	0.018033
**GO:0015701**	bicarbonate transport	2	172	14	11484	0.018033
**GO:0050840**	extracellular matrix binding	2	172	14	11484	0.018033
**GO:0071353**	cellular response to interleukin-4	2	172	14	11484	0.018033

**GO:1902042**	negative regulation of extrinsic apoptotic signaling pathway via death domain receptors	2	172	14	11484	0.018033
**GO:0046872**	metal ion binding	28	172	1254	11484	0.019896
**GO:0001974**	blood vessel remodeling	2	172	15	11484	0.020605
**GO:0004601**	peroxidase activity	2	172	15	11484	0.020605
**GO:0045648**	positive regulation of erythrocyte differentiation	2	172	15	11484	0.020605
**GO:0045672**	positive regulation of osteoclast differentiation	2	172	15	11484	0.020605
**GO:0071310**	cellular response to organic substance	2	172	15	11484	0.020605
**GO:0010628**	positive regulation of gene expression	5	172	105	11484	0.020669
**GO:0051082**	unfolded protein binding	4	172	70	11484	0.020724
**GO:0001822**	kidney development	4	172	71	11484	0.021712
**GO:0002020**	protease binding	3	172	41	11484	0.023214
**GO:0043202**	lysosomal lumen	3	172	41	11484	0.023214
**GO:0045944**	positive regulation of transcription from RNA polymerase II promoter	13	172	467	11484	0.023265
**GO:0010942**	positive regulation of cell death	2	172	16	11484	0.023319
**GO:0045907**	positive regulation of vasoconstriction	2	172	16	11484	0.023319
**GO:0006006**	glucose metabolic process	4	172	73	11484	0.023774
**GO:0007179**	transforming growth factor beta receptor signaling pathway	4	172	73	11484	0.023774
**GO:0043085**	positive regulation of catalytic activity	3	172	42	11484	0.024731
**GO:0051384**	response to glucocorticoid	3	172	42	11484	0.024731
**GO:0005102**	receptor binding	7	172	191	11484	0.024828
**GO:0051726**	regulation of cell cycle	4	172	74	11484	0.024848
**GO:0030335**	positive regulation of cell migration	4	172	75	11484	0.02595
**GO:0004866**	endopeptidase inhibitor activity	2	172	17	11484	0.026172
**GO:0006335**	DNA replication-dependent nucleosome assembly	2	172	17	11484	0.026172
**GO:0007346**	regulation of mitotic cell cycle	2	172	17	11484	0.026172
**GO:0030282**	bone mineralization	2	172	17	11484	0.026172
**GO:0043531**	ADP binding	2	172	17	11484	0.026172
**GO:0045765**	regulation of angiogenesis	2	172	17	11484	0.026172
**GO:0051924**	regulation of calcium ion transport	2	172	17	11484	0.026172
**GO:0071479**	cellular response to ionizing radiation	2	172	17	11484	0.026172
**GO:0097110**	scaffold protein binding	2	172	17	11484	0.026172

**GO:0006955**	immune response	10	172	329	11484	0.026278
**GO:0006469**	negative regulation of protein kinase activity	3	172	43	11484	0.0263
**GO:0008144**	drug binding	3	172	43	11484	0.0263
**GO:0060326**	cell chemotaxis	3	172	43	11484	0.0263
**GO:0042995**	cell projection	3	172	44	11484	0.027922
**GO:0002088**	lens development in camera-type eye	2	172	18	11484	0.029158
**GO:0006352**	DNA-templated transcription, initiation	2	172	18	11484	0.029158
**GO:0006953**	acute-phase response	2	172	18	11484	0.029158
**GO:0007263**	nitric oxide mediated signal transduction	2	172	18	11484	0.029158
**GO:0016234**	inclusion body	2	172	18	11484	0.029158
**GO:0042517**	positive regulation of tyrosine phosphorylation of Stat3 protein	2	172	18	11484	0.029158
**GO:2001238**	positive regulation of extrinsic apoptotic signaling pathway	2	172	18	11484	0.029158
**GO:0007507**	heart development	5	172	115	11484	0.029204
**GO:0045471**	response to ethanol	3	172	45	11484	0.029596
**GO:0001300**	chronological cell aging	1	172	2	11484	0.029732
**GO:0001543**	ovarian follicle rupture	1	172	2	11484	0.029732
**GO:0001562**	response to protozoan	1	172	2	11484	0.029732
**GO:0001878**	response to yeast	1	172	2	11484	0.029732
**GO:0001991**	regulation of systemic arterial blood pressure by circulatory renin-angiotensin	1	172	2	11484	0.029732
**GO:0002016**	regulation of blood volume by renin-angiotensin	1	172	2	11484	0.029732
**GO:0002034**	regulation of blood vessel size by renin-angiotensin	1	172	2	11484	0.029732
**GO:0002035**	brain renin-angiotensin system	1	172	2	11484	0.029732
**GO:0002438**	acute inflammatory response to antigenic stimulus	1	172	2	11484	0.029732
**GO:0002693**	positive regulation of cellular extravasation	1	172	2	11484	0.029732
**GO:0003051**	angiotensin-mediated drinking behavior	1	172	2	11484	0.029732
**GO:0003104**	positive regulation of glomerular filtration	1	172	2	11484	0.029732
**GO:0003158**	endothelium development	1	172	2	11484	0.029732
**GO:0004356**	glutamate-ammonia ligase activity	1	172	2	11484	0.029732
**GO:0004368**	glycerol-3-phosphate dehydrogenase activity	1	172	2	11484	0.029732

**GO:0004478**	methionine adenosyltransferase activity	1	172	2	11484	0.029732
**GO:0004667**	prostaglandin-D synthase activity	1	172	2	11484	0.029732
**GO:0004687**	myosin light chain kinase activity	1	172	2	11484	0.029732
**GO:0004731**	purine-nucleoside phosphorylase activity	1	172	2	11484	0.029732
**GO:0004810**	tRNA adenylyltransferase activity	1	172	2	11484	0.029732
**GO:0005146**	leukemia inhibitory factor receptor binding	1	172	2	11484	0.029732
**GO:0005153**	interleukin-8 receptor binding	1	172	2	11484	0.029732
**GO:0005589**	collagen type VI trimer	1	172	2	11484	0.029732
**GO:0005853**	eukaryotic translation elongation factor 1 complex	1	172	2	11484	0.029732
**GO:0006021**	inositol biosynthetic process	1	172	2	11484	0.029732
**GO:0006538**	glutamate catabolic process	1	172	2	11484	0.029732
**GO:0006542**	glutamine biosynthetic process	1	172	2	11484	0.029732
**GO:0006556**	S-adenosylmethionine biosynthetic process	1	172	2	11484	0.029732
**GO:0006769**	nicotinamide metabolic process	1	172	2	11484	0.029732
**GO:0006931**	substrate-dependent cell migration, cell attachment to substrate	1	172	2	11484	0.029732
**GO:0007098**	centrosome cycle	1	172	2	11484	0.029732
**GO:0008626**	granzyme-mediated apoptotic signaling pathway	1	172	2	11484	0.029732
**GO:0010565**	regulation of cellular ketone metabolic process	1	172	2	11484	0.029732
**GO:0010757**	negative regulation of plasminogen activation	1	172	2	11484	0.029732
**GO:0016476**	regulation of embryonic cell shape	1	172	2	11484	0.029732
**GO:0019966**	interleukin-1 binding	1	172	2	11484	0.029732
**GO:0021575**	hindbrain morphogenesis	1	172	2	11484	0.029732
**GO:0022614**	membrane to membrane docking	1	172	2	11484	0.029732
**GO:0030070**	insulin processing	1	172	2	11484	0.029732
**GO:0030185**	nitric oxide transport	1	172	2	11484	0.029732
**GO:0030492**	hemoglobin binding	1	172	2	11484	0.029732
**GO:0030801**	positive regulation of cyclic nucleotide metabolic process	1	172	2	11484	0.029732
**GO:0031701**	angiotensin receptor binding	1	172	2	11484	0.029732
**GO:0031997**	N-terminal myristoylation domain binding	1	172	2	11484	0.029732

**GO:0032027**	myosin light chain binding	1	172	2	11484	0.029732
**GO:0032204**	regulation of telomere maintenance	1	172	2	11484	0.029732
**GO:0032432**	actin filament bundle	1	172	2	11484	0.029732
**GO:0033188**	sphingomyelin synthase activity	1	172	2	11484	0.029732
**GO:0033364**	mast cell secretory granule organization	1	172	2	11484	0.029732
**GO:0033864**	positive regulation of NAD(P)H oxidase activity	1	172	2	11484	0.029732
**GO:0034124**	regulation of MyD88-dependent toll-like receptor signaling pathway	1	172	2	11484	0.029732
**GO:0034371**	chylomicron remodeling	1	172	2	11484	0.029732
**GO:0034372**	very-low-density lipoprotein particle remodeling	1	172	2	11484	0.029732
**GO:0034418**	urate biosynthetic process	1	172	2	11484	0.029732
**GO:0035411**	catenin import into nucleus	1	172	2	11484	0.029732
**GO:0036021**	endolysosome lumen	1	172	2	11484	0.029732
**GO:0043259**	laminin-10 complex	1	172	2	11484	0.029732
**GO:0043260**	laminin-11 complex	1	172	2	11484	0.029732
**GO:0043503**	skeletal muscle fiber adaptation	1	172	2	11484	0.029732
**GO:0045103**	intermediate filament-based process	1	172	2	11484	0.029732
**GO:0045187**	regulation of circadian sleep/wake cycle, sleep	1	172	2	11484	0.029732
**GO:0045636**	positive regulation of melanocyte differentiation	1	172	2	11484	0.029732
**GO:0045844**	positive regulation of striated muscle tissue development	1	172	2	11484	0.029732
**GO:0046320**	regulation of fatty acid oxidation	1	172	2	11484	0.029732
**GO:0046498**	S-adenosylhomocysteine metabolic process	1	172	2	11484	0.029732
**GO:0046499**	S-adenosylmethioninamine metabolic process	1	172	2	11484	0.029732
**GO:0047493**	ceramide cholinephosphotransferase activity	1	172	2	11484	0.029732
**GO:0048143**	astrocyte activation	1	172	2	11484	0.029732
**GO:0048659**	smooth muscle cell proliferation	1	172	2	11484	0.029732
**GO:0048667**	cell morphogenesis involved in neuron differentiation	1	172	2	11484	0.029732
**GO:0048769**	sarcomerogenesis	1	172	2	11484	0.029732
**GO:0051085**	chaperone mediated protein folding requiring cofactor	1	172	2	11484	0.029732
**GO:0051088**	PMA-inducible membrane protein ectodomain proteolysis	1	172	2	11484	0.029732

**GO:0051718**	DNA (cytosine-5-)-methyltransferase activity, acting on CpG substrates	1	172	2	11484	0.029732
**GO:0051782**	negative regulation of cell division	1	172	2	11484	0.029732
**GO:0052650**	NADP-retinol dehydrogenase activity	1	172	2	11484	0.029732
**GO:0060164**	regulation of timing of neuron differentiation	1	172	2	11484	0.029732
**GO:0060708**	spongiotrophoblast differentiation	1	172	2	11484	0.029732
**GO:0070293**	renal absorption	1	172	2	11484	0.029732
**GO:0070327**	thyroid hormone transport	1	172	2	11484	0.029732
**GO:0071578**	zinc ion transmembrane import	1	172	2	11484	0.029732
**GO:0071638**	negative regulation of monocyte chemotactic protein-1 production	1	172	2	11484	0.029732
**GO:0072012**	glomerulus vasculature development	1	172	2	11484	0.029732
**GO:0072108**	positive regulation of mesenchymal to epithelial transition involved in metanephros morphogenesis	1	172	2	11484	0.029732
**GO:0072144**	glomerular mesangial cell development	1	172	2	11484	0.029732
**GO:0072354**	histone kinase activity (H3-T3 specific)	1	172	2	11484	0.029732
**GO:0072542**	protein phosphatase activator activity	1	172	2	11484	0.029732
**GO:0090116**	C-5 methylation of cytosine	1	172	2	11484	0.029732
**GO:0090266**	regulation of mitotic cell cycle spindle assembly checkpoint	1	172	2	11484	0.029732
**GO:0090400**	stress-induced premature senescence	1	172	2	11484	0.029732
**GO:0097187**	dentinogenesis	1	172	2	11484	0.029732
**GO:1900016**	negative regulation of cytokine production involved in inflammatory response	1	172	2	11484	0.029732
**GO:1901844**	regulation of cell communication by electrical coupling involved in cardiac conduction	1	172	2	11484	0.029732
**GO:1902532**	negative regulation of intracellular signal transduction	1	172	2	11484	0.029732
**GO:1903016**	negative regulation of exo-alpha-sialidase activity	1	172	2	11484	0.029732

**GO:1903019**	negative regulation of glycoprotein metabolic process	1	172	2	11484	0.029732
**GO:2000278**	regulation of DNA biosynthetic process	1	172	2	11484	0.029732
**GO:2000978**	negative regulation of forebrain neuron differentiation	1	172	2	11484	0.029732
**GO:2000981**	negative regulation of inner ear receptor cell differentiation	1	172	2	11484	0.029732
**GO:0042803**	protein homodimerization activity	11	172	387	11484	0.031135
**GO:0030014**	CCR4-NOT complex	2	172	19	11484	0.032273
**GO:0030971**	receptor tyrosine kinase binding	2	172	19	11484	0.032273
**GO:0050873**	brown fat cell differentiation	2	172	19	11484	0.032273
**GO:0007517**	muscle organ development	3	172	48	11484	0.034928
**GO:0042060**	wound healing	3	172	48	11484	0.034928
**GO:0016310**	phosphorylation	5	172	121	11484	0.035255
**GO:0006402**	mRNA catabolic process	2	172	20	11484	0.035512
**GO:0009267**	cellular response to starvation	2	172	20	11484	0.035512
**GO:0034080**	centromere-specific nucleosome assembly	2	172	20	11484	0.035512
**GO:0005783**	endoplasmic reticulum	12	172	446	11484	0.035891
**GO:0016607**	nuclear speck	5	172	122	11484	0.036333
**GO:0005515**	protein binding	98	172	5733	11484	0.036767
**GO:0000122**	negative regulation of transcription from RNA polymerase II promoter	10	172	349	11484	0.037162
**GO:0031175**	neuron projection development	3	172	50	11484	0.03874
**GO:0002062**	chondrocyte differentiation	2	172	21	11484	0.038871
**GO:0048863**	stem cell differentiation	2	172	21	11484	0.038871
**GO:0072593**	reactive oxygen species metabolic process	2	172	21	11484	0.038871
**GO:0042127**	regulation of cell proliferation	4	172	86	11484	0.040019
**GO:0000979**	RNA polymerase II core promoter sequence-specific DNA binding	2	172	22	11484	0.042346
**GO:0003151**	outflow tract morphogenesis	2	172	22	11484	0.042346
**GO:0006306**	DNA methylation	2	172	22	11484	0.042346
**GO:0042771**	intrinsic apoptotic signaling pathway in response to DNA damage by p53 class mediator	2	172	22	11484	0.042346
**GO:0005178**	integrin binding	3	172	52	11484	0.042755
**GO:0001757**	somite specification	1	172	3	11484	0.044266
**GO:0001882**	nucleoside binding	1	172	3	11484	0.044266
**GO:0002070**	epithelial cell maturation	1	172	3	11484	0.044266
**GO:0002093**	auditory receptor cell morphogenesis	1	172	3	11484	0.044266

**GO:0002544**	chronic inflammatory response	1	172	3	11484	0.044266
**GO:0003331**	positive regulation of extracellular matrix constituent secretion	1	172	3	11484	0.044266
**GO:0003810**	protein-glutamine gamma-glutamyltransferase activity	1	172	3	11484	0.044266
**GO:0003886**	DNA (cytosine-5-)-methyltransferase activity	1	172	3	11484	0.044266
**GO:0004111**	creatine kinase activity	1	172	3	11484	0.044266
**GO:0004351**	glutamate decarboxylase activity	1	172	3	11484	0.044266
**GO:0004616**	phosphogluconate dehydrogenase (decarboxylating) activity	1	172	3	11484	0.044266
**GO:0004784**	superoxide dismutase activity	1	172	3	11484	0.044266
**GO:0005243**	gap junction channel activity	1	172	3	11484	0.044266
**GO:0005606**	laminin-1 complex	1	172	3	11484	0.044266
**GO:0005861**	troponin complex	1	172	3	11484	0.044266
**GO:0007021**	tubulin complex assembly	1	172	3	11484	0.044266
**GO:0007184**	SMAD protein import into nucleus	1	172	3	11484	0.044266
**GO:0007262**	STAT protein import into nucleus	1	172	3	11484	0.044266
**GO:0008065**	establishment of blood-nerve barrier	1	172	3	11484	0.044266
**GO:0009331**	glycerol-3-phosphate dehydrogenase complex	1	172	3	11484	0.044266
**GO:0010424**	DNA methylation on cytosine within a CG sequence	1	172	3	11484	0.044266
**GO:0010606**	positive regulation of cytoplasmic mRNA processing body assembly	1	172	3	11484	0.044266
**GO:0010886**	positive regulation of cholesterol storage	1	172	3	11484	0.044266
**GO:0014012**	peripheral nervous system axon regeneration	1	172	3	11484	0.044266
**GO:0014061**	regulation of norepinephrine secretion	1	172	3	11484	0.044266
**GO:0019563**	glycerol catabolic process	1	172	3	11484	0.044266
**GO:0019959**	interleukin-8 binding	1	172	3	11484	0.044266
**GO:0021587**	cerebellum morphogenesis	1	172	3	11484	0.044266
**GO:0022626**	cytosolic ribosome	1	172	3	11484	0.044266
**GO:0030485**	smooth muscle contractile fiber	1	172	3	11484	0.044266
**GO:0032387**	negative regulation of intracellular transport	1	172	3	11484	0.044266

**GO:0032876**	negative regulation of DNA endoreduplication	1	172	3	11484	0.044266
**GO:0033591**	response to L-ascorbic acid	1	172	3	11484	0.044266
**GO:0033629**	negative regulation of cell adhesion mediated by integrin	1	172	3	11484	0.044266
**GO:0035582**	sequestering of BMP in extracellular matrix	1	172	3	11484	0.044266
**GO:0035813**	regulation of renal sodium excretion	1	172	3	11484	0.044266
**GO:0035910**	ascending aorta morphogenesis	1	172	3	11484	0.044266
**GO:0036016**	cellular response to interleukin-3	1	172	3	11484	0.044266
**GO:0038031**	non-canonical Wnt signaling pathway via JNK cascade	1	172	3	11484	0.044266
**GO:0042304**	regulation of fatty acid biosynthetic process	1	172	3	11484	0.044266
**GO:0042308**	negative regulation of protein import into nucleus	1	172	3	11484	0.044266
**GO:0042403**	thyroid hormone metabolic process	1	172	3	11484	0.044266
**GO:0042511**	positive regulation of tyrosine phosphorylation of Stat1 protein	1	172	3	11484	0.044266
**GO:0042588**	zymogen granule	1	172	3	11484	0.044266
**GO:0042668**	auditory receptor cell fate determination	1	172	3	11484	0.044266
**GO:0042780**	tRNA 3'-end processing	1	172	3	11484	0.044266
**GO:0043353**	enucleate erythrocyte differentiation	1	172	3	11484	0.044266
**GO:0043426**	MRF binding	1	172	3	11484	0.044266
**GO:0043462**	regulation of ATPase activity	1	172	3	11484	0.044266
**GO:0044291**	cell-cell contact zone	1	172	3	11484	0.044266
**GO:0045347**	negative regulation of MHC class II biosynthetic process	1	172	3	11484	0.044266
**GO:0045590**	negative regulation of regulatory T cell differentiation	1	172	3	11484	0.044266
**GO:0045723**	positive regulation of fatty acid biosynthetic process	1	172	3	11484	0.044266
**GO:0045918**	negative regulation of cytolysis	1	172	3	11484	0.044266
**GO:0048144**	fibroblast proliferation	1	172	3	11484	0.044266
**GO:0048247**	lymphocyte chemotaxis	1	172	3	11484	0.044266
**GO:0048505**	regulation of timing of cell differentiation	1	172	3	11484	0.044266
**GO:0048592**	eye morphogenesis	1	172	3	11484	0.044266
**GO:0048861**	leukemia inhibitory factor signaling pathway	1	172	3	11484	0.044266

**GO:0051045**	negative regulation of membrane protein ectodomain proteolysis	1	172	3	11484	0.044266
**GO:0051387**	negative regulation of neurotrophin TRK receptor signaling pathway	1	172	3	11484	0.044266
**GO:0060020**	Bergmann glial cell differentiation	1	172	3	11484	0.044266
**GO:0060253**	negative regulation of glial cell proliferation	1	172	3	11484	0.044266
**GO:0060351**	cartilage development involved in endochondral bone morphogenesis	1	172	3	11484	0.044266
**GO:0060463**	lung lobe morphogenesis	1	172	3	11484	0.044266
**GO:0060574**	intestinal epithelial cell maturation	1	172	3	11484	0.044266
**GO:0061309**	cardiac neural crest cell development involved in outflow tract morphogenesis	1	172	3	11484	0.044266
**GO:0061337**	cardiac conduction	1	172	3	11484	0.044266
**GO:0070324**	thyroid hormone binding	1	172	3	11484	0.044266
**GO:0071280**	cellular response to copper ion	1	172	3	11484	0.044266
**GO:0071312**	cellular response to alkaloid	1	172	3	11484	0.044266
**GO:0071459**	protein localization to chromosome, centromeric region	1	172	3	11484	0.044266
**GO:0072049**	comma-shaped body morphogenesis	1	172	3	11484	0.044266
**GO:0072050**	S-shaped body morphogenesis	1	172	3	11484	0.044266
**GO:0072282**	metanephric nephron tubule morphogenesis	1	172	3	11484	0.044266
**GO:0072307**	regulation of metanephric nephron tubule epithelial cell differentiation	1	172	3	11484	0.044266
**GO:0090231**	regulation of spindle checkpoint	1	172	3	11484	0.044266
**GO:0097386**	glial cell projection	1	172	3	11484	0.044266
**GO:1900182**	positive regulation of protein localization to nucleus	1	172	3	11484	0.044266
**GO:1901214**	regulation of neuron death	1	172	3	11484	0.044266
**GO:1901385**	regulation of voltage-gated calcium channel activity	1	172	3	11484	0.044266
**GO:2000987**	positive regulation of behavioral fear response	1	172	3	11484	0.044266
**GO:2001022**	positive regulation of response to DNA damage stimulus	1	172	3	11484	0.044266
**GO:0009986**	cell surface	8	172	263	11484	0.044296

**GO:0005796**	Golgi lumen	3	172	53	11484	0.044838
**GO:0002053**	positive regulation of mesenchymal cell proliferation	2	172	23	11484	0.045932
**GO:0016525**	negative regulation of angiogenesis	2	172	23	11484	0.045932
**GO:0030514**	negative regulation of BMP signaling pathway	2	172	23	11484	0.045932
**GO:0051259**	protein oligomerization	2	172	23	11484	0.045932
**GO:0043123**	positive regulation of I-kappaB kinase/NF-kappaB signaling	4	172	91	11484	0.047601
**GO:0043005**	neuron projection	5	172	132	11484	0.048236
**GO:0071222**	cellular response to lipopolysaccharide	3	172	55	11484	0.049152
**GO:0016235**	aggresome	2	172	24	11484	0.049626
**GO:0021762**	substantia nigra development	2	172	24	11484	0.049626
**GO:0032024**	positive regulation of insulin secretion	2	172	24	11484	0.049626
**GO:0043066**	negative regulation of apoptotic process	9	172	319	11484	0.050639
**GO:0005507**	copper ion binding	2	172	25	11484	0.053423
**GO:0007519**	skeletal muscle tissue development	2	172	25	11484	0.053423
**GO:0047485**	protein N-terminus binding	3	172	57	11484	0.053662
**GO:0001077**	RNA polymerase II core promoter proximal region sequence-specific DNA binding transcription factor activity involved in positive regulation of transcription	4	172	96	11484	0.055925
**GO:0005200**	structural constituent of cytoskeleton	3	172	58	11484	0.055989
**GO:0008237**	metallopeptidase activity	3	172	58	11484	0.055989
**GO:0010033**	response to organic substance	3	172	58	11484	0.055989
**GO:0008284**	positive regulation of cell proliferation	8	172	277	11484	0.05678
**GO:0014068**	positive regulation of phosphatidylinositol 3-kinase signaling	2	172	26	11484	0.05732
**GO:0042157**	lipoprotein metabolic process	2	172	26	11484	0.05732
**GO:0038095**	Fc-epsilon receptor signaling pathway	4	172	97	11484	0.057678
**GO:0005814**	centriole	3	172	59	11484	0.058363
**GO:0001527**	microfibril	1	172	4	11484	0.058584
**GO:0001661**	conditioned taste aversion	1	172	4	11484	0.058584
**GO:0002018**	renin-angiotensin regulation of aldosterone production	1	172	4	11484	0.058584

**GO:0002523**	leukocyte migration involved in inflammatory response	1	172	4	11484	0.058584
**GO:0003266**	regulation of secondary heart field cardioblast proliferation	1	172	4	11484	0.058584
**GO:0003873**	6-phosphofructo-2-kinase activity	1	172	4	11484	0.058584
**GO:0004331**	fructose-2,6-bisphosphate 2-phosphatase activity	1	172	4	11484	0.058584
**GO:0004522**	pancreatic ribonuclease activity	1	172	4	11484	0.058584
**GO:0004740**	pyruvate dehydrogenase (acetyl-transferring) kinase activity	1	172	4	11484	0.058584
**GO:0005355**	glucose transmembrane transporter activity	1	172	4	11484	0.058584
**GO:0005865**	striated muscle thin filament	1	172	4	11484	0.058584
**GO:0006003**	fructose 2,6-bisphosphate metabolic process	1	172	4	11484	0.058584
**GO:0006686**	sphingomyelin biosynthetic process	1	172	4	11484	0.058584
**GO:0006883**	cellular sodium ion homeostasis	1	172	4	11484	0.058584
**GO:0007199**	G-protein coupled receptor signaling pathway coupled to cGMP nucleotide second messenger	1	172	4	11484	0.058584
**GO:0010469**	regulation of receptor activity	1	172	4	11484	0.058584
**GO:0010535**	positive regulation of activation of JAK2 kinase activity	1	172	4	11484	0.058584
**GO:0010544**	negative regulation of platelet activation	1	172	4	11484	0.058584
**GO:0010642**	negative regulation of platelet-derived growth factor receptor signaling pathway	1	172	4	11484	0.058584
**GO:0010762**	regulation of fibroblast migration	1	172	4	11484	0.058584
**GO:0010820**	positive regulation of T cell chemotaxis	1	172	4	11484	0.058584
**GO:0010890**	positive regulation of sequestering of triglyceride	1	172	4	11484	0.058584
**GO:0010955**	negative regulation of protein processing	1	172	4	11484	0.058584
**GO:0014824**	artery smooth muscle contraction	1	172	4	11484	0.058584
**GO:0016264**	gap junction assembly	1	172	4	11484	0.058584
**GO:0016628**	oxidoreductase activity, acting on the CH-CH group of donors, NAD or NADP as acceptor	1	172	4	11484	0.058584

**GO:0019932**	second-messenger-mediated signaling	1	172	4	11484	0.058584
**GO:0019992**	diacylglycerol binding	1	172	4	11484	0.058584
**GO:0021532**	neural tube patterning	1	172	4	11484	0.058584
**GO:0030239**	myofibril assembly	1	172	4	11484	0.058584
**GO:0030240**	skeletal muscle thin filament assembly	1	172	4	11484	0.058584
**GO:0030431**	sleep	1	172	4	11484	0.058584
**GO:0030595**	leukocyte chemotaxis	1	172	4	11484	0.058584
**GO:0030816**	positive regulation of cAMP metabolic process	1	172	4	11484	0.058584
**GO:0031669**	cellular response to nutrient levels	1	172	4	11484	0.058584
**GO:0032036**	myosin heavy chain binding	1	172	4	11484	0.058584
**GO:0032930**	positive regulation of superoxide anion generation	1	172	4	11484	0.058584
**GO:0033158**	regulation of protein import into nucleus, translocation	1	172	4	11484	0.058584
**GO:0033627**	cell adhesion mediated by integrin	1	172	4	11484	0.058584
**GO:0034755**	iron ion transmembrane transport	1	172	4	11484	0.058584
**GO:0035313**	wound healing, spreading of epidermal cells	1	172	4	11484	0.058584
**GO:0042503**	tyrosine phosphorylation of Stat3 protein	1	172	4	11484	0.058584
**GO:0042770**	signal transduction in response to DNA damage	1	172	4	11484	0.058584
**GO:0043374**	CD8-positive, alpha-beta T cell differentiation	1	172	4	11484	0.058584
**GO:0044782**	cilium organization	1	172	4	11484	0.058584
**GO:0045124**	regulation of bone resorption	1	172	4	11484	0.058584
**GO:0045162**	clustering of voltage-gated sodium channels	1	172	4	11484	0.058584
**GO:0045217**	cell-cell junction maintenance	1	172	4	11484	0.058584
**GO:0045322**	unmethylated CpG binding	1	172	4	11484	0.058584
**GO:0045656**	negative regulation of monocyte differentiation	1	172	4	11484	0.058584
**GO:0045835**	negative regulation of meiosis	1	172	4	11484	0.058584
**GO:0046822**	regulation of nucleocytoplasmic transport	1	172	4	11484	0.058584
**GO:0046850**	regulation of bone remodeling	1	172	4	11484	0.058584
**GO:0046888**	negative regulation of hormone secretion	1	172	4	11484	0.058584
**GO:0048260**	positive regulation of receptor-mediated endocytosis	1	172	4	11484	0.058584

**GO:0048642**	negative regulation of skeletal muscle tissue development	1	172	4	11484	0.058584
**GO:0050847**	progesterone receptor signaling pathway	1	172	4	11484	0.058584
**GO:0051343**	positive regulation of cyclic-nucleotide phosphodiesterase activity	1	172	4	11484	0.058584
**GO:0051549**	positive regulation of keratinocyte migration	1	172	4	11484	0.058584
**GO:0055009**	atrial cardiac muscle tissue morphogenesis	1	172	4	11484	0.058584
**GO:0060044**	negative regulation of cardiac muscle cell proliferation	1	172	4	11484	0.058584
**GO:0060298**	positive regulation of sarcomere organization	1	172	4	11484	0.058584
**GO:0060315**	negative regulation of ryanodine-sensitive calcium-release channel activity	1	172	4	11484	0.058584
**GO:0060371**	regulation of atrial cardiac muscle cell membrane depolarization	1	172	4	11484	0.058584
**GO:0060373**	regulation of ventricular cardiac muscle cell membrane depolarization	1	172	4	11484	0.058584
**GO:0060561**	apoptotic process involved in morphogenesis	1	172	4	11484	0.058584
**GO:0060628**	regulation of ER to Golgi vesicle-mediated transport	1	172	4	11484	0.058584
**GO:0061049**	cell growth involved in cardiac muscle cell development	1	172	4	11484	0.058584
**GO:0071253**	connexin binding	1	172	4	11484	0.058584
**GO:0097038**	perinuclear endoplasmic reticulum	1	172	4	11484	0.058584
**GO:0097084**	vascular smooth muscle cell development	1	172	4	11484	0.058584
**GO:2000178**	negative regulation of neural precursor cell proliferation	1	172	4	11484	0.058584
**GO:2000974**	negative regulation of pro-B cell differentiation	1	172	4	11484	0.058584
**GO:2001020**	regulation of response to DNA damage stimulus	1	172	4	11484	0.058584
**GO:0051260**	protein homooligomerization	4	172	98	11484	0.059461
**GO:0000902**	cell morphogenesis	3	172	60	11484	0.060785
**GO:0000978**	RNA polymerase II core promoter proximal region sequence-specific DNA binding	4	172	99	11484	0.061272

**GO:0004175**	endopeptidase activity	2	172	27	11484	0.061312
**GO:0009725**	response to hormone	2	172	27	11484	0.061312
**GO:0016049**	cell growth	2	172	27	11484	0.061312
**GO:0017148**	negative regulation of translation	2	172	27	11484	0.061312
**GO:0008360**	regulation of cell shape	3	172	61	11484	0.063252
**GO:0014704**	intercalated disc	2	172	28	11484	0.065396
**GO:0035019**	somatic stem cell maintenance	2	172	28	11484	0.065396
**GO:0060548**	negative regulation of cell death	2	172	28	11484	0.065396
**GO:0010468**	regulation of gene expression	4	172	103	11484	0.068808
**GO:0001658**	branching involved in ureteric bud morphogenesis	2	172	29	11484	0.069569
**GO:0010629**	negative regulation of gene expression	3	172	64	11484	0.070929
**GO:0030324**	lung development	3	172	64	11484	0.070929
**GO:0000307**	cyclin-dependent protein kinase holoenzyme complex	1	172	5	11484	0.072689
**GO:0001504**	neurotransmitter uptake	1	172	5	11484	0.072689
**GO:0002003**	angiotensin maturation	1	172	5	11484	0.072689
**GO:0002199**	zona pellucida receptor complex	1	172	5	11484	0.072689
**GO:0002819**	regulation of adaptive immune response	1	172	5	11484	0.072689
**GO:0003085**	negative regulation of systemic arterial blood pressure	1	172	5	11484	0.072689
**GO:0003214**	cardiac left ventricle morphogenesis	1	172	5	11484	0.072689
**GO:0004514**	nicotinate-nucleotide diphosphorylase (carboxylating) activity	1	172	5	11484	0.072689
**GO:0006072**	glycerol-3-phosphate metabolic process	1	172	5	11484	0.072689
**GO:0006600**	creatine metabolic process	1	172	5	11484	0.072689
**GO:0008154**	actin polymerization or depolymerization	1	172	5	11484	0.072689
**GO:0010941**	regulation of cell death	1	172	5	11484	0.072689
**GO:0015867**	ATP transport	1	172	5	11484	0.072689
**GO:0016504**	peptidase activator activity	1	172	5	11484	0.072689
**GO:0021861**	forebrain radial glial cell differentiation	1	172	5	11484	0.072689
**GO:0030432**	peristalsis	1	172	5	11484	0.072689
**GO:0032873**	negative regulation of stress-activated MAPK cascade	1	172	5	11484	0.072689
**GO:0034374**	low-density lipoprotein particle remodeling	1	172	5	11484	0.072689
**GO:0034405**	response to fluid shear stress	1	172	5	11484	0.072689

**GO:0035024**	negative regulation of Rho protein signal transduction	1	172	5	11484	0.072689
**GO:0035025**	positive regulation of Rho protein signal transduction	1	172	5	11484	0.072689
**GO:0035307**	positive regulation of protein dephosphorylation	1	172	5	11484	0.072689
**GO:0036094**	small molecule binding	1	172	5	11484	0.072689
**GO:0042473**	outer ear morphogenesis	1	172	5	11484	0.072689
**GO:0042627**	chylomicron	1	172	5	11484	0.072689
**GO:0042756**	drinking behavior	1	172	5	11484	0.072689
**GO:0043045**	DNA methylation involved in embryo development	1	172	5	11484	0.072689
**GO:0043208**	glycosphingolipid binding	1	172	5	11484	0.072689
**GO:0043394**	proteoglycan binding	1	172	5	11484	0.072689
**GO:0046339**	diacylglycerol metabolic process	1	172	5	11484	0.072689
**GO:0046697**	decidualization	1	172	5	11484	0.072689
**GO:0046790**	virion binding	1	172	5	11484	0.072689
**GO:0048070**	regulation of developmental pigmentation	1	172	5	11484	0.072689
**GO:0048644**	muscle organ morphogenesis	1	172	5	11484	0.072689
**GO:0051893**	regulation of focal adhesion assembly	1	172	5	11484	0.072689
**GO:0055013**	cardiac muscle cell development	1	172	5	11484	0.072689
**GO:0055123**	digestive system development	1	172	5	11484	0.072689
**GO:0060047**	heart contraction	1	172	5	11484	0.072689
**GO:0060395**	SMAD protein signal transduction	1	172	5	11484	0.072689
**GO:0060426**	lung vasculature development	1	172	5	11484	0.072689
**GO:0060541**	respiratory system development	1	172	5	11484	0.072689
**GO:0071480**	cellular response to gamma radiation	1	172	5	11484	0.072689
**GO:0071577**	zinc ion transmembrane transport	1	172	5	11484	0.072689
**GO:0090023**	positive regulation of neutrophil chemotaxis	1	172	5	11484	0.072689
**GO:0097197**	tetraspanin-enriched microdomain	1	172	5	11484	0.072689
**GO:1901216**	positive regulation of neuron death	1	172	5	11484	0.072689
**GO:2000279**	negative regulation of DNA biosynthetic process	1	172	5	11484	0.072689
**GO:0003714**	transcription corepressor activity	4	172	105	11484	0.072748
**GO:0007202**	activation of phospholipase C activity	2	172	30	11484	0.073827

**GO:0019827**	stem cell maintenance	2	172	30	11484	0.073827
**GO:0035690**	cellular response to drug	2	172	30	11484	0.073827
**GO:0045665**	negative regulation of neuron differentiation	2	172	30	11484	0.073827
**GO:0045732**	positive regulation of protein catabolic process	2	172	30	11484	0.073827
**GO:0046330**	positive regulation of JNK cascade	2	172	30	11484	0.073827
**GO:0060348**	bone development	2	172	30	11484	0.073827
**GO:0006200**	ATP catabolic process	6	172	198	11484	0.077224
**GO:0000228**	nuclear chromosome	2	172	31	11484	0.078166
**GO:0006921**	cellular component disassembly involved in execution phase of apoptosis	2	172	31	11484	0.078166
**GO:0008236**	serine-type peptidase activity	2	172	31	11484	0.078166
**GO:0030204**	chondroitin sulfate metabolic process	2	172	31	11484	0.078166
**GO:0031072**	heat shock protein binding	2	172	31	11484	0.078166
**GO:0031100**	organ regeneration	2	172	31	11484	0.078166
**GO:0051291**	protein heterooligomerization	2	172	31	11484	0.078166
**GO:0005925**	focal adhesion	3	172	67	11484	0.079003
**GO:0043647**	inositol phosphate metabolic process	2	172	32	11484	0.082583
**GO:0006629**	lipid metabolic process	4	172	111	11484	0.085237
**GO:0001849**	complement component C1q binding	1	172	6	11484	0.086584
**GO:0002237**	response to molecule of bacterial origin	1	172	6	11484	0.086584
**GO:0003014**	renal system process	1	172	6	11484	0.086584
**GO:0003143**	embryonic heart tube morphogenesis	1	172	6	11484	0.086584
**GO:0004012**	phospholipid-translocating ATPase activity	1	172	6	11484	0.086584
**GO:0004806**	triglyceride lipase activity	1	172	6	11484	0.086584
**GO:0005385**	zinc ion transmembrane transporter activity	1	172	6	11484	0.086584
**GO:0005501**	retinoid binding	1	172	6	11484	0.086584
**GO:0005798**	Golgi-associated vesicle	1	172	6	11484	0.086584
**GO:0006282**	regulation of DNA repair	1	172	6	11484	0.086584
**GO:0006346**	methylation-dependent chromatin silencing	1	172	6	11484	0.086584
**GO:0007062**	sister chromatid cohesion	1	172	6	11484	0.086584
**GO:0007064**	mitotic sister chromatid cohesion	1	172	6	11484	0.086584
**GO:0007589**	body fluid secretion	1	172	6	11484	0.086584
**GO:0008228**	opsonization	1	172	6	11484	0.086584

**GO:0008762**	UDP-N-acetylmuramate dehydrogenase activity	1	172	6	11484	0.086584
**GO:0009268**	response to pH	1	172	6	11484	0.086584
**GO:0010873**	positive regulation of cholesterol esterification	1	172	6	11484	0.086584
**GO:0010952**	positive regulation of peptidase activity	1	172	6	11484	0.086584
**GO:0014002**	astrocyte development	1	172	6	11484	0.086584
**GO:0016004**	phospholipase activator activity	1	172	6	11484	0.086584
**GO:0016614**	oxidoreductase activity, acting on CH-OH group of donors	1	172	6	11484	0.086584
**GO:0018149**	peptide cross-linking	1	172	6	11484	0.086584
**GO:0019430**	removal of superoxide radicals	1	172	6	11484	0.086584
**GO:0030660**	Golgi-associated vesicle membrane	1	172	6	11484	0.086584
**GO:0031005**	filamin binding	1	172	6	11484	0.086584
**GO:0031639**	plasminogen activation	1	172	6	11484	0.086584
**GO:0032465**	regulation of cytokinesis	1	172	6	11484	0.086584
**GO:0034698**	response to gonadotropin	1	172	6	11484	0.086584
**GO:0035050**	embryonic heart tube development	1	172	6	11484	0.086584
**GO:0042301**	phosphate ion binding	1	172	6	11484	0.086584
**GO:0043303**	mast cell degranulation	1	172	6	11484	0.086584
**GO:0043950**	positive regulation of cAMP-mediated signaling	1	172	6	11484	0.086584
**GO:0045109**	intermediate filament organization	1	172	6	11484	0.086584
**GO:0045742**	positive regulation of epidermal growth factor receptor signaling pathway	1	172	6	11484	0.086584
**GO:0046622**	positive regulation of organ growth	1	172	6	11484	0.086584
**GO:0046638**	positive regulation of alpha-beta T cell differentiation	1	172	6	11484	0.086584
**GO:0046967**	cytosol to ER transport	1	172	6	11484	0.086584
**GO:0048739**	cardiac muscle fiber development	1	172	6	11484	0.086584
**GO:0050687**	negative regulation of defense response to virus	1	172	6	11484	0.086584
**GO:0050708**	regulation of protein secretion	1	172	6	11484	0.086584
**GO:0051593**	response to folic acid	1	172	6	11484	0.086584
**GO:0051901**	positive regulation of mitochondrial depolarization	1	172	6	11484	0.086584
**GO:0060316**	positive regulation of ryanodine-sensitive calcium-release channel activity	1	172	6	11484	0.086584

**GO:0070307**	lens fiber cell development	1	172	6	11484	0.086584
**GO:0070997**	neuron death	1	172	6	11484	0.086584
**GO:0072358**	cardiovascular system development	1	172	6	11484	0.086584
**GO:0086014**	atrial cardiac muscle cell action potential	1	172	6	11484	0.086584
**GO:1901029**	negative regulation of mitochondrial outer membrane permeabilization involved in apoptotic signaling pathway	1	172	6	11484	0.086584
**GO:2000114**	regulation of establishment of cell polarity	1	172	6	11484	0.086584
**GO:2000737**	negative regulation of stem cell differentiation	1	172	6	11484	0.086584
**GO:2000810**	regulation of tight junction assembly	1	172	6	11484	0.086584
**GO:0006956**	complement activation	2	172	33	11484	0.087075
**GO:0045111**	intermediate filament cytoskeleton	2	172	33	11484	0.087075
**GO:0004222**	metalloendopeptidase activity	3	172	71	11484	0.09036
**GO:0006879**	cellular iron ion homeostasis	2	172	34	11484	0.091639
**GO:0034097**	response to cytokine	2	172	34	11484	0.091639
**GO:0007420**	brain development	4	172	115	11484	0.094108
**GO:0007155**	cell adhesion	7	172	259	11484	0.094521
**GO:0003700**	sequence-specific DNA binding transcription factor activity	14	172	636	11484	0.095653
**GO:0051592**	response to calcium ion	2	172	35	11484	0.096271
**GO:0006468**	protein phosphorylation	9	172	365	11484	0.097811
**GO:0006935**	chemotaxis	3	172	74	11484	0.099299
**GO:0016477**	cell migration	3	172	74	11484	0.099299
**GO:0000302**	response to reactive oxygen species	1	172	7	11484	0.100272
**GO:0001953**	negative regulation of cell-matrix adhesion	1	172	7	11484	0.100272
**GO:0006516**	glycoprotein catabolic process	1	172	7	11484	0.100272
**GO:0006878**	cellular copper ion homeostasis	1	172	7	11484	0.100272
**GO:0006937**	regulation of muscle contraction	1	172	7	11484	0.100272
**GO:0007095**	mitotic G2 DNA damage checkpoint	1	172	7	11484	0.100272
**GO:0008191**	metalloendopeptidase inhibitor activity	1	172	7	11484	0.100272
**GO:0009888**	tissue development	1	172	7	11484	0.100272
**GO:0010226**	response to lithium ion	1	172	7	11484	0.100272
**GO:0010288**	response to lead ion	1	172	7	11484	0.100272

**GO:0010880**	regulation of release of sequestered calcium ion into cytosol by sarcoplasmic reticulum	1	172	7	11484	0.100272
**GO:0015197**	peptide transporter activity	1	172	7	11484	0.100272
**GO:0016892**	endoribonuclease activity, producing 3'-phosphomonoesters	1	172	7	11484	0.100272
**GO:0017134**	fibroblast growth factor binding	1	172	7	11484	0.100272
**GO:0021984**	adenohypophysis development	1	172	7	11484	0.100272
**GO:0030194**	positive regulation of blood coagulation	1	172	7	11484	0.100272
**GO:0032516**	positive regulation of phosphoprotein phosphatase activity	1	172	7	11484	0.100272
**GO:0033147**	negative regulation of intracellular estrogen receptor signaling pathway	1	172	7	11484	0.100272
**GO:0035458**	cellular response to interferon-beta	1	172	7	11484	0.100272
**GO:0042043**	neurexin family protein binding	1	172	7	11484	0.100272
**GO:0042987**	amyloid precursor protein catabolic process	1	172	7	11484	0.100272
**GO:0043046**	DNA methylation involved in gamete generation	1	172	7	11484	0.100272
**GO:0043403**	skeletal muscle tissue regeneration	1	172	7	11484	0.100272
**GO:0048715**	negative regulation of oligodendrocyte differentiation	1	172	7	11484	0.100272
**GO:0048844**	artery morphogenesis	1	172	7	11484	0.100272
**GO:0050710**	negative regulation of cytokine secretion	1	172	7	11484	0.100272
**GO:0050880**	regulation of blood vessel size	1	172	7	11484	0.100272
**GO:0051016**	barbed-end actin filament capping	1	172	7	11484	0.100272
**GO:0051770**	positive regulation of nitric-oxide synthase biosynthetic process	1	172	7	11484	0.100272
**GO:0051895**	negative regulation of focal adhesion assembly	1	172	7	11484	0.100272
**GO:0051968**	positive regulation of synaptic transmission, glutamatergic	1	172	7	11484	0.100272
**GO:0055117**	regulation of cardiac muscle contraction	1	172	7	11484	0.100272
**GO:0060411**	cardiac septum morphogenesis	1	172	7	11484	0.100272

**GO:0061028**	establishment of endothelial barrier	1	172	7	11484	0.100272
**GO:0070848**	response to growth factor	1	172	7	11484	0.100272
**GO:0071243**	cellular response to arsenic-containing substance	1	172	7	11484	0.100272
**GO:0007219**	Notch signaling pathway	3	172	76	11484	0.105448
**GO:0060333**	interferon-gamma-mediated signaling pathway	3	172	76	11484	0.105448
**GO:0009749**	response to glucose	2	172	37	11484	0.105728
**GO:0050728**	negative regulation of inflammatory response	2	172	37	11484	0.105728
**GO:0000723**	telomere maintenance	2	172	38	11484	0.110548
**GO:0000775**	chromosome, centromeric region	2	172	38	11484	0.110548
**GO:0001558**	regulation of cell growth	2	172	38	11484	0.110548
**GO:0005901**	caveola	2	172	38	11484	0.110548
**GO:0043154**	negative regulation of cysteine-type endopeptidase activity involved in apoptotic process	2	172	38	11484	0.110548
**GO:0043410**	positive regulation of MAPK cascade	2	172	38	11484	0.110548
**GO:0044297**	cell body	2	172	38	11484	0.110548
**GO:0016023**	cytoplasmic membrane-bounded vesicle	3	172	78	11484	0.111744
**GO:0004745**	retinol dehydrogenase activity	1	172	8	11484	0.113755
**GO:0004861**	cyclin-dependent protein serine/threonine kinase inhibitor activity	1	172	8	11484	0.113755
**GO:0005614**	interstitial matrix	1	172	8	11484	0.113755
**GO:0006000**	fructose metabolic process	1	172	8	11484	0.113755
**GO:0006098**	pentose-phosphate shunt	1	172	8	11484	0.113755
**GO:0006536**	glutamate metabolic process	1	172	8	11484	0.113755
**GO:0006801**	superoxide metabolic process	1	172	8	11484	0.113755
**GO:0006970**	response to osmotic stress	1	172	8	11484	0.113755
**GO:0007597**	blood coagulation, intrinsic pathway	1	172	8	11484	0.113755
**GO:0008064**	regulation of actin polymerization or depolymerization	1	172	8	11484	0.113755
**GO:0010613**	positive regulation of cardiac muscle hypertrophy	1	172	8	11484	0.113755
**GO:0010801**	negative regulation of peptidyl-threonine phosphorylation	1	172	8	11484	0.113755

**GO:0010881**	regulation of cardiac muscle contraction by regulation of the release of sequestered calcium ion	1	172	8	11484	0.113755
**GO:0012501**	programmed cell death	1	172	8	11484	0.113755
**GO:0014912**	negative regulation of smooth muscle cell migration	1	172	8	11484	0.113755
**GO:0015075**	ion transmembrane transporter activity	1	172	8	11484	0.113755
**GO:0015833**	peptide transport	1	172	8	11484	0.113755
**GO:0021954**	central nervous system neuron development	1	172	8	11484	0.113755
**GO:0023029**	MHC class Ib protein binding	1	172	8	11484	0.113755
**GO:0030667**	secretory granule membrane	1	172	8	11484	0.113755
**GO:0030728**	ovulation	1	172	8	11484	0.113755
**GO:0031581**	hemidesmosome assembly	1	172	8	11484	0.113755
**GO:0031988**	membrane-bounded vesicle	1	172	8	11484	0.113755
**GO:0035066**	positive regulation of histone acetylation	1	172	8	11484	0.113755
**GO:0042119**	neutrophil activation	1	172	8	11484	0.113755
**GO:0042311**	vasodilation	1	172	8	11484	0.113755
**GO:0042989**	sequestering of actin monomers	1	172	8	11484	0.113755
**GO:0043068**	positive regulation of programmed cell death	1	172	8	11484	0.113755
**GO:0043422**	protein kinase B binding	1	172	8	11484	0.113755
**GO:0045598**	regulation of fat cell differentiation	1	172	8	11484	0.113755
**GO:0045744**	negative regulation of G-protein coupled receptor protein signaling pathway	1	172	8	11484	0.113755
**GO:0046685**	response to arsenic-containing substance	1	172	8	11484	0.113755
**GO:0046855**	inositol phosphate dephosphorylation	1	172	8	11484	0.113755
**GO:0048246**	macrophage chemotaxis	1	172	8	11484	0.113755
**GO:0048738**	cardiac muscle tissue development	1	172	8	11484	0.113755
**GO:0050872**	white fat cell differentiation	1	172	8	11484	0.113755
**GO:0051497**	negative regulation of stress fiber assembly	1	172	8	11484	0.113755
**GO:0051894**	positive regulation of focal adhesion assembly	1	172	8	11484	0.113755
**GO:0055093**	response to hyperoxia	1	172	8	11484	0.113755
**GO:0060135**	maternal process involved in female pregnancy	1	172	8	11484	0.113755

**GO:0061098**	positive regulation of protein tyrosine kinase activity	1	172	8	11484	0.113755
**GO:0070328**	triglyceride homeostasis	1	172	8	11484	0.113755
**GO:0071850**	mitotic cell cycle arrest	1	172	8	11484	0.113755
**GO:0072657**	protein localization to membrane	1	172	8	11484	0.113755
**GO:0090303**	positive regulation of wound healing	1	172	8	11484	0.113755
**GO:1900153**	positive regulation of nuclear-transcribed mRNA catabolic process, deadenylation-dependent decay	1	172	8	11484	0.113755
**GO:1901653**	cellular response to peptide	1	172	8	11484	0.113755
**GO:2000573**	positive regulation of DNA biosynthetic process	1	172	8	11484	0.113755
**GO:0008104**	protein localization	2	172	39	11484	0.115424
**GO:0045165**	cell fate commitment	2	172	39	11484	0.115424
**GO:0071356**	cellular response to tumor necrosis factor	2	172	39	11484	0.115424
**GO:0061024**	membrane organization	3	172	80	11484	0.11818
**GO:0007265**	Ras protein signal transduction	2	172	40	11484	0.120354
**GO:0048661**	positive regulation of smooth muscle cell proliferation	2	172	40	11484	0.120354
**GO:0006979**	response to oxidative stress	3	172	81	11484	0.121449
**GO:0020037**	heme binding	3	172	81	11484	0.121449
**GO:0008134**	transcription factor binding	5	172	175	11484	0.12254
**GO:0015030**	Cajal body	2	172	41	11484	0.125336
**GO:0060271**	cilium morphogenesis	2	172	41	11484	0.125336
**GO:0006915**	apoptotic process	9	172	386	11484	0.125757
**GO:0019899**	enzyme binding	5	172	177	11484	0.126858
**GO:0000975**	regulatory region DNA binding	1	172	9	11484	0.127038
**GO:0003746**	translation elongation factor activity	1	172	9	11484	0.127038
**GO:0004535**	poly(A)-specific ribonuclease activity	1	172	9	11484	0.127038
**GO:0004620**	phospholipase activity	1	172	9	11484	0.127038
**GO:0005513**	detection of calcium ion	1	172	9	11484	0.127038
**GO:0005771**	multivesicular body	1	172	9	11484	0.127038
**GO:0006195**	purine nucleotide catabolic process	1	172	9	11484	0.127038
**GO:0006796**	phosphate-containing compound metabolic process	1	172	9	11484	0.127038
**GO:0009435**	NAD biosynthetic process	1	172	9	11484	0.127038
**GO:0010977**	negative regulation of neuron projection development	1	172	9	11484	0.127038
**GO:0016125**	sterol metabolic process	1	172	9	11484	0.127038
**GO:0016595**	glutamate binding	1	172	9	11484	0.127038

**GO:0019060**	intracellular transport of viral protein in host cell	1	172	9	11484	0.127038
**GO:0019216**	regulation of lipid metabolic process	1	172	9	11484	0.127038
**GO:0019371**	cyclooxygenase pathway	1	172	9	11484	0.127038
**GO:0021542**	dentate gyrus development	1	172	9	11484	0.127038
**GO:0030207**	chondroitin sulfate catabolic process	1	172	9	11484	0.127038
**GO:0030208**	dermatan sulfate biosynthetic process	1	172	9	11484	0.127038
**GO:0030277**	maintenance of gastrointestinal epithelium	1	172	9	11484	0.127038
**GO:0030278**	regulation of ossification	1	172	9	11484	0.127038
**GO:0030500**	regulation of bone mineralization	1	172	9	11484	0.127038
**GO:0030866**	cortical actin cytoskeleton organization	1	172	9	11484	0.127038
**GO:0031430**	M band	1	172	9	11484	0.127038
**GO:0031996**	thioesterase binding	1	172	9	11484	0.127038
**GO:0032270**	positive regulation of cellular protein metabolic process	1	172	9	11484	0.127038
**GO:0033137**	negative regulation of peptidyl-serine phosphorylation	1	172	9	11484	0.127038
**GO:0034185**	apolipoprotein binding	1	172	9	11484	0.127038
**GO:0035815**	positive regulation of renal sodium excretion	1	172	9	11484	0.127038
**GO:0035902**	response to immobilization stress	1	172	9	11484	0.127038
**GO:0042491**	auditory receptor cell differentiation	1	172	9	11484	0.127038
**GO:0042825**	TAP complex	1	172	9	11484	0.127038
**GO:0043101**	purine-containing compound salvage	1	172	9	11484	0.127038
**GO:0043274**	phospholipase binding	1	172	9	11484	0.127038
**GO:0043408**	regulation of MAPK cascade	1	172	9	11484	0.127038
**GO:0045084**	positive regulation of interleukin-12 biosynthetic process	1	172	9	11484	0.127038
**GO:0045332**	phospholipid translocation	1	172	9	11484	0.127038
**GO:0045662**	negative regulation of myoblast differentiation	1	172	9	11484	0.127038
**GO:0045773**	positive regulation of axon extension	1	172	9	11484	0.127038
**GO:0045777**	positive regulation of blood pressure	1	172	9	11484	0.127038
**GO:0048741**	skeletal muscle fiber development	1	172	9	11484	0.127038

**GO:0050930**	induction of positive chemotaxis	1	172	9	11484	0.127038
**GO:0051090**	regulation of sequence-specific DNA binding transcription factor activity	1	172	9	11484	0.127038
**GO:0051926**	negative regulation of calcium ion transport	1	172	9	11484	0.127038
**GO:0060307**	regulation of ventricular cardiac muscle cell membrane repolarization	1	172	9	11484	0.127038
**GO:0070206**	protein trimerization	1	172	9	11484	0.127038
**GO:0071285**	cellular response to lithium ion	1	172	9	11484	0.127038
**GO:0071364**	cellular response to epidermal growth factor stimulus	1	172	9	11484	0.127038
**GO:0097150**	neuronal stem cell maintenance	1	172	9	11484	0.127038
**GO:0008543**	fibroblast growth factor receptor signaling pathway	3	172	83	11484	0.128086
**GO:0007224**	smoothened signaling pathway	2	172	42	11484	0.130366
**GO:0030307**	positive regulation of cell growth	2	172	42	11484	0.130366
**GO:0045087**	innate immune response	9	172	392	11484	0.13446
**GO:0006366**	transcription from RNA polymerase II promoter	7	172	284	11484	0.134593
**GO:0006325**	chromatin organization	3	172	85	11484	0.13485
**GO:0051092**	positive regulation of NF-kappaB transcription factor activity	3	172	85	11484	0.13485
**GO:0000242**	pericentriolar material	1	172	10	11484	0.140123
**GO:0001205**	RNA polymerase II distal enhancer sequence-specific DNA binding transcription factor activity involved in positive regulation of transcription	1	172	10	11484	0.140123
**GO:0009968**	negative regulation of signal transduction	1	172	10	11484	0.140123
**GO:0010038**	response to metal ion	1	172	10	11484	0.140123
**GO:0010510**	regulation of acetyl-CoA biosynthetic process from pyruvate	1	172	10	11484	0.140123
**GO:0019674**	NAD metabolic process	1	172	10	11484	0.140123
**GO:0019885**	antigen processing and presentation of endogenous peptide antigen via MHC class I	1	172	10	11484	0.140123
**GO:0030502**	negative regulation of bone mineralization	1	172	10	11484	0.140123
**GO:0030863**	cortical cytoskeleton	1	172	10	11484	0.140123

**GO:0031702**	type 1 angiotensin receptor binding	1	172	10	11484	0.140123
**GO:0031941**	filamentous actin	1	172	10	11484	0.140123
**GO:0032757**	positive regulation of interleukin-8 production	1	172	10	11484	0.140123
**GO:0033268**	node of Ranvier	1	172	10	11484	0.140123
**GO:0034614**	cellular response to reactive oxygen species	1	172	10	11484	0.140123
**GO:0035909**	aorta morphogenesis	1	172	10	11484	0.140123
**GO:0042445**	hormone metabolic process	1	172	10	11484	0.140123
**GO:0042562**	hormone binding	1	172	10	11484	0.140123
**GO:0042730**	fibrinolysis	1	172	10	11484	0.140123
**GO:0045088**	regulation of innate immune response	1	172	10	11484	0.140123
**GO:0046873**	metal ion transmembrane transporter activity	1	172	10	11484	0.140123
**GO:0046979**	TAP2 binding	1	172	10	11484	0.140123
**GO:0048747**	muscle fiber development	1	172	10	11484	0.140123
**GO:0052689**	carboxylic ester hydrolase activity	1	172	10	11484	0.140123
**GO:0070371**	ERK1 and ERK2 cascade	1	172	10	11484	0.140123
**GO:0071398**	cellular response to fatty acid	1	172	10	11484	0.140123
**GO:0071889**	14-3-3 protein binding	1	172	10	11484	0.140123
**GO:0090102**	cochlea development	1	172	10	11484	0.140123
**GO:2000378**	negative regulation of reactive oxygen species metabolic process	1	172	10	11484	0.140123
**GO:2000811**	negative regulation of anoikis	1	172	10	11484	0.140123
**GO:0001726**	ruffle	2	172	44	11484	0.140562
**GO:0003774**	motor activity	2	172	44	11484	0.140562
**GO:0051897**	positive regulation of protein kinase B signaling	2	172	44	11484	0.140562
**GO:0001501**	skeletal system development	3	172	87	11484	0.141735
**GO:0005794**	Golgi apparatus	10	172	453	11484	0.142482
**GO:0005506**	iron ion binding	3	172	88	11484	0.145221
**GO:0030574**	collagen catabolic process	2	172	45	11484	0.145724
**GO:0001701**	in utero embryonic development	4	172	137	11484	0.149953
**GO:0001078**	RNA polymerase II core promoter proximal region sequence-specific DNA binding transcription factor activity involved in negative regulation of transcription	2	172	46	11484	0.150924
**GO:0008277**	regulation of G-protein coupled receptor protein signaling pathway	2	172	46	11484	0.150924

**GO:0042102**	positive regulation of T cell proliferation	2	172	46	11484	0.150924
**GO:0016301**	kinase activity	3	172	90	11484	0.152277
**GO:0001516**	prostaglandin biosynthetic process	1	172	11	11484	0.153013
**GO:0001829**	trophectodermal cell differentiation	1	172	11	11484	0.153013
**GO:0003785**	actin monomer binding	1	172	11	11484	0.153013
**GO:0005680**	anaphase-promoting complex	1	172	11	11484	0.153013
**GO:0005720**	nuclear heterochromatin	1	172	11	11484	0.153013
**GO:0005922**	connexon complex	1	172	11	11484	0.153013
**GO:0006807**	nitrogen compound metabolic process	1	172	11	11484	0.153013
**GO:0014003**	oligodendrocyte development	1	172	11	11484	0.153013
**GO:0030048**	actin filament-based movement	1	172	11	11484	0.153013
**GO:0030224**	monocyte differentiation	1	172	11	11484	0.153013
**GO:0032728**	positive regulation of interferon-beta production	1	172	11	11484	0.153013
**GO:0034364**	high-density lipoprotein particle	1	172	11	11484	0.153013
**GO:0034704**	calcium channel complex	1	172	11	11484	0.153013
**GO:0044344**	cellular response to fibroblast growth factor stimulus	1	172	11	11484	0.153013
**GO:0046209**	nitric oxide metabolic process	1	172	11	11484	0.153013
**GO:0046427**	positive regulation of JAK-STAT cascade	1	172	11	11484	0.153013
**GO:0046658**	anchored component of plasma membrane	1	172	11	11484	0.153013
**GO:0050767**	regulation of neurogenesis	1	172	11	11484	0.153013
**GO:0051443**	positive regulation of ubiquitin-protein transferase activity	1	172	11	11484	0.153013
**GO:0060707**	trophoblast giant cell differentiation	1	172	11	11484	0.153013
**GO:0060716**	labyrinthine layer blood vessel development	1	172	11	11484	0.153013
**GO:0071901**	negative regulation of protein serine/threonine kinase activity	1	172	11	11484	0.153013
**GO:0007399**	nervous system development	5	172	189	11484	0.15418
**GO:0060070**	canonical Wnt signaling pathway	2	172	47	11484	0.15616
**GO:0007165**	signal transduction	16	172	811	11484	0.156796
**GO:0019901**	protein kinase binding	5	172	192	11484	0.161369
**GO:0005921**	gap junction	1	172	12	11484	0.165711
**GO:0006349**	regulation of gene expression by genetic imprinting	1	172	12	11484	0.165711
**GO:0006957**	complement activation, alternative pathway	1	172	12	11484	0.165711

**GO:0008589**	regulation of smoothened signaling pathway	1	172	12	11484	0.165711
**GO:0008652**	cellular amino acid biosynthetic process	1	172	12	11484	0.165711
**GO:0009416**	response to light stimulus	1	172	12	11484	0.165711
**GO:0009651**	response to salt stress	1	172	12	11484	0.165711
**GO:0015093**	ferrous iron transmembrane transporter activity	1	172	12	11484	0.165711
**GO:0015684**	ferrous iron transport	1	172	12	11484	0.165711
**GO:0016779**	nucleotidyltransferase activity	1	172	12	11484	0.165711
**GO:0021904**	dorsal/ventral neural tube patterning	1	172	12	11484	0.165711
**GO:0022011**	myelination in peripheral nervous system	1	172	12	11484	0.165711
**GO:0031214**	biomineral tissue development	1	172	12	11484	0.165711
**GO:0042310**	vasoconstriction	1	172	12	11484	0.165711
**GO:0042622**	photoreceptor outer segment membrane	1	172	12	11484	0.165711
**GO:0043034**	costamere	1	172	12	11484	0.165711
**GO:0045736**	negative regulation of cyclin-dependent protein serine/threonine kinase activity	1	172	12	11484	0.165711
**GO:0048169**	regulation of long-term neuronal synaptic plasticity	1	172	12	11484	0.165711
**GO:0048598**	embryonic morphogenesis	1	172	12	11484	0.165711
**GO:0050766**	positive regulation of phagocytosis	1	172	12	11484	0.165711
**GO:0051225**	spindle assembly	1	172	12	11484	0.165711
**GO:0051276**	chromosome organization	1	172	12	11484	0.165711
**GO:0060412**	ventricular septum morphogenesis	1	172	12	11484	0.165711
**GO:0070412**	R-SMAD binding	1	172	12	11484	0.165711
**GO:2000377**	regulation of reactive oxygen species metabolic process	1	172	12	11484	0.165711
**GO:0004672**	protein kinase activity	7	172	302	11484	0.167893
**GO:0000786**	nucleosome	2	172	50	11484	0.172067
**GO:0005516**	calmodulin binding	3	172	96	11484	0.174065
**GO:0035556**	intracellular signal transduction	6	172	251	11484	0.17506
**GO:0009636**	response to toxic substance	2	172	51	11484	0.177428
**GO:0051607**	defense response to virus	3	172	97	11484	0.17778
**GO:0004180**	carboxypeptidase activity	1	172	13	11484	0.178219
**GO:0004683**	calmodulin-dependent protein kinase activity	1	172	13	11484	0.178219
**GO:0005540**	hyaluronic acid binding	1	172	13	11484	0.178219
**GO:0005980**	glycogen catabolic process	1	172	13	11484	0.178219
**GO:0006826**	iron ion transport	1	172	13	11484	0.178219

**GO:0007250**	activation of NF-kappaB-inducing kinase activity	1	172	13	11484	0.178219
**GO:0008015**	blood circulation	1	172	13	11484	0.178219
**GO:0010906**	regulation of glucose metabolic process	1	172	13	11484	0.178219
**GO:0017022**	myosin binding	1	172	13	11484	0.178219
**GO:0030193**	regulation of blood coagulation	1	172	13	11484	0.178219
**GO:0030206**	chondroitin sulfate biosynthetic process	1	172	13	11484	0.178219
**GO:0030332**	cyclin binding	1	172	13	11484	0.178219
**GO:0030539**	male genitalia development	1	172	13	11484	0.178219
**GO:0032007**	negative regulation of TOR signaling	1	172	13	11484	0.178219
**GO:0043209**	myelin sheath	1	172	13	11484	0.178219
**GO:0043388**	positive regulation of DNA binding	1	172	13	11484	0.178219
**GO:0045879**	negative regulation of smoothened signaling pathway	1	172	13	11484	0.178219
**GO:0046978**	TAP1 binding	1	172	13	11484	0.178219
**GO:0048514**	blood vessel morphogenesis	1	172	13	11484	0.178219
**GO:0048566**	embryonic digestive tract development	1	172	13	11484	0.178219
**GO:0050999**	regulation of nitric-oxide synthase activity	1	172	13	11484	0.178219
**GO:1900026**	positive regulation of substrate adhesion-dependent cell spreading	1	172	13	11484	0.178219
**GO:2000036**	regulation of stem cell maintenance	1	172	13	11484	0.178219
**GO:0048011**	neurotrophin TRK receptor signaling pathway	4	172	148	11484	0.18172
**GO:0006508**	proteolysis	7	172	309	11484	0.181752
**GO:0016772**	transferase activity, transferring phosphorus-containing groups	7	172	309	11484	0.181752
**GO:0006974**	cellular response to DNA damage stimulus	3	172	99	11484	0.185274
**GO:0005622**	intracellular	16	172	838	11484	0.188825
**GO:0001786**	phosphatidylserine binding	1	172	14	11484	0.190541
**GO:0001958**	endochondral ossification	1	172	14	11484	0.190541
**GO:0001975**	response to amphetamine	1	172	14	11484	0.190541
**GO:0003281**	ventricular septum development	1	172	14	11484	0.190541
**GO:0005154**	epidermal growth factor receptor binding	1	172	14	11484	0.190541
**GO:0005319**	lipid transporter activity	1	172	14	11484	0.190541
**GO:0006641**	triglyceride metabolic process	1	172	14	11484	0.190541
**GO:0006829**	zinc ion transport	1	172	14	11484	0.190541

**GO:0010332**	response to gamma radiation	1	172	14	11484	0.190541
**GO:0016627**	oxidoreductase activity, acting on the CH-CH group of donors	1	172	14	11484	0.190541
**GO:0030001**	metal ion transport	1	172	14	11484	0.190541
**GO:0030414**	peptidase inhibitor activity	1	172	14	11484	0.190541
**GO:0034361**	very-low-density lipoprotein particle	1	172	14	11484	0.190541
**GO:0042326**	negative regulation of phosphorylation	1	172	14	11484	0.190541
**GO:0042572**	retinol metabolic process	1	172	14	11484	0.190541
**GO:0045747**	positive regulation of Notch signaling pathway	1	172	14	11484	0.190541
**GO:0046597**	negative regulation of viral entry into host cell	1	172	14	11484	0.190541
**GO:0046686**	response to cadmium ion	1	172	14	11484	0.190541
**GO:0051145**	smooth muscle cell differentiation	1	172	14	11484	0.190541
**GO:0090026**	positive regulation of monocyte chemotaxis	1	172	14	11484	0.190541
**GO:0090190**	positive regulation of branching involved in ureteric bud morphogenesis	1	172	14	11484	0.190541
**GO:0090398**	cellular senescence	1	172	14	11484	0.190541
**GO:0005694**	chromosome	2	172	54	11484	0.193659
**GO:0042605**	peptide antigen binding	2	172	54	11484	0.193659
**GO:0008022**	protein C-terminus binding	3	172	102	11484	0.196667
**GO:0032403**	protein complex binding	3	172	102	11484	0.196667
**GO:0032259**	methylation	2	172	55	11484	0.199112
**GO:0005743**	mitochondrial inner membrane	5	172	207	11484	0.199222
**GO:0016071**	mRNA metabolic process	4	172	154	11484	0.199919
**GO:0001772**	immunological synapse	1	172	15	11484	0.20268
**GO:0001937**	negative regulation of endothelial cell proliferation	1	172	15	11484	0.20268
**GO:0004143**	diacylglycerol kinase activity	1	172	15	11484	0.20268
**GO:0005520**	insulin-like growth factor binding	1	172	15	11484	0.20268
**GO:0006090**	pyruvate metabolic process	1	172	15	11484	0.20268
**GO:0006509**	membrane protein ectodomain proteolysis	1	172	15	11484	0.20268
**GO:0006730**	one-carbon metabolic process	1	172	15	11484	0.20268
**GO:0006885**	regulation of pH	1	172	15	11484	0.20268
**GO:0007159**	leukocyte cell-cell adhesion	1	172	15	11484	0.20268
**GO:0007569**	cell aging	1	172	15	11484	0.20268
**GO:0009116**	nucleoside metabolic process	1	172	15	11484	0.20268
**GO:0016601**	Rac protein signal transduction	1	172	15	11484	0.20268
**GO:0019229**	regulation of vasoconstriction	1	172	15	11484	0.20268

**GO:0019829**	cation-transporting ATPase activity	1	172	15	11484	0.20268
**GO:0045184**	establishment of protein localization	1	172	15	11484	0.20268
**GO:0045930**	negative regulation of mitotic cell cycle	1	172	15	11484	0.20268
**GO:0048147**	negative regulation of fibroblast proliferation	1	172	15	11484	0.20268
**GO:0051297**	centrosome organization	1	172	15	11484	0.20268
**GO:0070491**	repressing transcription factor binding	1	172	15	11484	0.20268
**GO:2000134**	negative regulation of G1/S transition of mitotic cell cycle	1	172	15	11484	0.20268
**GO:0006396**	RNA processing	2	172	56	11484	0.204584
**GO:0001764**	neuron migration	2	172	57	11484	0.210072
**GO:0006417**	regulation of translation	2	172	57	11484	0.210072
**GO:0007275**	multicellular organismal development	7	172	323	11484	0.210841
**GO:0007601**	visual perception	3	172	106	11484	0.212115
**GO:0000096**	sulfur amino acid metabolic process	1	172	16	11484	0.214637
**GO:0001816**	cytokine production	1	172	16	11484	0.214637
**GO:0004181**	metallocarboxypeptidase activity	1	172	16	11484	0.214637
**GO:0007220**	Notch receptor processing	1	172	16	11484	0.214637
**GO:0008306**	associative learning	1	172	16	11484	0.214637
**GO:0019838**	growth factor binding	1	172	16	11484	0.214637
**GO:0021537**	telencephalon development	1	172	16	11484	0.214637
**GO:0022400**	regulation of rhodopsin mediated signaling pathway	1	172	16	11484	0.214637
**GO:0030513**	positive regulation of BMP signaling pathway	1	172	16	11484	0.214637
**GO:0030901**	midbrain development	1	172	16	11484	0.214637
**GO:0033141**	positive regulation of peptidyl-serine phosphorylation of STAT protein	1	172	16	11484	0.214637
**GO:0042474**	middle ear morphogenesis	1	172	16	11484	0.214637
**GO:0042476**	odontogenesis	1	172	16	11484	0.214637
**GO:0043200**	response to amino acid	1	172	16	11484	0.214637
**GO:0045880**	positive regulation of smoothened signaling pathway	1	172	16	11484	0.214637
**GO:0046835**	carbohydrate phosphorylation	1	172	16	11484	0.214637
**GO:0048568**	embryonic organ development	1	172	16	11484	0.214637
**GO:0048678**	response to axon injury	1	172	16	11484	0.214637
**GO:0070207**	protein homotrimerization	1	172	16	11484	0.214637

**GO:0070373**	negative regulation of ERK1 and ERK2 cascade	1	172	16	11484	0.214637
**GO:0003924**	GTPase activity	4	172	159	11484	0.215489
**GO:0006897**	endocytosis	2	172	58	11484	0.215575
**GO:0016605**	PML body	2	172	58	11484	0.215575
**GO:0042384**	cilium assembly	2	172	58	11484	0.215575
**GO:0034641**	cellular nitrogen compound metabolic process	3	172	107	11484	0.216018
**GO:0005215**	transporter activity	4	172	160	11484	0.218644
**GO:0003951**	NAD+ kinase activity	1	172	17	11484	0.226416
**GO:0006027**	glycosaminoglycan catabolic process	1	172	17	11484	0.226416
**GO:0006695**	cholesterol biosynthetic process	1	172	17	11484	0.226416
**GO:0021983**	pituitary gland development	1	172	17	11484	0.226416
**GO:0031016**	pancreas development	1	172	17	11484	0.226416
**GO:0031623**	receptor internalization	1	172	17	11484	0.226416
**GO:0032570**	response to progesterone	1	172	17	11484	0.226416
**GO:0040018**	positive regulation of multicellular organism growth	1	172	17	11484	0.226416
**GO:0042288**	MHC class I protein binding	1	172	17	11484	0.226416
**GO:0043484**	regulation of RNA splicing	1	172	17	11484	0.226416
**GO:0050829**	defense response to Gram-negative bacterium	1	172	17	11484	0.226416
**GO:0051496**	positive regulation of stress fiber assembly	1	172	17	11484	0.226416
**GO:0071333**	cellular response to glucose stimulus	1	172	17	11484	0.226416
**GO:0006184**	GTP catabolic process	4	172	163	11484	0.22818
**GO:0006357**	regulation of transcription from RNA polymerase II promoter	5	172	218	11484	0.228738
**GO:0002224**	toll-like receptor signaling pathway	2	172	61	11484	0.232157
**GO:0006414**	translational elongation	2	172	61	11484	0.232157
**GO:0005667**	transcription factor complex	3	172	112	11484	0.235751
**GO:0007204**	positive regulation of cytosolic calcium ion concentration	2	172	62	11484	0.237703
**GO:0043524**	negative regulation of neuron apoptotic process	2	172	62	11484	0.237703
**GO:0050900**	leukocyte migration	2	172	62	11484	0.237703
**GO:0000049**	tRNA binding	1	172	18	11484	0.23802
**GO:0000792**	heterochromatin	1	172	18	11484	0.23802
**GO:0001104**	RNA polymerase II transcription cofactor activity	1	172	18	11484	0.23802
**GO:0006024**	glycosaminoglycan biosynthetic process	1	172	18	11484	0.23802
**GO:0007492**	endoderm development	1	172	18	11484	0.23802

**GO:0010595**	positive regulation of endothelial cell migration	1	172	18	11484	0.23802
**GO:0015914**	phospholipid transport	1	172	18	11484	0.23802
**GO:0016056**	rhodopsin mediated signaling pathway	1	172	18	11484	0.23802
**GO:0030148**	sphingolipid biosynthetic process	1	172	18	11484	0.23802
**GO:0032870**	cellular response to hormone stimulus	1	172	18	11484	0.23802
**GO:0048701**	embryonic cranial skeleton morphogenesis	1	172	18	11484	0.23802
**GO:0005125**	cytokine activity	3	172	113	11484	0.239737
**GO:0000922**	spindle pole	2	172	63	11484	0.243256
**GO:0017124**	SH3 domain binding	2	172	63	11484	0.243256
**GO:0046983**	protein dimerization activity	3	172	115	11484	0.247741
**GO:0005802**	trans-Golgi network	2	172	64	11484	0.248815
**GO:0000060**	protein import into nucleus, translocation	1	172	19	11484	0.24945
**GO:0001541**	ovarian follicle development	1	172	19	11484	0.24945
**GO:0006479**	protein methylation	1	172	19	11484	0.24945
**GO:0016529**	sarcoplasmic reticulum	1	172	19	11484	0.24945
**GO:0034612**	response to tumor necrosis factor	1	172	19	11484	0.24945
**GO:0045668**	negative regulation of osteoblast differentiation	1	172	19	11484	0.24945
**GO:0048545**	response to steroid hormone	1	172	19	11484	0.24945
**GO:0048662**	negative regulation of smooth muscle cell proliferation	1	172	19	11484	0.24945
**GO:0051898**	negative regulation of protein kinase B signaling	1	172	19	11484	0.24945
**GO:0060048**	cardiac muscle contraction	1	172	19	11484	0.24945
**GO:0060349**	bone morphogenesis	1	172	19	11484	0.24945
**GO:0070888**	E-box binding	1	172	19	11484	0.24945
**GO:0071560**	cellular response to transforming growth factor beta stimulus	1	172	19	11484	0.24945
**GO:0090503**	RNA phosphodiester bond hydrolysis, exonucleolytic	1	172	19	11484	0.24945
**GO:2001141**	regulation of RNA biosynthetic process	1	172	19	11484	0.24945
**GO:0008219**	cell death	3	172	116	11484	0.251759
**GO:0055114**	oxidation-reduction process	8	172	401	11484	0.253276
**GO:0070374**	positive regulation of ERK1 and ERK2 cascade	2	172	65	11484	0.254378
**GO:0030203**	glycosaminoglycan metabolic process	2	172	66	11484	0.259944
**GO:0005044**	scavenger receptor activity	1	172	20	11484	0.26071

**GO:0007162**	negative regulation of cell adhesion	1	172	20	11484	0.26071
**GO:0031252**	cell leading edge	1	172	20	11484	0.26071
**GO:0032760**	positive regulation of tumor necrosis factor production	1	172	20	11484	0.26071
**GO:0034446**	substrate adhesion-dependent cell spreading	1	172	20	11484	0.26071
**GO:0042594**	response to starvation	1	172	20	11484	0.26071
**GO:0043679**	axon terminus	1	172	20	11484	0.26071
**GO:0045595**	regulation of cell differentiation	1	172	20	11484	0.26071
**GO:0046854**	phosphatidylinositol phosphorylation	1	172	20	11484	0.26071
**GO:0060173**	limb development	1	172	20	11484	0.26071
**GO:0051091**	positive regulation of sequence-specific DNA binding transcription factor activity	2	172	67	11484	0.265512
**GO:0006461**	protein complex assembly	2	172	68	11484	0.271079
**GO:0008168**	methyltransferase activity	2	172	68	11484	0.271079
**GO:0042742**	defense response to bacterium	2	172	68	11484	0.271079
**GO:0001047**	core promoter binding	1	172	21	11484	0.271802
**GO:0001819**	positive regulation of cytokine production	1	172	21	11484	0.271802
**GO:0005913**	cell-cell adherens junction	1	172	21	11484	0.271802
**GO:0007157**	heterophilic cell-cell adhesion	1	172	21	11484	0.271802
**GO:0007339**	binding of sperm to zona pellucida	1	172	21	11484	0.271802
**GO:0009411**	response to UV	1	172	21	11484	0.271802
**GO:0015485**	cholesterol binding	1	172	21	11484	0.271802
**GO:0016485**	protein processing	1	172	21	11484	0.271802
**GO:0034644**	cellular response to UV	1	172	21	11484	0.271802
**GO:0050661**	NADP binding	1	172	21	11484	0.271802
**GO:0050715**	positive regulation of cytokine secretion	1	172	21	11484	0.271802
**GO:0051258**	protein polymerization	1	172	21	11484	0.271802
**GO:0097191**	extrinsic apoptotic signaling pathway	1	172	21	11484	0.271802
**GO:0016070**	RNA metabolic process	4	172	177	11484	0.27392
**GO:0030182**	neuron differentiation	2	172	69	11484	0.276646
**GO:0005741**	mitochondrial outer membrane	2	172	70	11484	0.28221
**GO:0001656**	metanephros development	1	172	22	11484	0.282728
**GO:0001664**	G-protein coupled receptor binding	1	172	22	11484	0.282728
**GO:0001837**	epithelial to mesenchymal transition	1	172	22	11484	0.282728
**GO:0030315**	T-tubule	1	172	22	11484	0.282728

**GO:0030838**	positive regulation of actin filament polymerization	1	172	22	11484	0.282728
**GO:0030890**	positive regulation of B cell proliferation	1	172	22	11484	0.282728
**GO:0032091**	negative regulation of protein binding	1	172	22	11484	0.282728
**GO:0045785**	positive regulation of cell adhesion	1	172	22	11484	0.282728
**GO:0048286**	lung alveolus development	1	172	22	11484	0.282728
**GO:0048387**	negative regulation of retinoic acid receptor signaling pathway	1	172	22	11484	0.282728
**GO:0097192**	extrinsic apoptotic signaling pathway in absence of ligand	1	172	22	11484	0.282728
**GO:0006805**	xenobiotic metabolic process	2	172	72	11484	0.293327
**GO:0000289**	nuclear-transcribed mRNA poly(A) tail shortening	1	172	23	11484	0.293492
**GO:0004864**	protein phosphatase inhibitor activity	1	172	23	11484	0.293492
**GO:0005109**	frizzled binding	1	172	23	11484	0.293492
**GO:0007200**	phospholipase C-activating G-protein coupled receptor signaling pathway	1	172	23	11484	0.293492
**GO:0008047**	enzyme activator activity	1	172	23	11484	0.293492
**GO:0046332**	SMAD binding	1	172	23	11484	0.293492
**GO:0048487**	beta-tubulin binding	1	172	23	11484	0.293492
**GO:0048839**	inner ear development	1	172	23	11484	0.293492
**GO:0042802**	identical protein binding	6	172	300	11484	0.294046
**GO:0001948**	glycoprotein binding	1	172	24	11484	0.304095
**GO:0010212**	response to ionizing radiation	1	172	24	11484	0.304095
**GO:0016328**	lateral plasma membrane	1	172	24	11484	0.304095
**GO:0031018**	endocrine pancreas development	1	172	24	11484	0.304095
**GO:0034976**	response to endoplasmic reticulum stress	1	172	24	11484	0.304095
**GO:0035176**	social behavior	1	172	24	11484	0.304095
**GO:0048589**	developmental growth	1	172	24	11484	0.304095
**GO:0071230**	cellular response to amino acid stimulus	1	172	24	11484	0.304095
**GO:0005882**	intermediate filament	2	172	74	11484	0.304422
**GO:0007605**	sensory perception of sound	2	172	75	11484	0.309958
**GO:0006144**	purine nucleobase metabolic process	1	172	25	11484	0.314539
**GO:0007205**	protein kinase C-activating G-protein coupled receptor signaling pathway	1	172	25	11484	0.314539
**GO:0021915**	neural tube development	1	172	25	11484	0.314539

**GO:0030145**	manganese ion binding	1	172	25	11484	0.314539
**GO:0030173**	integral component of Golgi membrane	1	172	25	11484	0.314539
**GO:0031982**	vesicle	1	172	25	11484	0.314539
**GO:0051117**	ATPase binding	1	172	25	11484	0.314539
**GO:0071277**	cellular response to calcium ion	1	172	25	11484	0.314539
**GO:0030027**	lamellipodium	2	172	77	11484	0.321003
**GO:0019221**	cytokine-mediated signaling pathway	4	172	191	11484	0.32105
**GO:0008270**	zinc ion binding	13	172	744	11484	0.322994
**GO:0002250**	adaptive immune response	1	172	26	11484	0.324828
**GO:0005876**	spindle microtubule	1	172	26	11484	0.324828
**GO:0006094**	gluconeogenesis	1	172	26	11484	0.324828
**GO:0007588**	excretion	1	172	26	11484	0.324828
**GO:0009103**	lipopolysaccharide biosynthetic process	1	172	26	11484	0.324828
**GO:0031047**	gene silencing by RNA	1	172	26	11484	0.324828
**GO:0045893**	positive regulation of transcription, DNA-templated	6	172	314	11484	0.330895
**GO:0000910**	cytokinesis	1	172	27	11484	0.334963
**GO:0006096**	glycolytic process	1	172	27	11484	0.334963
**GO:0006958**	complement activation, classical pathway	1	172	27	11484	0.334963
**GO:0007017**	microtubule-based process	1	172	27	11484	0.334963
**GO:0019722**	calcium-mediated signaling	1	172	27	11484	0.334963
**GO:0048538**	thymus development	1	172	27	11484	0.334963
**GO:0008289**	lipid binding	2	172	80	11484	0.337487
**GO:0007010**	cytoskeleton organization	2	172	81	11484	0.342957
**GO:0001890**	placenta development	1	172	28	11484	0.344947
**GO:0003007**	heart morphogenesis	1	172	28	11484	0.344947
**GO:0005811**	lipid particle	1	172	28	11484	0.344947
**GO:0005884**	actin filament	1	172	28	11484	0.344947
**GO:0006633**	fatty acid biosynthetic process	1	172	28	11484	0.344947
**GO:0007154**	cell communication	1	172	28	11484	0.344947
**GO:0022625**	cytosolic large ribosomal subunit	1	172	28	11484	0.344947
**GO:0030041**	actin filament polymerization	1	172	28	11484	0.344947
**GO:0043588**	skin development	1	172	28	11484	0.344947
**GO:0045071**	negative regulation of viral genome replication	1	172	28	11484	0.344947
**GO:0048469**	cell maturation	1	172	28	11484	0.344947
**GO:0048706**	embryonic skeletal system development	1	172	28	11484	0.344947
**GO:0051603**	proteolysis involved in cellular protein catabolic process	1	172	28	11484	0.344947
**GO:0030529**	ribonucleoprotein complex	2	172	82	11484	0.348412
**GO:0003712**	transcription cofactor activity	1	172	29	11484	0.354782

**GO:0032880**	regulation of protein localization	1	172	29	11484	0.354782
**GO:0050727**	regulation of inflammatory response	1	172	29	11484	0.354782
**GO:0051781**	positive regulation of cell division	1	172	29	11484	0.354782
**GO:0000082**	G1/S transition of mitotic cell cycle	2	172	84	11484	0.359276
**GO:0006334**	nucleosome assembly	2	172	84	11484	0.359276
**GO:0000980**	RNA polymerase II distal enhancer sequence-specific DNA binding	1	172	30	11484	0.36447
**GO:0001568**	blood vessel development	1	172	30	11484	0.36447
**GO:0008092**	cytoskeletal protein binding	1	172	30	11484	0.36447
**GO:0009953**	dorsal/ventral pattern formation	1	172	30	11484	0.36447
**GO:0015758**	glucose transport	1	172	30	11484	0.36447
**GO:0005887**	integral component of plasma membrane	11	172	645	11484	0.372896
**GO:0000186**	activation of MAPKK activity	1	172	31	11484	0.374014
**GO:0004197**	cysteine-type endopeptidase activity	1	172	31	11484	0.374014
**GO:0005070**	SH3/SH2 adaptor activity	1	172	31	11484	0.374014
**GO:0006626**	protein targeting to mitochondrion	1	172	31	11484	0.374014
**GO:0007126**	meiotic nuclear division	1	172	31	11484	0.374014
**GO:0008202**	steroid metabolic process	1	172	31	11484	0.374014
**GO:0043204**	perikaryon	1	172	31	11484	0.374014
**GO:0071363**	cellular response to growth factor stimulus	1	172	31	11484	0.374014
**GO:0005758**	mitochondrial intermembrane space	1	172	32	11484	0.383415
**GO:0005930**	axoneme	1	172	32	11484	0.383415
**GO:0019369**	arachidonic acid metabolic process	1	172	32	11484	0.383415
**GO:0019432**	triglyceride biosynthetic process	1	172	32	11484	0.383415
**GO:0048666**	neuron development	1	172	32	11484	0.383415
**GO:0048704**	embryonic skeletal system morphogenesis	1	172	32	11484	0.383415
**GO:0050839**	cell adhesion molecule binding	1	172	32	11484	0.383415
**GO:0097193**	intrinsic apoptotic signaling pathway	1	172	32	11484	0.383415
**GO:0006355**	regulation of transcription, DNA-templated	18	172	1101	11484	0.383943
**GO:0001947**	heart looping	1	172	33	11484	0.392675
**GO:0005080**	protein kinase C binding	1	172	33	11484	0.392675
**GO:0007623**	circadian rhythm	1	172	33	11484	0.392675

**GO:0010923**	negative regulation of phosphatase activity	1	172	33	11484	0.392675
**GO:0022627**	cytosolic small ribosomal subunit	1	172	33	11484	0.392675
**GO:0030509**	BMP signaling pathway	1	172	33	11484	0.392675
**GO:0042734**	presynaptic membrane	1	172	33	11484	0.392675
**GO:0043280**	positive regulation of cysteine-type endopeptidase activity involved in apoptotic process	1	172	33	11484	0.392675
**GO:0045860**	positive regulation of protein kinase activity	1	172	33	11484	0.392675
**GO:0048812**	neuron projection morphogenesis	1	172	33	11484	0.392675
**GO:0004252**	serine-type endopeptidase activity	2	172	91	11484	0.396746
**GO:0042626**	ATPase activity, coupled to transmembrane movement of substances	1	172	34	11484	0.401798
**GO:0010467**	gene expression	7	172	405	11484	0.404479
**GO:0008283**	cell proliferation	4	172	217	11484	0.409526
**GO:0007389**	pattern specification process	1	172	35	11484	0.410784
**GO:0009967**	positive regulation of signal transduction	1	172	35	11484	0.410784
**GO:0038032**	termination of G-protein coupled receptor signaling pathway	1	172	35	11484	0.410784
**GO:0042632**	cholesterol homeostasis	1	172	35	11484	0.410784
**GO:0003677**	DNA binding	19	172	1185	11484	0.412521
**GO:0044822**	poly(A) RNA binding	11	172	667	11484	0.415552
**GO:0019902**	phosphatase binding	1	172	36	11484	0.419636
**GO:0033138**	positive regulation of peptidyl-serine phosphorylation	1	172	36	11484	0.419636
**GO:0005768**	endosome	2	172	97	11484	0.428052
**GO:0006869**	lipid transport	1	172	37	11484	0.428355
**GO:0006887**	exocytosis	1	172	37	11484	0.428355
**GO:0006987**	activation of signaling protein activity involved in unfolded protein response	1	172	37	11484	0.428355
**GO:0008630**	intrinsic apoptotic signaling pathway in response to DNA damage	1	172	37	11484	0.428355
**GO:0042383**	sarcolemma	1	172	37	11484	0.428355
**GO:0045216**	cell-cell junction organization	1	172	37	11484	0.428355
**GO:0044255**	cellular lipid metabolic process	2	172	98	11484	0.433188
**GO:0045121**	membrane raft	2	172	99	11484	0.4383
**GO:0005525**	GTP binding	4	172	227	11484	0.443115
**GO:0043565**	sequence-specific DNA binding	6	172	356	11484	0.443378

**GO:0030336**	negative regulation of cell migration	1	172	39	11484	0.445406
**GO:0034146**	toll-like receptor 5 signaling pathway	1	172	39	11484	0.445406
**GO:0034166**	toll-like receptor 10 signaling pathway	1	172	39	11484	0.445406
**GO:0045596**	negative regulation of cell differentiation	1	172	39	11484	0.445406
**GO:0050680**	negative regulation of epithelial cell proliferation	1	172	39	11484	0.445406
**GO:0090502**	RNA phosphodiester bond hydrolysis, endonucleolytic	1	172	39	11484	0.445406
**GO:0000278**	mitotic cell cycle	4	172	228	11484	0.446446
**GO:0031625**	ubiquitin protein ligase binding	2	172	101	11484	0.448448
**GO:0051056**	regulation of small GTPase mediated signal transduction	2	172	101	11484	0.448448
**GO:0004674**	protein serine/threonine kinase activity	4	172	230	11484	0.453088
**GO:0004888**	transmembrane signaling receptor activity	2	172	102	11484	0.453484
**GO:0009055**	electron carrier activity	1	172	40	11484	0.453741
**GO:0032355**	response to estradiol	1	172	40	11484	0.453741
**GO:0005739**	mitochondrion	13	172	818	11484	0.453959
**GO:0030097**	hemopoiesis	1	172	41	11484	0.461951
**GO:0030176**	integral component of endoplasmic reticulum membrane	1	172	41	11484	0.461951
**GO:0006367**	transcription initiation from RNA polymerase II promoter	2	172	104	11484	0.463476
**GO:0044267**	cellular protein metabolic process	5	172	299	11484	0.465615
**GO:0000785**	chromatin	1	172	42	11484	0.470038
**GO:0006139**	nucleobase-containing compound metabolic process	1	172	42	11484	0.470038
**GO:0006767**	water-soluble vitamin metabolic process	1	172	42	11484	0.470038
**GO:0006977**	DNA damage response, signal transduction by p53 class mediator resulting in cell cycle arrest	1	172	42	11484	0.470038
**GO:0007229**	integrin-mediated signaling pathway	1	172	42	11484	0.470038
**GO:0042826**	histone deacetylase binding	1	172	42	11484	0.470038
**GO:0008203**	cholesterol metabolic process	1	172	43	11484	0.478005

**GO:0030512**	negative regulation of transforming growth factor beta receptor signaling pathway	1	172	43	11484	0.478005
**GO:0030658**	transport vesicle membrane	1	172	43	11484	0.478005
**GO:0030968**	endoplasmic reticulum unfolded protein response	1	172	43	11484	0.478005
**GO:0000288**	nuclear-transcribed mRNA catabolic process, deadenylation-dependent decay	1	172	44	11484	0.485852
**GO:0006338**	chromatin remodeling	1	172	44	11484	0.485852
**GO:0009058**	biosynthetic process	1	172	44	11484	0.485852
**GO:0035666**	TRIF-dependent toll-like receptor signaling pathway	1	172	44	11484	0.485852
**GO:0038123**	toll-like receptor TLR1:TLR2 signaling pathway	1	172	44	11484	0.485852
**GO:0038124**	toll-like receptor TLR6:TLR2 signaling pathway	1	172	44	11484	0.485852
**GO:0042393**	histone binding	1	172	44	11484	0.485852
**GO:0045766**	positive regulation of angiogenesis	1	172	44	11484	0.485852
**GO:0050660**	flavin adenine dinucleotide binding	1	172	44	11484	0.485852
**GO:0005975**	carbohydrate metabolic process	4	172	240	11484	0.485894
**GO:0046982**	protein heterodimerization activity	4	172	241	11484	0.489133
**GO:0019904**	protein domain specific binding	2	172	110	11484	0.492802
**GO:0002756**	MyD88-independent toll-like receptor signaling pathway	1	172	45	11484	0.493582
**GO:0005581**	collagen trimer	1	172	45	11484	0.493582
**GO:0032481**	positive regulation of type I interferon production	1	172	45	11484	0.493582
**GO:0034138**	toll-like receptor 3 signaling pathway	1	172	45	11484	0.493582
**GO:0034162**	toll-like receptor 9 signaling pathway	1	172	45	11484	0.493582
**GO:0044325**	ion channel binding	1	172	45	11484	0.493582
**GO:0007160**	cell-matrix adhesion	1	172	46	11484	0.501197
**GO:0016363**	nuclear matrix	1	172	46	11484	0.501197
**GO:0034134**	toll-like receptor 2 signaling pathway	1	172	46	11484	0.501197
**GO:0042391**	regulation of membrane potential	1	172	46	11484	0.501197
**GO:0042470**	melanosome	1	172	46	11484	0.501197
**GO:0000977**	RNA polymerase II regulatory region sequence-specific DNA binding	1	172	47	11484	0.508698

**GO:0005543**	phospholipid binding	1	172	47	11484	0.508698
**GO:0006898**	receptor-mediated endocytosis	1	172	47	11484	0.508698
**GO:0055086**	nucleobase-containing small molecule metabolic process	1	172	47	11484	0.508698
**GO:0000287**	magnesium ion binding	2	172	114	11484	0.511786
**GO:0016491**	oxidoreductase activity	3	172	182	11484	0.51542
**GO:0006766**	vitamin metabolic process	1	172	48	11484	0.516086
**GO:0035264**	multicellular organism growth	1	172	48	11484	0.516086
**GO:0043065**	positive regulation of apoptotic process	3	172	184	11484	0.522785
**GO:0001889**	liver development	1	172	49	11484	0.523365
**GO:0016773**	phosphotransferase activity, alcohol group as acceptor	1	172	49	11484	0.523365
**GO:0042981**	regulation of apoptotic process	2	172	117	11484	0.525718
**GO:0000165**	MAPK cascade	1	172	50	11484	0.530534
**GO:0002755**	MyD88-dependent toll-like receptor signaling pathway	1	172	50	11484	0.530534
**GO:0006665**	sphingolipid metabolic process	1	172	50	11484	0.530534
**GO:0016042**	lipid catabolic process	1	172	50	11484	0.530534
**GO:0030165**	PDZ domain binding	1	172	50	11484	0.530534
**GO:0042593**	glucose homeostasis	1	172	50	11484	0.530534
**GO:0000139**	Golgi membrane	5	172	324	11484	0.536628
**GO:0005770**	late endosome	1	172	51	11484	0.537596
**GO:0006874**	cellular calcium ion homeostasis	1	172	51	11484	0.537596
**GO:0009791**	post-embryonic development	1	172	51	11484	0.537596
**GO:0006810**	transport	5	172	326	11484	0.542132
**GO:0001503**	ossification	1	172	53	11484	0.551405
**GO:0003690**	double-stranded DNA binding	1	172	53	11484	0.551405
**GO:0034220**	ion transmembrane transport	2	172	123	11484	0.552772
**GO:0008234**	cysteine-type peptidase activity	1	172	54	11484	0.558155
**GO:0019083**	viral transcription	1	172	54	11484	0.558155
**GO:0014069**	postsynaptic density	1	172	55	11484	0.564804
**GO:0007264**	small GTPase mediated signal transduction	3	172	197	11484	0.56913
**GO:0006812**	cation transport	1	172	56	11484	0.571353
**GO:0016568**	chromatin modification	1	172	56	11484	0.571353
**GO:0034142**	toll-like receptor 4 signaling pathway	1	172	56	11484	0.571353
**GO:0005198**	structural molecule activity	2	172	128	11484	0.574478
**GO:0005777**	peroxisome	1	172	58	11484	0.58416
**GO:0005179**	hormone activity	1	172	59	11484	0.590419
**GO:0006415**	translational termination	1	172	59	11484	0.590419
**GO:0009653**	anatomical structure morphogenesis	1	172	59	11484	0.590419
**GO:0032321**	positive regulation of Rho GTPase activity	1	172	59	11484	0.590419

**GO:0005764**	lysosome	2	172	132	11484	0.591289
**GO:0000981**	sequence-specific DNA binding RNA polymerase II transcription factor activity	1	172	60	11484	0.596586
**GO:0006913**	nucleocytoplasmic transport	1	172	60	11484	0.596586
**GO:0006112**	energy reserve metabolic process	1	172	61	11484	0.602659
**GO:0009887**	organ morphogenesis	1	172	61	11484	0.602659
**GO:0016337**	single organismal cell-cell adhesion	1	172	61	11484	0.602659
**GO:0071456**	cellular response to hypoxia	1	172	61	11484	0.602659
**GO:0016192**	vesicle-mediated transport	2	172	135	11484	0.603571
**GO:0016324**	apical plasma membrane	2	172	136	11484	0.607603
**GO:0007218**	neuropeptide signaling pathway	1	172	62	11484	0.608642
**GO:0043235**	receptor complex	1	172	62	11484	0.608642
**GO:0006816**	calcium ion transport	1	172	63	11484	0.614536
**GO:0009952**	anterior/posterior pattern specification	1	172	63	11484	0.614536
**GO:0007166**	cell surface receptor signaling pathway	2	172	138	11484	0.615574
**GO:0015031**	protein transport	4	172	283	11484	0.616233
**GO:0045892**	negative regulation of transcription, DNA-templated	4	172	284	11484	0.619015
**GO:0005759**	mitochondrial matrix	2	172	139	11484	0.619514
**GO:0060337**	type I interferon signaling pathway	1	172	64	11484	0.620341
**GO:0003824**	catalytic activity	5	172	356	11484	0.620746
**GO:0005730**	nucleolus	6	172	427	11484	0.621059
**GO:0007067**	mitotic nuclear division	2	172	140	11484	0.623422
**GO:0006614**	SRP-dependent cotranslational protein targeting to membrane	1	172	66	11484	0.631691
**GO:0005938**	cell cortex	1	172	67	11484	0.63724
**GO:0005654**	nucleoplasm	9	172	647	11484	0.638646
**GO:0006351**	transcription, DNA-templated	16	172	1137	11484	0.642233
**GO:0008233**	peptidase activity	1	172	69	11484	0.648088
**GO:0007268**	synaptic transmission	3	172	222	11484	0.650112
**GO:0003682**	chromatin binding	3	172	223	11484	0.653117
**GO:0000790**	nuclear chromatin	1	172	70	11484	0.65339
**GO:0000184**	nuclear-transcribed mRNA catabolic process, nonsense-mediated decay	1	172	72	11484	0.663758
**GO:0002479**	antigen processing and presentation of exogenous peptide antigen via MHC class I, TAP-dependent	1	172	72	11484	0.663758

**GO:0031225**	anchored component of membrane	1	172	72	11484	0.663758
**GO:0006412**	translation	2	172	152	11484	0.667929
**GO:0008286**	insulin receptor signaling pathway	1	172	74	11484	0.673818
**GO:0015630**	microtubule cytoskeleton	1	172	75	11484	0.678735
**GO:0042590**	antigen processing and presentation of exogenous peptide antigen via MHC class I	1	172	75	11484	0.678735
**GO:0006464**	cellular protein modification process	1	172	76	11484	0.683578
**GO:0005815**	microtubule organizing center	1	172	77	11484	0.688349
**GO:0019058**	viral life cycle	1	172	77	11484	0.688349
**GO:0031410**	cytoplasmic vesicle	1	172	77	11484	0.688349
**GO:0006952**	defense response	1	172	78	11484	0.693048
**GO:0004872**	receptor activity	2	172	161	11484	0.698467
**GO:0005819**	spindle	1	172	80	11484	0.702236
**GO:0005096**	GTPase activator activity	1	172	81	11484	0.706727
**GO:0005929**	cilium	1	172	82	11484	0.711151
**GO:0043231**	intracellular membrane-bounded organelle	3	172	245	11484	0.714597
**GO:0005769**	early endosome	1	172	83	11484	0.715508
**GO:0008150**	biological_process	5	172	400	11484	0.720473
**GO:0006413**	translational initiation	1	172	85	11484	0.724028
**GO:0030054**	cell junction	3	172	250	11484	0.727343
**GO:0005874**	microtubule	2	172	173	11484	0.735547
**GO:0000086**	G2/M transition of mitotic cell cycle	1	172	88	11484	0.736334
**GO:0002474**	antigen processing and presentation of peptide antigen via MHC class I	1	172	89	11484	0.740313
**GO:0016032**	viral process	4	172	336	11484	0.745779
**GO:0019886**	antigen processing and presentation of exogenous peptide antigen via MHC class II	1	172	91	11484	0.748094
**GO:0007411**	axon guidance	2	172	179	11484	0.752597
**GO:0004713**	protein tyrosine kinase activity	3	172	263	11484	0.75841
**GO:0005911**	cell-cell junction	1	172	94	11484	0.759333
**GO:0030154**	cell differentiation	3	172	269	11484	0.771762
**GO:0005788**	endoplasmic reticulum lumen	1	172	98	11484	0.773546
**GO:0006457**	protein folding	1	172	98	11484	0.773546
**GO:0008017**	microtubule binding	1	172	98	11484	0.773546
**GO:0050776**	regulation of immune response	1	172	98	11484	0.773546
**GO:0006281**	DNA repair	2	172	187	11484	0.773859
**GO:0044237**	cellular metabolic process	1	172	101	11484	0.783655

**GO:0003735**	structural constituent of ribosome	1	172	104	11484	0.793316
**GO:0009897**	external side of plasma membrane	1	172	105	11484	0.79644
**GO:0005813**	centrosome	2	172	200	11484	0.80503
**GO:0005840**	ribosome	1	172	108	11484	0.805532
**GO:0019882**	antigen processing and presentation	1	172	109	11484	0.808473
**GO:0030246**	carbohydrate binding	1	172	111	11484	0.814221
**GO:0044212**	transcription regulatory region DNA binding	1	172	111	11484	0.814221
**GO:0006644**	phospholipid metabolic process	1	172	112	11484	0.817031
**GO:0031965**	nuclear membrane	1	172	112	11484	0.817031
**GO:0003674**	molecular_function	5	172	458	11484	0.821484
**GO:0045211**	postsynaptic membrane	1	172	115	11484	0.825209
**GO:0016311**	dephosphorylation	1	172	125	11484	0.849932
**GO:0003713**	transcription coactivator activity	1	172	130	11484	0.860957
**GO:0005575**	cellular_component	3	172	319	11484	0.861058
**GO:0043547**	positive regulation of GTPase activity	1	172	133	11484	0.867181
**GO:0016874**	ligase activity	1	172	138	11484	0.876945
**GO:0007283**	spermatogenesis	2	172	241	11484	0.879828
**GO:0030425**	dendrite	1	172	143	11484	0.885996
**GO:0004871**	signal transducer activity	1	172	147	11484	0.892757
**GO:0055085**	transmembrane transport	4	172	435	11484	0.895539
**GO:0005789**	endoplasmic reticulum membrane	4	172	436	11484	0.896542
**GO:0016020**	membrane	23	172	1928	11484	0.90813
**GO:0043025**	neuronal cell body	1	172	159	11484	0.91074
**GO:0000166**	nucleotide binding	2	172	276	11484	0.921775
**GO:0005765**	lysosomal membrane	1	172	170	11484	0.924575
**GO:0006886**	intracellular protein transport	1	172	189	11484	0.943636
**GO:0016567**	protein ubiquitination	1	172	198	11484	0.950909
**GO:0005886**	plasma membrane	26	172	2269	11484	0.95293
**GO:0003723**	RNA binding	2	172	334	11484	0.962582
**GO:0005488**	binding	1	172	218	11484	0.963901
**GO:0007186**	G-protein coupled receptor signaling pathway	4	172	663	11484	0.99103
**GO:0008152**	metabolic process	3	172	696	11484	0.998518
**GO:0003676**	nucleic acid binding	3	172	712	11484	0.998803
**GO:0016021**	integral component of membrane	21	172	2786	11484	0.999978

GO analyses of the dysregulated miRNA targets

GO_ID	GO_Term	S.gene. number	TS.gene. number	B.gene. number	TB.gene. number	pvalue
**GO:0009612**	response to mechanical stimulus	6	103	30	16571	2.62E-08
**GO:0005615**	extracellular space	16	103	618	16571	1.27E-06
**GO:0000302**	response to reactive oxygen species	3	103	7	16571	8.02E-06
**GO:0005576**	extracellular region	25	103	1718	16571	3.86E-05
**GO:0009725**	response to hormone stimulus	4	103	37	16571	7.94E-05
**GO:0009968**	negative regulation of signal transduction	4	103	41	16571	0.000119
**GO:0031093**	platelet alpha granule lumen	4	103	41	16571	0.000119
**GO:0001666**	response to hypoxia	6	103	127	16571	0.00014
**GO:0005319**	lipid transporter activity	3	103	18	16571	0.000178
**GO:0018149**	peptide cross-linking	3	103	20	16571	0.000246
**GO:0032570**	response to progesterone stimulus	3	103	21	16571	0.000286
**GO:0005604**	basement membrane	4	103	53	16571	0.000326
**GO:0002020**	protease binding	3	103	22	16571	0.00033
**GO:0050728**	negative regulation of inflammatory response	3	103	22	16571	0.00033
**GO:0051895**	negative regulation of focal adhesion formation	2	103	5	16571	0.000378
**GO:0008201**	heparin binding	5	103	107	16571	0.000542
**GO:0008034**	lipoprotein binding	2	103	6	16571	0.000565
**GO:0006942**	regulation of striated muscle contraction	2	103	7	16571	0.000787
**GO:0048741**	skeletal muscle fiber development	2	103	7	16571	0.000787
**GO:0006915**	apoptosis	9	103	398	16571	0.000834
**GO:0008285**	negative regulation of cell proliferation	7	103	253	16571	0.001016
**GO:0043537**	negative regulation of blood vessel endothelial cell migration	2	103	8	16571	0.001046
**GO:0003810**	protein-glutamine gamma-glutamyltransferase activity	2	103	9	16571	0.001339
**GO:0009617**	response to bacterium	2	103	9	16571	0.001339
**GO:0016209**	antioxidant activity	2	103	9	16571	0.001339
**GO:0045944**	positive regulation of transcription from RNA polymerase II promoter	7	103	275	16571	0.001641
**GO:0006898**	receptor-mediated endocytosis	3	103	39	16571	0.001812
**GO:0008307**	structural constituent of muscle	3	103	42	16571	0.002245
**GO:0051592**	response to calcium ion	3	103	44	16571	0.002567
**GO:0001503**	ossification	3	103	45	16571	0.002738
**GO:0048168**	regulation of neuronal synaptic plasticity	2	103	13	16571	0.002854

**GO:0005925**	focal adhesion	4	103	96	16571	0.003019
**GO:0001937**	negative regulation of endothelial cell proliferation	2	103	14	16571	0.003316
**GO:0006979**	response to oxidative stress	4	103	101	16571	0.003624
**GO:0034605**	cellular response to heat	2	103	15	16571	0.003811
**GO:0001501**	skeletal system development	4	103	107	16571	0.004452
**GO:0045727**	positive regulation of translation	2	103	17	16571	0.004896
**GO:0030036**	actin cytoskeleton organization	4	103	110	16571	0.004911
**GO:0001558**	regulation of cell growth	3	103	57	16571	0.005351
**GO:0048008**	platelet-derived growth factor receptor signaling pathway	2	103	18	16571	0.005486
**GO:0007568**	aging	3	103	59	16571	0.005892
**GO:0042246**	tissue regeneration	2	103	19	16571	0.006107
**GO:0048754**	branching morphogenesis of a tube	2	103	19	16571	0.006107
**GO:0001910**	regulation of leukocyte mediated cytotoxicity	1	103	1	16571	0.006216
**GO:0002315**	marginal zone B cell differentiation	1	103	1	16571	0.006216
**GO:0002457**	T cell antigen processing and presentation	1	103	1	16571	0.006216
**GO:0002581**	negative regulation of antigen processing and presentation of peptide or polysaccharide antigen via MHC class II	1	103	1	16571	0.006216
**GO:0002605**	negative regulation of dendritic cell antigen processing and presentation	1	103	1	16571	0.006216
**GO:0002689**	negative regulation of leukocyte chemotaxis	1	103	1	16571	0.006216
**GO:0002693**	positive regulation of cellular extravasation	1	103	1	16571	0.006216
**GO:0004810**	tRNA adenylyltransferase activity	1	103	1	16571	0.006216
**GO:0004941**	beta2-adrenergic receptor activity	1	103	1	16571	0.006216
**GO:0005151**	interleukin-1, Type II receptor binding	1	103	1	16571	0.006216
**GO:0005865**	striated muscle thin filament	1	103	1	16571	0.006216
**GO:0006358**	regulation of transcription from RNA polymerase II promoter, global	1	103	1	16571	0.006216
**GO:0008179**	adenylate cyclase binding	1	103	1	16571	0.006216
**GO:0010751**	negative regulation of nitric oxide mediated signal transduction	1	103	1	16571	0.006216

**GO:0010754**	negative regulation of cGMP-mediated signaling	1	103	1	16571	0.006216
**GO:0010757**	negative regulation of plasminogen activation	1	103	1	16571	0.006216
**GO:0010759**	positive regulation of macrophage chemotaxis	1	103	1	16571	0.006216
**GO:0019730**	antimicrobial humoral response	1	103	1	16571	0.006216
**GO:0032805**	positive regulation of low-density lipoprotein receptor catabolic process	1	103	1	16571	0.006216
**GO:0032996**	Bcl3-Bcl10 complex	1	103	1	16571	0.006216
**GO:0033371**	T cell secretory granule organization	1	103	1	16571	0.006216
**GO:0033373**	maintenance of protease location in mast cell secretory granule	1	103	1	16571	0.006216
**GO:0033382**	maintenance of granzyme B location in T cell secretory granule	1	103	1	16571	0.006216
**GO:0034755**	iron ion transmembrane transport	1	103	1	16571	0.006216
**GO:0042780**	tRNA 3'-end processing	1	103	1	16571	0.006216
**GO:0043460**	response to long exposure to lithium ion	1	103	1	16571	0.006216
**GO:0043503**	skeletal muscle fiber adaptation	1	103	1	16571	0.006216
**GO:0043652**	engulfment of apoptotic cell	1	103	1	16571	0.006216
**GO:0045064**	T-helper 2 cell differentiation	1	103	1	16571	0.006216
**GO:0045986**	negative regulation of smooth muscle contraction	1	103	1	16571	0.006216
**GO:0046911**	metal chelating activity	1	103	1	16571	0.006216
**GO:0048603**	fibroblast growth factor 2 binding	1	103	1	16571	0.006216
**GO:0050614**	delta24-sterol reductase activity	1	103	1	16571	0.006216
**GO:0051545**	negative regulation of elastin biosynthetic process	1	103	1	16571	0.006216
**GO:0051651**	maintenance of location in cell	1	103	1	16571	0.006216
**GO:0055013**	cardiac muscle cell development	1	103	1	16571	0.006216
**GO:0070051**	fibrinogen binding	1	103	1	16571	0.006216
**GO:0070287**	ferritin receptor activity	1	103	1	16571	0.006216
**GO:0070325**	lipoprotein receptor binding	1	103	1	16571	0.006216
**GO:0003779**	actin binding	6	103	267	16571	0.006434
**GO:0050873**	brown fat cell differentiation	2	103	20	16571	0.006758
**GO:0042127**	regulation of cell proliferation	3	103	62	16571	0.006763
**GO:0007596**	blood coagulation	3	103	64	16571	0.007384

**GO:0055010**	ventricular cardiac muscle tissue morphogenesis	2	103	21	16571	0.007439
**GO:0004860**	protein kinase inhibitor activity	2	103	22	16571	0.00815
**GO:0030017**	sarcomere	2	103	23	16571	0.00889
**GO:0031012**	extracellular matrix	2	103	23	16571	0.00889
**GO:0032496**	response to lipopolysaccharide	3	103	70	16571	0.009446
**GO:0007566**	embryo implantation	2	103	25	16571	0.010457
**GO:0031100**	organ regeneration	2	103	25	16571	0.010457
**GO:0005509**	calcium ion binding	12	103	907	16571	0.010516
**GO:0014070**	response to organic cyclic substance	3	103	73	16571	0.01059
**GO:0004857**	enzyme inhibitor activity	2	103	26	16571	0.011283
**GO:0010468**	regulation of gene expression	2	103	26	16571	0.011283
**GO:0048661**	positive regulation of smooth muscle cell proliferation	2	103	27	16571	0.012137
**GO:0000289**	nuclear-transcribed mRNA poly(A) tail shortening	1	103	2	16571	0.012393
**GO:0001300**	chronological cell aging	1	103	2	16571	0.012393
**GO:0002025**	vasodilation by norepinephrine-epinephrine involved in regulation of systemic arterial blood pressure	1	103	2	16571	0.012393
**GO:0002268**	follicular dendritic cell differentiation	1	103	2	16571	0.012393
**GO:0002291**	T cell activation via T cell receptor contact with antigen bound to MHC molecule on antigen presenting cell	1	103	2	16571	0.012393
**GO:0002455**	humoral immune response mediated by circulating immunoglobulin	1	103	2	16571	0.012393
**GO:0005146**	leukemia inhibitory factor receptor binding	1	103	2	16571	0.012393
**GO:0005501**	retinoid binding	1	103	2	16571	0.012393
**GO:0005602**	complement component C1 complex	1	103	2	16571	0.012393
**GO:0007023**	post-chaperonin tubulin folding pathway	1	103	2	16571	0.012393
**GO:0008147**	structural constituent of bone	1	103	2	16571	0.012393
**GO:0010670**	positive regulation of oxygen and reactive oxygen species metabolic process	1	103	2	16571	0.012393
**GO:0010763**	positive regulation of fibroblast migration	1	103	2	16571	0.012393
**GO:0015825**	L-serine transport	1	103	2	16571	0.012393

**GO:0018153**	isopeptide cross-linking via N6-(L-isoglutamyl)-L-lysine	1	103	2	16571	0.012393
**GO:0021587**	cerebellum morphogenesis	1	103	2	16571	0.012393
**GO:0031444**	slow-twitch skeletal muscle fiber contraction	1	103	2	16571	0.012393
**GO:0031649**	heat generation	1	103	2	16571	0.012393
**GO:0032036**	myosin heavy chain binding	1	103	2	16571	0.012393
**GO:0032288**	myelin assembly	1	103	2	16571	0.012393
**GO:0032914**	positive regulation of transforming growth factor-beta1 production	1	103	2	16571	0.012393
**GO:0033257**	Bcl3/NF-kappaB2 complex	1	103	2	16571	0.012393
**GO:0033364**	mast cell secretory granule organization	1	103	2	16571	0.012393
**GO:0034447**	very-low-density lipoprotein particle clearance	1	103	2	16571	0.012393
**GO:0043374**	CD8-positive, alpha-beta T cell differentiation	1	103	2	16571	0.012393
**GO:0043394**	proteoglycan binding	1	103	2	16571	0.012393
**GO:0043615**	astrocyte cell migration	1	103	2	16571	0.012393
**GO:0045082**	positive regulation of interleukin-10 biosynthetic process	1	103	2	16571	0.012393
**GO:0045088**	regulation of innate immune response	1	103	2	16571	0.012393
**GO:0045415**	negative regulation of interleukin-8 biosynthetic process	1	103	2	16571	0.012393
**GO:0045835**	negative regulation of meiosis	1	103	2	16571	0.012393
**GO:0048633**	positive regulation of skeletal muscle tissue growth	1	103	2	16571	0.012393
**GO:0050779**	RNA destabilization	1	103	2	16571	0.012393
**GO:0008016**	regulation of heart contraction	2	103	28	16571	0.013018
**GO:0043410**	positive regulation of MAPKKK cascade	2	103	28	16571	0.013018
**GO:0006958**	complement activation, classical pathway	2	103	29	16571	0.013926
**GO:0045768**	positive regulation of anti-apoptosis	2	103	30	16571	0.014861
**GO:0060021**	palate development	2	103	30	16571	0.014861
**GO:0002024**	diet induced thermogenesis	1	103	3	16571	0.018532
**GO:0002032**	desensitization of G-protein coupled receptor protein signaling pathway by arrestin	1	103	3	16571	0.018532
**GO:0002467**	germinal center formation	1	103	3	16571	0.018532
**GO:0002544**	chronic inflammatory response	1	103	3	16571	0.018532

**GO:0004687**	myosin light chain kinase activity	1	103	3	16571	0.018532
**GO:0004784**	superoxide dismutase activity	1	103	3	16571	0.018532
**GO:0006082**	organic acid metabolic process	1	103	3	16571	0.018532
**GO:0006658**	phosphatidylserine metabolic process	1	103	3	16571	0.018532
**GO:0008064**	regulation of actin polymerization or depolymerization	1	103	3	16571	0.018532
**GO:0009991**	response to extracellular stimulus	1	103	3	16571	0.018532
**GO:0015194**	L-serine transmembrane transporter activity	1	103	3	16571	0.018532
**GO:0015917**	aminophospholipid transport	1	103	3	16571	0.018532
**GO:0019829**	cation-transporting ATPase activity	1	103	3	16571	0.018532
**GO:0030239**	myofibril assembly	1	103	3	16571	0.018532
**GO:0031665**	negative regulation of lipopolysaccharide-mediated signaling pathway	1	103	3	16571	0.018532
**GO:0033627**	cell adhesion mediated by integrin	1	103	3	16571	0.018532
**GO:0040037**	negative regulation of fibroblast growth factor receptor signaling pathway	1	103	3	16571	0.018532
**GO:0042345**	regulation of NF-kappaB import into nucleus	1	103	3	16571	0.018532
**GO:0042473**	outer ear morphogenesis	1	103	3	16571	0.018532
**GO:0043268**	positive regulation of potassium ion transport	1	103	3	16571	0.018532
**GO:0043277**	apoptotic cell clearance	1	103	3	16571	0.018532
**GO:0043405**	regulation of MAP kinase activity	1	103	3	16571	0.018532
**GO:0045541**	negative regulation of cholesterol biosynthetic process	1	103	3	16571	0.018532
**GO:0046813**	virion attachment, binding of host cell surface receptor	1	103	3	16571	0.018532
**GO:0048592**	eye morphogenesis	1	103	3	16571	0.018532
**GO:0050921**	positive regulation of chemotaxis	1	103	3	16571	0.018532
**GO:0051380**	norepinephrine binding	1	103	3	16571	0.018532
**GO:0051856**	adhesion to symbiont	1	103	3	16571	0.018532
**GO:0070052**	collagen V binding	1	103	3	16571	0.018532
**GO:0070326**	very-low-density lipoprotein receptor binding	1	103	3	16571	0.018532
**GO:0007517**	muscle organ development	3	103	92	16571	0.019652

**GO:0006916**	anti-apoptosis	4	103	174	16571	0.023258
**GO:0009986**	cell surface	4	103	176	16571	0.02413
**GO:0042542**	response to hydrogen peroxide	2	103	39	16571	0.024418
**GO:0000293**	ferric-chelate reductase activity	1	103	4	16571	0.024634
**GO:0004740**	pyruvate dehydrogenase (acetyl-transferring) kinase activity	1	103	4	16571	0.024634
**GO:0005862**	muscle thin filament tropomyosin	1	103	4	16571	0.024634
**GO:0006739**	NADP metabolic process	1	103	4	16571	0.024634
**GO:0006883**	cellular sodium ion homeostasis	1	103	4	16571	0.024634
**GO:0007044**	cell-substrate junction assembly	1	103	4	16571	0.024634
**GO:0007216**	metabotropic glutamate receptor signaling pathway	1	103	4	16571	0.024634
**GO:0008330**	protein tyrosine/threonine phosphatase activity	1	103	4	16571	0.024634
**GO:0010544**	negative regulation of platelet activation	1	103	4	16571	0.024634
**GO:0010748**	negative regulation of plasma membrane long-chain fatty acid transport	1	103	4	16571	0.024634
**GO:0010765**	positive regulation of sodium ion transport	1	103	4	16571	0.024634
**GO:0012501**	programmed cell death	1	103	4	16571	0.024634
**GO:0014912**	negative regulation of smooth muscle cell migration	1	103	4	16571	0.024634
**GO:0016504**	peptidase activator activity	1	103	4	16571	0.024634
**GO:0019934**	cGMP-mediated signaling	1	103	4	16571	0.024634
**GO:0030055**	cell-substrate junction	1	103	4	16571	0.024634
**GO:0030516**	regulation of axon extension	1	103	4	16571	0.024634
**GO:0030828**	positive regulation of cGMP biosynthetic process	1	103	4	16571	0.024634
**GO:0031232**	extrinsic to external side of plasma membrane	1	103	4	16571	0.024634
**GO:0031638**	zymogen activation	1	103	4	16571	0.024634
**GO:0031639**	plasminogen activation	1	103	4	16571	0.024634
**GO:0031995**	insulin-like growth factor II binding	1	103	4	16571	0.024634
**GO:0032488**	Cdc42 protein signal transduction	1	103	4	16571	0.024634
**GO:0032695**	negative regulation of interleukin-12 production	1	103	4	16571	0.024634
**GO:0034363**	intermediate-density lipoprotein particle	1	103	4	16571	0.024634
**GO:0035240**	dopamine binding	1	103	4	16571	0.024634

**GO:0042536**	negative regulation of tumor necrosis factor biosynthetic process	1	103	4	16571	0.024634
**GO:0042629**	mast cell granule	1	103	4	16571	0.024634
**GO:0042832**	defense response to protozoan	1	103	4	16571	0.024634
**GO:0043032**	positive regulation of macrophage activation	1	103	4	16571	0.024634
**GO:0043218**	compact myelin	1	103	4	16571	0.024634
**GO:0045217**	cell-cell junction maintenance	1	103	4	16571	0.024634
**GO:0045638**	negative regulation of myeloid cell differentiation	1	103	4	16571	0.024634
**GO:0045778**	positive regulation of ossification	1	103	4	16571	0.024634
**GO:0045823**	positive regulation of heart contraction	1	103	4	16571	0.024634
**GO:0045879**	negative regulation of smoothened signaling pathway	1	103	4	16571	0.024634
**GO:0045880**	positive regulation of smoothened signaling pathway	1	103	4	16571	0.024634
**GO:0048861**	leukemia inhibitory factor signaling pathway	1	103	4	16571	0.024634
**GO:0050847**	progesterone receptor signaling pathway	1	103	4	16571	0.024634
**GO:0051019**	mitogen-activated protein kinase binding	1	103	4	16571	0.024634
**GO:0051045**	negative regulation of membrane protein ectodomain proteolysis	1	103	4	16571	0.024634
**GO:0051101**	regulation of DNA binding	1	103	4	16571	0.024634
**GO:0051393**	alpha-actinin binding	1	103	4	16571	0.024634
**GO:0051549**	positive regulation of keratinocyte migration	1	103	4	16571	0.024634
**GO:0051593**	response to folic acid	1	103	4	16571	0.024634
**GO:0055003**	cardiac myofibril assembly	1	103	4	16571	0.024634
**GO:0060047**	heart contraction	1	103	4	16571	0.024634
**GO:0060135**	maternal process involved in female pregnancy	1	103	4	16571	0.024634
**GO:0070053**	thrombospondin receptor activity	1	103	4	16571	0.024634
**GO:0004222**	metalloendopeptidase activity	3	103	101	16571	0.025061
**GO:0009408**	response to heat	2	103	40	16571	0.0256
**GO:0009749**	response to glucose stimulus	2	103	40	16571	0.0256
**GO:0007050**	cell cycle arrest	3	103	104	16571	0.027025
**GO:0030425**	dendrite	3	103	104	16571	0.027025
**GO:0005901**	caveola	2	103	42	16571	0.028034
**GO:0001527**	microfibril	1	103	5	16571	0.030698

**GO:0002040**	sprouting angiogenesis	1	103	5	16571	0.030698
**GO:0002102**	podosome	1	103	5	16571	0.030698
**GO:0004499**	flavin-containing monooxygenase activity	1	103	5	16571	0.030698
**GO:0005853**	eukaryotic translation elongation factor 1 complex	1	103	5	16571	0.030698
**GO:0007171**	activation of transmembrane receptor protein tyrosine kinase activity	1	103	5	16571	0.030698
**GO:0009404**	toxin metabolic process	1	103	5	16571	0.030698
**GO:0009649**	entrainment of circadian clock	1	103	5	16571	0.030698
**GO:0018106**	peptidyl-histidine phosphorylation	1	103	5	16571	0.030698
**GO:0022614**	membrane to membrane docking	1	103	5	16571	0.030698
**GO:0030194**	positive regulation of blood coagulation	1	103	5	16571	0.030698
**GO:0030240**	muscle thin filament assembly	1	103	5	16571	0.030698
**GO:0030277**	maintenance of gastrointestinal epithelium	1	103	5	16571	0.030698
**GO:0031434**	mitogen-activated protein kinase kinase binding	1	103	5	16571	0.030698
**GO:0034185**	apolipoprotein binding	1	103	5	16571	0.030698
**GO:0034380**	high-density lipoprotein particle assembly	1	103	5	16571	0.030698
**GO:0034382**	chylomicron remnant clearance	1	103	5	16571	0.030698
**GO:0042158**	lipoprotein biosynthetic process	1	103	5	16571	0.030698
**GO:0042159**	lipoprotein catabolic process	1	103	5	16571	0.030698
**GO:0042989**	sequestering of actin monomers	1	103	5	16571	0.030698
**GO:0048156**	tau protein binding	1	103	5	16571	0.030698
**GO:0048644**	muscle organ morphogenesis	1	103	5	16571	0.030698
**GO:0048771**	tissue remodeling	1	103	5	16571	0.030698
**GO:0050710**	negative regulation of cytokine secretion	1	103	5	16571	0.030698
**GO:0051347**	positive regulation of transferase activity	1	103	5	16571	0.030698
**GO:0051927**	negative regulation of calcium ion transport via voltage-gated calcium channel activity	1	103	5	16571	0.030698
**GO:0060228**	phosphatidylcholine-sterol O-acyltransferase activator activity	1	103	5	16571	0.030698
**GO:0060347**	heart trabecula formation	1	103	5	16571	0.030698
**GO:0001822**	kidney development	2	103	46	16571	0.033165
**GO:0043627**	response to estrogen stimulus	2	103	46	16571	0.033165
**GO:0016477**	cell migration	2	103	48	16571	0.035858

**GO:0002021**	response to dietary excess	1	103	6	16571	0.036725
**GO:0005138**	interleukin-6 receptor binding	1	103	6	16571	0.036725
**GO:0010225**	response to UV-C	1	103	6	16571	0.036725
**GO:0010875**	positive regulation of cholesterol efflux	1	103	6	16571	0.036725
**GO:0019885**	antigen processing and presentation of endogenous peptide antigen via MHC class I	1	103	6	16571	0.036725
**GO:0030500**	regulation of bone mineralization	1	103	6	16571	0.036725
**GO:0030502**	negative regulation of bone mineralization	1	103	6	16571	0.036725
**GO:0031994**	insulin-like growth factor I binding	1	103	6	16571	0.036725
**GO:0034372**	very-low-density lipoprotein particle remodeling	1	103	6	16571	0.036725
**GO:0034384**	high-density lipoprotein particle clearance	1	103	6	16571	0.036725
**GO:0043353**	enucleate erythrocyte differentiation	1	103	6	16571	0.036725
**GO:0045651**	positive regulation of macrophage differentiation	1	103	6	16571	0.036725
**GO:0051918**	negative regulation of fibrinolysis	1	103	6	16571	0.036725
**GO:0070207**	protein homotrimerization	1	103	6	16571	0.036725
**GO:0045941**	positive regulation of transcription	3	103	118	16571	0.037244
**GO:0003924**	GTPase activity	4	103	208	16571	0.040799
**GO:0001968**	fibronectin binding	1	103	7	16571	0.042714
**GO:0004935**	adrenoceptor activity	1	103	7	16571	0.042714
**GO:0005577**	fibrinogen complex	1	103	7	16571	0.042714
**GO:0005579**	membrane attack complex	1	103	7	16571	0.042714
**GO:0006801**	superoxide metabolic process	1	103	7	16571	0.042714
**GO:0010873**	positive regulation of cholesterol esterification	1	103	7	16571	0.042714
**GO:0017091**	AU-rich element binding	1	103	7	16571	0.042714
**GO:0030279**	negative regulation of ossification	1	103	7	16571	0.042714
**GO:0030898**	actin-dependent ATPase activity	1	103	7	16571	0.042714
**GO:0032026**	response to magnesium ion	1	103	7	16571	0.042714
**GO:0040015**	negative regulation of multicellular organism growth	1	103	7	16571	0.042714
**GO:0043488**	regulation of mRNA stability	1	103	7	16571	0.042714
**GO:0043536**	positive regulation of blood vessel endothelial cell migration	1	103	7	16571	0.042714

**GO:0045084**	positive regulation of interleukin-12 biosynthetic process	1	103	7	16571	0.042714
**GO:0051605**	protein maturation by peptide bond cleavage	1	103	7	16571	0.042714
**GO:0032355**	response to estradiol stimulus	2	103	53	16571	0.042943
**GO:0005578**	proteinaceous extracellular matrix	4	103	215	16571	0.045137
**GO:0001935**	endothelial cell proliferation	1	103	8	16571	0.048667
**GO:0003785**	actin monomer binding	1	103	8	16571	0.048667
**GO:0005861**	troponin complex	1	103	8	16571	0.048667
**GO:0005916**	fascia adherens	1	103	8	16571	0.048667
**GO:0032094**	response to food	1	103	8	16571	0.048667
**GO:0035313**	wound healing, spreading of epidermal cells	1	103	8	16571	0.048667
**GO:0042730**	fibrinolysis	1	103	8	16571	0.048667
**GO:0042987**	amyloid precursor protein catabolic process	1	103	8	16571	0.048667
**GO:0044424**	intracellular part	1	103	8	16571	0.048667
**GO:0051018**	protein kinase A binding	1	103	8	16571	0.048667
**GO:0051457**	maintenance of protein location in nucleus	1	103	8	16571	0.048667
**GO:0051602**	response to electrical stimulus	1	103	8	16571	0.048667
**GO:0030324**	lung development	2	103	57	16571	0.048953
**GO:0003700**	transcription factor activity	10	103	890	16571	0.049884
**GO:0005178**	integrin binding	2	103	59	16571	0.052066
**GO:0001542**	ovulation from ovarian follicle	1	103	9	16571	0.054583
**GO:0001786**	phosphatidylserine binding	1	103	9	16571	0.054583
**GO:0002027**	regulation of heart rate	1	103	9	16571	0.054583
**GO:0004115**	3',5'-cyclic-AMP phosphodiesterase activity	1	103	9	16571	0.054583
**GO:0016601**	Rac protein signal transduction	1	103	9	16571	0.054583
**GO:0030049**	muscle filament sliding	1	103	9	16571	0.054583
**GO:0033138**	positive regulation of peptidyl-serine phosphorylation	1	103	9	16571	0.054583
**GO:0042088**	T-helper 1 type immune response	1	103	9	16571	0.054583
**GO:0043236**	laminin binding	1	103	9	16571	0.054583
**GO:0045749**	negative regulation of S phase of mitotic cell cycle	1	103	9	16571	0.054583
**GO:0051000**	positive regulation of nitric-oxide synthase activity	1	103	9	16571	0.054583
**GO:0051044**	positive regulation of membrane protein ectodomain proteolysis	1	103	9	16571	0.054583
**GO:0051930**	regulation of sensory perception of pain	1	103	9	16571	0.054583

**GO:0055088**	lipid homeostasis	1	103	9	16571	0.054583
**GO:0060325**	face morphogenesis	1	103	9	16571	0.054583
**GO:0006629**	lipid metabolic process	4	103	231	16571	0.055986
**GO:0005516**	calmodulin binding	3	103	140	16571	0.056717
**GO:0005794**	Golgi apparatus	9	103	789	16571	0.056817
**GO:0005201**	extracellular matrix structural constituent	2	103	64	16571	0.060145
**GO:0001772**	immunological synapse	1	103	10	16571	0.060463
**GO:0006259**	DNA metabolic process	1	103	10	16571	0.060463
**GO:0006402**	mRNA catabolic process	1	103	10	16571	0.060463
**GO:0006707**	cholesterol catabolic process	1	103	10	16571	0.060463
**GO:0007512**	adult heart development	1	103	10	16571	0.060463
**GO:0008191**	metalloendopeptidase inhibitor activity	1	103	10	16571	0.060463
**GO:0010039**	response to iron ion	1	103	10	16571	0.060463
**GO:0017144**	drug metabolic process	1	103	10	16571	0.060463
**GO:0030169**	low-density lipoprotein binding	1	103	10	16571	0.060463
**GO:0032729**	positive regulation of interferon-gamma production	1	103	10	16571	0.060463
**GO:0033700**	phospholipid efflux	1	103	10	16571	0.060463
**GO:0042327**	positive regulation of phosphorylation	1	103	10	16571	0.060463
**GO:0042605**	peptide antigen binding	1	103	10	16571	0.060463
**GO:0046888**	negative regulation of hormone secretion	1	103	10	16571	0.060463
**GO:0048701**	embryonic cranial skeleton morphogenesis	1	103	10	16571	0.060463
**GO:0050431**	transforming growth factor beta binding	1	103	10	16571	0.060463
**GO:0051482**	elevation of cytosolic calcium ion concentration during G-protein signaling, coupled to IP3 second messenger (phospholipase C activating)	1	103	10	16571	0.060463
**GO:0005507**	copper ion binding	2	103	66	16571	0.063491
**GO:0005125**	cytokine activity	3	103	147	16571	0.063751
**GO:0006509**	membrane protein ectodomain proteolysis	1	103	11	16571	0.066306
**GO:0008301**	DNA bending activity	1	103	11	16571	0.066306
**GO:0009888**	tissue development	1	103	11	16571	0.066306
**GO:0019216**	regulation of lipid metabolic process	1	103	11	16571	0.066306
**GO:0019827**	stem cell maintenance	1	103	11	16571	0.066306
**GO:0042517**	positive regulation of tyrosine phosphorylation of Stat3 protein	1	103	11	16571	0.066306
**GO:0042627**	chylomicron	1	103	11	16571	0.066306

**GO:0043034**	costamere	1	103	11	16571	0.066306
**GO:0045214**	sarcomere organization	1	103	11	16571	0.066306
**GO:0045671**	negative regulation of osteoclast differentiation	1	103	11	16571	0.066306
**GO:0045909**	positive regulation of vasodilation	1	103	11	16571	0.066306
**GO:0046688**	response to copper ion	1	103	11	16571	0.066306
**GO:0050750**	low-density lipoprotein receptor binding	1	103	11	16571	0.066306
**GO:0000287**	magnesium ion binding	6	103	461	16571	0.067592
**GO:0006937**	regulation of muscle contraction	1	103	12	16571	0.072113
**GO:0008235**	metalloexopeptidase activity	1	103	12	16571	0.072113
**GO:0017127**	cholesterol transporter activity	1	103	12	16571	0.072113
**GO:0042474**	middle ear morphogenesis	1	103	12	16571	0.072113
**GO:0043235**	receptor complex	1	103	12	16571	0.072113
**GO:0043407**	negative regulation of MAP kinase activity	1	103	12	16571	0.072113
**GO:0048844**	artery morphogenesis	1	103	12	16571	0.072113
**GO:0050660**	FAD binding	2	103	71	16571	0.072119
**GO:0005525**	GTP binding	5	103	358	16571	0.072496
**GO:0000146**	microfilament motor activity	1	103	13	16571	0.077885
**GO:0000155**	two-component sensor activity	1	103	13	16571	0.077885
**GO:0001540**	beta-amyloid binding	1	103	13	16571	0.077885
**GO:0005154**	epidermal growth factor receptor binding	1	103	13	16571	0.077885
**GO:0005344**	oxygen transporter activity	1	103	13	16571	0.077885
**GO:0006200**	ATP catabolic process	1	103	13	16571	0.077885
**GO:0007271**	synaptic transmission, cholinergic	1	103	13	16571	0.077885
**GO:0015671**	oxygen transport	1	103	13	16571	0.077885
**GO:0017017**	MAP kinase tyrosine/serine/threonine phosphatase activity	1	103	13	16571	0.077885
**GO:0017022**	myosin binding	1	103	13	16571	0.077885
**GO:0021904**	dorsal/ventral neural tube patterning	1	103	13	16571	0.077885
**GO:0030539**	male genitalia development	1	103	13	16571	0.077885
**GO:0034362**	low-density lipoprotein particle	1	103	13	16571	0.077885
**GO:0034375**	high-density lipoprotein particle remodeling	1	103	13	16571	0.077885
**GO:0042311**	vasodilation	1	103	13	16571	0.077885
**GO:0042771**	DNA damage response, signal transduction by p53 class mediator resulting in induction of apoptosis	1	103	13	16571	0.077885
**GO:0045453**	bone resorption	1	103	13	16571	0.077885

**GO:0046697**	decidualization	1	103	13	16571	0.077885
**GO:0048662**	negative regulation of smooth muscle cell proliferation	1	103	13	16571	0.077885
**GO:0050840**	extracellular matrix binding	1	103	13	16571	0.077885
**GO:0003727**	single-stranded RNA binding	1	103	14	16571	0.083621
**GO:0005523**	tropomyosin binding	1	103	14	16571	0.083621
**GO:0006479**	protein amino acid methylation	1	103	14	16571	0.083621
**GO:0007026**	negative regulation of microtubule depolymerization	1	103	14	16571	0.083621
**GO:0007263**	nitric oxide mediated signal transduction	1	103	14	16571	0.083621
**GO:0007292**	female gamete generation	1	103	14	16571	0.083621
**GO:0008333**	endosome to lysosome transport	1	103	14	16571	0.083621
**GO:0030511**	positive regulation of transforming growth factor beta receptor signaling pathway	1	103	14	16571	0.083621
**GO:0043526**	neuroprotection	1	103	14	16571	0.083621
**GO:0048545**	response to steroid hormone stimulus	1	103	14	16571	0.083621
**GO:0001932**	regulation of protein amino acid phosphorylation	1	103	15	16571	0.089322
**GO:0002053**	positive regulation of mesenchymal cell proliferation	1	103	15	16571	0.089322
**GO:0004012**	phospholipid-translocating ATPase activity	1	103	15	16571	0.089322
**GO:0005859**	muscle myosin complex	1	103	15	16571	0.089322
**GO:0006957**	complement activation, alternative pathway	1	103	15	16571	0.089322
**GO:0010165**	response to X-ray	1	103	15	16571	0.089322
**GO:0032982**	myosin filament	1	103	15	16571	0.089322
**GO:0042809**	vitamin D receptor binding	1	103	15	16571	0.089322
**GO:0043154**	negative regulation of caspase activity	1	103	15	16571	0.089322
**GO:0043200**	response to amino acid stimulus	1	103	15	16571	0.089322
**GO:0045785**	positive regulation of cell adhesion	1	103	15	16571	0.089322
**GO:0045860**	positive regulation of protein kinase activity	1	103	15	16571	0.089322
**GO:0046658**	anchored to plasma membrane	1	103	15	16571	0.089322
**GO:0042995**	cell projection	2	103	81	16571	0.090409
**GO:0001502**	cartilage condensation	1	103	16	16571	0.094988
**GO:0006665**	sphingolipid metabolic process	1	103	16	16571	0.094988
**GO:0010811**	positive regulation of cell-substrate adhesion	1	103	16	16571	0.094988
**GO:0030136**	clathrin-coated vesicle	1	103	16	16571	0.094988
**GO:0034364**	high-density lipoprotein particle	1	103	16	16571	0.094988

**GO:0043691**	reverse cholesterol transport	1	103	16	16571	0.094988
**GO:0046627**	negative regulation of insulin receptor signaling pathway	1	103	16	16571	0.094988
**GO:0048169**	regulation of long-term neuronal synaptic plasticity	1	103	16	16571	0.094988
**GO:0048536**	spleen development	1	103	16	16571	0.094988
**GO:0048589**	developmental growth	1	103	16	16571	0.094988
**GO:0050900**	leukocyte migration	1	103	16	16571	0.094988
**GO:0006936**	muscle contraction	2	103	84	16571	0.096138
**GO:0043065**	positive regulation of apoptosis	2	103	84	16571	0.096138
**GO:0030308**	negative regulation of cell growth	2	103	85	16571	0.09807
**GO:0030414**	peptidase inhibitor activity	2	103	85	16571	0.09807
**GO:0045121**	membrane raft	2	103	85	16571	0.09807
**GO:0000060**	protein import into nucleus, translocation	1	103	17	16571	0.100618
**GO:0005520**	insulin-like growth factor binding	1	103	17	16571	0.100618
**GO:0006805**	xenobiotic metabolic process	1	103	17	16571	0.100618
**GO:0043499**	eukaryotic cell surface binding	1	103	17	16571	0.100618
**GO:0045597**	positive regulation of cell differentiation	1	103	17	16571	0.100618
**GO:0045765**	regulation of angiogenesis	1	103	17	16571	0.100618
**GO:0007264**	small GTPase mediated signal transduction	3	103	180	16571	0.101846
**GO:0004867**	serine-type endopeptidase inhibitor activity	2	103	87	16571	0.101968
**GO:0007163**	establishment or maintenance of cell polarity	1	103	18	16571	0.106214
**GO:0015914**	phospholipid transport	1	103	18	16571	0.106214
**GO:0019835**	cytolysis	1	103	18	16571	0.106214
**GO:0030217**	T cell differentiation	1	103	18	16571	0.106214
**GO:0030501**	positive regulation of bone mineralization	1	103	18	16571	0.106214
**GO:0030514**	negative regulation of BMP signaling pathway	1	103	18	16571	0.106214
**GO:0031214**	biomineral formation	1	103	18	16571	0.106214
**GO:0031227**	intrinsic to endoplasmic reticulum membrane	1	103	18	16571	0.106214
**GO:0007601**	visual perception	3	103	184	16571	0.10697
**GO:0016563**	transcription activator activity	3	103	184	16571	0.10697
**GO:0004888**	transmembrane receptor activity	2	103	91	16571	0.109889
**GO:0007243**	protein kinase cascade	2	103	91	16571	0.109889
**GO:0000188**	inactivation of MAPK activity	1	103	19	16571	0.111776
**GO:0001974**	blood vessel remodeling	1	103	19	16571	0.111776

**GO:0007259**	JAK-STAT cascade	1	103	19	16571	0.111776
**GO:0042157**	lipoprotein metabolic process	1	103	19	16571	0.111776
**GO:0043531**	ADP binding	1	103	19	16571	0.111776
**GO:0006414**	translational elongation	2	103	93	16571	0.113909
**GO:0003746**	translation elongation factor activity	1	103	20	16571	0.117303
**GO:0006641**	triglyceride metabolic process	1	103	20	16571	0.117303
**GO:0009409**	response to cold	1	103	20	16571	0.117303
**GO:0015662**	ATPase activity, coupled to transmembrane movement of ions, phosphorylative mechanism	1	103	20	16571	0.117303
**GO:0030216**	keratinocyte differentiation	1	103	20	16571	0.117303
**GO:0034361**	very-low-density lipoprotein particle	1	103	20	16571	0.117303
**GO:0046907**	intracellular transport	1	103	20	16571	0.117303
**GO:0050729**	positive regulation of inflammatory response	1	103	20	16571	0.117303
**GO:0005164**	tumor necrosis factor receptor binding	1	103	21	16571	0.122796
**GO:0006626**	protein targeting to mitochondrion	1	103	21	16571	0.122796
**GO:0007157**	heterophilic cell adhesion	1	103	21	16571	0.122796
**GO:0010332**	response to gamma radiation	1	103	21	16571	0.122796
**GO:0045429**	positive regulation of nitric oxide biosynthetic process	1	103	21	16571	0.122796
**GO:0001658**	branching involved in ureteric bud morphogenesis	1	103	22	16571	0.128256
**GO:0001725**	stress fiber	1	103	22	16571	0.128256
**GO:0005080**	protein kinase C binding	1	103	22	16571	0.128256
**GO:0030155**	regulation of cell adhesion	1	103	22	16571	0.128256
**GO:0042391**	regulation of membrane potential	1	103	22	16571	0.128256
**GO:0043588**	skin development	1	103	22	16571	0.128256
**GO:0050680**	negative regulation of epithelial cell proliferation	1	103	22	16571	0.128256
**GO:0000049**	tRNA binding	1	103	23	16571	0.133681
**GO:0007159**	leukocyte adhesion	1	103	23	16571	0.133681
**GO:0007422**	peripheral nervous system development	1	103	23	16571	0.133681
**GO:0009954**	proximal/distal pattern formation	1	103	23	16571	0.133681
**GO:0015459**	potassium channel regulator activity	1	103	23	16571	0.133681
**GO:0016525**	negative regulation of angiogenesis	1	103	23	16571	0.133681
**GO:0042733**	embryonic digit morphogenesis	1	103	23	16571	0.133681

**GO:0045444**	fat cell differentiation	1	103	23	16571	0.133681
**GO:0051087**	chaperone binding	1	103	23	16571	0.133681
**GO:0001525**	angiogenesis	2	103	103	16571	0.134544
**GO:0005884**	actin filament	1	103	24	16571	0.139073
**GO:0006695**	cholesterol biosynthetic process	1	103	24	16571	0.139073
**GO:0006800**	oxygen and reactive oxygen species metabolic process	1	103	24	16571	0.139073
**GO:0031526**	brush border membrane	1	103	24	16571	0.139073
**GO:0045766**	positive regulation of angiogenesis	1	103	24	16571	0.139073
**GO:0048666**	neuron development	1	103	24	16571	0.139073
**GO:0048705**	skeletal system morphogenesis	1	103	24	16571	0.139073
**GO:0048706**	embryonic skeletal system development	1	103	24	16571	0.139073
**GO:0043066**	negative regulation of apoptosis	2	103	107	16571	0.143021
**GO:0005887**	integral to plasma membrane	9	103	962	16571	0.143712
**GO:0007249**	I-kappaB kinase/NF-kappaB cascade	1	103	25	16571	0.144432
**GO:0048146**	positive regulation of fibroblast proliferation	1	103	25	16571	0.144432
**GO:0006879**	cellular iron ion homeostasis	1	103	26	16571	0.149758
**GO:0010243**	response to organic nitrogen	1	103	26	16571	0.149758
**GO:0043433**	negative regulation of transcription factor activity	1	103	26	16571	0.149758
**GO:0007507**	heart development	2	103	112	16571	0.153771
**GO:0005758**	mitochondrial intermembrane space	1	103	27	16571	0.155052
**GO:0007173**	epidermal growth factor receptor signaling pathway	1	103	27	16571	0.155052
**GO:0007611**	learning or memory	1	103	27	16571	0.155052
**GO:0001541**	ovarian follicle development	1	103	28	16571	0.160312
**GO:0004177**	aminopeptidase activity	1	103	28	16571	0.160312
**GO:0043204**	perikaryon	1	103	28	16571	0.160312
**GO:0030154**	cell differentiation	5	103	464	16571	0.162489
**GO:0007254**	JNK cascade	1	103	29	16571	0.16554
**GO:0016455**	RNA polymerase II transcription mediator activity	1	103	29	16571	0.16554
**GO:0043010**	camera-type eye development	1	103	29	16571	0.16554
**GO:0045165**	cell fate commitment	1	103	29	16571	0.16554
**GO:0005874**	microtubule	3	103	227	16571	0.167703
**GO:0007275**	multicellular organismal development	8	103	866	16571	0.170394
**GO:0006605**	protein targeting	1	103	30	16571	0.170736
**GO:0008277**	regulation of G-protein coupled receptor protein signaling pathway	1	103	30	16571	0.170736

**GO:0030178**	negative regulation of Wnt receptor signaling pathway	1	103	30	16571	0.170736
**GO:0043231**	intracellular membrane-bounded organelle	1	103	30	16571	0.170736
**GO:0050731**	positive regulation of peptidyl-tyrosine phosphorylation	1	103	30	16571	0.170736
**GO:0009897**	external side of plasma membrane	2	103	120	16571	0.17128
**GO:0005783**	endoplasmic reticulum	8	103	868	16571	0.171891
**GO:0043025**	cell soma	2	103	121	16571	0.173492
**GO:0007189**	activation of adenylate cyclase activity by G-protein signaling pathway	1	103	31	16571	0.1759
**GO:0005518**	collagen binding	1	103	32	16571	0.181032
**GO:0006953**	acute-phase response	1	103	32	16571	0.181032
**GO:0030307**	positive regulation of cell growth	1	103	32	16571	0.181032
**GO:0048306**	calcium-dependent protein binding	1	103	32	16571	0.181032
**GO:0050661**	NADP or NADPH binding	1	103	32	16571	0.181032
**GO:0051789**	response to protein stimulus	1	103	32	16571	0.181032
**GO:0032868**	response to insulin stimulus	1	103	33	16571	0.186132
**GO:0007267**	cell-cell signaling	3	103	239	16571	0.186177
**GO:0008284**	positive regulation of cell proliferation	3	103	241	16571	0.189307
**GO:0005626**	insoluble fraction	1	103	34	16571	0.191201
**GO:0007205**	activation of protein kinase C activity by G-protein coupled receptor protein signaling pathway	1	103	34	16571	0.191201
**GO:0030326**	embryonic limb morphogenesis	1	103	34	16571	0.191201
**GO:0050679**	positive regulation of epithelial cell proliferation	1	103	34	16571	0.191201
**GO:0008134**	transcription factor binding	2	103	130	16571	0.193598
**GO:0008022**	protein C-terminus binding	2	103	132	16571	0.198108
**GO:0000079**	regulation of cyclin-dependent protein kinase activity	1	103	36	16571	0.201245
**GO:0022625**	cytosolic large ribosomal subunit	1	103	36	16571	0.201245
**GO:0008415**	acyltransferase activity	2	103	134	16571	0.202631
**GO:0006955**	immune response	4	103	375	16571	0.204923
**GO:0005770**	late endosome	1	103	37	16571	0.20622
**GO:0008092**	cytoskeletal protein binding	1	103	37	16571	0.20622
**GO:0016459**	myosin complex	1	103	37	16571	0.20622
**GO:0008283**	cell proliferation	3	103	252	16571	0.206761
**GO:0000082**	G1/S transition of mitotic cell cycle	1	103	38	16571	0.211165

**GO:0003729**	mRNA binding	1	103	38	16571	0.211165
**GO:0006469**	negative regulation of protein kinase activity	1	103	38	16571	0.211165
**GO:0008144**	drug binding	1	103	38	16571	0.211165
**GO:0042632**	cholesterol homeostasis	1	103	39	16571	0.21608
**GO:0051591**	response to cAMP	1	103	39	16571	0.21608
**GO:0005905**	coated pit	1	103	40	16571	0.220964
**GO:0019825**	oxygen binding	1	103	40	16571	0.220964
**GO:0004497**	monooxygenase activity	1	103	41	16571	0.225818
**GO:0051092**	positive regulation of NF-kappaB transcription factor activity	1	103	41	16571	0.225818
**GO:0007242**	intracellular signaling cascade	3	103	265	16571	0.22784
**GO:0006006**	glucose metabolic process	1	103	42	16571	0.230642
**GO:0005044**	scavenger receptor activity	1	103	43	16571	0.235436
**GO:0008654**	phospholipid biosynthetic process	1	103	43	16571	0.235436
**GO:0034097**	response to cytokine stimulus	1	103	43	16571	0.235436
**GO:0042383**	sarcolemma	1	103	43	16571	0.235436
**GO:0042593**	glucose homeostasis	1	103	43	16571	0.235436
**GO:0005543**	phospholipid binding	1	103	44	16571	0.240201
**GO:0016049**	cell growth	1	103	44	16571	0.240201
**GO:0007268**	synaptic transmission	2	103	151	16571	0.241467
**GO:0005802**	trans-Golgi network	1	103	45	16571	0.244936
**GO:0030674**	protein binding, bridging	1	103	45	16571	0.244936
**GO:0008083**	growth factor activity	2	103	154	16571	0.248368
**GO:0016820**	hydrolase activity, acting on acid anhydrides, catalyzing transmembrane movement of substances	1	103	46	16571	0.249642
**GO:0030182**	neuron differentiation	1	103	46	16571	0.249642
**GO:0006754**	ATP biosynthetic process	1	103	47	16571	0.254319
**GO:0009791**	post-embryonic development	1	103	47	16571	0.254319
**GO:0005789**	endoplasmic reticulum membrane	2	103	157	16571	0.255277
**GO:0006917**	induction of apoptosis	2	103	157	16571	0.255277
**GO:0005096**	GTPase activator activity	2	103	158	16571	0.257581
**GO:0043434**	response to peptide hormone stimulus	1	103	49	16571	0.263586
**GO:0003774**	motor activity	1	103	50	16571	0.268177
**GO:0004672**	protein kinase activity	1	103	50	16571	0.268177
**GO:0005097**	Rab GTPase activator activity	1	103	50	16571	0.268177
**GO:0006986**	response to unfolded protein	1	103	50	16571	0.268177
**GO:0008217**	regulation of blood pressure	1	103	50	16571	0.268177
**GO:0032313**	regulation of Rab GTPase activity	1	103	50	16571	0.268177

**GO:0000187**	activation of MAPK activity	1	103	51	16571	0.27274
**GO:0040008**	regulation of growth	1	103	51	16571	0.27274
**GO:0003674**	molecular_function	5	103	566	16571	0.276042
**GO:0008104**	protein localization	1	103	52	16571	0.277274
**GO:0006508**	proteolysis	4	103	432	16571	0.281468
**GO:0006396**	RNA processing	1	103	53	16571	0.281781
**GO:0007010**	cytoskeleton organization	1	103	53	16571	0.281781
**GO:0016044**	membrane organization	1	103	53	16571	0.281781
**GO:0005625**	soluble fraction	3	103	298	16571	0.282902
**GO:0005515**	protein binding	37	103	5444	16571	0.285001
**GO:0006357**	regulation of transcription from RNA polymerase II promoter	2	103	170	16571	0.285233
**GO:0006874**	cellular calcium ion homeostasis	1	103	55	16571	0.29071
**GO:0005506**	iron ion binding	2	103	174	16571	0.294437
**GO:0005737**	cytoplasm	30	103	4368	16571	0.294762
**GO:0007229**	integrin-mediated signaling pathway	1	103	57	16571	0.29953
**GO:0008360**	regulation of cell shape	1	103	57	16571	0.29953
**GO:0030198**	extracellular matrix organization	1	103	57	16571	0.29953
**GO:0045471**	response to ethanol	1	103	58	16571	0.303898
**GO:0051260**	protein homooligomerization	1	103	58	16571	0.303898
**GO:0005198**	structural molecule activity	2	103	184	16571	0.317371
**GO:0016568**	chromatin modification	2	103	184	16571	0.317371
**GO:0005249**	voltage-gated potassium channel activity	1	103	62	16571	0.321106
**GO:0007584**	response to nutrient	1	103	62	16571	0.321106
**GO:0042742**	defense response to bacterium	1	103	62	16571	0.321106
**GO:0007265**	Ras protein signal transduction	1	103	63	16571	0.325342
**GO:0046982**	protein heterodimerization activity	2	103	188	16571	0.326501
**GO:0043565**	sequence-specific DNA binding	4	103	465	16571	0.32774
**GO:0003690**	double-stranded DNA binding	1	103	64	16571	0.329551
**GO:0007411**	axon guidance	1	103	64	16571	0.329551
**GO:0008233**	peptidase activity	4	103	467	16571	0.330567
**GO:0007565**	female pregnancy	1	103	65	16571	0.333735
**GO:0032403**	protein complex binding	1	103	65	16571	0.333735
**GO:0042493**	response to drug	2	103	192	16571	0.335601
**GO:0006869**	lipid transport	1	103	67	16571	0.342024
**GO:0009790**	embryonic development	1	103	67	16571	0.342024
**GO:0014069**	postsynaptic density	1	103	67	16571	0.342024
**GO:0005768**	endosome	2	103	195	16571	0.342403
**GO:0005923**	tight junction	1	103	68	16571	0.34613
**GO:0042803**	protein homodimerization activity	3	103	339	16571	0.352654
**GO:0004725**	protein tyrosine phosphatase activity	1	103	70	16571	0.354267

**GO:0016779**	nucleotidyltransferase activity	1	103	70	16571	0.354267
**GO:0045892**	negative regulation of transcription, DNA-dependent	1	103	71	16571	0.358298
**GO:0006810**	transport	3	103	344	16571	0.361153
**GO:0005200**	structural constituent of cytoskeleton	1	103	74	16571	0.370241
**GO:0005788**	endoplasmic reticulum lumen	1	103	74	16571	0.370241
**GO:0006954**	inflammatory response	2	103	216	16571	0.389337
**GO:0009952**	anterior/posterior pattern formation	1	103	80	16571	0.393471
**GO:0030054**	cell junction	3	103	367	16571	0.400006
**GO:0016055**	Wnt receptor signaling pathway	1	103	82	16571	0.401024
**GO:0008076**	voltage-gated potassium channel complex	1	103	84	16571	0.408484
**GO:0042470**	melanosome	1	103	85	16571	0.412179
**GO:0046983**	protein dimerization activity	1	103	85	16571	0.412179
**GO:0005792**	microsome	2	103	228	16571	0.415509
**GO:0007417**	central nervous system development	1	103	88	16571	0.423129
**GO:0019899**	enzyme binding	1	103	88	16571	0.423129
**GO:0022900**	electron transport chain	1	103	88	16571	0.423129
**GO:0005730**	nucleolus	5	103	687	16571	0.425023
**GO:0006974**	response to DNA damage stimulus	2	103	233	16571	0.426252
**GO:0030528**	transcription regulator activity	1	103	90	16571	0.430316
**GO:0009653**	anatomical structure morphogenesis	1	103	91	16571	0.433877
**GO:0007165**	signal transduction	9	103	1324	16571	0.440808
**GO:0019904**	protein domain specific binding	1	103	94	16571	0.444426
**GO:0008289**	lipid binding	1	103	96	16571	0.45135
**GO:0045087**	innate immune response	1	103	96	16571	0.45135
**GO:0006897**	endocytosis	1	103	97	16571	0.45478
**GO:0006928**	cellular component movement	1	103	99	16571	0.461577
**GO:0006461**	protein complex assembly	1	103	100	16571	0.464944
**GO:0016491**	oxidoreductase activity	3	103	412	16571	0.473916
**GO:0043123**	positive regulation of I-kappaB kinase/NF-kappaB cascade	1	103	103	16571	0.47492
**GO:0008150**	biological_process	4	103	570	16571	0.474984
**GO:0005886**	plasma membrane	16	103	2481	16571	0.478459
**GO:0006468**	protein amino acid phosphorylation	3	103	421	16571	0.488231
**GO:0042981**	regulation of apoptosis	1	103	110	16571	0.497486
**GO:0043234**	protein complex	1	103	110	16571	0.497486
**GO:0009615**	response to virus	1	103	111	16571	0.50063
**GO:0030424**	axon	1	103	112	16571	0.503755
**GO:0009887**	organ morphogenesis	1	103	114	16571	0.509947

**GO:0020037**	heme binding	1	103	114	16571	0.509947
**GO:0006366**	transcription from RNA polymerase II promoter	1	103	115	16571	0.513014
**GO:0008219**	cell death	1	103	115	16571	0.513014
**GO:0016324**	apical plasma membrane	1	103	120	16571	0.528066
**GO:0007049**	cell cycle	3	103	447	16571	0.528508
**GO:0006470**	protein amino acid dephosphorylation	1	103	124	16571	0.539776
**GO:0016887**	ATPase activity	1	103	124	16571	0.539776
**GO:0016481**	negative regulation of transcription	1	103	125	16571	0.542658
**GO:0005624**	membrane fraction	3	103	463	16571	0.552423
**GO:0042802**	identical protein binding	2	103	298	16571	0.555545
**GO:0016564**	transcription repressor activity	1	103	131	16571	0.55958
**GO:0045211**	postsynaptic membrane	1	103	135	16571	0.570515
**GO:0005856**	cytoskeleton	4	103	652	16571	0.581443
**GO:0005694**	chromosome	1	103	141	16571	0.586415
**GO:0004252**	serine-type endopeptidase activity	1	103	144	16571	0.594145
**GO:0005759**	mitochondrial matrix	1	103	145	16571	0.59669
**GO:0006813**	potassium ion transport	1	103	147	16571	0.601732
**GO:0003735**	structural constituent of ribosome	1	103	151	16571	0.61163
**GO:0006281**	DNA repair	1	103	154	16571	0.618893
**GO:0055114**	oxidation reduction	3	103	512	16571	0.621083
**GO:0000166**	nucleotide binding	11	103	1888	16571	0.636594
**GO:0005764**	lysosome	1	103	170	16571	0.655418
**GO:0005634**	nucleus	28	103	4744	16571	0.663343
**GO:0019941**	modification-dependent protein catabolic process	2	103	369	16571	0.671958
**GO:0005840**	ribosome	1	103	178	16571	0.672358
**GO:0005524**	ATP binding	8	103	1434	16571	0.676946
**GO:0005575**	cellular_component	3	103	572	16571	0.69507
**GO:0031225**	anchored to membrane	1	103	191	16571	0.698143
**GO:0045449**	regulation of transcription	5	103	937	16571	0.699035
**GO:0005975**	carbohydrate metabolic process	1	103	199	16571	0.713001
**GO:0016740**	transferase activity	6	103	1137	16571	0.717808
**GO:0000122**	negative regulation of transcription from RNA polymerase II promoter	1	103	208	16571	0.728852
**GO:0031410**	cytoplasmic vesicle	1	103	213	16571	0.73728
**GO:0006355**	regulation of transcription, DNA-dependent	5	103	990	16571	0.744226
**GO:0007186**	G-protein coupled receptor protein signaling pathway	2	103	425	16571	0.745517
**GO:0005743**	mitochondrial inner membrane	1	103	226	16571	0.757999

**GO:0019898**	extrinsic to membrane	2	103	443	16571	0.765997
**GO:0045202**	synapse	1	103	240	16571	0.778504
**GO:0050896**	response to stimulus	1	103	243	16571	0.782668
**GO:0004872**	receptor activity	4	103	863	16571	0.790877
**GO:0005829**	cytosol	6	103	1245	16571	0.795191
**GO:0008152**	metabolic process	2	103	475	16571	0.798921
**GO:0007283**	spermatogenesis	1	103	257	16571	0.801102
**GO:0046872**	metal ion binding	12	103	2349	16571	0.808264
**GO:0005488**	binding	2	103	491	16571	0.81381
**GO:0004871**	signal transducer activity	1	103	268	16571	0.814491
**GO:0044419**	interspecies interaction between organisms	1	103	271	16571	0.817985
**GO:0005622**	intracellular	9	103	1841	16571	0.821531
**GO:0007155**	cell adhesion	2	103	506	16571	0.826877
**GO:0008270**	zinc ion binding	11	103	2227	16571	0.83343
**GO:0005215**	transporter activity	1	103	286	16571	0.834502
**GO:0004930**	G-protein coupled receptor activity	1	103	295	16571	0.84369
**GO:0004674**	protein serine/threonine kinase activity	1	103	306	16571	0.854236
**GO:0007399**	nervous system development	1	103	328	16571	0.87326
**GO:0016787**	hydrolase activity	3	103	926	16571	0.93231
**GO:0005739**	mitochondrion	3	103	1007	16571	0.953948
**GO:0006811**	ion transport	1	103	496	16571	0.956712
**GO:0003723**	RNA binding	1	103	550	16571	0.969425
**GO:0016020**	membrane	15	103	3609	16571	0.975692
**GO:0003677**	DNA binding	2	103	1184	16571	0.995768
**GO:0016021**	integral to membrane	12	103	3564	16571	0.996852

GO analyses of the dysregulated lncRNA targets

**GO_ID**	GO_Term	S.gene. number	TS.gene. number	B.gene. number	TB.gene. number	pvalue
**GO:0030049**	muscle filament sliding	5	41	19	11484	5.05E-09
**GO:0006936**	muscle contraction	6	41	52	11484	2.55E-08
**GO:0031093**	platelet alpha granule lumen	4	41	21	11484	8.01E-07
**GO:0071682**	endocytic vesicle lumen	3	41	8	11484	2.34E-06
**GO:0005829**	cytosol	17	41	1536	11484	8.09E-06
**GO:0002576**	platelet degranulation	4	41	40	11484	1.16E-05
**GO:0031720**	haptoglobin binding	2	41	2	11484	1.24E-05
**GO:0031838**	haptoglobin-hemoglobin complex	2	41	2	11484	1.24E-05
**GO:0005615**	extracellular space	11	41	661	11484	1.37E-05
**GO:0070062**	extracellular vesicular exosome	16	41	1439	11484	1.61E-05
**GO:0030198**	extracellular matrix organization	6	41	172	11484	3.01E-05
**GO:0000791**	euchromatin	2	41	3	11484	3.72E-05

**GO:0005862**	muscle thin filament tropomyosin	2	41	3	11484	3.72E-05
**GO:0008307**	structural constituent of muscle	3	41	22	11484	6.21E-05
**GO:0005576**	extracellular region	12	41	935	11484	6.80E-05
**GO:0031012**	extracellular matrix	5	41	122	11484	6.88E-05
**GO:0030017**	sarcomere	3	41	23	11484	7.12E-05
**GO:0006942**	regulation of striated muscle contraction	2	41	4	11484	7.43E-05
**GO:0042542**	response to hydrogen peroxide	3	41	25	11484	9.20E-05
**GO:0022617**	extracellular matrix disassembly	4	41	72	11484	0.000121
**GO:0001725**	stress fiber	3	41	29	11484	0.000145
**GO:0005833**	hemoglobin complex	2	41	7	11484	0.000258
**GO:0097067**	cellular response to thyroid hormone stimulus	2	41	7	11484	0.000258
**GO:0072562**	blood microparticle	4	41	88	11484	0.000263
**GO:0005518**	collagen binding	3	41	38	11484	0.000327
**GO:0005344**	oxygen transporter activity	2	41	8	11484	0.000344
**GO:0015671**	oxygen transport	2	41	9	11484	0.000441
**GO:0030168**	platelet activation	4	41	111	11484	0.000636
**GO:0031674**	I band	2	41	12	11484	0.000802
**GO:0001968**	fibronectin binding	2	41	13	11484	0.000946
**GO:0042744**	hydrogen peroxide catabolic process	2	41	13	11484	0.000946
**GO:0015701**	bicarbonate transport	2	41	14	11484	0.001101
**GO:0055010**	ventricular cardiac muscle tissue morphogenesis	2	41	14	11484	0.001101
**GO:0004601**	peroxidase activity	2	41	15	11484	0.001268
**GO:0010942**	positive regulation of cell death	2	41	16	11484	0.001446
**GO:0019825**	oxygen binding	2	41	16	11484	0.001446
**GO:0043531**	ADP binding	2	41	17	11484	0.001635
**GO:0045765**	regulation of angiogenesis	2	41	17	11484	0.001635
**GO:0010243**	response to organonitrogen compound	2	41	20	11484	0.002269
**GO:0030308**	negative regulation of cell growth	3	41	75	11484	0.002387
**GO:0007596**	blood coagulation	5	41	273	11484	0.002723
**GO:0042771**	intrinsic apoptotic signaling pathway in response to DNA damage by p53 class mediator	2	41	22	11484	0.002746
**GO:0030016**	myofibril	2	41	23	11484	0.003
**GO:0001869**	negative regulation of complement activation, lectin pathway	1	41	1	11484	0.00357
**GO:0005602**	complement component C1 complex	1	41	1	11484	0.00357

**GO:0008112**	nicotinamide N-methyltransferase activity	1	41	1	11484	0.00357
**GO:0008934**	inositol monophosphate 1-phosphatase activity	1	41	1	11484	0.00357
**GO:0009991**	response to extracellular stimulus	1	41	1	11484	0.00357
**GO:0014734**	skeletal muscle hypertrophy	1	41	1	11484	0.00357
**GO:0019912**	cyclin-dependent protein kinase activating kinase activity	1	41	1	11484	0.00357
**GO:0031109**	microtubule polymerization or depolymerization	1	41	1	11484	0.00357
**GO:0033371**	T cell secretory granule organization	1	41	1	11484	0.00357
**GO:0033373**	maintenance of protease location in mast cell secretory granule	1	41	1	11484	0.00357
**GO:0033382**	maintenance of granzyme B location in T cell secretory granule	1	41	1	11484	0.00357
**GO:0035491**	positive regulation of leukotriene production involved in inflammatory response	1	41	1	11484	0.00357
**GO:0038133**	ERBB2-ERBB3 signaling pathway	1	41	1	11484	0.00357
**GO:0042694**	muscle cell fate specification	1	41	1	11484	0.00357
**GO:0043120**	tumor necrosis factor binding	1	41	1	11484	0.00357
**GO:0043241**	protein complex disassembly	1	41	1	11484	0.00357
**GO:0044027**	hypermethylation of CpG island	1	41	1	11484	0.00357
**GO:0051089**	constitutive protein ectodomain proteolysis	1	41	1	11484	0.00357
**GO:0052832**	inositol monophosphate 3-phosphatase activity	1	41	1	11484	0.00357
**GO:0052833**	inositol monophosphate 4-phosphatase activity	1	41	1	11484	0.00357
**GO:0061041**	regulation of wound healing	1	41	1	11484	0.00357
**GO:0061044**	negative regulation of vascular wound healing	1	41	1	11484	0.00357
**GO:0070557**	PCNA-p21 complex	1	41	1	11484	0.00357
**GO:0071888**	macrophage apoptotic process	1	41	1	11484	0.00357
**GO:2000751**	histone H3-T3 phosphorylation involved in chromosome passenger complex localization to kinetochore	1	41	1	11484	0.00357
**GO:0046872**	metal ion binding	11	41	1254	11484	0.003605
**GO:0060348**	bone development	2	41	30	11484	0.005078
**GO:0031100**	organ regeneration	2	41	31	11484	0.005416

**GO:0051291**	protein heterooligomerization	2	41	31	11484	0.005416
**GO:0001300**	chronological cell aging	1	41	2	11484	0.007128
**GO:0005853**	eukaryotic translation elongation factor 1 complex	1	41	2	11484	0.007128
**GO:0006021**	inositol biosynthetic process	1	41	2	11484	0.007128
**GO:0006931**	substrate-dependent cell migration, cell attachment to substrate	1	41	2	11484	0.007128
**GO:0008626**	granzyme-mediated apoptotic signaling pathway	1	41	2	11484	0.007128
**GO:0010757**	negative regulation of plasminogen activation	1	41	2	11484	0.007128
**GO:0019966**	interleukin-1 binding	1	41	2	11484	0.007128
**GO:0030185**	nitric oxide transport	1	41	2	11484	0.007128
**GO:0030492**	hemoglobin binding	1	41	2	11484	0.007128
**GO:0032027**	myosin light chain binding	1	41	2	11484	0.007128
**GO:0032432**	actin filament bundle	1	41	2	11484	0.007128
**GO:0033364**	mast cell secretory granule organization	1	41	2	11484	0.007128
**GO:0036021**	endolysosome lumen	1	41	2	11484	0.007128
**GO:0043259**	laminin-10 complex	1	41	2	11484	0.007128
**GO:0043260**	laminin-11 complex	1	41	2	11484	0.007128
**GO:0043503**	skeletal muscle fiber adaptation	1	41	2	11484	0.007128
**GO:0046498**	S-adenosylhomocysteine metabolic process	1	41	2	11484	0.007128
**GO:0046499**	S-adenosylmethioninamine metabolic process	1	41	2	11484	0.007128
**GO:0051088**	PMA-inducible membrane protein ectodomain proteolysis	1	41	2	11484	0.007128
**GO:0051718**	DNA (cytosine-5-)-methyltransferase activity, acting on CpG substrates	1	41	2	11484	0.007128
**GO:0070293**	renal absorption	1	41	2	11484	0.007128
**GO:0072354**	histone kinase activity (H3-T3 specific)	1	41	2	11484	0.007128
**GO:0090116**	C-5 methylation of cytosine	1	41	2	11484	0.007128
**GO:0090400**	stress-induced premature senescence	1	41	2	11484	0.007128
**GO:0097187**	dentinogenesis	1	41	2	11484	0.007128
**GO:1902532**	negative regulation of intracellular signal transduction	1	41	2	11484	0.007128
**GO:2000098**	negative regulation of smooth muscle cell-matrix adhesion	1	41	2	11484	0.007128
**GO:2000278**	regulation of DNA biosynthetic process	1	41	2	11484	0.007128
**GO:0015629**	actin cytoskeleton	3	41	115	11484	0.007908

**GO:0000775**	chromosome, centromeric region	2	41	38	11484	0.00806
**GO:0006468**	protein phosphorylation	5	41	365	11484	0.009238
**GO:0002020**	protease binding	2	41	41	11484	0.009338
**GO:0051384**	response to glucocorticoid	2	41	42	11484	0.009783
**GO:0050679**	positive regulation of epithelial cell proliferation	2	41	43	11484	0.010238
**GO:0003886**	DNA (cytosine-5-)-methyltransferase activity	1	41	3	11484	0.010673
**GO:0004111**	creatine kinase activity	1	41	3	11484	0.010673
**GO:0005606**	laminin-1 complex	1	41	3	11484	0.010673
**GO:0005861**	troponin complex	1	41	3	11484	0.010673
**GO:0007021**	tubulin complex assembly	1	41	3	11484	0.010673
**GO:0010424**	DNA methylation on cytosine within a CG sequence	1	41	3	11484	0.010673
**GO:0019959**	interleukin-8 binding	1	41	3	11484	0.010673
**GO:0032387**	negative regulation of intracellular transport	1	41	3	11484	0.010673
**GO:0032876**	negative regulation of DNA endoreduplication	1	41	3	11484	0.010673
**GO:0033591**	response to L-ascorbic acid	1	41	3	11484	0.010673
**GO:0033629**	negative regulation of cell adhesion mediated by integrin	1	41	3	11484	0.010673
**GO:0038031**	non-canonical Wnt signaling pathway via JNK cascade	1	41	3	11484	0.010673
**GO:0042588**	zymogen granule	1	41	3	11484	0.010673
**GO:0060574**	intestinal epithelial cell maturation	1	41	3	11484	0.010673
**GO:0071459**	protein localization to chromosome, centromeric region	1	41	3	11484	0.010673
**GO:0090231**	regulation of spindle checkpoint	1	41	3	11484	0.010673
**GO:0005604**	basement membrane	2	41	44	11484	0.010701
**GO:0030574**	collagen catabolic process	2	41	45	11484	0.011174
**GO:0005578**	proteinaceous extracellular matrix	3	41	136	11484	0.012473
**GO:0007517**	muscle organ development	2	41	48	11484	0.012646
**GO:0007267**	cell-cell signaling	3	41	140	11484	0.013482
**GO:0031175**	neuron projection development	2	41	50	11484	0.013672
**GO:0004522**	pancreatic ribonuclease activity	1	41	4	11484	0.014206
**GO:0005865**	striated muscle thin filament	1	41	4	11484	0.014206
**GO:0010469**	regulation of receptor activity	1	41	4	11484	0.014206
**GO:0010820**	positive regulation of T cell chemotaxis	1	41	4	11484	0.014206
**GO:0021532**	neural tube patterning	1	41	4	11484	0.014206

**GO:0030195**	negative regulation of blood coagulation	1	41	4	11484	0.014206
**GO:0030240**	skeletal muscle thin filament assembly	1	41	4	11484	0.014206
**GO:0032036**	myosin heavy chain binding	1	41	4	11484	0.014206
**GO:0033158**	regulation of protein import into nucleus, translocation	1	41	4	11484	0.014206
**GO:0045162**	clustering of voltage-gated sodium channels	1	41	4	11484	0.014206
**GO:0045322**	unmethylated CpG binding	1	41	4	11484	0.014206
**GO:0048260**	positive regulation of receptor-mediated endocytosis	1	41	4	11484	0.014206
**GO:0050847**	progesterone receptor signaling pathway	1	41	4	11484	0.014206
**GO:0060561**	apoptotic process involved in morphogenesis	1	41	4	11484	0.014206
**GO:0097038**	perinuclear endoplasmic reticulum	1	41	4	11484	0.014206
**GO:0005694**	chromosome	2	41	54	11484	0.015828
**GO:0004867**	serine-type endopeptidase inhibitor activity	2	41	57	11484	0.017535
**GO:0000307**	cyclin-dependent protein kinase holoenzyme complex	1	41	5	11484	0.017727
**GO:0006600**	creatine metabolic process	1	41	5	11484	0.017727
**GO:0009629**	response to gravity	1	41	5	11484	0.017727
**GO:0010941**	regulation of cell death	1	41	5	11484	0.017727
**GO:0035024**	negative regulation of Rho protein signal transduction	1	41	5	11484	0.017727
**GO:0042117**	monocyte activation	1	41	5	11484	0.017727
**GO:0043045**	DNA methylation involved in embryo development	1	41	5	11484	0.017727
**GO:0043208**	glycosphingolipid binding	1	41	5	11484	0.017727
**GO:0043394**	proteoglycan binding	1	41	5	11484	0.017727
**GO:0045820**	negative regulation of glycolytic process	1	41	5	11484	0.017727
**GO:0051918**	negative regulation of fibrinolysis	1	41	5	11484	0.017727
**GO:0060047**	heart contraction	1	41	5	11484	0.017727
**GO:0071480**	cellular response to gamma radiation	1	41	5	11484	0.017727
**GO:0097197**	tetraspanin-enriched microdomain	1	41	5	11484	0.017727
**GO:1901216**	positive regulation of neuron death	1	41	5	11484	0.017727
**GO:0005200**	structural constituent of cytoskeleton	2	41	58	11484	0.018121

**GO:0016605**	PML body	2	41	58	11484	0.018121
**GO:0006928**	cellular component movement	2	41	61	11484	0.019927
**GO:0001649**	osteoblast differentiation	2	41	62	11484	0.020545
**GO:0005798**	Golgi-associated vesicle	1	41	6	11484	0.021235
**GO:0006346**	methylation-dependent chromatin silencing	1	41	6	11484	0.021235
**GO:0007062**	sister chromatid cohesion	1	41	6	11484	0.021235
**GO:0007064**	mitotic sister chromatid cohesion	1	41	6	11484	0.021235
**GO:0016004**	phospholipase activator activity	1	41	6	11484	0.021235
**GO:0046967**	cytosol to ER transport	1	41	6	11484	0.021235
**GO:0050687**	negative regulation of defense response to virus	1	41	6	11484	0.021235
**GO:0051901**	positive regulation of mitochondrial depolarization	1	41	6	11484	0.021235
**GO:0061045**	negative regulation of wound healing	1	41	6	11484	0.021235
**GO:0010629**	negative regulation of gene expression	2	41	64	11484	0.021805
**GO:0008168**	methyltransferase activity	2	41	68	11484	0.02442
**GO:0001953**	negative regulation of cell-matrix adhesion	1	41	7	11484	0.024732
**GO:0010165**	response to X-ray	1	41	7	11484	0.024732
**GO:0010226**	response to lithium ion	1	41	7	11484	0.024732
**GO:0010288**	response to lead ion	1	41	7	11484	0.024732
**GO:0015197**	peptide transporter activity	1	41	7	11484	0.024732
**GO:0016892**	endoribonuclease activity, producing 3'-phosphomonoesters	1	41	7	11484	0.024732
**GO:0030194**	positive regulation of blood coagulation	1	41	7	11484	0.024732
**GO:0043046**	DNA methylation involved in gamete generation	1	41	7	11484	0.024732
**GO:0050710**	negative regulation of cytokine secretion	1	41	7	11484	0.024732
**GO:0050880**	regulation of blood vessel size	1	41	7	11484	0.024732
**GO:0055003**	cardiac myofibril assembly	1	41	7	11484	0.024732
**GO:0071243**	cellular response to arsenic-containing substance	1	41	7	11484	0.024732
**GO:0004861**	cyclin-dependent protein serine/threonine kinase inhibitor activity	1	41	8	11484	0.028216
**GO:0007597**	blood coagulation, intrinsic pathway	1	41	8	11484	0.028216
**GO:0010801**	negative regulation of peptidyl-threonine phosphorylation	1	41	8	11484	0.028216

**GO:0014912**	negative regulation of smooth muscle cell migration	1	41	8	11484	0.028216
**GO:0015833**	peptide transport	1	41	8	11484	0.028216
**GO:0023029**	MHC class Ib protein binding	1	41	8	11484	0.028216
**GO:0031581**	hemidesmosome assembly	1	41	8	11484	0.028216
**GO:0031672**	A band	1	41	8	11484	0.028216
**GO:0031988**	membrane-bounded vesicle	1	41	8	11484	0.028216
**GO:0043068**	positive regulation of programmed cell death	1	41	8	11484	0.028216
**GO:0043422**	protein kinase B binding	1	41	8	11484	0.028216
**GO:0045653**	negative regulation of megakaryocyte differentiation	1	41	8	11484	0.028216
**GO:0046685**	response to arsenic-containing substance	1	41	8	11484	0.028216
**GO:0046855**	inositol phosphate dephosphorylation	1	41	8	11484	0.028216
**GO:0051497**	negative regulation of stress fiber assembly	1	41	8	11484	0.028216
**GO:0051894**	positive regulation of focal adhesion assembly	1	41	8	11484	0.028216
**GO:0055093**	response to hyperoxia	1	41	8	11484	0.028216
**GO:0071850**	mitotic cell cycle arrest	1	41	8	11484	0.028216
**GO:0003779**	actin binding	3	41	186	11484	0.028408
**GO:0005515**	protein binding	27	41	5733	11484	0.028897
**GO:0030335**	positive regulation of cell migration	2	41	75	11484	0.029286
**GO:0048015**	phosphatidylinositol-mediated signaling	2	41	75	11484	0.029286
**GO:0005102**	receptor binding	3	41	191	11484	0.030402
**GO:0003746**	translation elongation factor activity	1	41	9	11484	0.031687
**GO:0005212**	structural constituent of eye lens	1	41	9	11484	0.031687
**GO:0006796**	phosphate-containing compound metabolic process	1	41	9	11484	0.031687
**GO:0019060**	intracellular transport of viral protein in host cell	1	41	9	11484	0.031687
**GO:0031432**	titin binding	1	41	9	11484	0.031687
**GO:0033137**	negative regulation of peptidyl-serine phosphorylation	1	41	9	11484	0.031687
**GO:0035574**	histone H4-K20 demethylation	1	41	9	11484	0.031687
**GO:0035575**	histone demethylase activity (H4-K20 specific)	1	41	9	11484	0.031687
**GO:0042825**	TAP complex	1	41	9	11484	0.031687
**GO:0043292**	contractile fiber	1	41	9	11484	0.031687
**GO:0043408**	regulation of MAPK cascade	1	41	9	11484	0.031687

**GO:0045662**	negative regulation of myoblast differentiation	1	41	9	11484	0.031687
**GO:0045773**	positive regulation of axon extension	1	41	9	11484	0.031687
**GO:0048741**	skeletal muscle fiber development	1	41	9	11484	0.031687
**GO:0051412**	response to corticosterone	1	41	9	11484	0.031687
**GO:0070206**	protein trimerization	1	41	9	11484	0.031687
**GO:0071364**	cellular response to epidermal growth factor stimulus	1	41	9	11484	0.031687
**GO:0010951**	negative regulation of endopeptidase activity	2	41	79	11484	0.032227
**GO:0020037**	heme binding	2	41	81	11484	0.03374
**GO:0000242**	pericentriolar material	1	41	10	11484	0.035147
**GO:0009968**	negative regulation of signal transduction	1	41	10	11484	0.035147
**GO:0019885**	antigen processing and presentation of endogenous peptide antigen via MHC class I	1	41	10	11484	0.035147
**GO:0030502**	negative regulation of bone mineralization	1	41	10	11484	0.035147
**GO:0030863**	cortical cytoskeleton	1	41	10	11484	0.035147
**GO:0031941**	filamentous actin	1	41	10	11484	0.035147
**GO:0032757**	positive regulation of interleukin-8 production	1	41	10	11484	0.035147
**GO:0033268**	node of Ranvier	1	41	10	11484	0.035147
**GO:0042629**	mast cell granule	1	41	10	11484	0.035147
**GO:0042730**	fibrinolysis	1	41	10	11484	0.035147
**GO:0046979**	TAP2 binding	1	41	10	11484	0.035147
**GO:0048747**	muscle fiber development	1	41	10	11484	0.035147
**GO:0071889**	14-3-3 protein binding	1	41	10	11484	0.035147
**GO:2000378**	negative regulation of reactive oxygen species metabolic process	1	41	10	11484	0.035147
**GO:0042127**	regulation of cell proliferation	2	41	86	11484	0.03764
**GO:0002102**	podosome	1	41	11	11484	0.038595
**GO:0003785**	actin monomer binding	1	41	11	11484	0.038595
**GO:0005720**	nuclear heterochromatin	1	41	11	11484	0.038595
**GO:0071901**	negative regulation of protein serine/threonine kinase activity	1	41	11	11484	0.038595
**GO:0005506**	iron ion binding	2	41	88	11484	0.039247
**GO:0001666**	response to hypoxia	2	41	89	11484	0.04006
**GO:0005856**	cytoskeleton	3	41	214	11484	0.040505
**GO:0005605**	basal lamina	1	41	12	11484	0.042031
**GO:0006349**	regulation of gene expression by genetic imprinting	1	41	12	11484	0.042031

**GO:0022011**	myelination in peripheral nervous system	1	41	12	11484	0.042031
**GO:0031214**	biomineral tissue development	1	41	12	11484	0.042031
**GO:0031668**	cellular response to extracellular stimulus	1	41	12	11484	0.042031
**GO:0045736**	negative regulation of cyclin-dependent protein serine/threonine kinase activity	1	41	12	11484	0.042031
**GO:2000352**	negative regulation of endothelial cell apoptotic process	1	41	12	11484	0.042031
**GO:0017022**	myosin binding	1	41	13	11484	0.045454
**GO:0030332**	cyclin binding	1	41	13	11484	0.045454
**GO:0032007**	negative regulation of TOR signaling	1	41	13	11484	0.045454
**GO:0046978**	TAP1 binding	1	41	13	11484	0.045454
**GO:1900026**	positive regulation of substrate adhesion-dependent cell spreading	1	41	13	11484	0.045454
**GO:0051607**	defense response to virus	2	41	97	11484	0.046791
**GO:0000278**	mitotic cell cycle	3	41	228	11484	0.047392
**GO:0007173**	epidermal growth factor receptor signaling pathway	2	41	98	11484	0.04766
**GO:0042803**	protein homodimerization activity	4	41	387	11484	0.048293
**GO:0006336**	DNA replication-independent nucleosome assembly	1	41	14	11484	0.048866
**GO:0042326**	negative regulation of phosphorylation	1	41	14	11484	0.048866
**GO:0046686**	response to cadmium ion	1	41	14	11484	0.048866
**GO:0050840**	extracellular matrix binding	1	41	14	11484	0.048866
**GO:0090026**	positive regulation of monocyte chemotaxis	1	41	14	11484	0.048866
**GO:0090398**	cellular senescence	1	41	14	11484	0.048866
**GO:1902042**	negative regulation of extrinsic apoptotic signaling pathway via death domain receptors	1	41	14	11484	0.048866
**GO:0009615**	response to virus	2	41	100	11484	0.049416
**GO:0005520**	insulin-like growth factor binding	1	41	15	11484	0.052266
**GO:0006509**	membrane protein ectodomain proteolysis	1	41	15	11484	0.052266
**GO:0051297**	centrosome organization	1	41	15	11484	0.052266
**GO:2000134**	negative regulation of G1/S transition of mitotic cell cycle	1	41	15	11484	0.052266
**GO:0007220**	Notch receptor processing	1	41	16	11484	0.055654

**GO:0019838**	growth factor binding	1	41	16	11484	0.055654
**GO:2000379**	positive regulation of reactive oxygen species metabolic process	1	41	16	11484	0.055654
**GO:0004866**	endopeptidase inhibitor activity	1	41	17	11484	0.05903
**GO:0006335**	DNA replication-dependent nucleosome assembly	1	41	17	11484	0.05903
**GO:0007346**	regulation of mitotic cell cycle	1	41	17	11484	0.05903
**GO:0042288**	MHC class I protein binding	1	41	17	11484	0.05903
**GO:0050829**	defense response to Gram-negative bacterium	1	41	17	11484	0.05903
**GO:0051496**	positive regulation of stress fiber assembly	1	41	17	11484	0.05903
**GO:0071479**	cellular response to ionizing radiation	1	41	17	11484	0.05903
**GO:0005634**	nucleus	17	41	3346	11484	0.061644
**GO:0000792**	heterochromatin	1	41	18	11484	0.062395
**GO:0002088**	lens development in camera-type eye	1	41	18	11484	0.062395
**GO:0006352**	DNA-templated transcription, initiation	1	41	18	11484	0.062395
**GO:0007492**	endoderm development	1	41	18	11484	0.062395
**GO:2001238**	positive regulation of extrinsic apoptotic signaling pathway	1	41	18	11484	0.062395
**GO:0007507**	heart development	2	41	115	11484	0.063303
**GO:0001525**	angiogenesis	2	41	117	11484	0.065245
**GO:0030971**	receptor tyrosine kinase binding	1	41	19	11484	0.065747
**GO:0034612**	response to tumor necrosis factor	1	41	19	11484	0.065747
**GO:0048545**	response to steroid hormone	1	41	19	11484	0.065747
**GO:0060048**	cardiac muscle contraction	1	41	19	11484	0.065747
**GO:0009986**	cell surface	3	41	263	11484	0.066957
**GO:0007162**	negative regulation of cell adhesion	1	41	20	11484	0.069088
**GO:0007566**	embryo implantation	1	41	20	11484	0.069088
**GO:0034080**	centromere-specific nucleosome assembly	1	41	20	11484	0.069088
**GO:0034446**	substrate adhesion-dependent cell spreading	1	41	20	11484	0.069088
**GO:0046854**	phosphatidylinositol phosphorylation	1	41	20	11484	0.069088
**GO:0009411**	response to UV	1	41	21	11484	0.072418
**GO:0016485**	protein processing	1	41	21	11484	0.072418
**GO:0048863**	stem cell differentiation	1	41	21	11484	0.072418
**GO:0051258**	protein polymerization	1	41	21	11484	0.072418

**GO:0072593**	reactive oxygen species metabolic process	1	41	21	11484	0.072418
**GO:0097191**	extrinsic apoptotic signaling pathway	1	41	21	11484	0.072418
**GO:0006306**	DNA methylation	1	41	22	11484	0.075735
**GO:0030890**	positive regulation of B cell proliferation	1	41	22	11484	0.075735
**GO:0097192**	extrinsic apoptotic signaling pathway in absence of ligand	1	41	22	11484	0.075735
**GO:0005794**	Golgi apparatus	4	41	453	11484	0.076936
**GO:0005109**	frizzled binding	1	41	23	11484	0.079041
**GO:0030514**	negative regulation of BMP signaling pathway	1	41	23	11484	0.079041
**GO:0048839**	inner ear development	1	41	23	11484	0.079041
**GO:0034605**	cellular response to heat	1	41	24	11484	0.082336
**GO:0035176**	social behavior	1	41	24	11484	0.082336
**GO:0035914**	skeletal muscle cell differentiation	1	41	24	11484	0.082336
**GO:0071230**	cellular response to amino acid stimulus	1	41	24	11484	0.082336
**GO:0005791**	rough endoplasmic reticulum	1	41	25	11484	0.085619
**GO:0048146**	positive regulation of fibroblast proliferation	1	41	25	11484	0.085619
**GO:0002250**	adaptive immune response	1	41	26	11484	0.088891
**GO:0009103**	lipopolysaccharide biosynthetic process	1	41	26	11484	0.088891
**GO:0014068**	positive regulation of phosphatidylinositol 3-kinase signaling	1	41	26	11484	0.088891
**GO:0048306**	calcium-dependent protein binding	1	41	26	11484	0.088891
**GO:0004175**	endopeptidase activity	1	41	27	11484	0.092151
**GO:0006958**	complement activation, classical pathway	1	41	27	11484	0.092151
**GO:0007017**	microtubule-based process	1	41	27	11484	0.092151
**GO:0016049**	cell growth	1	41	27	11484	0.092151
**GO:0004672**	protein kinase activity	3	41	302	11484	0.092441
**GO:0003007**	heart morphogenesis	1	41	28	11484	0.0954
**GO:0005884**	actin filament	1	41	28	11484	0.0954
**GO:0050729**	positive regulation of inflammatory response	1	41	28	11484	0.0954
**GO:0051591**	response to cAMP	1	41	28	11484	0.0954
**GO:0051603**	proteolysis involved in cellular protein catabolic process	1	41	28	11484	0.0954
**GO:0006508**	proteolysis	3	41	309	11484	0.097394

**GO:0016772**	transferase activity, transferring phosphorus-containing groups	3	41	309	11484	0.097394
**GO:0048011**	neurotrophin TRK receptor signaling pathway	2	41	148	11484	0.097698
**GO:0005737**	cytoplasm	14	41	2791	11484	0.101339
**GO:0008092**	cytoskeletal protein binding	1	41	30	11484	0.101863
**GO:0019827**	stem cell maintenance	1	41	30	11484	0.101863
**GO:0000228**	nuclear chromosome	1	41	31	11484	0.105078
**GO:0004197**	cysteine-type endopeptidase activity	1	41	31	11484	0.105078
**GO:0007126**	meiotic nuclear division	1	41	31	11484	0.105078
**GO:0008236**	serine-type peptidase activity	1	41	31	11484	0.105078
**GO:0045429**	positive regulation of nitric oxide biosynthetic process	1	41	31	11484	0.105078
**GO:0071363**	cellular response to growth factor stimulus	1	41	31	11484	0.105078
**GO:0005758**	mitochondrial intermembrane space	1	41	32	11484	0.108282
**GO:0016459**	myosin complex	1	41	32	11484	0.108282
**GO:0043647**	inositol phosphate metabolic process	1	41	32	11484	0.108282
**GO:0051403**	stress-activated MAPK cascade	1	41	32	11484	0.108282
**GO:0097193**	intrinsic apoptotic signaling pathway	1	41	32	11484	0.108282
**GO:0003924**	GTPase activity	2	41	159	11484	0.110132
**GO:0005201**	extracellular matrix structural constituent	1	41	33	11484	0.111475
**GO:0006956**	complement activation	1	41	33	11484	0.111475
**GO:0009612**	response to mechanical stimulus	1	41	33	11484	0.111475
**GO:0022627**	cytosolic small ribosomal subunit	1	41	33	11484	0.111475
**GO:0045111**	intermediate filament cytoskeleton	1	41	33	11484	0.111475
**GO:0045860**	positive regulation of protein kinase activity	1	41	33	11484	0.111475
**GO:0034097**	response to cytokine	1	41	34	11484	0.114656
**GO:0042626**	ATPase activity, coupled to transmembrane movement of substances	1	41	34	11484	0.114656
**GO:0043434**	response to peptide hormone	1	41	34	11484	0.114656
**GO:0006184**	GTP catabolic process	2	41	163	11484	0.114754
**GO:0051592**	response to calcium ion	1	41	35	11484	0.117826
**GO:0000079**	regulation of cyclin-dependent protein serine/threonine kinase activity	1	41	36	11484	0.120985

**GO:0008217**	regulation of blood pressure	1	41	36	11484	0.120985
**GO:0006887**	exocytosis	1	41	37	11484	0.124133
**GO:0000723**	telomere maintenance	1	41	38	11484	0.12727
**GO:0001558**	regulation of cell growth	1	41	38	11484	0.12727
**GO:0008009**	chemokine activity	1	41	38	11484	0.12727
**GO:0043010**	camera-type eye development	1	41	38	11484	0.12727
**GO:0043154**	negative regulation of cysteine-type endopeptidase activity involved in apoptotic process	1	41	38	11484	0.12727
**GO:0030336**	negative regulation of cell migration	1	41	39	11484	0.130397
**GO:0090502**	RNA phosphodiester bond hydrolysis, endonucleolytic	1	41	39	11484	0.130397
**GO:0007265**	Ras protein signal transduction	1	41	40	11484	0.133512
**GO:0032355**	response to estradiol	1	41	40	11484	0.133512
**GO:0015030**	Cajal body	1	41	41	11484	0.136616
**GO:0030097**	hemopoiesis	1	41	41	11484	0.136616
**GO:0030176**	integral component of endoplasmic reticulum membrane	1	41	41	11484	0.136616
**GO:0042733**	embryonic digit morphogenesis	1	41	41	11484	0.136616
**GO:0043202**	lysosomal lumen	1	41	41	11484	0.136616
**GO:0000785**	chromatin	1	41	42	11484	0.13971
**GO:0006977**	DNA damage response, signal transduction by p53 class mediator resulting in cell cycle arrest	1	41	42	11484	0.13971
**GO:0007229**	integrin-mediated signaling pathway	1	41	42	11484	0.13971
**GO:0030307**	positive regulation of cell growth	1	41	42	11484	0.13971
**GO:0043085**	positive regulation of catalytic activity	1	41	42	11484	0.13971
**GO:0042493**	response to drug	2	41	186	11484	0.142242
**GO:0030512**	negative regulation of transforming growth factor beta receptor signaling pathway	1	41	43	11484	0.142792
**GO:0060326**	cell chemotaxis	1	41	43	11484	0.142792
**GO:0001726**	ruffle	1	41	44	11484	0.145864
**GO:0042393**	histone binding	1	41	44	11484	0.145864
**GO:0045766**	positive regulation of angiogenesis	1	41	44	11484	0.145864
**GO:0051897**	positive regulation of protein kinase B signaling	1	41	44	11484	0.145864
**GO:0005581**	collagen trimer	1	41	45	11484	0.148925
**GO:0045471**	response to ethanol	1	41	45	11484	0.148925

**GO:0019901**	protein kinase binding	2	41	192	11484	0.149635
**GO:0016363**	nuclear matrix	1	41	46	11484	0.151976
**GO:0042060**	wound healing	1	41	48	11484	0.158045
**GO:0016773**	phosphotransferase activity, alcohol group as acceptor	1	41	49	11484	0.161063
**GO:0045087**	innate immune response	3	41	392	11484	0.163535
**GO:0000786**	nucleosome	1	41	50	11484	0.164071
**GO:0006874**	cellular calcium ion homeostasis	1	41	51	11484	0.167069
**GO:0009636**	response to toxic substance	1	41	51	11484	0.167069
**GO:0009791**	post-embryonic development	1	41	51	11484	0.167069
**GO:0005743**	mitochondrial inner membrane	2	41	207	11484	0.168446
**GO:0005178**	integrin binding	1	41	52	11484	0.170056
**GO:0001503**	ossification	1	41	53	11484	0.173032
**GO:0008234**	cysteine-type peptidase activity	1	41	54	11484	0.175998
**GO:0042605**	peptide antigen binding	1	41	54	11484	0.175998
**GO:0005622**	intracellular	5	41	838	11484	0.176434
**GO:0044281**	small molecule metabolic process	5	41	839	11484	0.177039
**GO:0014069**	postsynaptic density	1	41	55	11484	0.178954
**GO:0032259**	methylation	1	41	55	11484	0.178954
**GO:0071222**	cellular response to lipopolysaccharide	1	41	55	11484	0.178954
**GO:0001764**	neuron migration	1	41	57	11484	0.184835
**GO:0006417**	regulation of translation	1	41	57	11484	0.184835
**GO:0030018**	Z disc	1	41	57	11484	0.184835
**GO:0008237**	metallopeptidase activity	1	41	58	11484	0.187759
**GO:0010033**	response to organic substance	1	41	58	11484	0.187759
**GO:0042384**	cilium assembly	1	41	58	11484	0.187759
**GO:0003682**	chromatin binding	2	41	223	11484	0.188944
**GO:0003700**	sequence-specific DNA binding transcription factor activity	4	41	636	11484	0.190114
**GO:0005814**	centriole	1	41	59	11484	0.190674
**GO:0005509**	calcium ion binding	3	41	423	11484	0.1911
**GO:0005525**	GTP binding	2	41	227	11484	0.194126
**GO:0005730**	nucleolus	3	41	427	11484	0.194745
**GO:0002224**	toll-like receptor signaling pathway	1	41	61	11484	0.196473
**GO:0006414**	translational elongation	1	41	61	11484	0.196473
**GO:0004674**	protein serine/threonine kinase activity	2	41	230	11484	0.198025
**GO:0043234**	protein complex	2	41	232	11484	0.20063
**GO:0006816**	calcium ion transport	1	41	63	11484	0.202231
**GO:0017124**	SH3 domain binding	1	41	63	11484	0.202231
**GO:0030324**	lung development	1	41	64	11484	0.205094
**GO:0007283**	spermatogenesis	2	41	241	11484	0.212406

**GO:0051091**	positive regulation of sequence-specific DNA binding transcription factor activity	1	41	67	11484	0.213626
**GO:0006351**	transcription, DNA-templated	6	41	1137	11484	0.215774
**GO:0006461**	protein complex assembly	1	41	68	11484	0.21645
**GO:0043231**	intracellular membrane-bounded organelle	2	41	245	11484	0.217664
**GO:0030182**	neuron differentiation	1	41	69	11484	0.219264
**GO:0005741**	mitochondrial outer membrane	1	41	70	11484	0.222068
**GO:0051082**	unfolded protein binding	1	41	70	11484	0.222068
**GO:0005524**	ATP binding	5	41	913	11484	0.224031
**GO:0004222**	metalloendopeptidase activity	1	41	71	11484	0.224863
**GO:0035556**	intracellular signal transduction	2	41	251	11484	0.225574
**GO:0002479**	antigen processing and presentation of exogenous peptide antigen via MHC class I, TAP-dependent	1	41	72	11484	0.227647
**GO:0006805**	xenobiotic metabolic process	1	41	72	11484	0.227647
**GO:0007568**	aging	1	41	72	11484	0.227647
**GO:0007179**	transforming growth factor beta receptor signaling pathway	1	41	73	11484	0.230422
**GO:0032496**	response to lipopolysaccharide	1	41	73	11484	0.230422
**GO:0006935**	chemotaxis	1	41	74	11484	0.233187
**GO:0008201**	heparin binding	1	41	74	11484	0.233187
**GO:0016477**	cell migration	1	41	74	11484	0.233187
**GO:0051726**	regulation of cell cycle	1	41	74	11484	0.233187
**GO:0014070**	response to organic cyclic compound	1	41	75	11484	0.235943
**GO:0015630**	microtubule cytoskeleton	1	41	75	11484	0.235943
**GO:0042590**	antigen processing and presentation of exogenous peptide antigen via MHC class I	1	41	75	11484	0.235943
**GO:0007155**	cell adhesion	2	41	259	11484	0.236159
**GO:0007219**	Notch signaling pathway	1	41	76	11484	0.238688
**GO:0007050**	cell cycle arrest	1	41	77	11484	0.241425
**GO:0031410**	cytoplasmic vesicle	1	41	77	11484	0.241425
**GO:0006952**	defense response	1	41	78	11484	0.244151
**GO:0016023**	cytoplasmic membrane-bounded vesicle	1	41	78	11484	0.244151
**GO:0003677**	DNA binding	6	41	1185	11484	0.244366
**GO:0005819**	spindle	1	41	80	11484	0.249576
**GO:0005929**	cilium	1	41	82	11484	0.254962
**GO:0008543**	fibroblast growth factor receptor signaling pathway	1	41	83	11484	0.257641
**GO:0000082**	G1/S transition of mitotic cell cycle	1	41	84	11484	0.260311

**GO:0006334**	nucleosome assembly	1	41	84	11484	0.260311
**GO:0006325**	chromatin organization	1	41	85	11484	0.262971
**GO:0051092**	positive regulation of NF-kappaB transcription factor activity	1	41	85	11484	0.262971
**GO:0000086**	G2/M transition of mitotic cell cycle	1	41	88	11484	0.270896
**GO:0002474**	antigen processing and presentation of peptide antigen via MHC class I	1	41	89	11484	0.273519
**GO:0016301**	kinase activity	1	41	90	11484	0.276133
**GO:0004252**	serine-type endopeptidase activity	1	41	91	11484	0.278738
**GO:0019886**	antigen processing and presentation of exogenous peptide antigen via MHC class II	1	41	91	11484	0.278738
**GO:0043123**	positive regulation of I-kappaB kinase/NF-kappaB signaling	1	41	91	11484	0.278738
**GO:0038095**	Fc-epsilon receptor signaling pathway	1	41	97	11484	0.294175
**GO:0006457**	protein folding	1	41	98	11484	0.296717
**GO:0008017**	microtubule binding	1	41	98	11484	0.296717
**GO:0051260**	protein homooligomerization	1	41	98	11484	0.296717
**GO:0006974**	cellular response to DNA damage stimulus	1	41	99	11484	0.299249
**GO:0051056**	regulation of small GTPase mediated signal transduction	1	41	101	11484	0.304287
**GO:0032403**	protein complex binding	1	41	102	11484	0.306793
**GO:0006367**	transcription initiation from RNA polymerase II promoter	1	41	104	11484	0.311779
**GO:0003714**	transcription corepressor activity	1	41	105	11484	0.314258
**GO:0043066**	negative regulation of apoptotic process	2	41	319	11484	0.315952
**GO:0034641**	cellular nitrogen compound metabolic process	1	41	107	11484	0.319191
**GO:0019882**	antigen processing and presentation	1	41	109	11484	0.324089
**GO:0016887**	ATPase activity	1	41	110	11484	0.326526
**GO:0007165**	signal transduction	4	41	811	11484	0.328303
**GO:0006955**	immune response	2	41	329	11484	0.329172
**GO:0005739**	mitochondrion	4	41	818	11484	0.334105
**GO:0007420**	brain development	1	41	115	11484	0.338579
**GO:0008219**	cell death	1	41	116	11484	0.340964
**GO:0016310**	phosphorylation	1	41	121	11484	0.352765

**GO:0016607**	nuclear speck	1	41	122	11484	0.355101
**GO:0006355**	regulation of transcription, DNA-templated	5	41	1101	11484	0.356713
**GO:0005764**	lysosome	1	41	132	11484	0.378007
**GO:0016020**	membrane	8	41	1928	11484	0.381723
**GO:0001701**	in utero embryonic development	1	41	137	11484	0.38916
**GO:0007166**	cell surface receptor signaling pathway	1	41	138	11484	0.391367
**GO:0016874**	ligase activity	1	41	138	11484	0.391367
**GO:0007067**	mitotic nuclear division	1	41	140	11484	0.395758
**GO:0005654**	nucleoplasm	3	41	647	11484	0.409059
**GO:0006412**	translation	1	41	152	11484	0.421462
**GO:0055114**	oxidation-reduction process	2	41	401	11484	0.421906
**GO:0010467**	gene expression	2	41	405	11484	0.426891
**GO:0005215**	transporter activity	1	41	160	11484	0.438002
**GO:0005874**	microtubule	1	41	173	11484	0.463901
**GO:0003676**	nucleic acid binding	3	41	712	11484	0.471534
**GO:0019899**	enzyme binding	1	41	177	11484	0.471633
**GO:0007411**	axon guidance	1	41	179	11484	0.475458
**GO:0043065**	positive regulation of apoptotic process	1	41	184	11484	0.484902
**GO:0003674**	molecular_function	2	41	458	11484	0.490813
**GO:0007264**	small GTPase mediated signal transduction	1	41	197	11484	0.508689
**GO:0006200**	ATP catabolic process	1	41	198	11484	0.510474
**GO:0016567**	protein ubiquitination	1	41	198	11484	0.510474
**GO:0005813**	centrosome	1	41	200	11484	0.514024
**GO:0006954**	inflammatory response	1	41	210	11484	0.531403
**GO:0008283**	cell proliferation	1	41	217	11484	0.543206
**GO:0005488**	binding	1	41	218	11484	0.544868
**GO:0006357**	regulation of transcription from RNA polymerase II promoter	1	41	218	11484	0.544868
**GO:0008285**	negative regulation of cell proliferation	1	41	238	11484	0.5769
**GO:0046982**	protein heterodimerization activity	1	41	241	11484	0.581511
**GO:0004713**	protein tyrosine kinase activity	1	41	263	11484	0.61386
**GO:0030154**	cell differentiation	1	41	269	11484	0.62225
**GO:0008284**	positive regulation of cell proliferation	1	41	277	11484	0.633161
**GO:0044267**	cellular protein metabolic process	1	41	299	11484	0.661605
**GO:0042802**	identical protein binding	1	41	300	11484	0.662845
**GO:0005575**	cellular_component	1	41	319	11484	0.685588
**GO:0007275**	multicellular organismal development	1	41	323	11484	0.690182

**GO:0000139**	Golgi membrane	1	41	324	11484	0.69132
**GO:0006810**	transport	1	41	326	11484	0.693584
**GO:0044822**	poly(A) RNA binding	2	41	667	11484	0.697069
**GO:0016032**	viral process	1	41	336	11484	0.704663
**GO:0048471**	perinuclear region of cytoplasm	1	41	342	11484	0.711122
**GO:0000122**	negative regulation of transcription from RNA polymerase II promoter	1	41	349	11484	0.718483
**GO:0003824**	catalytic activity	1	41	356	11484	0.725661
**GO:0005886**	plasma membrane	7	41	2269	11484	0.726909
**GO:0008270**	zinc ion binding	2	41	744	11484	0.754101
**GO:0006915**	apoptotic process	1	41	386	11484	0.754455
**GO:0008150**	biological_process	1	41	400	11484	0.766861
**GO:0055085**	transmembrane transport	1	41	435	11484	0.795262
**GO:0005789**	endoplasmic reticulum membrane	1	41	436	11484	0.796022
**GO:0005783**	endoplasmic reticulum	1	41	446	11484	0.80347
**GO:0045944**	positive regulation of transcription from RNA polymerase II promoter	1	41	467	11484	0.818256
**GO:0016021**	integral component of membrane	4	41	2786	11484	0.994997

Supplementary Table S5B.

KEGG analyses of the dysregulated mRNAs, miRNA targets and lncRNA targets

KEGG analyses of the dysregulated mRNAs

**Pathway Id**	**pathway_name**	**S.gene.number**	**TS.gene.number**	**B.gene.number**	**TB.gene.number**	**p value**
**ko05144**	Malaria	10	135	43	5298	7.75E-08
**ko04610**	Complement and coagulation cas	10	135	63	5298	3.35E-06
**ko05020**	Prion diseases	7	135	32	5298	1.18E-05
**ko05143**	African trypanosomiasis	7	135	32	5298	1.18E-05
**ko05410**	Hypertrophic cardiomyopathy (H	9	135	72	5298	7.57E-05
**ko05323**	Rheumatoid arthritis	9	135	78	5298	0.000142
**ko04260**	Cardiac muscle contraction	8	135	64	5298	0.000192
**ko05414**	Dilated cardiomyopathy	8	135	79	5298	0.000822
**ko05416**	Viral myocarditis	7	135	63	5298	0.001005
**ko05142**	Chagas disease (American trypa	8	135	87	5298	0.001552
**ko04621**	NOD-like receptor signaling path	6	135	53	5298	0.002102
**ko05146**	Amoebiasis	8	135	93	5298	0.00238
**ko05164**	Influenza A	10	135	150	5298	0.004668
**ko04060**	Cytokine-cytokine receptor intera	13	135	237	5298	0.006994
**ko04512**	ECM-receptor interaction	6	135	73	5298	0.010304
**ko04530**	Tight junction	8	135	125	5298	0.013948
**ko04115**	p53 signaling pathway	5	135	61	5298	0.018957

**ko04612**	Antigen processing and presenta	5	135	62	5298	0.020211
**ko04978**	Mineral absorption	4	135	46	5298	0.028807
**ko04620**	Toll-like receptor signaling pathw	6	135	92	5298	0.029226
**ko04010**	MAPK signaling pathway	11	135	239	5298	0.040442
**ko04062**	Chemokine signaling pathway	8	135	163	5298	0.055456
**ko05010**	Alzheimer's disease	7	135	135	5298	0.055922
**ko05322**	Systemic lupus erythematosus	6	135	110	5298	0.060891
**ko03018**	RNA degradation	4	135	61	5298	0.069059
**ko05219**	Bladder cancer	3	135	39	5298	0.076072
**ko04110**	Cell cycle	6	135	117	5298	0.077201
**ko04510**	Focal adhesion	8	135	180	5298	0.087594
**ko00760**	Nicotinate and nicotinamide meta	2	135	22	5298	0.106935
**ko04070**	Phosphatidylinositol signaling sys	4	135	72	5298	0.110509
**ko04270**	Vascular smooth muscle contrac	5	135	100	5298	0.110819
**ko05150**	Staphylococcus aureus infection	3	135	47	5298	0.116926
**ko04744**	Phototransduction	2	135	24	5298	0.123702
**ko04145**	Phagosome	6	135	134	5298	0.126042
**ko05216**	Thyroid cancer	2	135	26	5298	0.141051
**ko00330**	Arginine and proline metabolism	3	135	52	5298	0.145815
**ko04380**	Osteoclast differentiation	5	135	110	5298	0.148337
**ko04623**	Cytosolic DNA-sensing pathway	3	135	53	5298	0.151854
**ko05162**	Measles	5	135	118	5298	0.18174
**ko00250**	Alanine, aspartate and glutamate	2	135	31	5298	0.186334
**ko05332**	Graft-versus-host disease	2	135	32	5298	0.195631
**ko04912**	GnRH signaling pathway	4	135	90	5298	0.196129
**ko05120**	Epithelial cell signaling in Helicob	3	135	60	5298	0.196141
**ko05140**	Leishmaniasis	3	135	60	5298	0.196141
**ko05214**	Glioma	3	135	60	5298	0.196141
**ko00270**	Cysteine and methionine metabol	2	135	34	5298	0.21439
**ko05412**	Arrhythmogenic right ventricular	3	135	64	5298	0.22273
**ko05152**	Tuberculosis	6	135	160	5298	0.22274
**ko04940**	Type I diabetes mellitus	2	135	36	5298	0.233309
**ko03010**	Ribosome	3	135	72	5298	0.277722
**ko04670**	Leukocyte transendothelial migra	4	135	106	5298	0.28452
**ko00360**	Phenylalanine metabolism	1	135	13	5298	0.285331
**ko04630**	Jak-STAT signaling pathway	5	135	142	5298	0.295021
**ko04640**	Hematopoietic cell lineage	3	135	76	5298	0.305744
**ko05145**	Toxoplasmosis	4	135	111	5298	0.313401
**ko00561**	Glycerolipid metabolism	2	135	45	5298	0.31888
**ko05160**	Hepatitis C	4	135	115	5298	0.336706
**ko04614**	Renin-angiotensin system	1	135	16	5298	0.338721
**ko04012**	ErbB signaling pathway	3	135	81	5298	0.340924
**ko04970**	Salivary secretion	3	135	81	5298	0.340924
**ko04020**	Calcium signaling pathway	5	135	152	5298	0.345613
**ko04144**	Endocytosis	6	135	189	5298	0.350658
**ko00100**	Steroid biosynthesis	1	135	17	5298	0.355622

**ko04910**	Insulin signaling pathway	4	135	122	5298	0.377626
**ko05210**	Colorectal cancer	2	135	55	5298	0.411146
**ko00910**	Nitrogen metabolism	1	135	21	5298	0.419045
**ko04710**	Circadian rhythm - mammal	1	135	21	5298	0.419045
**ko04950**	Maturity onset diabetes of the yo	1	135	21	5298	0.419045
**ko00982**	Drug metabolism - cytochrome P	2	135	63	5298	0.480439
**ko04622**	RIG-I-like receptor signaling patl	2	135	63	5298	0.480439
**ko04720**	Long-term potentiation	2	135	63	5298	0.480439
**ko04350**	TGF-beta signaling pathway	2	135	64	5298	0.488753
**ko00340**	Histidine metabolism	1	135	26	5298	0.489676
**ko00410**	beta-Alanine metabolism	1	135	26	5298	0.489676
**ko03320**	PPAR signaling pathway	2	135	65	5298	0.496983
**ko04810**	Regulation of actin cytoskeleton	5	135	182	5298	0.497357
**ko05220**	Chronic myeloid leukemia	2	135	66	5298	0.50513
**ko04971**	Gastric acid secretion	2	135	67	5298	0.513192
**ko03040**	Spliceosome	3	135	108	5298	0.523201
**ko04141**	Protein processing in endoplasmi	4	135	149	5298	0.530234
**ko04142**	Lysosome	3	135	111	5298	0.541862
**ko00564**	Glycerophospholipid metabolism	2	135	73	5298	0.55973
**ko00051**	Fructose and mannose metabolis	1	135	32	5298	0.563265
**ko00600**	Sphingolipid metabolism	1	135	32	5298	0.563265
**ko04514**	Cell adhesion molecules (CAMs)	3	135	117	5298	0.577907
**ko05222**	Small cell lung cancer	2	135	76	5298	0.581795
**ko05340**	Primary immunodeficiency	1	135	34	5298	0.585372
**ko00350**	Tyrosine metabolism	1	135	35	5298	0.596006
**ko04672**	Intestinal immune network for Ig	1	135	36	5298	0.606369
**ko05200**	Pathways in cancer	7	135	290	5298	0.614544
**ko00860**	Porphyrin and chlorophyll metabo	1	135	38	5298	0.626309
**ko04962**	Vasopressin-regulated water rea	1	135	40	5298	0.645247
**ko04930**	Type II diabetes mellitus	1	135	42	5298	0.663231
**ko04916**	Melanogenesis	2	135	89	5298	0.667997
**ko02010**	ABC transporters	1	135	43	5298	0.671881
**ko04310**	Wnt signaling pathway	3	135	135	5298	0.674934
**ko04330**	Notch signaling pathway	1	135	44	5298	0.68031
**ko04150**	mTOR signaling pathway	1	135	46	5298	0.696529
**ko04340**	Hedgehog signaling pathway	1	135	46	5298	0.696529
**ko05213**	Endometrial cancer	1	135	48	5298	0.711932
**ko00562**	Inositol phosphate metabolism	1	135	50	5298	0.726557
**ko05221**	Acute myeloid leukemia	1	135	52	5298	0.740446
**ko04114**	Oocyte meiosis	2	135	103	5298	0.744307
**ko00590**	Arachidonic acid metabolism	1	135	53	5298	0.747125
**ko00830**	Retinol metabolism	1	135	55	5298	0.759976
**ko05131**	Shigellosis	1	135	55	5298	0.759976
**ko05218**	Melanoma	1	135	56	5298	0.766157
**ko00010**	Glycolysis / Gluconeogenesis	1	135	58	5298	0.778047
**ko00980**	Metabolism of xenobiotics by cyt	1	135	60	5298	0.789337

**ko04722**	Neurotrophin signaling pathway	2	135	114	5298	0.793338
**ko04920**	Adipocytokine signaling pathway	1	135	61	5298	0.794767
**ko04724**	Glutamatergic synapse	2	135	115	5298	0.79736
**ko05100**	Bacterial invasion of epithelial ce	1	135	65	5298	0.81513
**ko04976**	Bile secretion	1	135	67	5298	0.824547
**ko04520**	Adherens junction	1	135	69	5298	0.833487
**ko04662**	B cell receptor signaling pathway	1	135	70	5298	0.837786
**ko04146**	Peroxisome	1	135	72	5298	0.846056
**ko03013**	RNA transport	2	135	132	5298	0.855909
**ko04210**	Apoptosis	1	135	75	5298	0.857683
**ko04974**	Protein digestion and absorption	1	135	76	5298	0.861361
**ko04540**	Gap junction	1	135	77	5298	0.864945
**ko05215**	Prostate cancer	1	135	81	5298	0.878385
**ko04666**	Fc gamma R-mediated phagocyt	1	135	85	5298	0.890496
**ko04972**	Pancreatic secretion	1	135	90	5298	0.903964
**ko04660**	T cell receptor signaling pathway	1	135	96	5298	0.917972
**ko04725**	Cholinergic synapse	1	135	98	5298	0.922175
**ko04120**	Ubiquitin mediated proteolysis	1	135	119	5298	0.955254
**ko04650**	Natural killer cell mediated cytot	1	135	125	5298	0.961815
**ko05016**	Huntington's disease	1	135	148	5298	0.979242
**ko00230**	Purine metabolism	1	135	150	5298	0.980316
**ko04080**	Neuroactive ligand-receptor inte	2	135	235	5298	0.984993
**ko01100**	Metabolic pathways	14	135	998	5298	0.997723
**ko04740**	Olfactory transduction	2	135	373	5298	0.999471

KEGG analyses of the dysregulated miRNA targets

**Pathway Id**	pathway_name	S.gene. number	TS.gene. number	B.gene. number	TB.gene. number	p value
**ko04610**	Complement and coagulation cas	4	58	98	11403	0.001526
**ko05010**	Alzheimer's disease	2	58	24	11403	0.006531
**ko03018**	RNA degradation	3	58	80	11403	0.007777
**ko04260**	Cardiac muscle contraction	2	58	27	11403	0.008225
**ko04917**	Prolactin signaling pathway	1	58	5	11403	0.025179
**ko05020**	Prion diseases	1	58	7	11403	0.035075
**ko04668**	TNF signaling pathway	2	58	60	11403	0.037281
**ko00982**	Drug metabolism - cytochrome P	1	58	8	11403	0.039986
**ko04012**	ErbB signaling pathway	2	58	81	11403	0.063808
**ko04020**	Calcium signaling pathway	3	58	197	11403	0.078585
**ko04710**	Circadian rhythm	1	58	18	11403	0.087765
**ko00051**	Fructose and mannose metabolis	1	58	25	11403	0.119811
**ko04810**	Regulation of actin cytoskeleton	2	58	120	11403	0.124296
**ko00100**	Steroid biosynthesis	1	58	30	11403	0.142021
**ko05166**	HTLV-I infection	1	58	30	11403	0.142021
**ko05016**	Huntington's disease	1	58	33	11403	0.155082
**ko00270**	Cysteine and methionine metabol	1	58	34	11403	0.159392
**ko04340**	Hedgehog signaling pathway	1	58	39	11403	0.180621
**ko04022**	cGMP - PKG signaling pathway	1	58	40	11403	0.184803

**ko04512**	ECM-receptor interaction	1	58	42	11403	0.193104
**ko04151**	PI3K-Akt signaling pathway	2	58	160	11403	0.195515
**ko05206**	MicroRNAs in cancer	1	58	44	11403	0.201322
**ko04978**	Mineral absorption	1	58	45	11403	0.2054
**ko04380**	Osteoclast differentiation	1	58	48	11403	0.217512
**ko00600**	Sphingolipid metabolism	1	58	69	11403	0.297361
**ko04630**	Jak-STAT signaling pathway	2	58	220	11403	0.308431
**ko00561**	Glycerolipid metabolism	1	58	77	11403	0.325625
**ko03013**	RNA transport	2	58	250	11403	0.364378
**ko04064**	NF-kappa B signaling pathway	1	58	104	11403	0.413011
**ko04068**	FoxO signaling pathway	1	58	112	11403	0.436695
**ko04390**	Hippo signaling pathway	1	58	113	11403	0.439588
**ko04060**	Cytokine-cytokine receptor intera	1	58	142	11403	0.517433
**ko04010**	MAPK signaling pathway	2	58	350	11403	0.535491
**ko04310**	Wnt signaling pathway	1	58	161	11403	0.562565
**ko01200**	Carbon metabolism	1	58	179	11403	0.601482
**ko04015**	Rap1 signaling pathway	1	58	183	11403	0.609657
**ko04014**	Ras signaling pathway	2	58	405	11403	0.615716
**ko03010**	Ribosome	1	58	193	11403	0.62938
**ko04514**	Cell adhesion molecules (CAMs)	1	58	222	11403	0.681203
**ko04120**	Ubiquitin mediated proteolysis	1	58	242	11403	0.712723
**ko00230**	Purine metabolism	1	58	320	11403	0.80893

KEGG analyses of the dysregulated lncRNA targets

**Pathway Id**	pathway_name	S.gene. number	TS.gene. number	B.gene. number	TB.gene. number	p value
**ko05144**	Malaria	5	45	43	5298	2.66E-05
**ko05143**	African trypanosomiasis	4	45	32	5298	0.000137
**ko04610**	Complement and coagulation cas	4	45	63	5298	0.001878
**ko04260**	Cardiac muscle contraction	4	45	64	5298	0.001991
**ko05410**	Hypertrophic cardiomyopathy (H	4	45	72	5298	0.003068
**ko05414**	Dilated cardiomyopathy	4	45	79	5298	0.004292
**ko05142**	Chagas disease (American trypa	4	45	87	5298	0.006053
**ko04115**	p53 signaling pathway	3	45	61	5298	0.014624
**ko05323**	Rheumatoid arthritis	3	45	78	5298	0.027982
**ko05020**	Prion diseases	2	45	32	5298	0.029775
**ko05150**	Staphylococcus aureus infection	2	45	47	5298	0.059921
**ko05322**	Systemic lupus erythematosus	3	45	110	5298	0.065908
**ko00330**	Arginine and proline metabolism	2	45	52	5298	0.071585
**ko04110**	Cell cycle	3	45	117	5298	0.076292
**ko04530**	Tight junction	3	45	125	5298	0.088995
**ko04612**	Antigen processing and presenta	2	45	62	5298	0.096857
**ko05416**	Viral myocarditis	2	45	63	5298	0.09951
**ko04145**	Phagosome	3	45	134	5298	0.104284
**ko03010**	Ribosome	2	45	72	5298	0.124253
**ko04512**	ECM-receptor interaction	2	45	73	5298	0.127089
**ko05164**	Influenza A	3	45	150	5298	0.13382

**ko04060**	Cytokine-cytokine receptor intera	4	45	237	5298	0.140252
**ko04062**	Chemokine signaling pathway	3	45	163	5298	0.159745
**ko04710**	Circadian rhythm - mammal	1	45	21	5298	0.164288
**ko00760**	Nicotinate and nicotinamide meta	1	45	22	5298	0.171415
**ko04620**	Toll-like receptor signaling pathw	2	45	92	5298	0.183483
**ko05146**	Amoebiasis	2	45	93	5298	0.186554
**ko04510**	Focal adhesion	3	45	180	5298	0.19577
**ko05216**	Thyroid cancer	1	45	26	5298	0.199332
**ko04380**	Osteoclast differentiation	2	45	110	5298	0.239687
**ko04142**	Lysosome	2	45	111	5298	0.242849
**ko00270**	Cysteine and methionine metabol	1	45	34	5298	0.252434
**ko05340**	Primary immunodeficiency	1	45	34	5298	0.252434
**ko05160**	Hepatitis C	2	45	115	5298	0.255518
**ko00860**	Porphyrin and chlorophyll metabo	1	45	38	5298	0.277678
**ko04910**	Insulin signaling pathway	2	45	122	5298	0.277729
**ko05219**	Bladder cancer	1	45	39	5298	0.283857
**ko04930**	Type II diabetes mellitus	1	45	42	5298	0.302088
**ko02010**	ABC transporters	1	45	43	5298	0.308063
**ko04150**	mTOR signaling pathway	1	45	46	5298	0.32569
**ko04978**	Mineral absorption	1	45	46	5298	0.32569
**ko00562**	Inositol phosphate metabolism	1	45	50	5298	0.348512
**ko04621**	NOD-like receptor signaling path	1	45	53	5298	0.36513
**ko04623**	Cytosolic DNA-sensing pathway	1	45	53	5298	0.36513
**ko05218**	Melanoma	1	45	56	5298	0.381334
**ko05140**	Leishmaniasis	1	45	60	5298	0.402312
**ko05214**	Glioma	1	45	60	5298	0.402312
**ko04920**	Adipocytokine signaling pathway	1	45	61	5298	0.407447
**ko04622**	RIG-I-like receptor signaling path	1	45	63	5298	0.417587
**ko05220**	Chronic myeloid leukemia	1	45	66	5298	0.432481
**ko05200**	Pathways in cancer	3	45	290	5298	0.450529
**ko04070**	Phosphatidylinositol signaling sys	1	45	72	5298	0.461159
**ko04810**	Regulation of actin cytoskeleton	2	45	182	5298	0.461281
**ko04974**	Protein digestion and absorption	1	45	76	5298	0.479485
**ko05222**	Small cell lung cancer	1	45	76	5298	0.479485
**ko04012**	ErbB signaling pathway	1	45	81	5298	0.501537
**ko05215**	Prostate cancer	1	45	81	5298	0.501537
**ko04912**	GnRH signaling pathway	1	45	90	5298	0.538953
**ko04270**	Vascular smooth muscle contrac	1	45	100	5298	0.577308
**ko04670**	Leukocyte transendothelial migra	1	45	106	5298	0.598804
**ko04010**	MAPK signaling pathway	2	45	239	5298	0.609539
**ko05145**	Toxoplasmosis	1	45	111	5298	0.615898
**ko04120**	Ubiquitin mediated proteolysis	1	45	119	5298	0.641778
**ko03013**	RNA transport	1	45	132	5298	0.68024
**ko04630**	Jak-STAT signaling pathway	1	45	142	5298	0.707049
**ko04141**	Protein processing in endoplasmi	1	45	149	5298	0.724494
**ko04020**	Calcium signaling pathway	1	45	152	5298	0.731656

**ko05152**	Tuberculosis	1	45	160	5298	0.749876
**ko04080**	Neuroactive ligand-receptor inter	1	45	235	5298	0.871314
**ko01100**	Metabolic pathways	5	45	998	5298	0.944499

Supplementary Table S6. ROC curve analyses of the selected RNAs

Test Result Variable(s)	Tissue	Are*	Std. Error*	Asymptotic Sig.**	Asymptotic 95% Confidence Interval	
					Lower Bound	Upper Bound
miR-6089	muscle	0.814	0.042	<0.0001	0.725	0.884
miR-27b-3p	muscle	0.94	0.027	<0.0001	0.875	0.977
miR-214-3p	muscle	0.648	0.056	0.0082	0.548	0.74
miR-150-5p	muscle	0.774	0.05	<0.0001	0.681	0.851
let-7e-5p	muscle	0.841	0.044	<0.0001	0.755	0.905
miR-145-5p	muscle	0.692	0.053	0.0003	0.594	0.78
LINC01405	muscle	0.923	0.025	<0.0001	0.853	0.966
SNHG12	muscle	0.924	0.027	<0.0001	0.855	0.967
RP11-403P17.4	muscle	0.918	0.026	<0.0001	0.847	0.963
CTC-260E6.6	muscle	0.872	0.035	<0.0001	0.791	0.929
RP11-357D18.1	muscle	0.742	0.054	<0.0001	0.646	0.823
miR-27b-3p	serum	0.879	0.037	<0.0001	0.806	0.952
lactate	serum	0.841	0.04	<0.0001	0.762	0.919

*Under the nonparametric assumption
**Null hypothesis: true area = 0.5

Supplementary Table S7. The primer sequences of selected lncRNAs and mRNAs for qPCR

Name of genes	Forward primer (5′-3′)	Reverse primer (5′-3′)
PDK4	CCTTTGGCTGGTTTTGGTTA	CCTGCTTGGGATACACCAGT
CDKN1A	ATGAAATTCACCCCCTTTCC	CCCTAGGCTGTGCTCACTTC
ATP2A2	ACCCACATTCGAGTTGGAAG	CCAACGAAGGTCAGATTGGT
SOD3	CTGGGTGCAGCTCTCTTTTC	ACATGTCTCGGATCCACTCC
DDIT4	TGTTTAGCTCCGCCAACTCT	CACCCCAAAAGTTCAGTCGT
MKNK2	TGGAGATGCTGTACCAGTGC	AGAGGATGTTTTCCGGCTTT
LINC01405	AAGGTGCTTGTTTCCCCTTT	AAAGATGTTCCTCGCCCTCT
SNHG12	AAACGGTCCCATCAAGACTG	GTCCCCTGCATTTCATCACT
RP11-403P17.4	AAGCAAGTGGAAAGGCAGAA	GAGAGACGCCTCAGAACCAC
CTC-260E6.6	GTCTGGGACCATTGCAGTTT	CCGTGCTTGCTACCAGAAAT
RP11-357D18.1	TTCTGCCTGCTTATGCAAGAT	ACAGGATGGCTGCTAGGATG
GAPDH	TGTTGCCATCAATGACCCCTT	CTCCACGACGTACTCAGCG
CTC-260E6.6	GTCTGGGACCATTGCAGTTT	CCGTGCTTGCTACCAGAAAT
RP11-357D18.1	TTCTGCCTGCTTATGCAAGAT	ACAGGATGGCTGCTAGGATG
GAPDH	TGTTGCCATCAATGACCCCTT	CTCCACGACGTACTCAGCG
